# Chiral Bismuth‐Rhodium Paddlewheel Complexes Empowered by London Dispersion: The C−H Functionalization Nexus

**DOI:** 10.1002/anie.202212546

**Published:** 2022-10-11

**Authors:** Michael Buchsteiner, Santanu Singha, Jonathan Decaens, Alois Fürstner

**Affiliations:** ^1^ Max-Planck-Institut für Kohlenforschung 45470 Mülheim/Ruhr Germany

**Keywords:** Asymmetric Catalysis, C−H Functionalization, Heterobimetallic Complexes, London Dispersion, Rhodium Carbenes

## Abstract

Heterobimetallic [BiRh] tetracarboxylate catalysts endowed with 1,3‐disilylated phenylglycine paddlewheels benefit from interligand London dispersion. They were originally designed for asymmetric cyclopropanation but are now shown to perform very well in asymmetric C−H functionalization reactions too. Because of the confined ligand sphere about the derived donor/acceptor carbenes, insertions into unhindered methyl groups are kinetically favored, although methylene units also react with excellent levels of asymmetric induction; even gaseous ethane is a suitable substrate. Moreover, many functional groups in both partners are tolerated. The resulting products are synthetically equivalent to the outcome of traditional asymmetric ester alkylation, allylation, benzylation, propargylation and aldol reactions and therefore constitute a valuable nexus to more conventional chemical logic.

## Graphical abstract

The encounter of a donor/acceptor diazo derivative with a hydrocarbon inside the chiral binding pocket crafted by London dispersion about a heterobimetallic [BiRh] paddlewheel complex entails highly effective asymmetric C−H insertion; the resulting products formally correspond to the outcome of classical ester alkylation, allylation, benzylation, or aldol reactions.

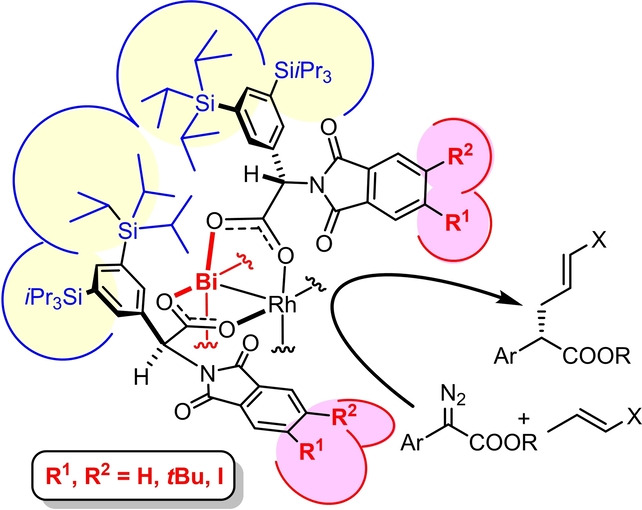

## Introduction

**Figure 1:**
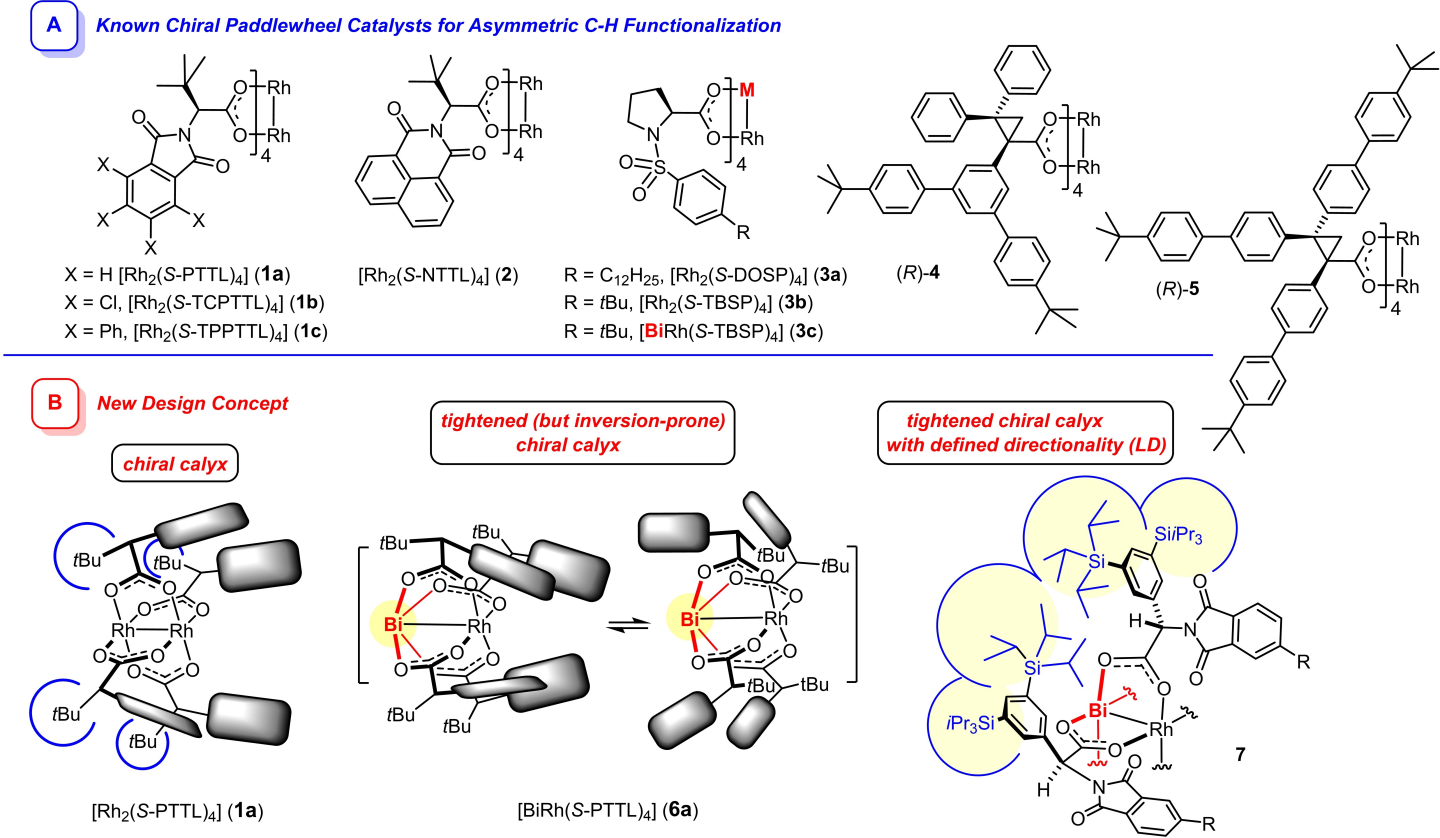
Two conceptually different approaches to chiral paddlewheel complexes for asymmetric catalysis, see Text; the paddles shown in panel **B** denote the phthalimido substituents of the PTTL ligands of **1 a** and **6 a**; in **7**, only two of the four chiral ligands are drawn for the sake of clarity

Despite tremendous progress in the field of catalytic C−H functionalization in general,^[1]^ very few *undirected* transformations of this type have reached strategy‐level status, especially when it comes to the use of aliphatic substrates.^[2]^ The insertion of “super‐electrophilic” rhodium carbenes into aliphatic C−H bonds arguably belongs to this exclusive category.[^3^, ^4^] While the pioneering studies had largely focused on entropically favored intramolecular settings,[^5^, ^6^] (asymmetric) intermolecular reactions of this type are rapidly making up ground.[^7^, ^8^] They likely proceed in a concerted yet non‐synchronous manner: the electrophilic carbene center is attacked by the H‐atom in question that develops notable hydridic character in a transition state characterized by an obtuse C−H−C angle.[^9^, ^10^, ^11^, ^12^] In line with such a “hydride transfer” pathway, insertions are intrinsically favored at sites able to stabilize the complementary positive charge at carbon (allylic, benzylic, α to oxygen or nitrogen; tertiary ≥ secondary > primary).[^8c^, ^13^] With the availability of ever more congested chiral rhodium complexes, however, it was recognized that this inherent electronic bias can be counterbalanced and eventually overruled by steric effects (Figure 1A). This development culminated in the development of a set of catalysts such as **1 b**, **1 c**, **4** and **5** with mutually orthogonal preferences, which allow for functionalization of a given aliphatic hydrocarbon at either the tertiary *or* the secondary *or* even the least activated primary C−H bonds with good to excellent levels of regio‐, diastereo‐, and enantioselectivity in many cases.[^14^, ^15^, ^16^, ^17^]

## Results and Discussion

This success is largely due to the availability of ever more sophisticated chiral homobimetallic dirhodium paddlewheel catalysts (Figure 1A).[^18^, ^19^, ^20^] In a rare comparative study, [Rh_2_((*S)‐*TBSP)_4_] (**3 b**) was found to score significantly better than its heterobimetallic cousin [BiRh((*S)‐*TBSP)_4_] (**3 c**) in prototype C−H functionalization reactions.[^21^, ^22^] Although this evidence suggests that incorporation of the main group element into the bimetallic core is counterproductive, our excellent experiences with a new type of [BiRh] paddlewheel catalysts led us reconsider this notion. Specifically, complexes **7 a** (R=H) and **7 b** (R=*t*Bu) were recently shown to perform exceedingly well in asymmetric [2+1] cycloaddition reactions, furnishing diversely functionalized cyclopropanes and cyclopropenes in excellent optical purity and high yield with largely unparalleled reaction rates at low catalyst loadings.[^23^, ^24^] These powerful tools formally descend from the classical catalyst [Rh_2_((*S)‐*PTTL)_4_] (**1 a**) originally developed by the Hashimoto group (Figure 1B):^[25]^ In a first step, one of the two rhodium atoms was deliberately replaced by a larger Bi(+2) center in order to impart a conical shape onto the chiral ligand sphere,[^26^, ^27^, ^28^] which adopts an α,α,α,α‐conformation in the stereodetermining transition state.[^29^, ^30^] As long as the narrower pore surrounds the catalytically active Rh(+2) site of [BiRh((*S)‐*PTTL)_4_] (**6 a**), improved asymmetric induction will ensue.^[31]^ The directionality, however, is a derivative of the ligand structure and cannot be taken for granted. To warrant this critically important parameter, the *tert*‐butyl residues in **6 a** were replaced in a next design step by phenyl rings carrying lateral −Si(*i*Pr)_3_ substituents, which get close enough to each other to entertain a large number of attractive interligand London dispersion (LD) interactions[^32^, ^33^] that stabilize the desired calyx of the resulting complex **7 a** (R=H).[^23^, ^34^] Finally, it was noticed that *tert*‐butyl substituents on each of the phthalimido paddles^[35]^ in **7 b** (R=*t*Bu) further confine the chiral pocket, again due to supplementary LD between these groups, and hence entail even higher ee's.^[23]^

**Scheme 1:**
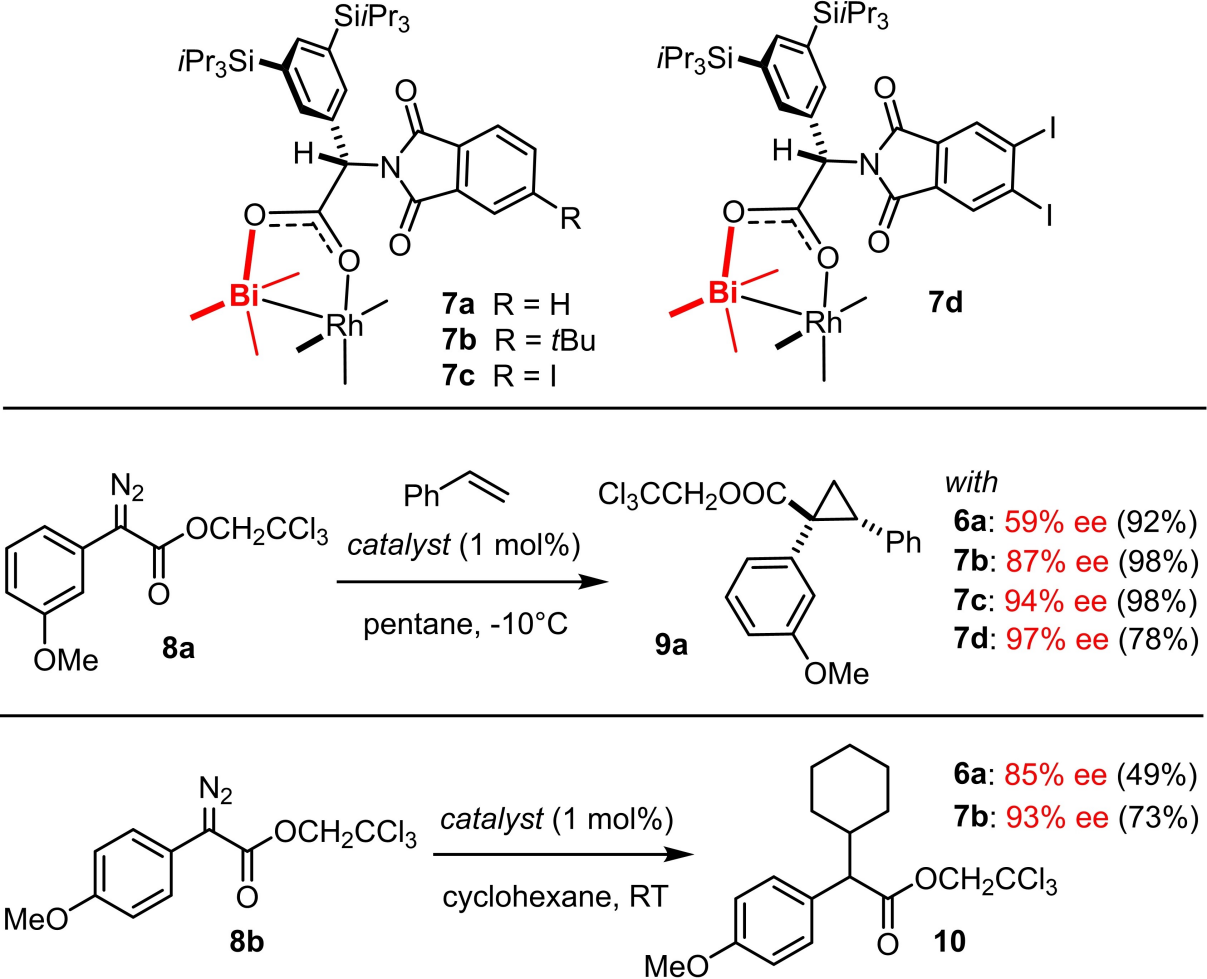
Known and new heterobimetallic paddlewheel complexes benefitting from LD; only one of the four identical chiral ligands of complexes **7** is drawn for clarity

Based on this rationale, it is reasonable to expect that polarizable substituents on the rim other than *tert*‐butyl groups provide further opportunities for optimization. To this end, we now present complexes **7 c** and **7 d** bearing one or two iodides on the phthalimido groups, respectively (Scheme 1; for their synthesis, see the Supporting Information). As expected, these new catalysts also perform remarkably well, on bar with or even superior to **7 b**, as witnessed by the formation of cyclopropane **9 a**. This particular test reaction was chosen because it had been the least enantioselective of the 32 examples contained in our previous report on asymmetric [2+1] cycloaddition with the aid of catalyst **7 b**:^[23]^ while **7 b** afforded “only” 87 % ee, the new iodinated variants **7 c** (94 % ee) and **7 d** (97 % ee) perform notably better in this demanding case. In any event, our new type of heterobimetallic [BiRh] complexes empowered by London dispersion ranks amongst the most effective and selective catalysts known to date for asymmetric cyclopropanation/cyclopropenation of terminal alkenes/alkynes with donor/acceptor diazo derivatives.^[23]^ Their ability to effect intermolecular C−H insertion, however, was illustrated only by a single example, in which the α‐diazoester **8 b** was reacted with cyclohexane using **7 b** as the catalyst to give product **10** in 73 % yield and 93 % ee (Scheme 1).^[23]^ Although the result is clearly better than that obtained with the first‐generation chiral heterobimetallic complex [BiRh((*S)‐*PTTL)_4_] (**6 a**) (49 %, 85 % ee), it is no more than an encouraging but fairly simple test since cyclohexane as a cyclic hydrocarbon poses no problems with regard to site‐selectivity and can be used in large excess as reagent and solvent at the same time.

As outlined below, both issues are easily overcome. Thus, 2–5 equivalents of the hydrocarbon usually suffice to ensure good yields of the corresponding C−H insertion products (Table 1). With (moderately) activated substrates, the reactions are best performed in pentane as the solvent at −10 °C, with only traces of competing insertion into this medium, if any.^[36]^ 1,4‐Cyclohexadiene and tetrahydrofuran fall into this category, which are electronically predisposed to site‐selective C−H functionalization and unencumbered at the same time (entries 1–4). This bias notwithstanding, the fact that product **13** was formed virtually as a single isomer (dr ≥ 50 : 1 (HPLC), 99 % ee) is remarkable and compares favorably to the literature.^[8c]^

**Table 1:** Asymmetric C−H insertion reactions catalyzed by [BiRh] paddlewheel complexes of type **7**.^[a]^

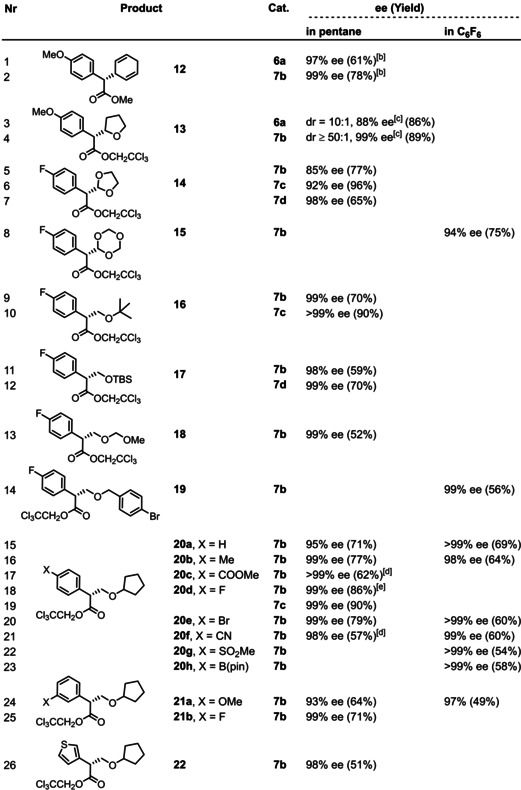
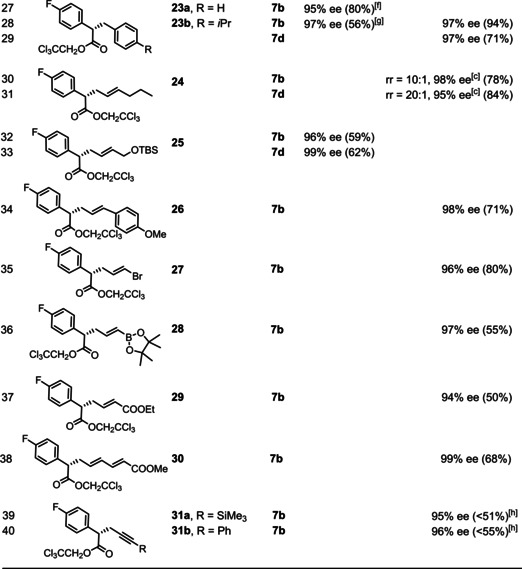

[a] Unless stated otherwise, all reactions were performed with a catalyst loading of 0.5 mol% in either pentane (at −10 °C) or in C_6_F_6_ (at RT); [b] at RT; [c] ee of the major isomer; [d] in pentane/CH_2_Cl_2_ to solubilize the diazo derivative; [e] 500 mg scale using 0.1 mol% of the catalyst; [f] in toluene as reagent and solvent; [g] ca. 30 % of competing insertion into pentane, see Text; [h] the decomposition product **11** could not be fully removed by flash chromatography, see the Supporting Information; rr=ratio of regioisomers.

In case of less activated substrates, however, the pentane solvent may interfere, leading to the formation of side products. Compound **23 b**, for example, was formed upon exclusive functionalization of the least hindered C−H bond of *p*‐cymene and could be isolated in respectable yield (56 %) and no less than 97 % ee (entry 28), but reaction with pentane was substantial too (ca. 30 %).^[36]^ More ambitious insertions into even less activated C−H bonds might require a more inert medium. Inspired by our excellent experience with hexafluorobenzene in asymmetric fluorinations of copper carbenes,^[37]^ we resorted to this particular solvent. Gratifyingly, its use allowed the yield of **23 b** to be significantly improved without compromising the excellent level of asymmetric induction (94 %, 97 % ee), even though the reaction had to be performed at ambient temperature.^[38]^ The new complex **7 d** was equally selective but lower yielding (71 %, 97 % ee): what looks like a handicap at first sight could ultimately turn out to be an asset since the chosen 0.5 mol% of **7 d** did not visibly dissolve in hexafluorobenzene. Only a tiny fraction must account for the reaction, which we were, so far, unable to quantify precisely; therefore, the attained 71 % yield likely stands for a very high turnover number. In view of the price of rhodium, this aspect is relevant and subject to further study.

**Scheme 2:**
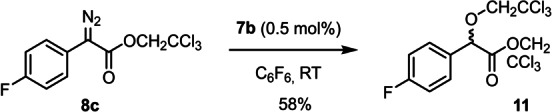
A self‐destructive pathway able to compete with productive C−H insertion.

Collectively, the examples compiled in Table 1 show a remarkable level of asymmetric induction that is basically unaffected by whether pentane or C_6_F_6_ is chosen as the solvent. The impact on the chemical yield, however, is less uniform: while C_6_F_6_ meant a significant boost for **23 b**, we also noted cases in which this medium resulted in diminished yields (see compound **20 b**,**e**, **21 a**). This inconsistency has to do with a previously undescribed self‐destructive pathway of the diazo derivatives which occasionally interferes with the desired C−H functionalization; it becomes the major path if the hydrocarbon substrate is left out, as exemplified by the transformation of **8 c** into **11**, which was racemic independent of the chosen chiral catalyst (Scheme 2). Wherever this process occurs at a competitive rate, it drastically reduces the yield of the desired C−H insertion product or even prevents its formation, because two equivalents of the starting diazo ester are consumed.

This aspect notwithstanding, a number of arguably challenging substrates was found to be well behaved. Specifically, insertion into *t*BuOMe, TBSOMe, cyclopentyl methyl ether, ArCH_2_OMe, and even dimethoxymethane (methylal) occurred exclusively into the primary −OMe group to give the corresponding O‐protected aldol surrogates **16–22** with excellent ee's,^[39]^ independent of whether complex **7 b** or one of the new iodo‐substituted complexes **7 c,d** was chosen as the catalyst. As expected, the reactions scale well and the catalyst loading can be further reduced to 0.1 mol% without loss in yield or optical purity (entry 18). In case of methylal, the observed formation of **18** is particularly striking, as the acetal methylene group should be the favored site of reaction. This expectation seems all the more plausible since 1,3‐dioxolane as well as 1,3,5‐trioxane as cheap formaldehyde surrogates both get functionalized at this position without incident to give products **14** and **15**, respectively; thereby, the new complexes **7 c,d** led to notably higher optical purity than the parent catalyst **7 b** (compare entries 5–7). These examples demonstrate that insertion of the transiently formed metal carbene into a secondary O−CH_2_−O methylene group is not precluded on steric grounds by the bulky ligand sphere of **7**; the site selectivity for methylal leading to the formation of **18** is therefore likely kinetic in origin.

For the same reason, TBS‐protected 2‐buten‐1‐ol reacts almost exclusively at the terminal allylic methyl group to give product **25** (r. r. ≥ 20 : 1, 96 % ee); the already excellent optical purity reached with **7 b** is further improved to 99 % ee with the new complex **7 d** (compare entries 32/33). For comparison: classical catalysts such as [Rh_2_(NTTL)_4_] (**2**) or [Rh_2_(DOSP)_4_] (**3 a**) show the opposite site‐preference in that insertions occur exclusively into the electronically favored −CH_2_‐OTBS group of this substrate;[^40^, ^41^] although [Rh_2_((*S)‐*TPPTTL)_4_] (**1 c**) has previously been recommended for the selective functionalization of methyl groups, this bulky catalyst affords a very modest result with this particular substrate (r. r.=66 : 34, 68 % yield (NMR)).^[40]^ In any case, product **25** now available in optically pure form showcases that catalytic asymmetric C−H functionalization with the aid of catalysts of type **7** is synthetically equivalent to asymmetric allylation of an ester enolate (or, alternatively, an asymmetric Claisen‐type allyl ester rearrangement), just like the formation of **23** equals an asymmetric benzylation reaction.

**Scheme 3:**
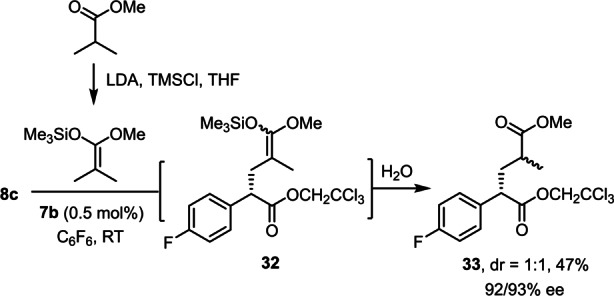
Remote functionalization of an ester via the corresponding silylketene acetal.

The inclination to insert into the primary allylic bond also dominates the reaction with 2‐hexene to give product **24**; the site selectivity was particularly high with **7 d** as the catalyst.^[42]^ It is arguably relevant that the concept of such formal “asymmetric allylation” reactions can be extended to much less activated substrates. Specifically, (*E*)‐1‐methoxy‐4‐(prop‐1‐en‐1‐yl)benzene, (*E*)‐1‐bromo‐1‐propene, (*E*)‐prop‐1‐en‐1‐yl pinacolboronate, and ethyl crotonate^[43]^ afforded the corresponding products **26–29** with impeccable enantioselectivity, despite the fact that the MeCH=CHX unit is conjugated to substituents −X of increasingly electron‐withdrawing character that deactivate the primary C−H bond on electronic grounds (−Ar < −Br < −B(pin) < −COOEt).[^44^, ^45^] The exquisite level of asymmetric induction is retained even in the reaction of doubly‐conjugated methyl (2*E*,4*E*)‐hexa‐2,4‐dienoate, which again reacts exclusively at the methyl terminus to give product **30**.^[43]^ Even formal asymmetric propargylations are possible upon reaction with certain methyl‐capped alkynes, although the reactions are less clean because of competing formation of **11** and the scope is therefore narrow;^[46]^ the optical purity of the resulting products **31 a,b**, however, is again very high. Moreover, we would like to emphasize that even an ordinary ester such as methyl propionate, can be “armed” for functionalization at the β‐position (Scheme 3): thus, the derived silyl ketene acetal behaves like an ordinary allylic substrate vis‐à‐vis the carbene intermediate and affords the corresponding allylation product **33** after hydrolytic work up.^[47]^

**Scheme 4:**
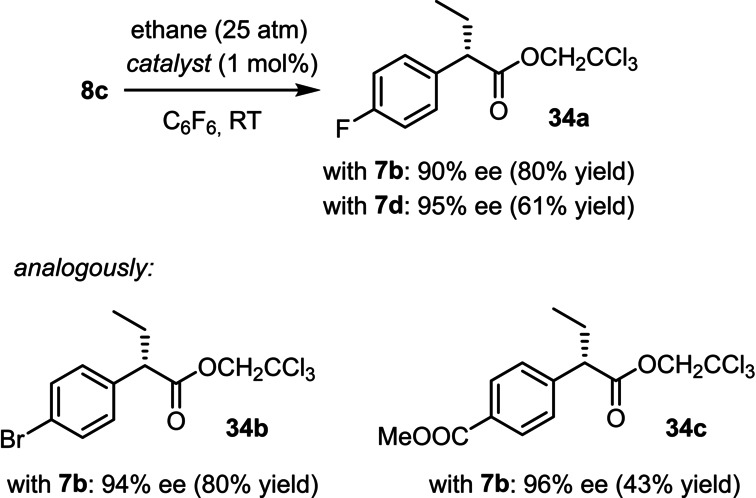
C−H functionalization of ethane.

The good solubility of hydrocarbons in C_6_F_6_ potentially allows gaseous substrates to be engaged in C−H functionalization. Indeed, reactions in an autoclave under an ethane atmosphere (≈ 25 atm) afforded the corresponding alkylation products **34 a–c** in appreciable yields and excellent ee's (Scheme 4).^[48]^ In structural terms, the outcome corresponds to an asymmetric alkylation of an ester enolate, which would require stoichiometric base, (over)stoichiometric amounts of ethyl iodide, and, in the most general set‐up, the use of a chiral auxiliary. The catalytic alternative using ethane as the “alkylating agent” is therefore attractive. For the time being, however, an attempted extension to methane as the substrate met with failure but is subject to further study in this laboratory.^[49]^

**Scheme 5:**
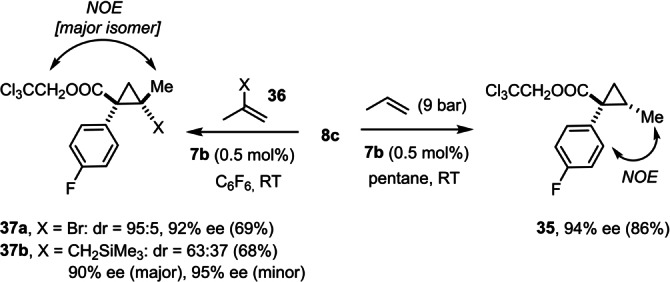
Allylic substrates undergoing cyclopropanation rather than C−H functionalization.

A few other limitations need explicit mentioning too. In line with our earlier findings that complexes **7 a**,**b** catalyze cyclopropanations of terminal alkenes very well, gaseous 1‐propene afforded **35** rather than the product of allylic C−H functionalization (Scheme 5). Likewise, cyclopropanation prevails in case of propene derivatives of type **36** bearing substituents at the internal position, in which the insertion of the bulky chiral carbene intermediate into the terminal C−H bond is obviously impeded by the central branch. Note that (*E*)‐1‐bromo‐1‐propene—the regioisomer of **36 a** (X=Br)—undergoes C−H functionalization at the methyl terminus, as witnessed by the high yielding formation of product **27** (Table 1, entry 35). This comparison shows the subtle balance between the two competing pathways that warrants further investigation.

## Conclusion

Despite these current limitations, catalysts of type **7** allow a variety of substrates bearing valuable synthetic handles for downstream functionalization to be engaged in asymmetric C−H insertion; therefore, they constitute an important link to the established logic and practice of (target‐oriented) synthesis. At the same time, they illustrate the previously unrecognized virtues of heterobimetallic [BiRh] paddlewheel complexes in that levels of asymmetric induction close to the limits of detection are reached in many cases, while ensuring excellent site‐selectivity and respectable yields. Moreover, their performance can be fine‐tuned by lateral modifications, which augurs well for future applications to specific target compounds. In strategic terms, this catalytic methodology represents a valuable nexus between advanced C−H insertion chemistry and more traditional organic synthesis based on the selective manipulation of functional groups.

## Conflict of interest

The authors declare no conflict of interest.

1

## Supporting information

As a service to our authors and readers, this journal provides supporting information supplied by the authors. Such materials are peer reviewed and may be re‐organized for online delivery, but are not copy‐edited or typeset. Technical support issues arising from supporting information (other than missing files) should be addressed to the authors.

Supporting Information

## Data Availability

The data that support the findings of this study are available in the supplementary material of this article.

## References

[1] J.-Q. Yu , Science of Synthesis: Catalytic Transformations via C-H Activation, Thieme, Stuttgart, 2015.

[2] J. F. Hartwig , M. A. Larsen , ACS Cent. Sci. 2016, 2, 281–292. 27294201 10.1021/acscentsci.6b00032 PMC4898263

[3] D. Qiu , J. Wang , Recent Developments of Diazo Compounds in Organic Synthesis, World Scientific, London, 2021.

[4a] A. Ford , H. Miel , A. Ring , C. N. Slattery , A. R. Maguire , M. A. McKervey , Chem. Rev. 2015, 115, 9981–10080; 26284754 10.1021/acs.chemrev.5b00121

[4b] H. M. L. Davies , J. R. Manning , Nature 2008, 451, 417–424; 18216847 10.1038/nature06485 PMC3033428

[4c] M. P. Doyle , R. Duffy , M. Ratnikov , L. Zhou , Chem. Rev. 2010, 110, 704–724; 19785457 10.1021/cr900239n

[4d] H. M. L. Davies , Y. Lian , Acc. Chem. Res. 2012, 45, 923; 22577963 10.1021/ar300013t PMC3378806

[4e] A. DeAngelis , R. Panish , J. M. Fox , Acc. Chem. Res. 2016, 49, 115; 26689221 10.1021/acs.accounts.5b00425 PMC4898907

[4f] P. M. P. Gois , C. A. M. Afonso , Eur. J. Org. Chem. 2004, 3773;

[4g] A. Caballero , M. M. Díaz-Requejo , M. R. Fructos , A. Olmos , J. Urbano , P. J. Pérez , Dalton Trans. 2015, 44, 20295; 26568268 10.1039/c5dt03450g

[4h] Y. He , Z. Huang , K. Wu , J. Ma , Y.-G. Zhou , Z. Yu , Chem. Soc. Rev. 2022, 51, 2759–2852. 35297455 10.1039/d1cs00895a

[5a] D. F. Taber , R. E. Ruckle , J. Am. Chem. Soc. 1986, 108, 7686–7693; 22283275 10.1021/ja00284a037

[5b] G. Stork , N. Kazuhiko , Tetrahedron Lett. 1988, 29, 2283–2286.

[6] The strategic relevance was recognized early on and the transformation therefore soon embraced by the synthetic community; for pioneering applications, see the following for leading references:

[6a] D. E. Cane , P. J. Thomas , J. Am. Chem. Soc. 1984, 106, 5295–5303;

[6b] D. F. Taber , J. L. Schuchardt , J. Am. Chem. Soc. 1985, 107, 5289–5290;

[6c] A. Hinman , J. Du Bois , J. Am. Chem. Soc. 2003, 125, 11510–11511; 13129349 10.1021/ja0368305

[6d] W. Kurosawa , H. Kobayashi , T. Kan , T. Fukuyama , Tetrahedron 2004, 60, 9615–9628;

[6e] H. M. L. Davies , A. M. Walji , Angew. Chem. Int. Ed. 2005, 44, 1733–1735; 10.1002/anie.200462227 15712310

[7] For the pioneering work, see: A. Demonceau , A. F. Noels , A. J. Hubert , P. Theyssié , J. Chem. Soc. Chem. Commun. 1981, 688–689.

[8a] H. M. L. Davies , T. Hansen , J. Am. Chem. Soc. 1997, 119, 9075–9076;

[8b] H. M. L. Davies , T. Hansen , D. W. Hopper , S. A. Panaro , J. Am. Chem. Soc. 1999, 121, 6509–6510;

[8c] H. M. L. Davies , T. Hansen , M. R. Churchill , J. Am. Chem. Soc. 2000, 122, 3063–3070;

[8d] H. M. L. Davies , C. Venkataramani , T. Hansen , D. W. Hopper , J. Am. Chem. Soc. 2003, 125, 6462–6468. 12785786 10.1021/ja0290072

[9] E. Nakamura , N. Yoshikai , M. Yamanaka , J. Am. Chem. Soc. 2002, 124, 7181–7192. 12059244 10.1021/ja017823o

[10] J. Hansen , J. Autschbach , H. M. L. Davies , J. Org. Chem. 2009, 74, 6555–6563. 19637894 10.1021/jo9009968

[11] Earlier studies advocated an alignment of the C=Rh and the C−H bond, see:

[11a] D. F. Taber , K. K. You , A. L. Rheingold , J. Am. Chem. Soc. 1996, 118, 547–556;

[11b] M. P. Doyle , L. J. Westrum , W. N. E. Wolthuis , M. M. See , W. P. Boone , V. Bagheri , M. M. Pearson , J. Am. Chem. Soc. 1993, 115, 958–964; see also:;

[11c] D. F. Taber , P. V. Joshi , J. Org. Chem. 2004, 69, 4276–7278. 15176860 10.1021/jo0303766

[12] For a recent study from this laboratory in which the temperature-dependence of the kinetic isotope effect was used to characterize the transition state of a ruthenium catalyzed C−H insertion reaction as a “hydride transfer” process, see: S. Peil , A. Gutiérrez González , M. Leutzsch , A. Fürstner , J. Am. Chem. Soc. 2022, 144, 4158–4167. 35170941 10.1021/jacs.1c13446 PMC8915261

[13] For an accurate determination of the relative reactivity of different C−H bonds, see: A. Olmos , R. Gava , B. Noverges , D. Bellezza , K. Jacob , M. Besora , W. M. C. Sameera , M. Etienne , F. Maseras , G. Asensio , A. Caballero , P. J. Pérez , Angew. Chem. Int. Ed. 2018, 57, 13848–13852; 10.1002/anie.201807448 30015368

[14] K. Liao , S. Negretti , D. G. Musaev , J. Bacsa , H. M. L. Davies , Nature 2016, 533, 230–234. 27172046 10.1038/nature17651

[15] K. Liao , Y.-F. Yang , Y. Li , J. N. Sanders , K. N. Houk , D. G. Musaev , H. M. L. Davies , Nat. Chem. 2018, 10, 1048–1055. 30082883 10.1038/s41557-018-0087-7 PMC6650386

[16] C. Qin , H. M. L. Davies , J. Am. Chem. Soc. 2014, 136, 9792–9796. 24933043 10.1021/ja504797x

[17] K. Liao , T. C. Pickel , V. Boyarskikh , J. Bacsa , D. G. Musaev , H. M. L. Davies , Nature 2017, 551, 609–613. 29156454 10.1038/nature24641

[18] F. G. Adly , Catalysts 2017, 7, 347.

[19] R. Hrdina , Eur. J. Inorg. Chem. 2021, 501–528.

[20] For notable exceptions using catalysts based on metals other than Rh, see:

[20a] H.-Y. Thu , G. S.-M. Tong , J.-S. Huang , S. L.-F. Chan , Q.-H. Deng , C.-M. Che , Angew. Chem. Int. Ed. 2008, 47, 9747–9751; 10.1002/anie.200803157 18855950

[20b] H. Suematsu , T. Katsuki , J. Am. Chem. Soc. 2009, 131, 14218–14219. 19757773 10.1021/ja9065267

[21] Z. Ren , T. L. Sunderland , C. Tortoreto , T. Yang , J. F. Berry , D. G. Musaev , H. M. L. Davies , ACS Catal. 2018, 8, 10676–10682.

[22] In cyclopropanation, the heterobimetallic complex **3 c** afforded lower yields but higher asymmetric induction than the dirhodium complex **3 b**.

[23] S. Singha , M. Buchsteiner , G. Bistoni , R. Goddard , A. Fürstner , J. Am. Chem. Soc. 2021, 143, 5666–5673. 33829767 10.1021/jacs.1c01972 PMC8154533

[24] For a complementary catalyst design for asymmetric cyclopropanation developed in this laboratory, see:

[24a] F. P. Caló , A. Zimmer , G. Bistoni , A. Fürstner , J. Am. Chem. Soc. 2022, 144, 7465–7478; 35420801 10.1021/jacs.2c02258 PMC9052758

[24b] F. P. Caló , A. Fürstner , Angew. Chem. Int. Ed. 2020, 59, 13900–13907; 10.1002/anie.202004377 PMC7496581 32426901

[25a] S. Hashimoto , N. Watanabe , S. Ikegami , Tetrahedron Lett. 1990, 31, 5173–5174;

[25b] N. Watanabe , T. Ogawa , Y. Ohtake , S. Ikegami , S. Hashimoto , Synlett 1996, 1996, 85–86;

[25c] S. Kitagaki , M. Anada , O. Kataoka , K. Matsuno , C. Umeda , N. Watanabe , S. Hashimoto , J. Am. Chem. Soc. 1999, 121, 1417–1418;

[25d] S. Kitagaki , M. Kinoshita , M. Takeba , M. Anada , S. Hashimoto , Tetrahedron: Asymmetry 2000, 11, 3855;

[25e] M. Yamawaki , H. Tsutsui , S. Kitagaki , M. Anada , S. Hashimoto , Tetrahedron Lett. 2002, 43, 9561–9564;

[25f] H. Tsutsui , Y. Yamaguchi , S. Kitagaki , S. Nakamura , M. Anada , S. Hashimoto , Tetrahedron: Asymmetry 2003, 14, 817;

[25g] T. Goto , K. Takeda , M. Anada , K. Ando , S. Hashimoto , Tetrahedron Lett. 2011, 52, 4200;

[25h] T. Goto , K. Takeda , N. Shimada , H. Nambu , M. Anada , M. Shiro , K. Ando , S. Hashimoto , Angew. Chem. Int. Ed. 2011, 50, 6803; 10.1002/anie.201101905 21674740

[25i] M. Ito , Y. Kondo , H. Nambu , M. Anada , K. Takeda , S. Hashimoto , Tetrahedron Lett. 2015, 56, 1397–1400.

[26] Incorporation of the Bi(+2) center also impacts on the electronics of the resulting carbene complexes, see:

[26a] L. R. Collins , M. van Gastel , F. Neese , A. Fürstner , J. Am. Chem. Soc. 2018, 140, 13042; 30217113 10.1021/jacs.8b08384

[26b] F. P. Caló , G. Bistoni , A. A. Auer , M. Leutzsch , A. Fürstner , J. Am. Chem. Soc. 2021, 143, 12473–12479. 34351134 10.1021/jacs.1c06414 PMC8377716

[27] For improved procedures for the preparation of such heterobimetallic catalysts, see: L. E. Löffler , M. Buchsteiner , L. R. Collins , F. P. Caló , S. Singha , A. Fürstner , Helv. Chim. Acta 2021, 104, e2100042.

[28] For pioneering studies into achiral [BiRh] paddlewheel complexes, see:

[28a] E. V. Dikarev , T. G. Gray , B. Li , Angew. Chem. Int. Ed. 2005, 44, 1721–1724; 10.1002/anie.200462433 15693042

[28b] E. V. Dikarev , B. Li , H. Zhang , J. Am. Chem. Soc. 2006, 128, 2814–2815; 16506756 10.1021/ja058294h

[28c] J. Hansen , B. Li , E. Dikarev , J. Autschbach , H. M. L. Davies , J. Org. Chem. 2009, 74, 6564–6571; 19637895 10.1021/jo900998s

[28d] A. S. Filatov , M. Napier , V. D. Vreshch , N. J. Sumner , E. V. Dikarev , M. A. Petrukhina , Inorg. Chem. 2012, 51, 566–571; 22133106 10.1021/ic202089p

[28e] T. L. Sunderland , J. F. Berry , Dalton Trans. 2016, 45, 50–55. 26599620 10.1039/c5dt03740a PMC6779122

[29] C. Werlé , R. Goddard , P. Philipps , C. Farès , A. Fürstner , Angew. Chem. Int. Ed. 2016, 55, 10760–10765; 10.1002/anie.201605502 27484943

[30a] C. Werlé , R. Goddard , A. Fürstner , Angew. Chem. Int. Ed. 2015, 54, 15452–15456; 10.1002/anie.201506902 PMC4832834 26534892

[30b] C. Werlé , R. Goddard , P. Philipps , C. Farès , A. Fürstner , J. Am. Chem. Soc. 2016, 138, 3797–3805. 26910883 10.1021/jacs.5b13321

[31] L. R. Collins , S. Auris , R. Goddard , A. Fürstner , Angew. Chem. Int. Ed. 2019, 58, 3557; 10.1002/anie.201900265 30672077

[32a] J. P. Wagner , P. R. Schreiner , Angew. Chem. Int. Ed. 2015, 54, 12274; 10.1002/anie.201503476 26262562

[32b] E. Solel , M. Ruth , P. R. Schreiner , J. Am. Chem. Soc. 2021, 143, 20837–20848. 34846890 10.1021/jacs.1c09222

[33a] D. J. Liptrot , P. P. Power , Nat. Rev. Chem. 2017, 1, 0004;

[33b] K. L. Mears , P. P. Power , Acc. Chem. Res. 2022, 55, 1337–1348. 35427132 10.1021/acs.accounts.2c00116

[34] Importantly, such intramolecular LD does not get canceled in solution, see: J. M. Schümann , J. P. Wagner , A. K. Eckhardt , H. Quanz , P. R. Schreiner , J. Am. Chem. Soc. 2021, 143, 41–45. 33320651 10.1021/jacs.0c09597

[35] F. G. Adly , M. G. Gardiner , A. Ghanem , Chem. Eur. J. 2016, 22, 3447–3461. 26833989 10.1002/chem.201504817

[36] In the absence of any other reagent, insertion of the rhodium carbene derived from diazoester **8 c** and catalyst **7 d** into pentane used as solvent gave a mixture of isomers in 85 % NMR yield; the regioselectivity for insertion at C2:C1 ≈ 64 : 36, see the Supporting Information.

[37] M. Buchsteiner , L. Martinez-Rodriguez , P. Jerabek , I. Pozo , M. Patzer , N. Nöthling , C. Lehmann , A. Fürstner , Chem. Eur. J. 2020, 26, 2509. 31916634 10.1002/chem.202000081 PMC7065061

[38] The absolute configuration of product **23 b** was determined by X-ray diffraction analysis; the other compounds shown in Table 1 were assigned by analogy. In case of compounds **12** and **20 e**, this tentative assignment could be confirmed by comparison with literature data; for details, see the Supporting Information.

[39] For comparison: the classical catalyst [Rh_2_((*R)-*BPCP)_4_] furnished the insertion product into *tert*-butyl methyl ether in 74 % yield with 82 % ee, see: D. M. Guptill , H. M. L. Davies , J. Am. Chem. Soc. 2014, 136, 17718–17721.25474724

[40] J. Vaitla , Y. T. Boni , H. M. L. Davies , Angew. Chem. Int. Ed. 2020, 59, 7397–7402; 10.1002/anie.201916530 PMC7233467 31908146

[41] H. M. L. Davies , E. G. Antoulinakis , T. Hansen , Org. Lett. 1999, 1, 383–386.

[42] For related findings, see ref 16 and the following: N. A. Falcone , A. T. Bosse , H. Park , J.-Q. Yu , H. M. L. Davies , E. J. Sorensen , Org. Lett. 2021, 23, 9393–9397. 34865494 10.1021/acs.orglett.1c03502

[43] L. Fu , D. M. Guptill , H. M. L. Davies , J. Am. Chem. Soc. 2016, 138, 5761–5764. 27064173 10.1021/jacs.6b01941

[44] This result must be assessed against the fact that polar substituents exert long-range inductive effects even within fully saturated alkyl chains that strongly impact on the site selectivity of C−H insertion reactions, see: K. Liao , W. Liu , Z. L. Niemeyer , Z. Ren , J. Bacsa , D. G. Musaev , M. S. Sigman , H. M. L. Davies , ACS Catal. 2018, 8, 678–682.

[45] However, the analogous *Z*-configured alkenes largely failed to react as they might not fit well into the narrow pore of the chiral catalyst.

[46] More electron rich donor/acceptor diazo derivatives such as compound **8 b** gave only low yields of the corresponding propargylation products.

[47] Control over the remote stereocenter is subject to further study in this laboratory.

[48] The reaction with gaseous propane afforded an inseparable ≈1 : 3 mixture of isomers formed by insertion into C1 and C2.

[49] For the reaction of methane with ethyl diazoacetate catalyzed by an achiral silver complex in supercritical CO_2_, see: A. Caballero , E. Despagnet-Ayoub , M. Mar Díaz-Requejo , A. Díaz-Rodríguez , M. E. González-Núñez , R. Mello , B. K. Muñoz , W.-S. Ojo , G. Asensio , M. Etienne , P. J. Pérez , Science 2011, 332, 835–838. 21566191 10.1126/science.1204131

