# Supplementary material for: Chiral Bismuth‐Rhodium Paddlewheel Complexes Empowered by London Dispersion: The C−H Functionalization Nexus
Source: Angew Chem Int Ed Engl. 2022 Oct 11;61(45):e202212546. doi: 10.1002/anie.202212546 (PMC9828831; doi:10.1002/anie.202212546)
Supplement: Supplementary file 1 — Supporting Information [file ANIE-61-0-s001.pdf]

Angewandte Chemie
Eine Zeitschrift der Gesellschaft Deutscher Chemiker

Supporting Information

Chiral Bismuth-Rhodium Paddlewheel Complexes Empowered by London Dispersion: The C–H Functionalization Nexus

*M. Buchsteiner, S. Singha, J. Decaens, A. Fürstner**

Supporting Crystallographic Data

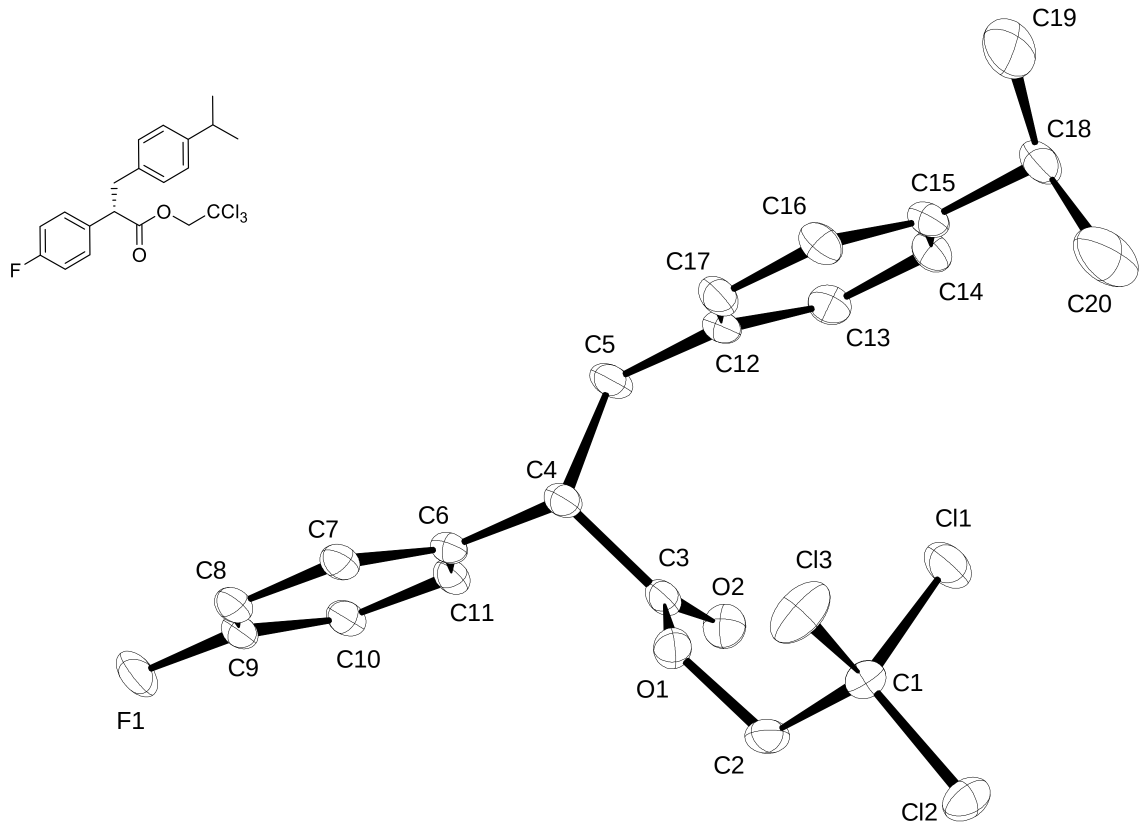

Figure S1. Structure of 2,2,2-trichloroethyl (*S*)-2-(4-fluorophenyl)3-(4-isopropylphenyl)propanoate (**23b**) in the solid state; all H-atoms removed for clarity

X-ray Crystal Structure Analysis of Compound 23b: $C_{20} H_{20} Cl_3 F O_2$, M_r = 417.73 g mol^{-1}, colorless prism, crystal size 0.31 x 0.14 x 0.11 mm^3, monoclinic, space group $P2_1$[4], a = 10.3857(19) Å, b = 5.6716(12) Å, c = 17.111(3) Å, β = 98.769(11)°, V = 996.1(3) Å^3, T = 100(2) K, Z = 2, D_{calc} = 1.393 g·cm^3, λ = 0.71073 Å, μ(*Mo-K*$_\alpha$) = 0.480 mm^{-1}, Gaussian absorption correction ($T_{\min}$ = 0.90, $T_{\max}$ = 0.95), Bruker-AXS Kappa Mach3 with APEX-II detector and IµS microfocus source, 2.878 < θ < 33.078°, 17357 measured reflections, 7426 independent reflections, 6619 reflections with $I > 2\sigma(I)$, R_{int} = 0.0673. The structure was solved by *SHELXT* and refined by full-matrix least-squares (*SHELXL*) against F^2 to R_1 = 0.071 [$I > 2\sigma(I)$], wR_2 = 0.189, S = 1.067, 237 parameters, absolute structure parameter = 0.04(7).

Largest diff. peak and hole = 1.1 (0.81 Å from Cl2) and -1.0 (0.74 Å from Cl1) e · Å^{-3}.

Complete .cif-data of the compound are available under **CCDC- 2191047**

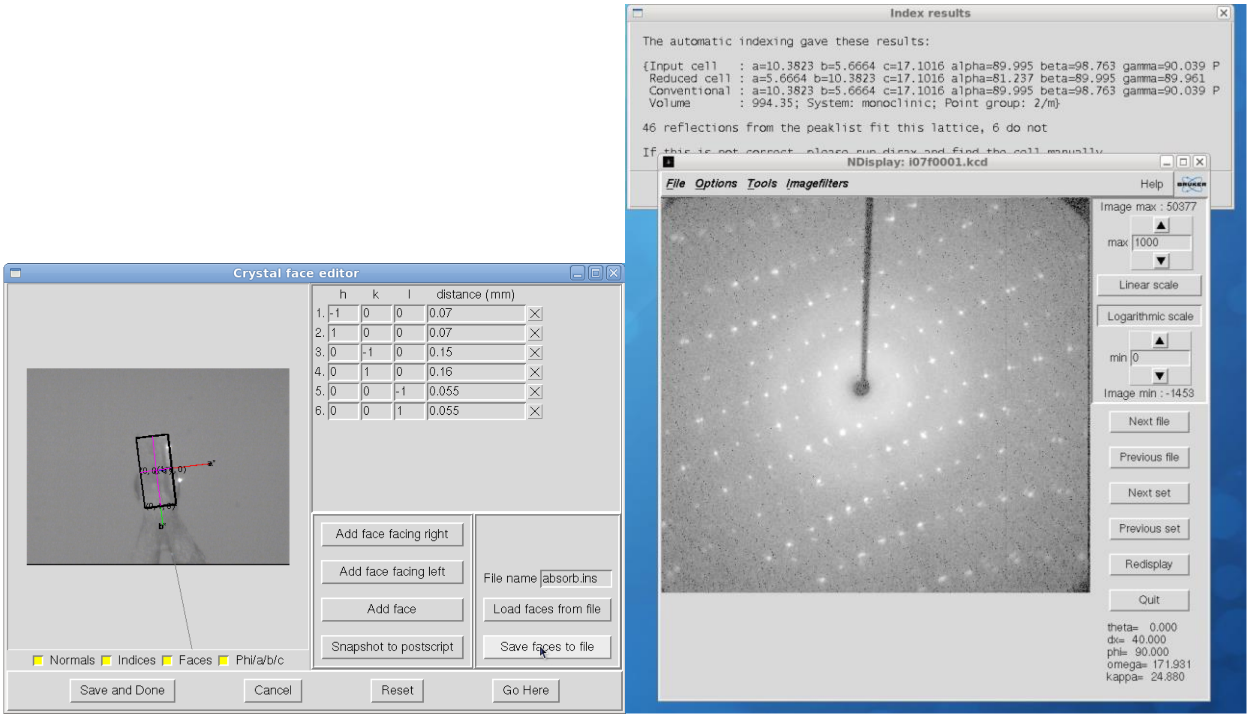

INTENSITY STATISTICS FOR DATASET # 1 14225sadabs.raw

Resolution	#Data	#Theory	%Complete	Redundancy	Mean I	Mean I/s	Rmerge	Rsigma
Inf - 2.61	112	120	93.3	3.61	170.32	21.36	0.0665	0.0482
2.61 - 1.76	266	266	100.0	3.13	75.75	18.99	0.0723	0.0523
1.76 - 1.40	366	367	99.7	3.13	40.62	18.21	0.0640	0.0518
1.40 - 1.23	391	392	99.7	2.90	31.99	17.46	0.0625	0.0549
1.23 - 1.11	384	385	99.7	2.98	26.98	17.28	0.0605	0.0554
1.11 - 1.03	397	397	100.0	2.77	17.09	15.65	0.0618	0.0584
1.03 - 0.97	354	354	100.0	2.69	12.26	14.17	0.0590	0.0606
0.97 - 0.92	405	405	100.0	2.63	10.94	14.09	0.0631	0.0637
0.92 - 0.88	401	401	100.0	2.44	11.47	12.84	0.0638	0.0679
0.88 - 0.85	309	309	100.0	2.30	9.48	11.98	0.0620	0.0728
0.85 - 0.82	372	373	99.7	2.25	7.52	11.43	0.0635	0.0777
0.82 - 0.79	454	458	99.1	2.18	6.47	10.20	0.0622	0.0826
0.79 - 0.77	361	363	99.4	2.01	5.24	9.09	0.0762	0.0964
0.77 - 0.75	371	372	99.7	1.99	5.03	8.53	0.0790	0.1003
0.75 - 0.73	412	416	99.0	1.92	4.82	7.90	0.0880	0.1079
0.73 - 0.71	430	439	97.9	1.88	4.35	7.36	0.0898	0.1189
0.71 - 0.70	245	251	97.6	1.78	3.70	6.34	0.0983	0.1412
0.70 - 0.68	580	594	97.6	1.76	3.17	5.40	0.1130	0.1708
0.68 - 0.67	258	281	91.8	1.62	3.26	4.98	0.1190	0.1862
0.67 - 0.66	305	334	91.3	1.60	2.61	4.32	0.1335	0.2259
0.66 - 0.65	259	297	87.2	1.51	2.70	4.12	0.1284	0.2347
0.75 - 0.65	2489	2612	95.3	1.74	3.59	5.93	0.1036	0.1543
Inf - 0.65	7432	7574	98.1	2.29	15.94	11.23	0.0672	0.0648

Table 2. Bond lengths [Å] and angles [°].

Cl(1)-C(1)	1.768(4)	Cl(2)-C(1)	1.770(4)
Cl(3)-C(1)	1.763(4)	F(1)-C(9)	1.357(4)
O(1)-C(2)	1.424(4)	O(1)-C(3)	1.351(4)
O(2)-C(3)	1.198(5)	C(1)-C(2)	1.519(5)
C(3)-C(4)	1.516(5)	C(4)-C(5)	1.539(4)
C(4)-C(6)	1.524(4)	C(5)-C(12)	1.503(4)
C(6)-C(7)	1.392(5)	C(6)-C(11)	1.391(5)
C(7)-C(8)	1.396(4)	C(8)-C(9)	1.374(6)
C(9)-C(10)	1.384(5)	C(10)-C(11)	1.394(4)
C(12)-C(13)	1.387(5)	C(12)-C(17)	1.399(5)
C(13)-C(14)	1.395(5)	C(14)-C(15)	1.388(5)
C(15)-C(16)	1.396(5)	C(15)-C(18)	1.521(5)
C(16)-C(17)	1.392(4)	C(18)-C(19)	1.503(7)
C(18)-C(20)	1.515(6)		
C(3)-O(1)-C(2)	118.4(3)	Cl(1)-C(1)-Cl(2)	110.2(2)
Cl(3)-C(1)-Cl(1)	108.92(19)	Cl(3)-C(1)-Cl(2)	108.62(18)
C(2)-C(1)-Cl(1)	110.2(2)	C(2)-C(1)-Cl(2)	107.7(2)
C(2)-C(1)-Cl(3)	111.2(3)	O(1)-C(2)-C(1)	109.4(3)
O(1)-C(3)-C(4)	109.3(3)	O(2)-C(3)-O(1)	124.8(3)
O(2)-C(3)-C(4)	125.9(3)	C(3)-C(4)-C(5)	109.9(3)
C(3)-C(4)-C(6)	110.1(3)	C(6)-C(4)-C(5)	111.4(3)
C(12)-C(5)-C(4)	114.3(3)	C(7)-C(6)-C(4)	118.9(3)
C(11)-C(6)-C(4)	121.5(3)	C(11)-C(6)-C(7)	119.6(3)
C(6)-C(7)-C(8)	120.6(3)	C(9)-C(8)-C(7)	118.2(3)
F(1)-C(9)-C(8)	118.7(3)	F(1)-C(9)-C(10)	118.1(3)
C(8)-C(9)-C(10)	123.2(3)	C(9)-C(10)-C(11)	117.8(3)
C(6)-C(11)-C(10)	120.7(3)	C(13)-C(12)-C(5)	120.8(3)
C(13)-C(12)-C(17)	118.3(3)	C(17)-C(12)-C(5)	120.9(3)
C(12)-C(13)-C(14)	121.1(3)	C(15)-C(14)-C(13)	120.9(3)
C(14)-C(15)-C(16)	118.1(3)	C(14)-C(15)-C(18)	120.6(3)
C(16)-C(15)-C(18)	121.3(3)	C(17)-C(16)-C(15)	121.2(4)
C(16)-C(17)-C(12)	120.4(3)	C(19)-C(18)-C(15)	112.7(4)
C(19)-C(18)-C(20)	111.3(6)	C(20)-C(18)-C(15)	110.5(3)

General. Unless stated otherwise, all reactions were carried out under argon atmosphere in flame dried Schlenk glassware. The solvents were purified by distillation over the indicated drying agents under argon: THF (Mg/anthracene), Et_2O (Mg/anthacene), pentane (Na/K), CH_2Cl_2 (CaH_2). MeCN and Et_3N were dried by an absorption solvent purification system based on molecular sieves. Flash chromatography: VWR Chemicals silica gel 40 – 63 µm. TLCs were stained with vanillin/H_2SO_4, anisaldehyde or PMA.

C_6F_6 was purchased from ABCR and used as received

NMR spectra were recorded on Bruker DPX 300, AV 400, AV 500 or AV III 600 spectrometers in the solvents indicated; chemical shifts are given in ppm relative to TMS, coupling constants (*J*) in Hz. The solvent signals were used as references and the chemical shifts converted to the TMS scale ($CDCl_3$: δ_C = 77.2 ppm; residual $CHCl_3$: δ_H = 7.26 ppm; CD_2Cl_2: δ_C = 54.0 ppm; residual $CHDCl_2$: δ_H = 5.32 ppm; $(CD_3)_2SO$: δ_C = 39.5 ppm; residual $(CD_3)(CD_2H)SO$: δ_H = 2.50 ppm; C_6D_6: δ_C = 128.1 ppm; residual C_6D_5H: δ_H = 7.16 ppm). Proton and carbon assignments were established using HSQC, HMBC and NOESY experiments.

IR: Alpha Platinum ATR (Bruker), wavenumbers ($\tilde{\nu}$) in cm^{-1}.

MS (EI): Finnigan MAT 8200 (70 eV), ESI-MS: ESQ 3000 (Bruker) or Thermo Scientific LTQ-FT or Thermo Scientific Exactive. HRMS: Bruker APEX III FT-MS (7 T magnet) or MAT 95 (Finnigan) or Thermo Scientific LTQ-FT or Thermo Scientific Exactive. GC-MS was measured on a Shimadzu GCMS-QP2010 Ultra instrument.

HPLC analyses for the determination of enantiomeric excesses were conducted on a Shimadzu LC 2020 instrument equipped with a Shimadzu SPD-M20A UV/VIS detector. Solvents were purchased in HPLC grade and used without further purification. The exact conditions are specified for each substrate.

Optical rotations were measured with an A-Krüss Otronic Model P8000-t polarimeter at a wavelength of 589 nm. The values are given as specific optical rotation with exact temperature, concentration (c/(10 mg/mL)) and solvent.

Unless stated otherwise, all commercially available compounds (abcr, Acros, TCI, Aldrich, Alfa Aesar, Fluoro Chem) were used as received.

$[BiRh(OC(O)CF_3)_4]$ was prepared according to the literature.[1]

The diazo derivatives were prepared according to literature procedures; the recorded characterization data matched the literature.[2,3,6]

Preparation of the New Heterobimetallic Paddlewheel Complexes

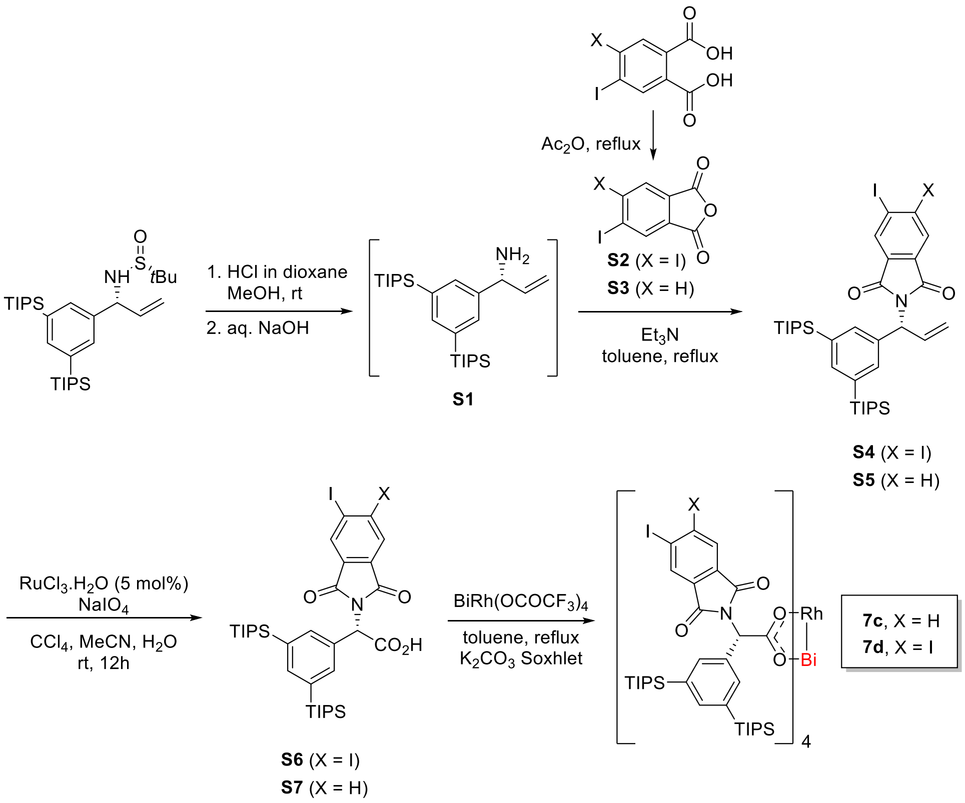

Scheme S1. Preparation of the new [BiRh] tetracarboxylate complexes **7c**,**d** comprising iodinated phthalimido "paddles"

5,6-Diiodoisobenzofuran-1,3-dione (S2). Acetic anhydride (15 mL) was added to a round-bottom flask

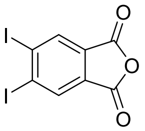

charged with 4,5-diiodophthalic acid (2.45 g, 5.86 mmol)[4] and the mixture was stirred at 145°C (bath temperature) for 2 h. Excess acetic anhydride was removed under reduced pressure and the residue was dried under high vacuum to give the desired product as a pale yellow solid (2.12 mg, 90%). ^{1}H NMR (400 MHz, DMSO-d$_6$): δ = 8.53 (s, 2H); ^{13}C NMR (101 MHz, DMSO-d$_6$): δ = 161.8, 134.6, 131.5, 119.7; IR (ATR): ṽ = 1843, 1777, 1730, 1698, 1537, 1350, 1289, 1235, 1080, 899, 870, 853, 727, 693, 583 cm^{-1}; HRMS (EI$^+$) for $C_8H_2O_3I_2$ [M]$^+$: calcd: 399.80879, found: 399.80889.

5-Iodoisobenzofuran-1,3-dione (S3). Prepared according to the literature procedure.[5] Characterization

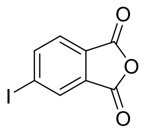

data matched with the reported data. ^{1}H NMR (400 MHz, CDCl$_3$): δ = 8.38 (dd, J = 1.4, 0.6 Hz, 1H), 8.26 (dd, J = 8.0, 1.4 Hz, 1H), 7.73 (dd, J = 8.0, 0.6 Hz, 1H); ^{13}C NMR (101 MHz, CDCl$_3$): δ = 162.2, 161.3, 145.2, 134.8, 132.6, 130.4, 126.6, 103.6; IR (ATR): ṽ = 3098, 1842, 1766, 1590, 1411, 1318, 1241, 1168, 1101, 885, 854, 838, 726, 684, 658, 632, 577, 540, 480, 406 cm^{-1}; HRMS (ESI$^+$) for $C_8H_3O_3I$ [M+H]$^+$: calcd: 274.91997, found: 274.91980.

(*R*)-2-(1-(3,5-Bis(triisopropylsilyl)phenyl)allyl)-5,6-diiodoisoindoline-1,3-dione (S4). HCl (4 M in dioxane, 0.33 mL, 1.347 mmol) was added at 0°C under air to a solution of (*R*)-N-((*R*)-1-(3,5-bis(triisopropylsilyl)phenyl)allyl)-2-methylpropane-2-sulfinamide (**S1**) (247 mg, 0.449 mmol)[6] in methanol (HPLC-grade, 6 mL). The flask was capped with a rubber septum and the solution was stirred at room temperature for 1 h. The mixture was concentrated under vacuum. Water (20 mL) and CH_2Cl_2 (20 mL) were added to the residue and the aqueous phase was basified to pH ≈ 10 upon addition of aqueous NaOH (3 M) before it was extracted with CH_2Cl_2 (3 x 20 mL). The combined organic layers were dried over Na_2SO_4 and the solvent was removed in vacuum to give (*R*)-1-(3,5-bis(triisopropylsilyl)phenyl)prop-2-en-1-amine, which was used directly in the next step.

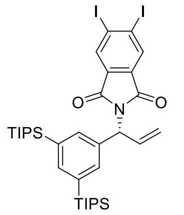

5,6-Diiodoisobenzofuran-1,3-dione (**S2**) (197.3 mg, 0.493 mmol) and Et_3N (63 µL, 0.449 mmol) were added to the crude amine in toluene (20 mL) and the resulting mixture was stirred at reflux temperature for 36 h while the released water was collected in a Dean-Stark apparatus. Evaporation of the solvent and purification of the residue by flash chromatography (SiO_2) using 4% Et_2O in pentane as eluent afforded the title compound as a colorless waxy solid (315 mg, 85% yield over 2 steps). $[\alpha]_D^{20}$ = 7.2 (c = 0.5, $CHCl_3$); ^1H NMR (400 MHz, $CDCl_3$): δ = 8.28 (s, 2H), 7.53 (d, *J* = 2.7 Hz, 3H), 6.61 (ddd, *J* = 17.3, 10.3, 7.2 Hz, 1H), 5.90 (dd, *J* = 7.2, 1.48 Hz, 1H), 5.39 – 5.24 (m, 2H), 1.37 (hept, *J* = 7.4 Hz, 6H), 1.03 (dd, *J* = 7.5, 2.1 Hz, 36H); ^{13}C NMR (101 MHz, $CDCl_3$): δ = 166.0, 142.1, 136.0, 135.2, 134.4, 133.9, 133.8, 132.2, 119.0, 115.2, 58.0, 18.7, 10.9; IR (ATR): ṽ = 2941, 2862, 1772, 1712, 1461, 1366, 1337, 1130, 1015, 993, 879, 715, 641, 563, 503, cm^{-1}; HRMS (ESI^+) for $C_{35}H_{51}NO_2Si_2I_2Na$ $[M+Na]^+$: calcd: 850.14400, found: 850.14313.

(*R*)-2-(1-(3,5-Bis(triisopropylsilyl)phenyl)allyl)-5-iodoisoindoline-1,3-dione (S5). Prepared analogously from compound **S1** (542 mg, 1.21 mmol) and anhydride **S3** (430 mg, 1.57 mmol) as a colorless sticky solid (605 mg, 71%). ^1H NMR (400 MHz, $CDCl_3$): δ = 8.17 (d, *J* = 1.4 Hz, 1H), 8.05 (dd, *J* = 7.8, 1.5 Hz, 1H), 7.58 – 7.50 (m, 4H), 6.62 (ddd, *J* = 17.3, 10.2, 7.2 Hz, 1H), 5.93 (d, *J* = 7.2 Hz, 1H), 5.44 – 5.22 (m, 2H), 1.36 (hept, *J* = 7.5 Hz, 6H), 1.03 (dd, *J* = 7.5, 2.3 Hz, 36H); ^{13}C NMR (101 MHz, $CDCl_3$) δ 167.3, 166.4, 143.0, 142.0, 136.1, 135.1, 134.6, 133.8, 133.5, 132.5, 131.3, 124.7, 118.9, 100.9, 57.8, 18.6 (2 x), 10.9; IR (ATR): ṽ = 2941, 2889, 2863, 1772, 1715, 1602, 1461, 1412, 1367, 1343, 1312, 1239, 1170, 1134, 1015, 993, 881, 840, 789, 744, 712, 675, 641, 562, 502 cm^{-1}; HRMS (ESI^+) for $C_{35}H_{52}NO_2Si_2I$ $[M+Na]^+$: calcd: 724.24735, found: 724.24688.

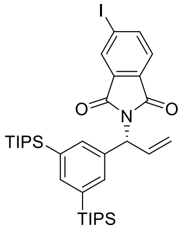

(*S*)-2-(3,5-Bis(triisopropylsilyl)phenyl)-2-(5,6-diiodo-1,3-dioxoisoindolin-2-yl)acetic acid (S6). A round

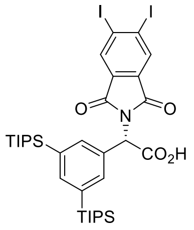

bottom flask containing a magnetic stir-bar was charged with (*R*)-2-(1-(3,5-bis(triisopropylsilyl)phenyl)allyl)-5,6-diiodoisoindoline-1,3-dione (**S4**) (290 mg, 0.35 mmol), sodium metaperiodate (375 mg, 1.752 mmol), water (3 mL), acetonitrile (2 mL) and CCl_4 (2 mL). Ruthenium trichloride hydrate (3.6 mg, 0.017 mmol, 5 mol%) was added to the biphasic mixture, which was stirred vigorously for 12 h at ambient temperature. The mixture was diluted with CH_2Cl_2 (10 mL) and the phases were separated. The aqueous layer was extracted with CH_2Cl_2 (3 x 20 mL), the combined extracts were dried over Na_2SO_4, filtered through a Celite® pad, and the filtrate was concentrated. The crude product was purified by flash chromatography (SiO_2) using 10% EtOAc in pentane + 1% AcOH as eluent to afford the title compound as a colorless solid (215 mg, 73%). $[\alpha]_D^{20}$ = 3.2 (c = 2.7, $CHCl_3$); ^{1}H NMR (400 MHz, $CDCl_3$): δ = 8.32 (s, 2H), 7.62 (d, *J* = 1.0 Hz, 2H), 7.58 (d, *J* = 1.3 Hz, 1H), 6.02 (s, 1H), 1.38 (h, *J* = 7.4 Hz, 6H), 1.04 (d, *J* = 7.5 Hz, 36H); ^{13}C NMR (101 MHz, $CDCl_3$): δ = 173.1, 165.3, 142.9, 137.0, 134.1, 134.1, 132.0, 131.6, 115.6, 56.6, 18.6, 18.6, 10.8; IR (ATR): ṽ = 2941, 2863, 1778, 1714, 1461, 1366, 1230, 1129, 1106, 1015, 881, 746, 673, 642, 582, 502 cm^{-1}; HRMS (ESI^+) for $C_{34}H_{49}NO_4Si_2I_2Na$ $[M+Na]^+$: calcd: 868.11818, found: 868.11798

(*S*)-2-(3,5-Bis(triisopropylsilyl)phenyl)-2-(5-iodo-1,3-dioxoisoindolin-2-yl)acetic acid (S7). Prepared

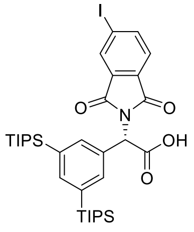

analogously from compound **S5** (600 mg, 0.855 mmol) as a colorless solid (440 mg, 71%). ^{1}H NMR (400 MHz, $CDCl_3$): δ = 8.19 (d, *J* = 1.5 Hz, 1H), 8.07 (dd, *J* = 7.9, 1.5 Hz, 1H), 7.64 (s, 2H), 7.61 – 7.54 (m, 2H), 6.04 (s, 1H), 1.39 (hept, *J* = 7.6 Hz, 6H), 1.04 (d, *J* = 7.4 Hz, 36H); ^{13}C NMR (101 MHz, $CDCl_3$): δ = 173.2, 166.4, 165.5, 143.1, 142.6, 136.8, 133.8, 133.1, 132.7, 131.7, 130.9, 124.9, 101.1, 56.4, 18.5, 18.5, 10.7; IR (ATR): ṽ = 2942, 2863, 1777, 1720, 1603, 1461, 1413, 1369, 1107, 1015, 916, 881, 789, 743, 729, 675, 641, 561, 500, 464 cm^{-1}; HRMS (ESI^+) for $C_{34}H_{51}NO_4ISi_2$ $[M+H]^+$: calcd: 720.23959, found: 720.23993.

Complex 7d. A mixture of $[BiRh(OCOCF_3)_4]$ (36 mg, 0.047 mmol)[1] and acid **S6** (200 mg, 0.236 mmol) in

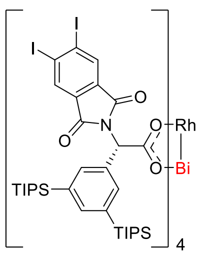

toluene (25 mL) was stirred at reflux temperature for 3 h, passing the condensed vapor through a Soxhlet apparatus filled with K_2CO_3; at this point, ligand exchange was complete as judged by ^{19}F NMR. The mixture was concentrated in vacuum and the residue was purified by flash chromatography using 90% $CHCl_3$ in pentane as eluent to give the title complex as a yellow solid (163 mg, 93%). NMR spectra were recorded at 80°C; at lower temperature only very broad signals with poor resolution were observed. $[\alpha]_D^{20}$ = 111.9 (c = 1.4, $CHCl_3$); ^{1}H NMR (600 MHz, $CDCl_3$, 353K) : δ = 8.41 (s, 8H), 7.62 (s, 8H), 7.57 (s, 4H), 6.31 (s, 4H), 1.35

(hept, J = 7. 5 Hz, 24H), 1.02 (dd, J = 7.5, 5.3 Hz, 144H); ^{13}C NMR (151 MHz, $CDCl_3$, 353K): δ = 181.7, 165.1, 142.4, 137.8, 134.4, 133.6, 133.3, 132.7, 114.9, 58.4, 18.8, 18.8, 11.1; IR (ATR): ṽ = 2941, 2863, 1777, 1717, 1593, 1463, 1364, 1130, 993, 880, 751, 643, 581 cm^{-1}; HRMS (ESI$^+$) for this complex could not be measured due to poor ionization.

Complex 7c. Prepared analogously from [BiRh(OTfa)$_4$] (32 mg, 0.042 mmol) and acid **S7** (174 mg, 0.242 mmol) as a yellow solid (118 mg, 88%). NMR spectra were recorded at 80 °C; at lower temperature only very broad signals with poor resolution were observed. ^{1}H NMR (600 MHz, $CDCl_3$, 353K): δ = 8.26 (d, J = 1.5 Hz, 4H), 8.00 (dd, J = 7.8, 1.5 Hz, 4H), 7.65 (s, 8H), 7.60 (d, J = 7.8 Hz, 4H), 7.56 (s, 4H), 6.33 (s, 4H), 1.34 (h, J = 7.5 Hz, 24H), 1.09 (d, J = 7.5 Hz, 12H), 1.02 (dd, J = 7.5, 4.8 Hz, 132H); ^{13}C NMR (151 MHz, $CDCl_3$, 353K): δ = 181.9, 166.2, 165.4, 142.9, 142.3, 137.8, 134.0, 133.8, 133.6, 133.0, 131.8, 125.0, 100.6, 58.3, 18.8 (2 x), 11.2; IR (ATR): ṽ = 2940, 2889, 2863, 1775, 1717, 1598, 1461, 1411, 1362, 1326, 1265, 1101, 1013, 880, 781, 747, 713, 675, 663, 642, 563, 500, 421 cm^{-1}; HRMS (ESI$^+$) for this complex could not be measured due to poor ionization.

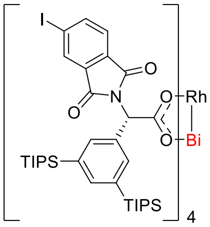

Cyclopropanation

2,2,2-Trichloroethyl (1*S*,2*R*)-1-(3-methoxyphenyl)-2-phenylcyclopropane-1-carboxylate (9a). An oven dried jacketed Schlenk flask equipped with a magnetic stir bar was charged with the [BiRh] catalyst (0.001 mmol, 1 mol%) under argon. Styrene (52.1 mg, 0.5 mmol) and pentane (1 mL) were added and the resulting solution cooled to −10 °C. A solution of the diazo compound **8a** (32.2 mg, 0.1 mmol) in pentane (3 mL) was added dropwise over 10 min. The resulting mixture was stirred at −10 °C until TLC analysis indicated the complete consumption of the diazo compound. For work up, the mixture was absorbed on silica, which was loaded on top of a silica column. Purification by flash chromatography (hexanes/EtOAc) afforded the title compound as a colorless oil; with [BiRh(*S*-PTTL)$_4$]·MeCN (**6a**): 92%, 59% ee; with catalyst **7b**: 98%, 87% ee; with **7d**: 77%, 97% ee. [The ee was determined by HPLC analysis: Daicel 150 mm Chiralpak OJ-3, Ø 4.6 mm, *n*-heptane/*iso*-propanol = 90/10, v = 1.0 mL/min, λ = 210 nm, t(major) = 6.85 min, t(minor) = 4.81 min.] $[\alpha]_D^{20}$ = +17.8 (c = 1.2, $CHCl_3$); ^{1}H NMR (500 MHz, $CDCl_3$): δ = 7.09 (dd, J = 5.0, 1.9 Hz, 3H), 7.05 (t, J = 7.9 Hz, 1H), 6.85 – 6.80 (m, 2H), 6.68 (dddd, J = 7.0, 3.6, 2.1, 1.0 Hz, 2H), 6.56 (dd, J = 2.6, 1.6 Hz, 1H), 4.86 (d, J = 11.9 Hz, 1H), 4.64 (d, J = 11.9 Hz, 1H), 3.59 (s, 3H), 3.20 (dd, J = 9.4, 7.4 Hz, 1H), 2.26 (dd, J = 9.4, 5.1 Hz, 1H), 2.00 (dd, J = 7.5, 5.1 Hz, 1H); ^{13}C NMR (101 MHz, $CDCl_3$): δ = 172.2, 159.0, 135.9, 135.3, 128.7, 128.2, 128.0, 126.8,

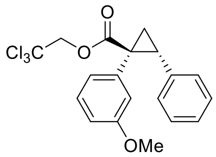

124.6, 117.6, 113.6, 95.2, 74.5, 55.2, 37.3, 34.0, 20.5; IR (ATR): ṽ = 2957, 1732, 1584, 1433, 1238, 1147, 1043, 804, 694, 572 cm^{-1}; HRMS (ESI^+) for $C_{19}H_{17}O_3Cl_3Na$ $[M+Na^+]^+$: calcd: 421.01355, found: 421.01384.

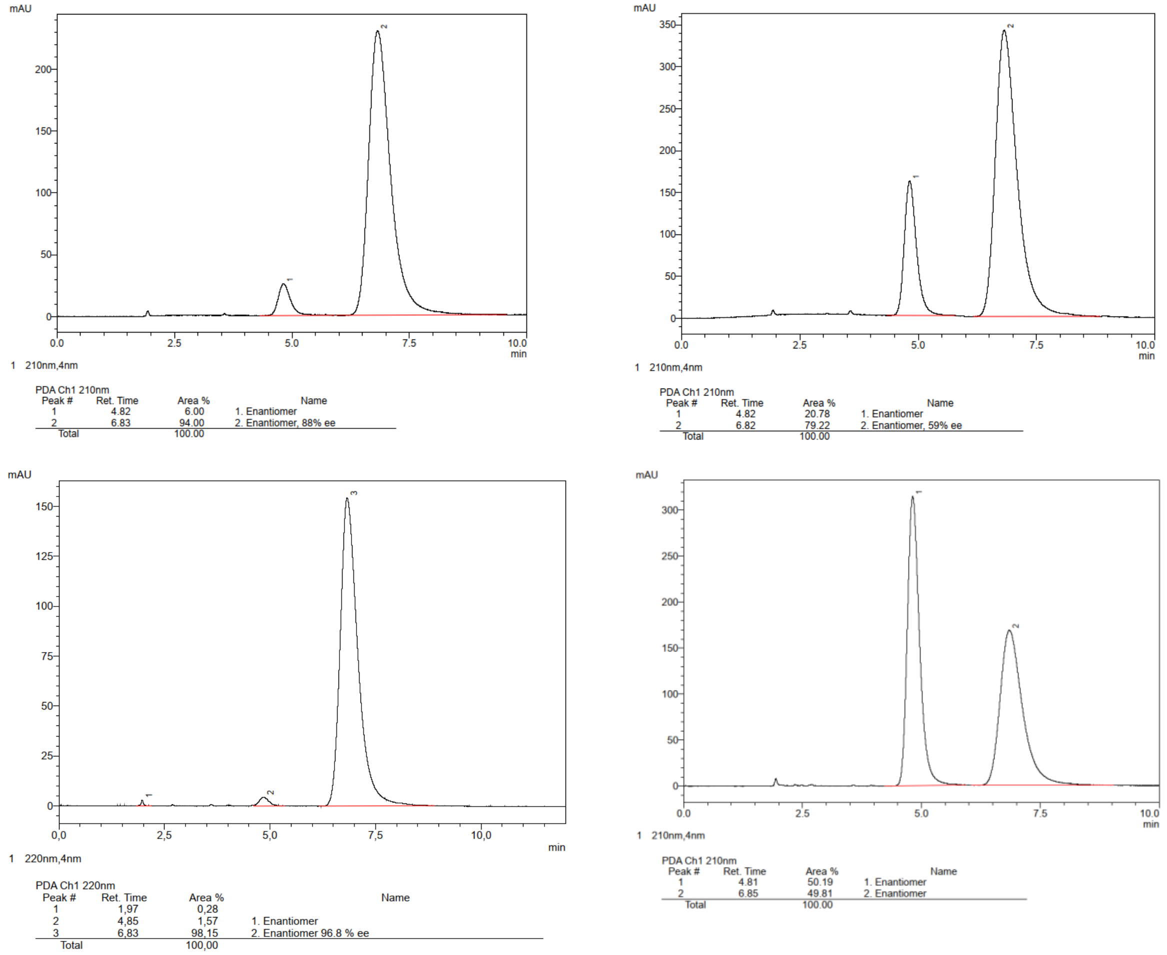

Figure S2. HPLC traces of compound **9a**: with catalyst **7b** (top, left); with [BiRh(*S*-PTTL)$_4$]·MeCN (**6a**) (top, right); with **7d** (bottom, left); the corresponding racemate (bottom, right).

C–H Insertion Reactions

General procedure A: Pentane as the Solvent. An oven-dried jacketed Schlenk flask equipped with a magnetic stir bar was charged with the [BiRh] catalyst (0.0005 mmol, 0.5 mol%) under argon. The substrate (0.25 mmol) and pentane (1 mL) were added to the catalyst and the resulting solution cooled to −10 °C. A solution of the diazo compound (0.1 mmol) in pentane (3 mL) was added dropwise over 60 min. The resulting mixture was stirred at −10 °C until TLC analysis indicated the complete consumption of the diazo compound. For work up, the mixture was absorbed on silica, which was loaded on top of a silica column.

Purification by flash chromatography (n-pentane/Et_2O or hexanes/EtOAc) afforded the desired C–H insertion product.

General procedure B: C_6F_6 as the Solvent. An oven dried Schlenk flask equipped with a magnetic stir bar was charged with the [BiRh] catalyst (0.0005 mmol, 0.5 mol%) under argon. The alkane substrate (0.4 mmol) and C_6F_6 (1 mL) were added. A solution of the diazo compound (0.1 mmol) in C_6F_6 (3 mL) was added dropwise over 20 min. The resulting mixture was stirred at ambient temperture until TLC analysis indicated the complete consumption of the diazo compound (5 min to 2 h). For work up, the mixture was absorbed on silica, which was loaded on top of a silica column. Purification by flash chromatography (n-pentane/Et_2O or hexanes/EtOAc) afforded the desired C–H insertion product.

Larger Scale Experiment. Preparation of 2,2,2-Trichloroethyl (*R*)-3-(cyclopentyloxy)-2-(4-fluorophenyl) propanoate (20d). An oven dried Schlenk flask equipped with a magnetic stir bar was charged with catalyst **7b** (4.4 mg, 0.0015 mmol, 0.1 mol%) under argon. Cyclopentyl methyl ether (0.875 mL, 7.5 mmol) and pentane (15 mL) were added and the resulting solution was cooled to –10 °C. A solution of the diazo derivative **8c** (468 mg, 1.5 mmol) in pentane (45 mL) was added dropwise over 2 h. The resulting mixture was stirred at –10 °C during 18 h. For work up, the mixture was absorbed on silica, which was then loaded on top of a silica column. Purification by flash chromatography (hexanes/*tert*-butyl methyl ether, 98:2) afforded the title compound as a colorless liquid (494.1 mg, 86% yield, 99% ee). The analytical data are compiled below.

Stereochemical Assignment. The absolute configuration of the products was assigned in analogy to the stereostructure of product **23b** determined by X-ray diffraction (Figure S1). In case of products **12** and **20e**, this tentative assignment could be confirmed by comparison with literature data.

Diazoester Decomposition. 2,2,2-Trichloroethyl 2-(4-fluorophenyl)-3-(2,2,2-trichloroethoxy)propanoate

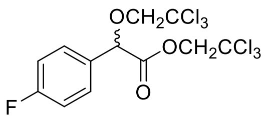

(11). An oven dried Schlenk flask equipped with a magnetic stir bar was charged with catalyst **7b** (1.5 mg, 0.0005 mmol, 0.5 mol%) under argon. C_6F_6 (1 mL) was added before a solution of the diazo derivative **8c** (0.1 mmol, 31.1 mg) in C_6F_6 (3 mL) was added dropwise over 2 h and the resulting mixture was stirred at ambient temperature for 18 h. For work up, the mixture was absorbed on silica, which was loaded on top of a silica column. Purification by flash chromatography (hexanes/*tert*-butyl methyl ether, 98:2) afforded the ttle compound as a colorless liquid (12.5 mg, 58% yield). ^1H NMR (600 MHz, $CDCl_3$) δ = 7.57 – 7.50 (m, 2H), 7.13 – 7.06 (m, 2H), 5.41 (s, 1H), 4.85 (d, *J* = 11.9 Hz, 1H), 4.72 (d, *J* = 11.9 Hz, 1H), 4.34 (d, *J* = 11.5 Hz, 1H), 4.12 (d, *J* = 11.5 Hz, 1H); ^{13}C

NMR (151 MHz, $CDCl_3$) δ = 168.3, 163.5 (d, J = 248.7 Hz), 130.4 (d, J = 3.3 Hz), 129.5 (d, J = 8.5 Hz), 116.0 (d, J = 21.9 Hz), 96.4, 94.4, 81.5, 81.2, 74.4; ^{19}F NMR (470 MHz, $CDCl_3$) δ = –111.7; IR (ATR): ṽ = 2962, 1725, 1613, 1501, 1424, 1370, 1281, 1255, 1219, 1151, 1116, 1063, 874, 801, 725, 602, 529 cm^{-1}; HRMS (EI^+) for $C_{12}H_9Cl_6FO_3Na$ $[M+Na^+]^+$: calcd: 452.8559, found: 452.8550.

C–H Insertion into the Pentane Solvent. An oven dried Schlenk flask equipped with a magnetic stir bar was charged with the [BiRh] catalyst **7d** (0.001 mmol, 1 mol%) and pentane (1 mL) under argon. A solution of the diazo derivative **8c** (0.1 mmol) in pentane (3 mL) was added dropwise over 10 min and the resulting mixture was stirred at RT for 10 min. The yield (85%) was determined by NMR analysis of the crude product using CH_2Br_2 as internal standard. The peak assignment for the determination of the regio- and diastereoselectivity followed a literature procedure (insertion at C2:C1: rr ≈ 64:36; with this catalyst, insertion at C3 was below the limits of detection; ratio of the diastereomers formed by insertion at C2: dr ≈ 78:22).[7]

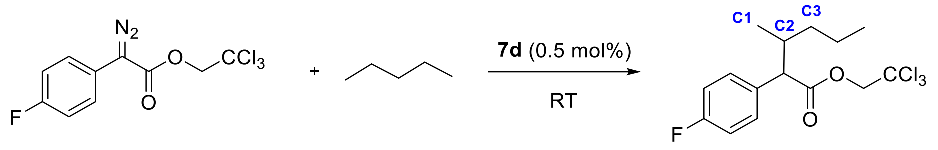

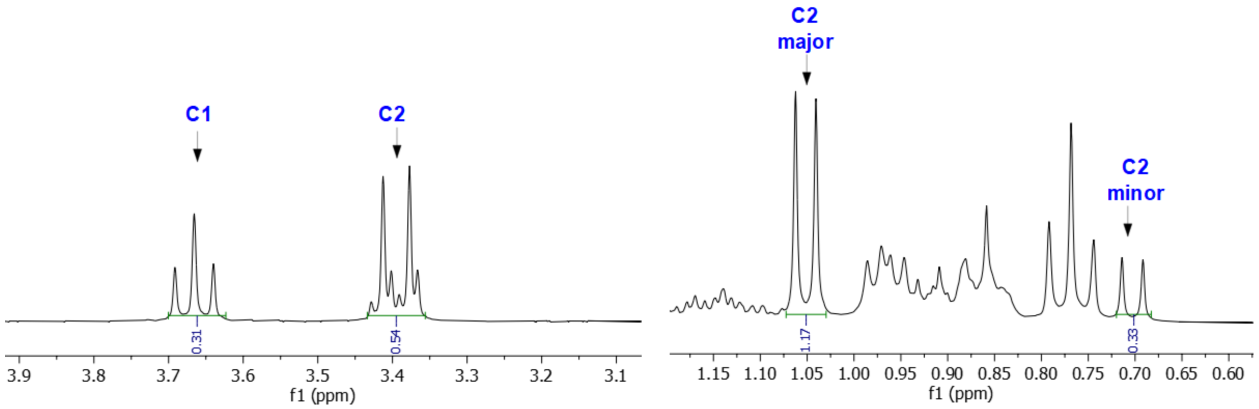

Methyl (*S*)-2-(cyclohexa-2,5-dien-1-yl)-2-(4-methoxyphenyl)acetate (12). Prepared at ambient temperature according to the general procedure **A** as a colorless oil; with [BiRh(*S*-PTTL)$_4$] (**6a**): 61%, 97% ee; with catalyst **7b**: 78%, 99% ee [The ee was determined by HPLC analysis: Daicel 150 mm Chiralcel OJ-3, Ø 4.6 mm, *n*-heptane/*iso*-propanol = 95/5, v = 1.0 mL/min, λ = 230 nm, t(minor) = 5.78 min, t(major) = 7.49 min].

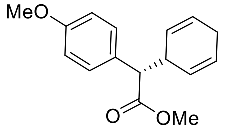

$[\alpha]_D^{20}$ = +143 (c = 0.6, CHCl$_3$); the literature reports for (*R*)-**12**: $[\alpha]_D^{21}$ = –126.1 (c = 1.18, CHCl$_3$).[8] This comparison further confirms the assignment originally based on comparison to the stereostructure of product **23b** (X-ray, Figure S1)

^{1}H NMR (400 MHz, CDCl$_3$): δ = 7.26 – 7.22 (m, 2H), 6.88 – 6.83 (m, 2H), 5.80 (dtt, *J* = 10.0, 3.2, 1.6 Hz, 1H), 5.73 – 5.64 (m, 2H), 5.33 – 5.25 (m, 1H), 3.80 (s, 3H), 3.67 (s, 3H), 3.44 (tddt, *J* = 9.0, 5.7, 3.1, 1.6 Hz, 1H), 3.36 (d, *J* = 10.37 Hz, 1H), 2.66 – 2.56 (m, 2H); ^{13}C NMR (101 MHz, CDCl$_3$): δ = 173.8, 159.0, 129.7, 128.9, 126.8, 126.3, 126.1, 125.9, 114.0, 57.6, 55.4, 52.0, 38.7, 26.5.

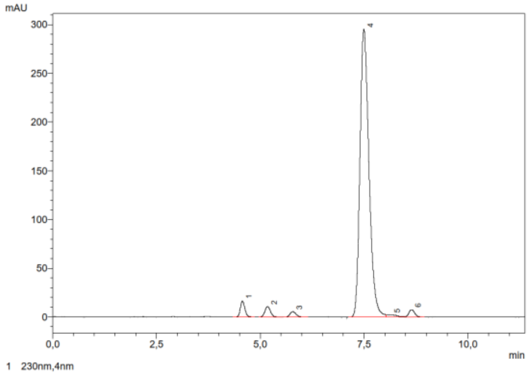

PDA Ch1 230nm

Peak #	Ret. Time	Area %	Name
1	4,57	2,62	
2	5,17	2,13	
3	5,78	1,23	1. Enantiomer
4	7,49	91,63	2. Enantiomer 97.4 % ee
5	8,14	0,84	
6	8,64	1,55	
Total		100,00	

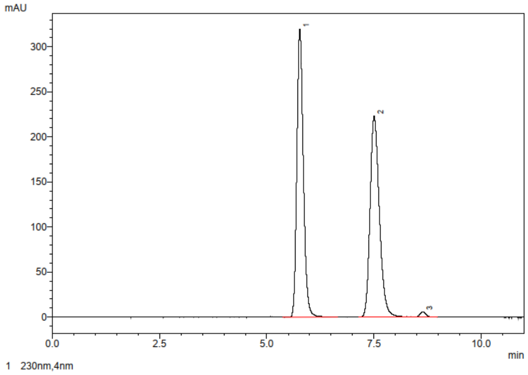

PDA Ch1 230nm

Peak #	Ret. Time	Area %	Name
1	5.77	49.49	1. Enantiomer
2	7.50	49.62	2. Enantiomer
3	8.65	0.89	
Total		100.00	

Figure S3. HPLC traces of compound **12**: with [BiRh(*S*-PTTL)$_4$] (**6a**) (left); the corresponding racemate (right).

2,2,2-Trichloroethyl (*R*)-2-(4-methoxyphenyl)-2-((tetrahydrofuran-2-yl)acetate (13). Prepared according to the general procedure **A** as a colorless oil; with [BiRh(*S*-PTTL)$_4$] (**6a**): 86%, 10:1 *dr*, 88% ee (major diastereomer), 78% ee (minor diastereomer); with catalyst **7b**: 89%, 52:1 *dr*, 99% ee (major diastereomer), 94% ee (minor diastereomer). [The ee was determined by 2D-HPLC analysis: Achiral separation: 50 mm Zorbax Eclipse Plus C18, 1.8 µm, Ø 4.6 mm, MeOH/water = 60/40, v = 1.0 mL/min, λ = 220 nm, t(minor) = 11.11 min, t(major) = 11.77 min; chiral separation: Daicel 150 mm Chiralcel OZ-3R, Ø 4.6 mm, MeCN/water = 50/50, v = 1.0 mL/min, λ = 230 nm, t(minor diastereomer, minor enantiomer) = 11.26 min, t(minor diastereomer, major enantiomer) = 12.32 min, t(major diastereomer, major enantiomer = 11.54 min, t(major diastereomer, major enantiomer) = 12.05 min]. $[\alpha]_D^{20}$ = −13.6 (c = 0.8, $CHCl_3$); ^1H NMR (400 MHz, $CDCl_3$): δ = 7.3 – 7.3 (m, 2H), 6.9 – 6.8 (m, 2H), 4.8 – 4.7 (m, 2H), 4.6 (dt, *J* = 10.0, 6.7 Hz, 1H), 4.0 – 3.9 (m, 1H), 3.8 (ddd, *J* = 8.3, 7.4, 6.1 Hz, 1H), 3.8 (s, 3H), 3.6 (d, *J* = 10.0 Hz, 1H), 1.9 – 1.8 (m, 2H), 1.8 – 1.7 (m, 1H), 1.5 (ddt, *J* = 12.4, 8.5, 6.9 Hz, 1H); ^{13}C NMR (101 MHz, $CDCl_3$): δ = 171.3, 159.4, 129.8, 127.2, 114.3, 95.0, 80.5, 74.2, 68.6, 56.8, 55.4, 29.6, 25.6; IR (ATR): ṽ = 2955, 1750, 1610, 1512, 1443, 1246, 1179, 1137, 1064, 1031, 920, 832, 791, 755, 717, 573, 530 cm^{-1}; HRMS (ESI$^+$) for $C_{15}H_{17}O_4Cl_3Na$ [M+Na$^+$]$^+$: calcd: 389.00846, found: 389.00842.

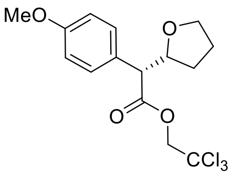

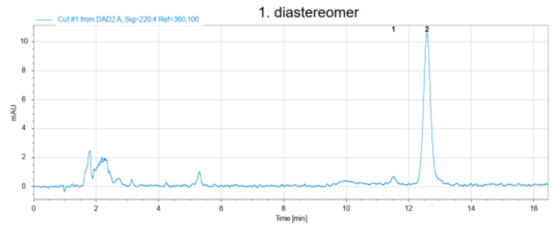

Signal: DAD2 A, Sig=220,4 Ref=360,100

Compound	Cut	Ret.Time	Area	Width	Height	Symmetry
1	1	11.516	4.855 **4.855**	0.163	0.498	0.943
2	1	12.587	160.226 **160.226**	0.184	10.278	0.952

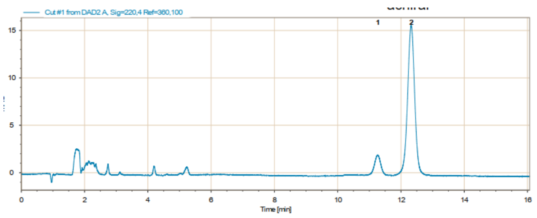

Signal: DAD2 A, Sig=220,4 Ref=360,100

Compound	Cut	Ret.Time	Area	Width	Height	Symmetry	
1	1	11.263	29.743 **29.743**	0.165	2.140	0.994	1. Diastereomer 1. Enantiomer
2	1	12.324	244.230 **244.230**	0.196	15.750	1.042	1. Diastereomer 2. Enantiomer

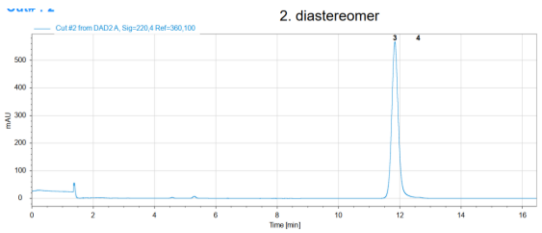

Signal: DAD2 A, Sig=220,4 Ref=360,100

Compound	Cut	Ret.Time	Area	Width	Height	Symmetry
3	2	11.841	8479.012 **8479.012**	0.249	566.975	0.890
4	2	12.608	48.057 **48.057**	0.215	3.731	0.508

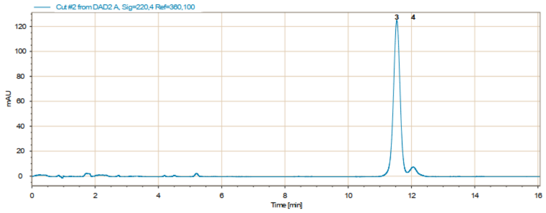

Signal: DAD2 A, Sig=220,4 Ref=360,100

Compound	Cut	Ret.Time	Area	Width	Height	Symmetry	
3	2	11.536	1848.523 **1848.523**	0.223	125.416	1.024	2. Diastereomer 1. Enantiomer
4	2	12.053	131.487 **131.487**	0.202	7.642	0.844	2. Diastereomer 2. Enantiomer

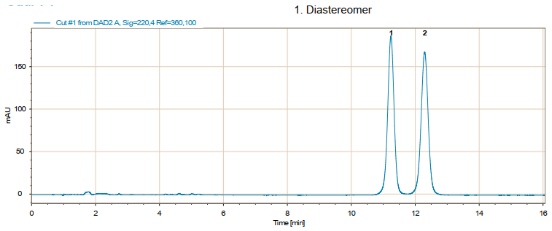

Signal: DAD2 A, Sig=220,4 Ref=360,100

Compound	Cut	Ret.Time	Area	Width	Height	Symmetry	
1	1	11.240	2655.173<br>**2655.173**	0.218	186.976	1.010	1. Diastereomer 1. Enantiomer
2	1	12.296	2662.848<br>**2662.848**	0.243	168.165	1.016	1. Diastereomer 2. Enantiomer

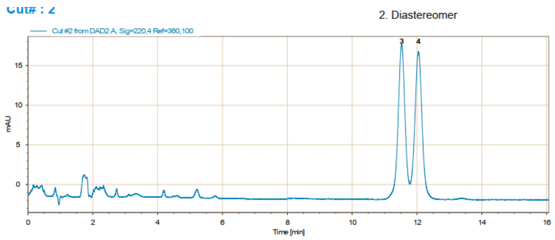

Signal: DAD2 A, Sig=220,4 Ref=360,100

Compound	Cut	Ret.Time	Area	Width	Height	Symmetry	
3	2	11.527	293.511<br>**293.511**	0.229	19.547	1.037	2. Diastereomer 1. Enantiomer
4	2	12.047	293.136<br>**293.136**	0.240	18.630	0.978	2. Diastereomer 2. Enantiomer

Figure S4. HPLC traces of compound **13**: with catalyst **7b** (top, left); with [BiRh(*S*-PTTL)$_4$] (**6a**) (top, right); the corresponding racemate (bottom).

2,2,2-Trichloroethyl (*R*)-2-(1,3-dioxolan-2-yl)-2-(4-fluorophenyl)acetate (14). Prepared according to the general procedure **A** as a colorless oil; with catalyst **7b**: 77%, 85% ee; with complex **7c**: 96%, 92% ee; with complex **7d**: 65%, 98% ee. [The ee was determined by HPLC analysis: Daicel 150 mm Chiralpak IA-3, Ø 4.6 mm, *n*-heptane/*iso*-propanol = 98/2, v = 1.0 mL/min, λ = 210 nm, t(minor) = 7.94 min, t(major) = 6.89 min.] $[\alpha]_D^{20}$ = +5.9 (c = 1.1, $CHCl_3$); ^{1}H NMR (400 MHz, $CDCl_3$): δ = 7.46 – 7.36 (m, 2H), 7.10 – 6.99 (m, 2H), 5.51 (d, *J* = 6.6 Hz, 1H), 4.78 (d, *J* = 1.2 Hz, 2H), 3.98 – 3.81 (m, 5H); ^{13}C NMR (101 MHz, $CDCl_3$): δ = 169.0, 162.8 (d, *J* = 246.9 Hz), 130.9 (d, *J* = 8.0 Hz), 129.1 (d, *J* = 3.2 Hz), 115.9 (dd, *J* = 27.2, 21.5 Hz), 104.2, 94.7, 74.3, 65.5, 55.7; ^{19}F NMR (282 MHz, $CDCl_3$): δ = −114.0; IR (ATR): ṽ = 2891, 1752, 1606, 1510, 1224, 1191, 1129, 1098, 1061, 1033, 943, 871, 838, 804, 758, 716, 573, 546, 520, 440 cm^{-1}, HRMS (ESI$^+$) for $C_{13}H_{12}O_4FCl_3Na$ [M+Na$^+$]$^+$: calcd: 378.96774, found: 378.96810.

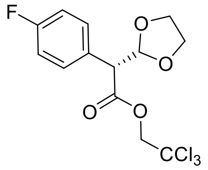

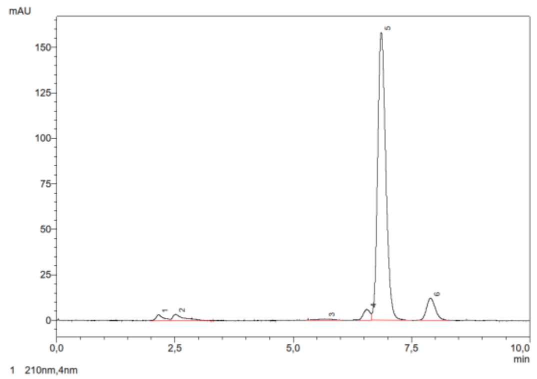

PDA Ch1 210nm

Peak #	Ret. Time	Area %	Name
1	2,15	1,72	
2	2,51	2,70	
3	5,68	0,63	
4	6,56	2,57	
5	6,86	85,24	1. Enantiomer 84.6 % ee
6	7,90	7,13	2. Enantiomer
Total		100,00	

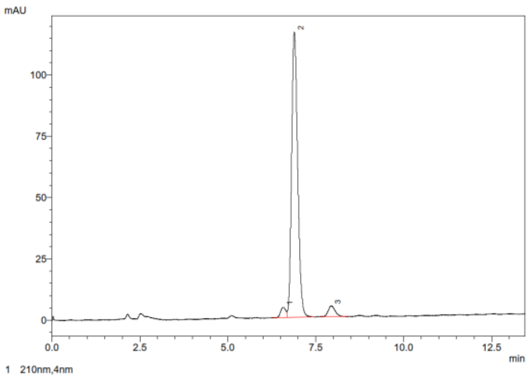

PDA Ch1 210nm

Peak #	Ret. Time	Area %	Name
1	6.58	2.81	
2	6.89	93.06	1. Enantiomer, ee=92%
3	7.94	4.12	2. Enantiomer
Total		100.00	

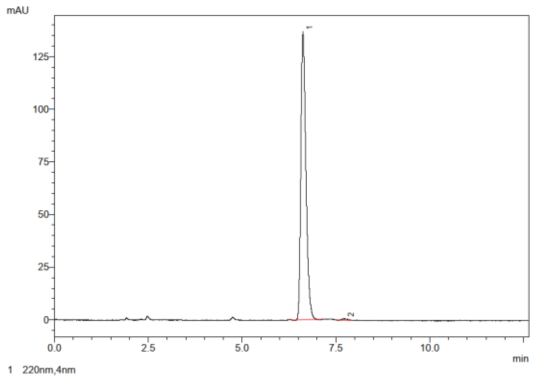

PDA Ch1 220nm

Peak #	Ret. Time	Area %	Name
1	6.62	99.46	
2	7.73	0.54	
Total		100.00	

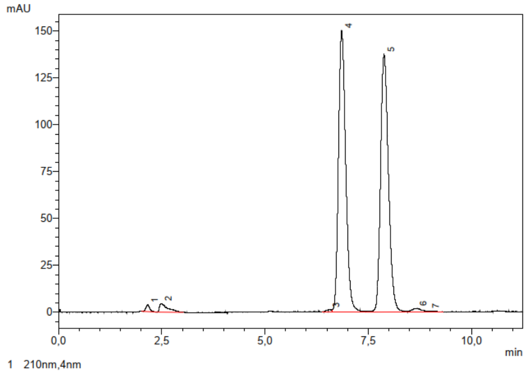

PDA Ch1 210nm

Peak #	Ret. Time	Area %	Name
1	2,16	0,60	
2	2,49	1,66	
3	6,55	0,31	
4	6,86	48,29	1. Enantiomer
5	7,88	48,12	2. Enantiomer
6	8,66	0,86	
7	8,97	0,15	
Total		100,00	

Figure S5. HPLC traces of compound **14**: with catalyst **7b** (top, left); with **7c** (top, right); with **7d** (bottom, left); the corresponding racemate (bottom, right).

2,2,2-Trichloroethyl (*R*)-2-(4-fluorophenyl)-2-(1,3,5-trioxan-2-yl)acetate (15). Prepared according to the general procedure **B** as a white solid; with complex **7b**: 75% yield, 94% ee. [The ee was determined by HPLC analysis: Daicel 150 mm Chiralpak IA-3, Ø 4.6 mm, n-heptane/i-propanol = 95/5, v = 1.0 mL/min, λ = 220 nm, t(major) = 6.18 min, t(minor) = 13.39 min]. $[\alpha]_D^{20}$ = 57.7 (c = 1.9, $CHCl_3$); ^{1}H NMR (400 MHz, $CDCl_3$): δ = 7.44 – 7.35 (m, 2H), 7.11 – 6.99 (m, 2H), 5.49 (d, *J* = 7.9 Hz, 1H), 5.24 (dd, *J* = 6.3, 1.3 Hz, 1H), 5.19 – 5.12 (m, 2H), 5.04 (d, *J* = 6.3 Hz, 1H), 4.79 (d, *J* = 12.0 Hz, 1H), 4.71 (d, *J* = 12.0 Hz, 1H), 4.07 (d, *J* = 7.9 Hz, 1H); ^{13}C NMR (101 MHz, $CDCl_3$): δ = 168.4, 162.9 (d, *J* = 247.5 Hz), 130.9 (d, *J* = 8.1 Hz), 128.0 (d, *J* = 3.5 Hz), 115.9 (d, *J* = 21.6 Hz), 101.1, 94.6, 93.5, 93.4, 74.4, 55.6; ^{19}F NMR (282 MHz, $CDCl_3$): δ = −113.50; IR (ATR): ṽ = 1756, 1741, 1604, 1511, 1377, 1328, 1314, 1214, 1168, 1138, 1095, 1062, 1010, 980, 946, 877, 842, 806, 756, 718, 564, 537 cm^{-1}; HRMS (ESI$^+$) for $C_{13}H_{12}Cl_3FO_5Na$ [M+Na]$^+$: calcd: 394.96266, found: 394.96298.

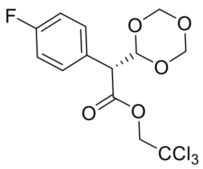

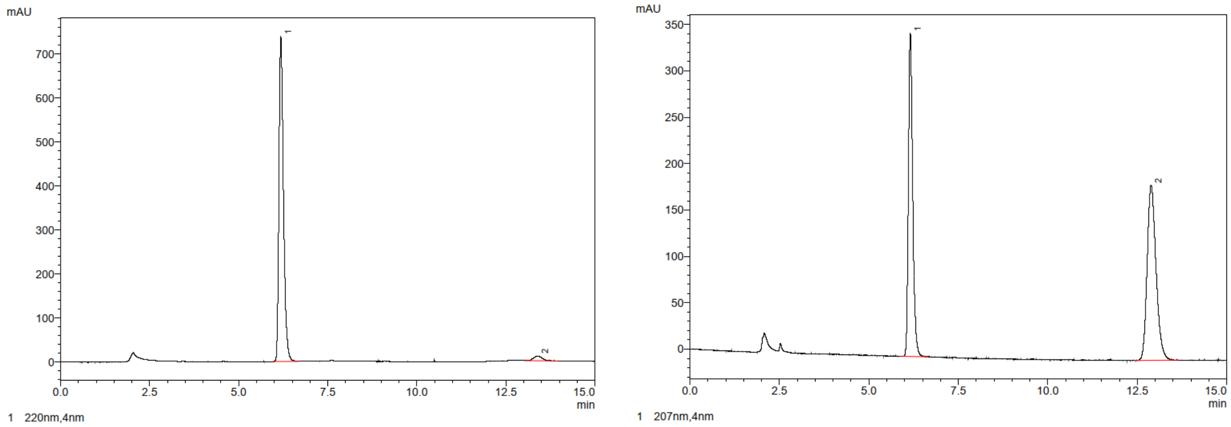

PDA Ch1 220nm

Peak #	Ret. Time	Area %
1	6.18	97.21
2	13.39	2.79
Total		100.00

PDA Ch1 207nm

Peak #	Ret. Time	Area %
1	6.16	47.49
2	12.89	52.51
Total		100.00

Figure S6. HPLC traces of compound **15**: with complex **7b** (left); the corresponding racemate (right).

2,2,2-Trichloroethyl (*R*)-3-(*tert*-butoxy)-2-(4-fluorophenyl)propanoate (16). Prepared according to the general procedure **A** as a colorless oil; with catalyst **7b**: 70%, 99% ee; with catalyst **7c**: 90%, 99% ee. [The ee was determined by HPLC analysis: Daicel 150 mm Chiralpak IB-N3, Ø 4.6 mm, *n*-heptane/*iso*-propanol = 95.9/0.1, v = 1.0 mL/min, λ = 220 nm, t(minor) = 5.07 min, t(major) = 5.40 min.] $[\alpha]_D^{20}$ = −10.9 (c = 1.1, $CHCl_3$); ^1H NMR (400 MHz, $CDCl_3$): δ = 7.40 – 7.30 (m, 2H), 7.07 – 6.96 (m, 2H), 4.79 (d, *J* = 12.0 Hz, 1H), 4.71 (d, *J* = 12.0 Hz, 1H), 4.02 – 3.88 (m, 2H), 3.58 (dd, *J* = 7.8, 4.4 Hz, 1H), 1.17 (s, 9H); ^{13}C NMR (101 MHz, $CDCl_3$): δ = 171.2, 162.5 (d, *J* = 246.5 Hz), 131.2 (d, *J* = 3.2 Hz), 130.1 (d, *J* = 8.1 Hz), 115.7 (d, *J* = 21.5 Hz), 95.0, 74.3, 73.6, 63.9, 52.1, 27.5; ^{19}F NMR (282 MHz, $CDCl_3$): δ = −114.53; IR (ATR): ṽ = 2974, 1754, 1606, 1509, 1364, 1229, 1193, 1138, 1088, 1046, 908, 837, 804, 753, 736, 717, 630, 569, 519, 429 cm^{-1}; HRMS (ESI) for $C_{15}H_{18}O_3Cl_3FNa$ $[M+Na]^+$: calcd: 393.01978, found: 393.02013.

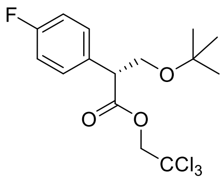

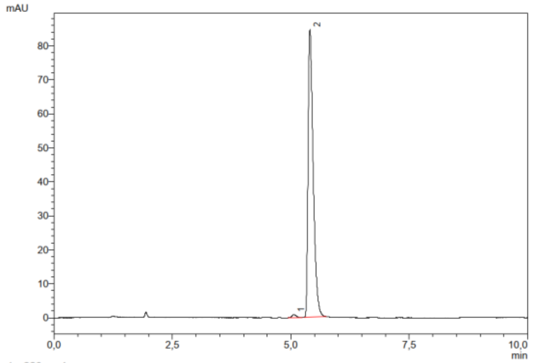

1 220nm,4nm

PDA Ch1 220nm

Peak #	Ret. Time	Area %	Name
1	5,07	0,92	1. Enantiomer
2	5,40	99,08	2. Enantiomer
Total		100,00	

ee = 98.9%

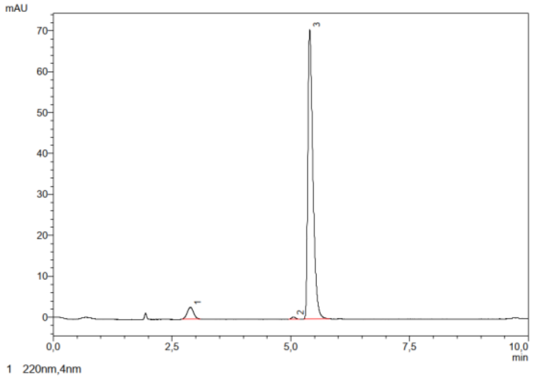

1 220nm,4nm

PDA Ch1 220nm

Peak #	Ret. Time	Area %	Name
1	2,89	4,71	
2	5,05	0,55	1. Enantiomer
3	5,40	94,74	2. Enantiomer
Total		100,00	

ee = 98.8%

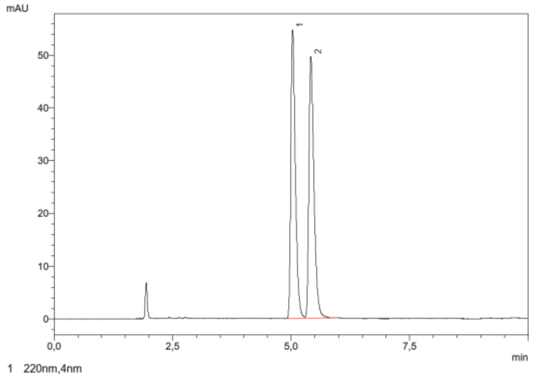

1 220nm,4nm

PDA Ch1 220nm

Peak #	Ret. Time	Area %	Name
1	5,03	49,81	1. Enantiomer
2	5,42	50,19	2. Enantiomer
Total		100,00	

Figure S7. HPLC traces of compound **16**: with catalyst **7b** (top, left); with catalyst **7c** (top, right); the corresponding racemate (bottom).

2,2,2-Trichloroethyl (*R*)-3-((*tert*-butyldimethylsilyl)oxy)-2-(4-fluorophenyl)propanoate (17). Prepared according to the general procedure **A** as a colorless oil; with catalyst **7b**: 59%, 98% ee; with catalyst **7d**: 70%, 99% ee. [The ee was determined by HPLC analysis: Daicel 150 mm Chiralpak IB-N-3, Ø 4.6 mm, n-heptane/iso-propanol = 99.99/0.01, v = 1.0 mL/min, λ = 210 nm, t(minor) = 4.03 min, t(major) = 4.26 min.] $[\alpha]_D^{20}$ = +2.5 (c = 1.0, $CHCl_3$); ^1H NMR (400 MHz, $CDCl_3$): δ = 7.38 – 7.29 (m, 2H), 7.07 – 6.97 (m, 2H), 4.78 (d, *J* = 12.0 Hz, 1H), 4.71 (d, *J* = 12.0 Hz, 1H), 4.19 (dd, *J* = 9.5, 8.6 Hz, 1H), 3.93 (dd, *J* = 8.6, 5.6 Hz, 1H), 3.84 (dd, *J* = 9.5, 5.6 Hz, 1H), 0.85 (s, 9H), 0.02 (d, *J* = 6.3 Hz, 6H); ^{13}C NMR (101 MHz, $CDCl_3$): δ = 171.0, 162.6 (d, *J* = 246.4 Hz), 131.0 (d, *J* = 3.1 Hz), 130.2 (d, *J* = 8.1 Hz), 115.7 (d, *J* = 21.6 Hz), 94.9, 74.3, 65.2, 53.9, 25.9, 18.3, −5.4; ^{19}F NMR (282 MHz, $CDCl_3$): δ = −114.5; IR (ATR): ṽ = 2929, 2857, 1755, 1606, 1510, 1464, 1255, 1230, 1141, 1097, 1068, 1006, 890, 834, 807, 777, 717, 665, 574, 548, 518, 430 cm^{-1}; HRMS (ESI^+) for $C_{17}H_{24}O_3FCl_3SiNa$ $[M+Na^+]^+$: calcd: 451.04366, found: 451.04369.

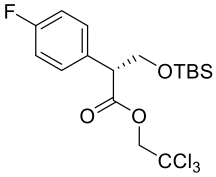

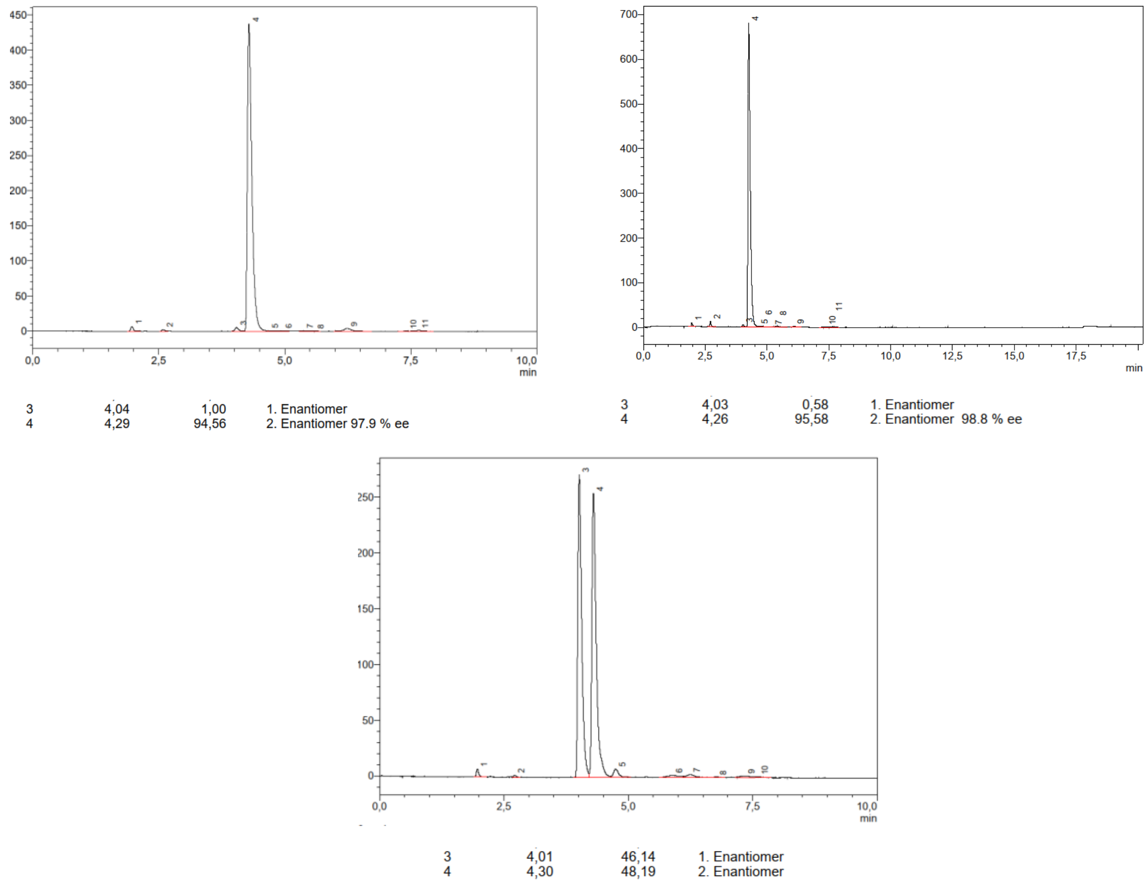

Figure S8. HPLC traces of compound **17**: with catalyst **7b** (top, left); with **7d** (top, right); the corresponding racemate (bottom).

2,2,2-Trichloroethyl (*R*)-2-(4-fluorophenyl)-3-(methoxymethoxy)propanoate (18). Prepared according to the general procedure **A** as a colorless oil; with catalyst **7b**: 52%, 99% ee. [The ee was determined by HPLC analysis: Daicel 150 mm Chiralcel OJ-3, Ø 4.6 mm, *n*-heptane/*iso*-propanol = 98/2, v = 1.0 mL/min, λ = 210 nm, t(minor) = 7.55 min, t(major) = 6.29 min.] $[\alpha]_D^{20}$ = −12.2 (c = 0.5, $CHCl_3$); ^{1}H NMR (300 MHz, $CDCl_3$): δ = 7.40 – 7.29 (m, 2H), 7.11 – 6.96 (m, 2H), 4.82 – 4.70 (m, 2H), 4.67 – 4.59 (m, 2H), 4.17 (t, *J* = 9.3 Hz, 1H), 4.03 (dd, *J* = 9.2, 5.3 Hz, 1H), 3.81 (dd, *J* = 9.4, 5.3 Hz, 1H), 3.32 (s, 3H); ^{13}C NMR (101 MHz, $CDCl_3$): δ = 170.7, 162.6 (d, *J* = 246.9 Hz), 130.7 (d, *J* = 3.5 Hz), 130.1 (d, *J* = 8.0 Hz), 115.9 (d, *J* = 21.6 Hz), 96.8, 94.8, 74.3, 68.9, 55.6, 51.4; ^{19}F NMR (282 MHz, $CDCl_3$): δ = −114.1; IR (ATR): ṽ = 2887, 1752, 1605, 1510, 1225, 1145, 1108, 1035, 918, 838, 804, 744, 717, 574, 555, 519, 444 cm^{-1}; HRMS (ESI^+) for $C_{13}H_{14}O_4FCl_3Na$ $[M+Na^+]^+$: calcd: 380.98339, found: 380.98354.

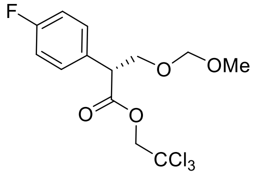

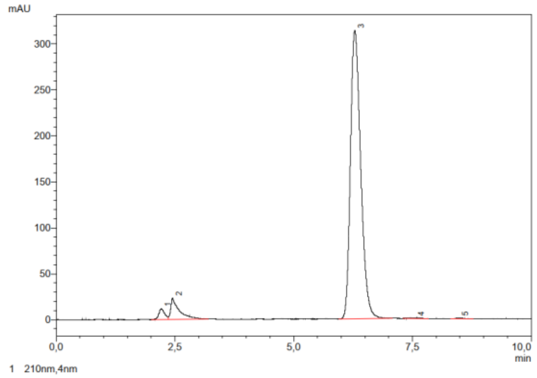

PDA Ch1 210nm

Peak #	Ret. Time	Area %	Name
1	2,21	2,01	
2	2,45	5,52	
3	6,29	92,07	1. Enantiomer 99.5 % ee
4	7,55	0,23	2. Enantiomer
5	8,48	0,17	
Total		100,00	

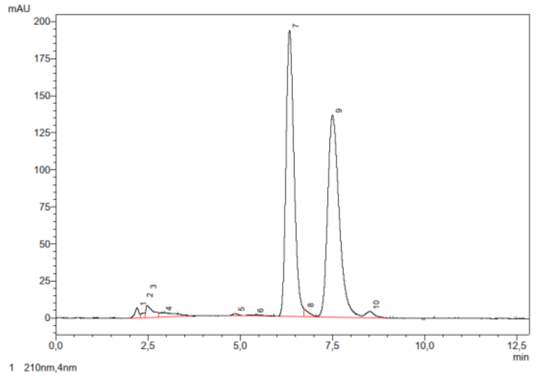

PDA Ch1 210nm

Peak #	Ret. Time	Area %	Name
1	2,20	0,84	
2	2,38	0,39	
3	2,47	1,77	
4	2,89	1,48	
5	4,86	0,28	
6	5,38	0,28	
7	6,34	46,39	1. Enantiomer
8	6,74	0,75	
9	7,50	46,64	2. Enantiomer
10	8,52	1,18	
Total		100,00	

Figure S9. HPLC traces of compound **18**: with catalyst **7b** (left); the corresponding racemate (right).

2,2,2-Trichloroethyl (*R*)-3-((4-bromobenzyl)oxy)-2-(4-fluorophenyl)propanoate (19). Prepared according to the general procedure **B** as a colorless liquid; with complex **7b**: 56% yield, 99% ee. [The ee was determined by HPLC analysis: Daicel 150 mm Chiralpak IB-N-3, Ø 4.6 mm, n-heptane/iso-propanol = 99/1, v = 1.0 mL/min, λ = 220 nm, t(minor) = 5.41 min, t(major) = 5.90 min]. $[\alpha]_D^{20}$ = 11.8 (c = 0.65, $CHCl_3$); ^{1}H NMR (400 MHz, $CDCl_3$): δ = 7.51 – 7.41 (m, 2H), 7.36 – 7.27 (m, 2H), 7.19 – 7.10 (m, 2H), 7.07 – 6.97 (m, 2H), 4.76 (d, *J* = 12.0 Hz, 1H), 4.73 (d, *J* = 12.0 Hz, 1H), 4.55 – 4.46 (m, 2H), 4.12 – 4.00 (m, 2H), 3.71 (dd, *J* = 7.1, 3.3 Hz, 1H); ^{13}C NMR (101 MHz, $CDCl_3$): δ = 170.6, 162.6 (d, *J* = 246.9 Hz), 136.9, 131.7, 130.6 (d, *J* = 3.4 Hz), 130.1 (d, *J* = 8.2 Hz), 129.4, 121.8, 115.9 (d, *J* = 21.4 Hz), 94.8, 74.3, 72.8, 71.5, 51.4; ^{19}F NMR (282 MHz, $CDCl_3$): δ = –114.0 (tt, *J* = 8.4, 5.2 Hz); IR (ATR): ṽ = 2953, 2865, 1752, 1605, 1509, 1487, 1372, 1227, 1142, 1094, 1070, 1011, 908, 837, 794, 717, 574, 518, 481 cm^{-1}; HRMS (EI$^+$) for $C_{18}H_{15}BrCl_3FO_3$ [M]$^+$: calcd: 481.92488, found: 481.92462.

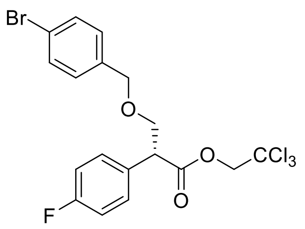

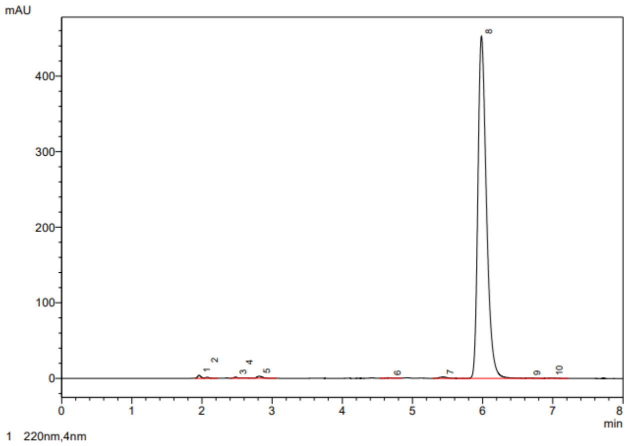

PDA Ch1 220nm

Peak #	Ret. Time	Area %	Name
1	1,96	0,34	
2	2,07	0,12	
3	2,48	0,13	
4	2,57	0,14	
5	2,81	0,40	
6	4,68	0,11	
7	5,43	0,36	1. Enantiomer
8	5,98	98,12	2. Enantiomer > 99 % (99.3%) ee
9	6,67	0,14	
10	6,99	0,12	
Total		100,00	

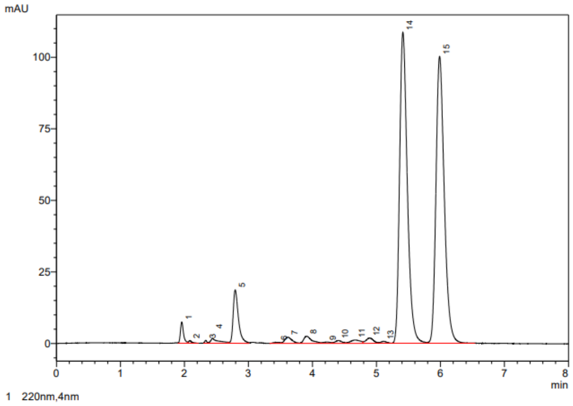

PDA Ch1 220nm

Peak #	Ret. Time	Area %	Name
1	1,96	1,21	
2	2,08	0,15	
3	2,33	0,11	
4	2,44	0,71	
5	2,79	5,20	
6	3,45	0,13	
7	3,61	0,83	
8	3,91	1,06	
9	4,21	0,19	
10	4,40	0,35	
11	4,67	0,59	
12	4,89	0,73	
13	5,11	0,21	
14	5,41	44,50	1. Enantiomer
15	5,99	44,02	2. Enantiomer
Total		100,00	

Figure S10. HPLC traces of compound **19**: with complex **7b** (left); the corresponding racemate (right).

2,2,2-Trichloroethyl (*R*)-3-(cyclopentyloxy)-2-phenylpropanoate (20a). Prepared according to the general procedure **A** as a colorless liquid; with complex **7b**: 71% yield, 95% ee; general procedure **B** as a colorless liquid; with complex **7b**: 69% yield, >99% ee. [The ee was determined by HPLC analysis: Daicel 150 mm Chiralpak OJ-3R, Ø 4.6 mm, methanol/water = 90/10, v = 0.5 mL/min, λ = 210 nm, t(major) = 10.22 min, t(minor) = 11.19 min]. $[\alpha]_D^{20}$ = 4.5 (c = 1.31, $CHCl_3$); ^1H NMR (400 MHz, $CDCl_3$): δ = 7.52 – 7.27 (m, 5H), 4.79 (d, *J* = 12.0 Hz, 1H), 4.72 (d, *J* = 12.0 Hz, 1H), 4.06 – 3.97 (m, 2H), 3.96 – 3.89 (m, 1H), 3.65 (dd, *J* = 7.5, 3.3 Hz, 1H), 1.76 – 1.59 (m, 6H), 1.52 – 1.44 (m, 2H); ^{13}C NMR (101 MHz, $CDCl_3$): δ = 171.2, 135.2, 128.9, 128.4, 128.0, 95.0, 82.1, 74.3, 70.3, 52.5, 32.3, 32.2, 23.7; IR (ATR): ṽ = 2956, 2870, 1753, 1452, 1348, 1262, 1138, 1095, 801, 716, 697, 571 cm^{-1}; HRMS (ESI^+) for $C_{16}H_{19}Cl_3NaO_3$ $[M+Na]^+$: calcd: 387.02920, found: 387.02885.

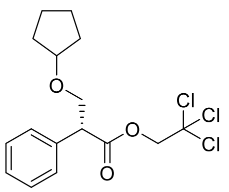

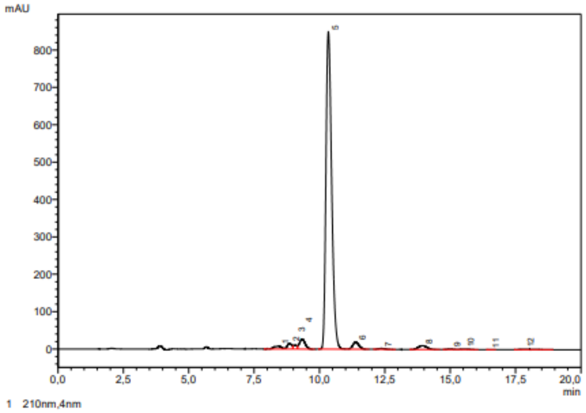

PDA Ch1 210nm

Peak #	Ret. Time	Area %	Name
1	8,44	1,07	
2	8,85	1,26	
3	9,06	0,71	
4	9,34	2,85	
5	10,34	89,05	1. Enantiomer 95.2 % ee
6	11,39	2,17	2. Enantiomer
7	12,37	0,33	
8	13,94	1,66	
9	15,00	0,20	
10	15,53	0,27	
11	16,48	0,01	
12	17,81	0,41	
Total		100,00	

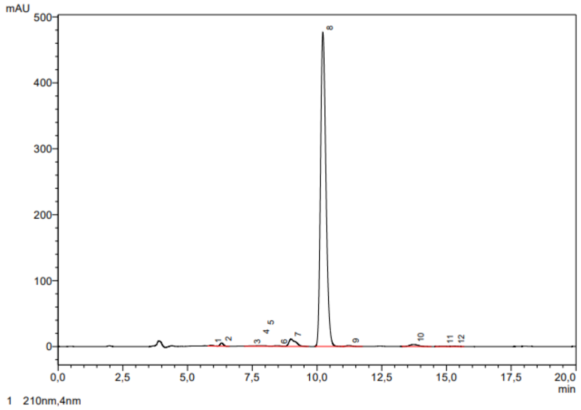

PDA Ch1 210nm

Peak #	Ret. Time	Area %	Name
1	5,92	0,16	
2	6,32	0,57	
3	7,42	0,18	
4	7,77	0,18	
5	7,94	0,22	
6	8,46	0,28	
7	9,00	2,81	
8	10,23	94,06	1. Enantiomer > 99 % ee
9	11,23	0,31	2. Enantiomer
10	13,74	0,99	
11	14,87	0,11	
12	15,29	0,14	
Total		100,00	

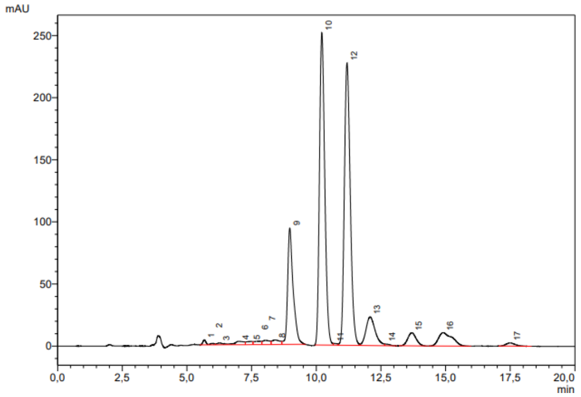

Figure S11. HPLC traces of compound **20a**: with complex **7b**; procedure **A** (top left); with procedure **B** (top right); the corresponding racemate (bottom).

2,2,2-Trichloroethyl (*R*)-3-(cyclopentyloxy)-2-(p-tolyl)propanoate (20b). Prepared according to the general procedure **A** as a colorless liquid; with complex **7b**: 77% yield, 99% ee; general procedure **B** as a colorless liquid; with complex **7b**: 64% yield, 98% ee. [The ee was determined by HPLC analysis: Daicel 150 mm Chiralpak IG-3, Ø 4.6 mm, methanol/water = 95/5, v = 1.0 mL/min, λ = 220 nm, t(major) = 4.28 min, t(minor) = 5.08 min]. $[\alpha]_D^{20}$ = 5.5 (c = 0.53, $CHCl_3$); ^1H NMR (400 MHz, $CDCl_3$): δ = 7.32 – 7.22 (m, 2H), 7.21 – 7.13 (m, 2H), 4.83 (d, *J* = 11.9 Hz, 1H), 4.74 (d, *J* = 11.9 Hz, 1H), 4.08 – 3.92 (m, 3H), 3.65 (dd, *J* = 8.1, 3.9 Hz, 1H), 2.37 (s, 3H), 1.82 – 1.63 (m, 6H), 1.57 – 1.49 (m, 2H); ^{13}C NMR (101 MHz, $CDCl_3$): δ = 171.3, 137.7, 132.1, 129.5, 128.2, 95.0, 82.1, 74.3, 70.4, 52.1, 32.3, 32.2, 23.7, 21.2; IR (ATR): ṽ = 2954, 2869, 1754, 1514, 1345, 1138, 1095, 820, 798, 717, 573, 506 cm^{-1}; HRMS (ESI^+) for $C_{17}H_{21}Cl_3NaO_3$ $[M+Na]^+$: calcd: 401.04485, found: 401.04466.

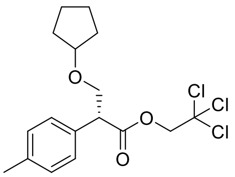

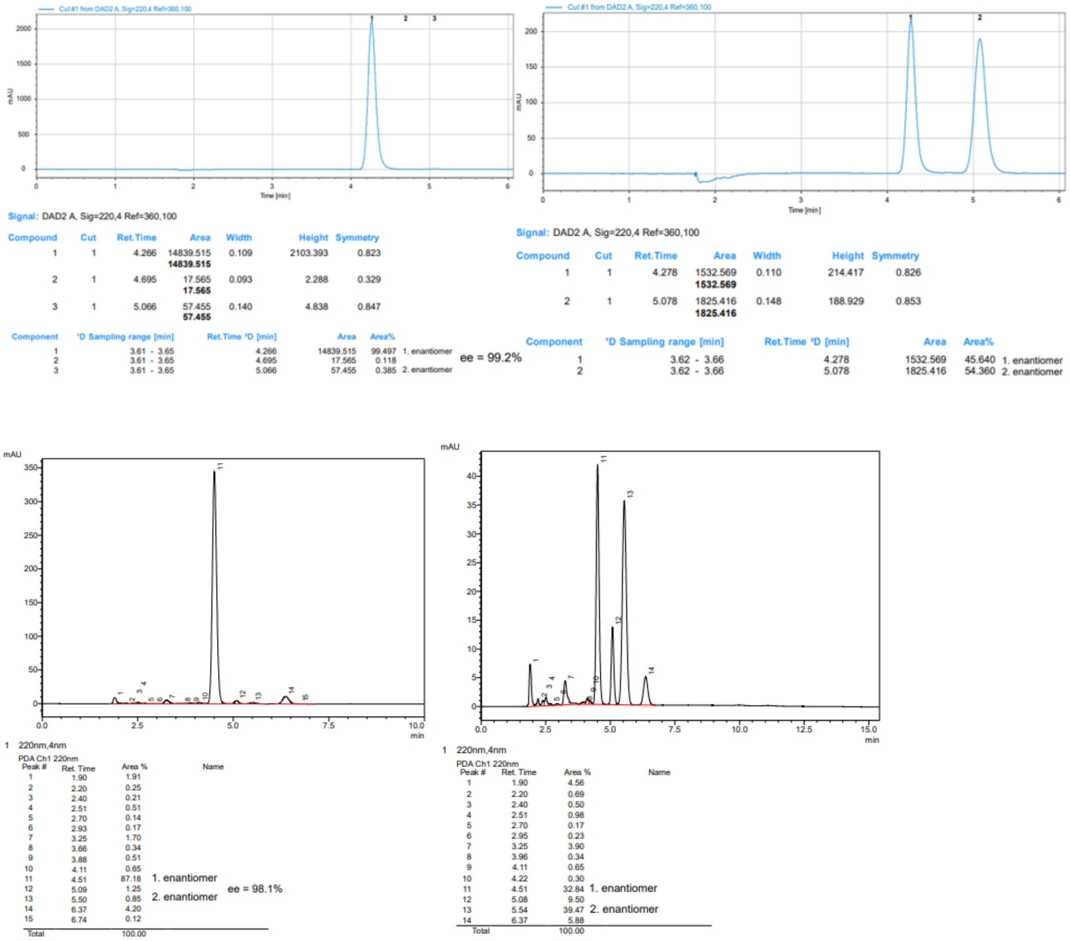

Figure S12. HPLC traces of compound **20b**: following procedure **A** with complex **7b** (top left); the corresponding racemate (top right); following procedure **B** with complex **7b** (bottom left); the corresponding racemate (bottom right).

Methyl (*R*)-4-(3-(cyclopentyloxy)-1-oxo-1-(2,2,2-trichloroethoxy)propan-2-yl)benzoate (20c). Prepared according to the general procedure **A** in pentane/CH_2Cl_2 as a colorless liquid; with complex **7b**: 62% yield, >99% ee. [The ee was determined by HPLC analysis: Daicel 150 mm Chiralpak IG-3, Ø 4.6 mm, methanol/water = 70% to 95% methanol in 10 min, v = 1.0 mL/min, λ = 220 nm, t(minor) = 16.23 min, t(major) = 16.89 min]. $[\alpha]_D^{20}$ = 6.5 (c = 1.06, $CHCl_3$); ^1H NMR (400 MHz, $CDCl_3$): δ = 8.04 – 7.96 (m, 2H), 7.48 – 7.37 (m, 2H), 4.76 (d, *J* = 0.7 Hz, 2H), 4.11 – 3.99 (m, 2H), 3.94 – 3.91 (m, 4H), 3.68 (dd, *J* = 7.6, 3.8 Hz, 1H), 1.75 – 1.57 (m, 6H), 1.54 – 1.42 (m, 2H); ^{13}C NMR (101 MHz, $CDCl_3$): δ = 170.5, 166.9, 140.3, 130.1, 129.9, 128.6, 94.8, 82.2, 74.4, 69.8, 52.5, 52.3, 32.3, 32.2, 23.6; IR (ATR): ṽ = 2953, 2870, 1754, 1721, 1612, 1435, 1217, 1182, 1141, 1097, 1020, 801, 718, 572 cm^{-1}; HRMS (ESI^+) for $C_{18}H_{22}Cl_3O_5$ $[M+H]^+$: calcd: 423.05273, found: 423.05308.

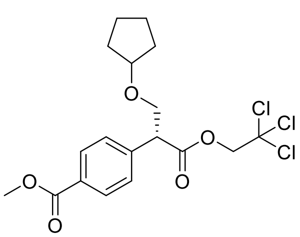

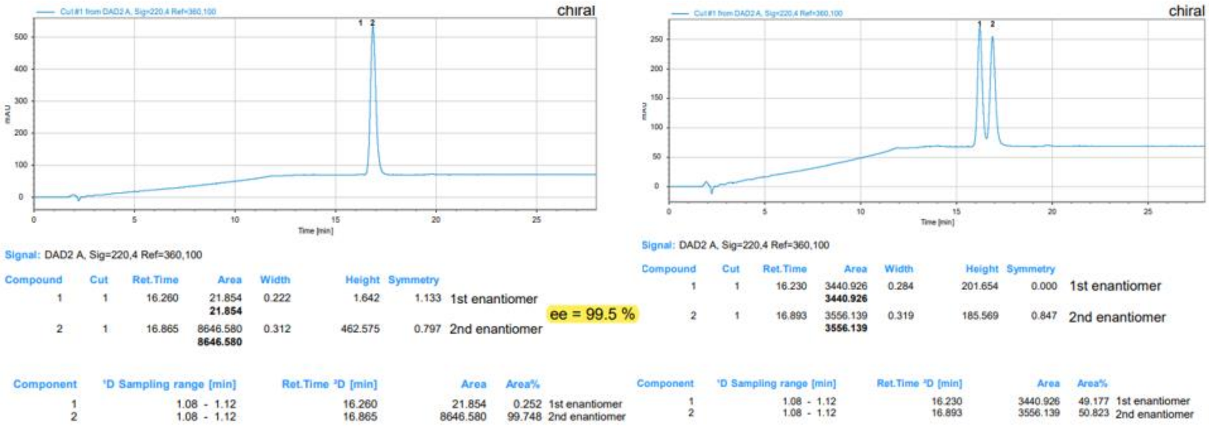

Figure S13. HPLC traces of compound **20c**: with complex **7b** (left); the corresponding racemate (right).

2,2,2-Trichloroethyl (*R*)-3-(cyclopentyloxy)-2-(4-fluorophenyl)propanoate (20d). Prepared according to

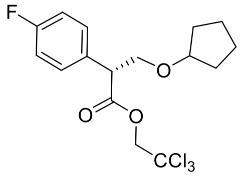

the general procedure **A** as a colorless oil; with catalyst **7b** (500 mg scale, see above): 86%, 99% ee; with **7c**: 90%, 99% ee. [The ee was determined by HPLC analysis: Daicel 150 mm Chiralpak AS-3R, Ø 4.6 mm, MeCN/water = 50/50, v = 1.0 mL/min, λ = 220 nm, t(minor) = 21.70 min, t(major) = 23.16 min.] $[\alpha]_D^{20}$ = −11.1 (c = 1.1, $CHCl_3$); ^1H NMR (400 MHz, $CDCl_3$): δ = 7.37 – 7.30 (m, 2H), 7.05 – 6.98 (m, 2H), 4.79 – 4.70 (m, 2H), 4.02 – 3.90 (m, 3H), 3.68 – 3.59 (m, 1H), 1.70 – 1.44 (m, 8H); ^{13}C NMR (101 MHz, $CDCl_3$): δ = 171.0, 162.6 (d, *J* = 246.6 Hz), 131.0 (d, *J* = 3.4 Hz), 130.1 (d, *J* = 8.1 Hz), 115.7 (d, *J* = 21.2 Hz), 94.9, 82.2, 74.3, 70.2, 51.7, 32.3, 32.2, 23.6; ^{19}F NMR (282 MHz, $CDCl_3$): δ = −114.4; IR (ATR): ṽ = 2956, 2870, 1753, 1606, 1509, 1346, 1227, 1139, 1096, 1047, 837, 801, 743, 717, 574, 517, 422 cm^{-1}; HRMS (ESI) for $C_{16}H_{18}O_3FCl_3Na$ $[M+Na]^+$: calcd: 405.01978, found: 405.02004.

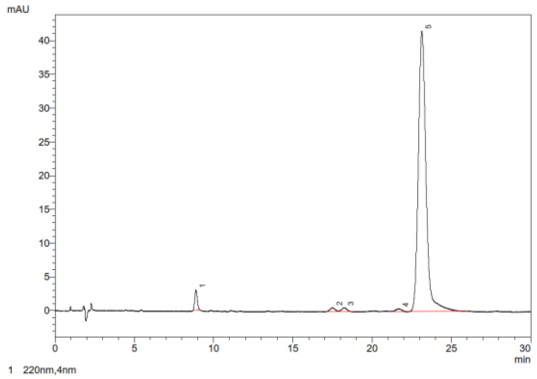

1 220nm,4nm

PDA Ch1 220nm

Peak #	Ret. Time	Area %	Name
1	8.89	2.27	
2	17.53	0.93	
3	18.24	0.93	
4	21.69	0.72	1. Enantiomer
5	23.14	95.14	2. Enantiomer
Total		100.00	

ee = 98.5 %

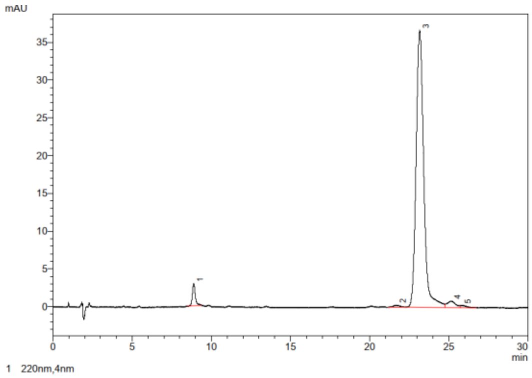

1 220nm,4nm

PDA Ch1 220nm

Peak #	Ret. Time	Area %	Name
1	8.89	2.90	
2	21.70	0.49	1. Enantiomer
3	23.16	93.64	2. Enantiomer
4	25.14	2.41	
5	25.81	0.57	
Total		100.00	

ee = 99.0 %

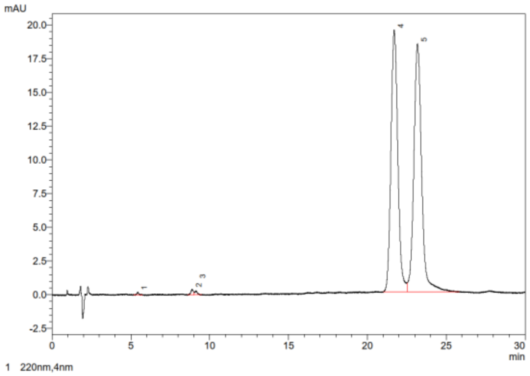

1 220nm,4nm

PDA Ch1 220nm

Peak #	Ret. Time	Area %	Name
1	5.43	0.12	
2	8.90	0.37	
3	9.14	0.27	
4	21.70	47.87	1. Enantiomer
5	23.18	51.38	2. Enantiomer
Total		100.00	

Figure S14. HPLC traces of compound **20d**: with catalyst **7b** (top, left); with **7c** (top, right); the corresponding racemate (bottom).

2,2,2-Trichloroethyl (*R*)-3-(cyclopentyloxy)-2-(4-bromophenyl)propanoate (20e). Prepared according to the general procedure **A** as a yellow liquid; with complex **7b**: 79% yield, 99% ee; general procedure **B** with complex **7b**: 60% yield, >99% ee. [The ee was determined by HPLC analysis: Daicel 150 mm Chiralpak OJ-3R, Ø 4.6 mm, acetonitrile/water = 80/20, v = 0.5 mL/min, λ = 225 nm, t(major) = 7.40 min, t(minor) = 7.79 min].

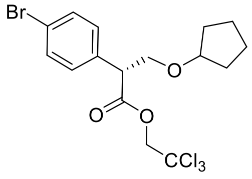

$[\alpha]_D^{20}$ = –2.1 (c = 0.5, $CHCl_3$); the literature reports for (*R*)-**20d**: $[\alpha]_D^{20}$ = –1.47 (c = 1.07, $CHCl_3$).[9] This comparison further confirms the assignment originally based on comparison to the stereostructure of product **23b** (X-ray, Figure S1)

^{1}H NMR (400 MHz, $CDCl_3$): δ = 7.50 – 7.42 (m, 2H), 7.25 –7.23 (m, 2H), 4.77 (d, *J* = 11.9 Hz, 1H), 4.73 (d, *J* = 11.9 Hz, 1H), 4.01 – 3.94 (m, 2H), 3.94 – 3.89 (m, 1H), 3.68 – 3.60 (m, 1H), 1.73 – 1.56 (m, 6H), 1.52 – 1.44 (m, 2H); ^{13}C NMR (101 MHz, $CDCl_3$): δ = 170.7, 134.3, 132.0, 130.2, 122.1, 94.9, 82.2, 74.4, 69.9, 51.9, 32.3, 32.2, 23.6; IR (ATR): ṽ = 2972, 2865, 1725, 1610, 1435, 1369, 1278, 1103, 1019, 720, 571 cm^{-1}; HRMS (ESI$^+$) for $C_{16}H_{18}BrCl_3O_3Na$ $[M+Na]^+$: calcd: 464.93972, found: 464.93973.

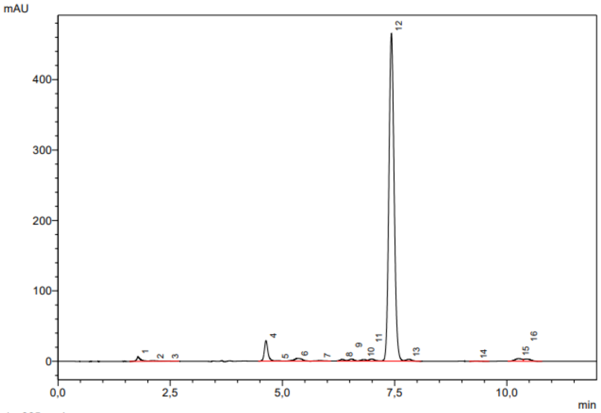

1 225nm,4nm

PDA Ch1 225nm

Peak #	Ret. Time	Area %	Name
1	1,78	0,93	
2	2,12	0,28	
3	2,45	0,11	
4	4,63	3,79	
5	4,90	0,14	
6	5,34	1,23	
7	5,84	0,30	
8	6,33	0,46	
9	6,54	0,59	
10	6,81	0,47	
11	6,99	0,71	
12	7,43	88,49	1. Enantiomer 98.7 % ee
13	7,82	0,58	2. Enantiomer
14	9,32	0,11	
15	10,26	0,98	
16	10,45	0,83	
Total		100,00	

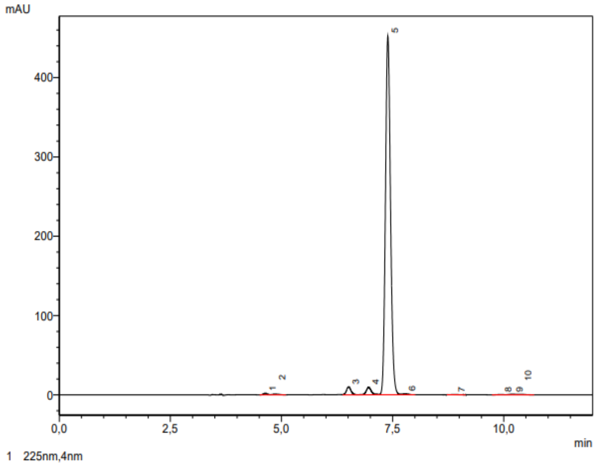

1 225nm,4nm

PDA Ch1 225nm

Peak #	Ret. Time	Area %	Name
1	4,64	0,29	
2	4,85	0,23	
3	6,51	1,87	
4	6,96	2,08	
5	7,39	94,56	1. Enantiomer > 99 % ee
6	7,78	0,33	2. Enantiomer
7	8,89	0,10	
8	9,94	0,10	
9	10,19	0,25	
10	10,39	0,17	
Total		100,00	

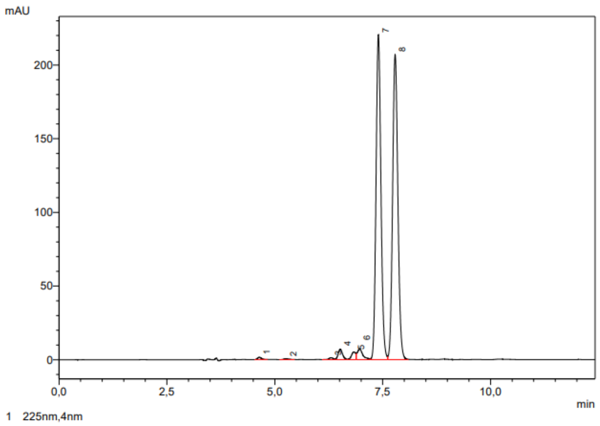

PDA Ch1 225nm

Peak #	Ret. Time	Area %	Name
1	4,64	0,26	
2	5,26	0,16	
3	6,31	0,25	
4	6,52	1,37	
5	6,83	0,97	
6	6,97	1,77	
7	7,40	47,51	
8	7,79	47,72	
Total		100,00	

Figure S15. HPLC traces of compound **20e**: with complex **7b**; procedure **A** (top left); with procedure **B** (top right); the corresponding racemate (bottom).

2,2,2-Trichloroethyl (*R*)-2-(4-cyanophenyl)-3-(cyclopentyloxy)propanoate (20f). Prepared according to

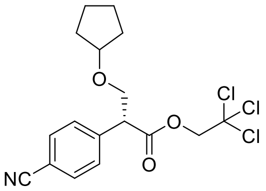

the general procedure **A** in pentane/CH_2Cl_2 as a colorless liquid; with complex **7b**: 57% yield, 99% ee; general procedure **B** with complex **7b**: 60% yield, 99% ee. [The ee was determined by HPLC analysis: Daicel 150 mm Chiralpak OJ-3R, Ø 4.6 mm, methanol/water = 85/15, v = 0.5 mL/min, λ = 230 nm, t(major) = 16.91 min, t(minor) = 18.61 min]. $[\alpha]_D^{20}$ = –1.2 (c = 0.52, $CHCl_3$); ^1H NMR (400 MHz, $CDCl_3$): δ = 7.67 – 7.59 (m, 2H), 7.54 – 7.45 (m, 2H), 4.76 (s, 2H), 4.07 – 3.94 (m, 2H), 3.94 – 3.86 (m, 1H), 3.71 (dd, *J* = 8.8, 5.4 Hz, 1H), 1.72 – 1.56 (m, 6H), 1.53 – 1.41 (m, 2H); ^{13}C NMR (101 MHz, $CDCl_3$): δ = 170.0, 140.7, 132.5, 129.5, 118.7, 112.0, 94.7, 82.3, 74.4, 69.5, 52.4, 32.23, 32.18, 23.6; IR (ATR): ṽ = 2957, 2870, 2230, 1753, 1505, 1347, 1143, 1096, 839, 801, 778, 719, 563 cm^{-1}; HRMS (ESI$^+$) for $C_{17}H_{18}Cl_3NO_3Na$ $[M+Na]^+$: calcd: 412.02445, found: 412.02463.

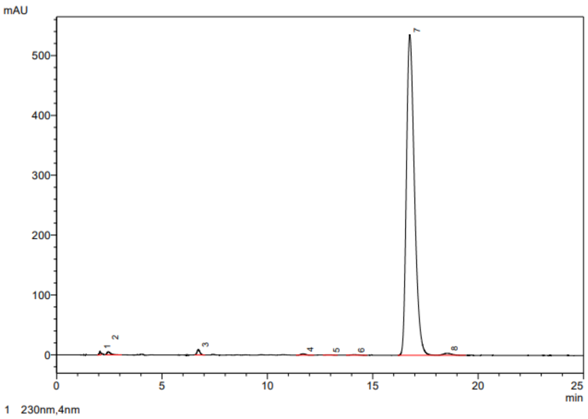

PDA Ch1 230nm

Peak #	Ret. Time	Area %	Name
1	2,06	0,20	
2	2,45	0,45	
3	6,72	0,61	
4	11,70	0,25	
5	12,94	0,10	
6	14,12	0,16	
7	16,76	97,54	1. Enantiomer 98.6 % ee
8	18,54	0,70	2. Enantiomer
Total		100,00	

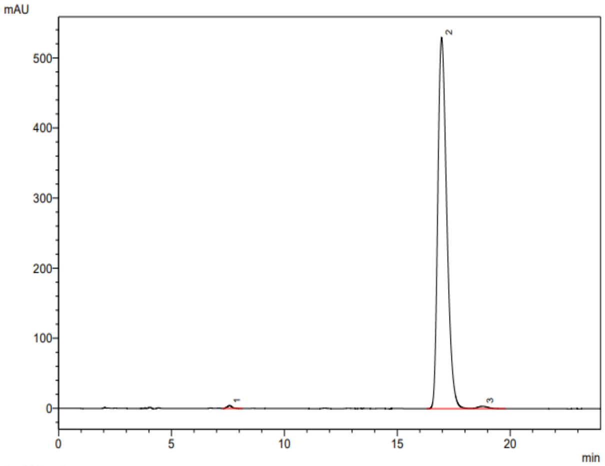

PDA Ch1 230nm

Peak #	Ret. Time	Area %	Name
1	7,58	0,44	
2	16,97	98,81	1. Enantiomer
3	18,78	0,75	2. Enantiomer 98.5 % ee
Total		100,00	

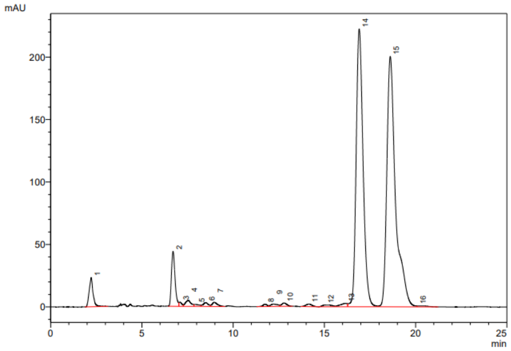

PDA Ch1 230nm

Peak #	Ret. Time	Area %	Name
1	2,22	2,46	
2	6,71	4,32	
3	7,09	0,26	
4	7,54	0,72	
5	7,95	0,21	
6	8,49	0,41	
7	8,97	0,47	
8	11,75	0,21	
9	12,21	0,36	
10	12,79	0,37	
11	14,15	0,35	
12	15,03	0,37	
13	16,15	0,51	
14	16,91	41,51	1. Enantiomer
15	18,61	47,29	2. Enantiomer + impurity
16	20,08	0,20	
Total		100,00	

Figure S16. HPLC traces of compound **20f**: with complex **7b;** procedure **A** (top left); with procedure **B** (top right); the corresponding racemate (bottom).

2,2,2-Trichloroethyl (*R*)-3-(cyclopentyloxy)-2-(4-(methylsulfonyl)phenyl)propanoate (20g). Prepared according to the general procedure **B** as a colorless liquid; with complex **7b**: 54% yield, >99% ee. [The ee was determined by HPLC analysis: Daicel 150 mm Chiralcel OJ-3R, Ø 4.6 mm, methanol/water = 95/5, v = 1.0 mL/min, λ = 220 nm, t(major) = 6.19 min, t(minor) = 7.34 min]. $[\alpha]_D^{20}$ = -1.3 (c = 1.00, $CHCl_3$); ^1H NMR (400 MHz, $CDCl_3$): δ = 7.96 – 7.86 (m, 2H), 7.63 – 7.54 (m, 2H), 4.78 (d, *J* = 12.0, Hz, 1H), 4.74 (d, *J* = 12.0, Hz, 1H), 4.08 (dd, *J* = 8.2, 5.5 Hz, 1H), 3.99 –3.94 (m, 1H), 3.94 –3.90 (m, 1H), 3.74 (dd, *J* = 9.0, 5.5 Hz, 1H), 3.04 (s, 3H), 1.73 – 1.41 (m, 8H); ^{13}C NMR (101 MHz, $CDCl_3$): δ = 170.0, 141.7, 140.2, 129.7, 127.8, 94.7, 82.3, 74.4, 69.6, 52.3, 44.6, 32.25, 32.18, 23.6; IR (ATR): ṽ = 2957, 2870, 1752, 1599, 1306, 1090, 956, 760, 717, 533 cm^{-1}; HRMS (ESI^+) for $C_{17}H_{21}Cl_3O_5SNa$ $[M+Na]^+$: calcd: 465.0068, found: 465.0067.

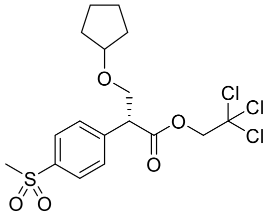

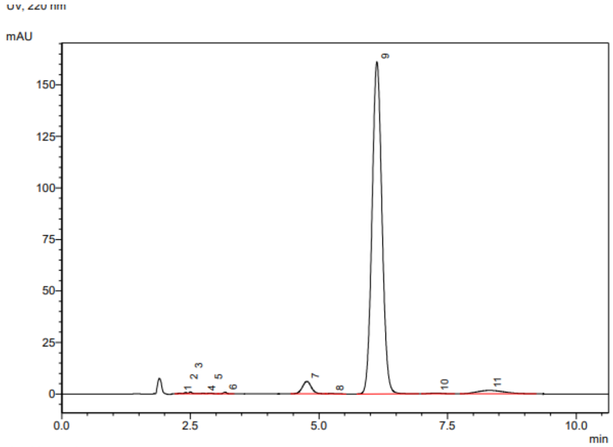

1 220nm,4nm

PDA Ch1 220nm

Peak #	Ret. Time	Area %	Name
1	2.29	0.07	
2	2.41	0.12	
3	2.50	0.19	
4	2.75	0.12	
5	2.88	0.13	
6	3.17	0.17	
7	4.77	3.19	
8	5.24	0.09	
9	6.13	92.95	1. enantiomer
10	7.28	0.27	2. enantiomer
11	8.31	2.69	
Total		100.00	

ee = 99.4%

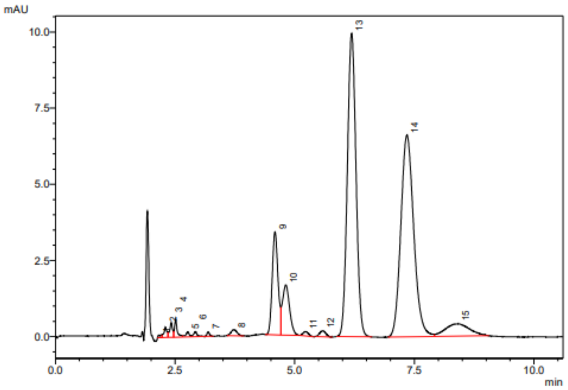

1 220nm,4nm

PDA Ch1 220nm

Peak #	Ret. Time	Area %	Name
1	2.16	0.12	
2	2.30	0.46	
3	2.42	0.64	
4	2.51	0.70	
5	2.76	0.26	
6	2.93	0.26	
7	3.19	0.16	
8	3.73	0.54	
9	4.59	8.89	
10	4.81	5.28	
11	5.23	0.35	
12	5.59	0.53	
13	6.19	38.84	1. enantiomer
14	7.34	38.55	2. enantiomer
15	8.41	4.42	
Total		100.00	

Figure S17. HPLC traces of compound **20g**: with complex **7b** (left); the corresponding racemate (right).

2,2,2-Trichloroethyl (*R*)-3-(cyclopentyloxy)-2-(4-(4,4,5,5-tetramethyl-1,3,2-dioxaborolan-2-yl)phenyl) propanoate (20h).

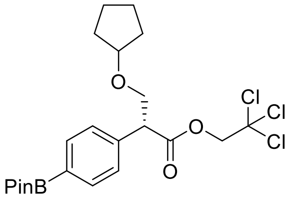

Prepared according to the general procedure **B** as a yellow liquid; with complex **7b**: 58% yield, >99% ee. [The ee was determined by HPLC analysis: Daicel 150 mm Chiralcel OJ-3R, Ø 4.6 mm, acetonitrile/water = 80/20, v = 0.5 mL/min, λ = 230 nm, t(major) = 8.40 min, t(minor) = 13.83 min]. $[\alpha]_D^{20}$ = 13.2 (c = 0.62, $CHCl_3$); ^{1}H NMR (400 MHz, $CDCl_3$): 7.77 (d, *J* = 8.1 Hz, 2H), 7.36 (d, *J* = 8.1 Hz, 2H), 4.77 (d, *J* = 12.0 Hz, 1H), 4.71 (d, *J* = 12.0 Hz, 1H), 4.06 – 3.95 (m, 2H), 3.95 – 3.84 (m, 1H), 3.69 – 3.57 (m, 1H), 1.76 – 1.56 (m, 6H), 1.52 – 1.46 (m, 2H), 1.34 (s, 12H); ^{13}C NMR (101 MHz, $CDCl_3$): δ = 170.9, 138.2, 135.3, 127.8, 95.0, 84.0, 82.1, 74.3, 70.2, 52.7, 32.3, 32.2, 25.0, 23.6; IR (ATR): ṽ = 2958, 2866, 1755, 1716, 1612, 1398, 1359, 1324, 1271, 1140, 1089, 1021, 858, 800, 719, 657, 573 cm^{-1}; HRMS (ESI^+) for $C_{22}H_{30}BCl_3O_5Na$ $[M+Na]^+$: calcd: 513.11441, found: 513.11474.

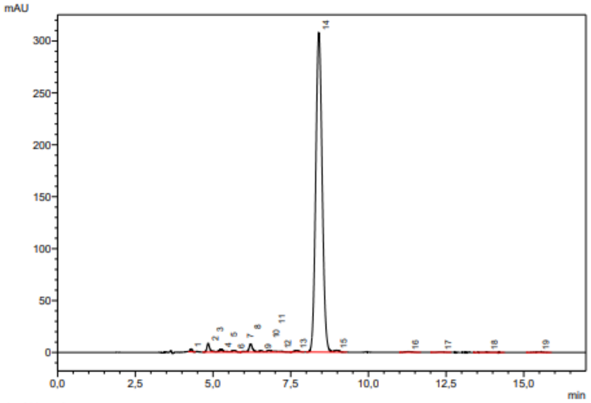

1 230nm,4nm

PDA Ch1 230nm

Peak #	Ret. Time	Area %	Name
1	4,29	0,28	
2	4,85	1,09	
3	5,00	0,14	
4	5,27	0,54	
5	5,45	0,11	
6	5,68	0,36	
7	5,98	0,15	
8	6,21	1,32	
9	6,54	0,27	
10	6,81	0,37	
11	6,99	0,19	
12	7,19	0,19	
13	7,69	0,34	
14	8,41	93,54	1. Enantiomer > 99 % ee
15	8,98	0,46	
16	11,29	0,27	
17	12,33	0,13	
18	13,83	0,11	2. Enantiomer
19	15,48	0,16	

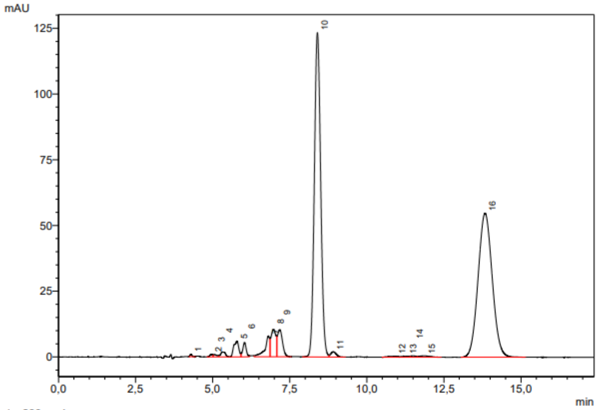

1 230nm,4nm

PDA Ch1 230nm

Peak #	Ret. Time	Area %	Name
1	4,29	0,10	
2	4,96	0,16	
3	5,06	0,18	
4	5,31	0,51	
5	5,79	1,69	
6	6,03	1,05	
7	6,80	2,16	
8	6,97	2,85	
9	7,18	2,90	
10	8,40	43,59	
11	8,91	0,61	
12	10,91	0,22	
13	11,27	0,17	
14	11,49	0,16	
15	11,87	0,32	
16	13,83	43,33	
Total		100,00	

Figure S18. HPLC traces of compound **20h**: with complex **7b** (left); the corresponding racemate (right).

2,2,2-Trichloroethyl (*R*)-3-(cyclopentyloxy)-2-(3-methoxyphenyl)propanoate (21a). Prepared according to the general procedure **A** as a colorless liquid; with complex **7b**: 64% yield, 93% ee; general procedure **B** as a colorless liquid; with complex **7b**: 49% yield, 97% ee. [The ee was determined by HPLC analysis: Daicel 150 mm Chiralpak OJ-3R, Ø 4.6 mm, methanol/water = 80/20, v = 1.0 mL/min, λ = 220 nm, t(major) = 21.55 min, t(minor) = 24.41 min]. $[\alpha]_D^{20}$ = 4.4 (c = 1.05, $CHCl_3$); ^1H NMR (400 MHz, $CDCl_3$): δ = 7.28 – 7.20 (m, 1H), 6.97 – 6.89 (m, 2H), 6.83 (ddd, *J* = 8.2, 2.5, 1.0 Hz, 1H), 4.79 (d, *J* = 12.0 Hz, 1H), 4.72 (d, *J* = 12.0 Hz, 1H), 4.06 – 3.90 (m, 3H), 3.80 (s, 3H), 3.64 (dd, *J* = 8.2, 3.9 Hz, 1H), 1.81 – 1.54 (m, 6H), 1.49 (m, 2H); ^{13}C NMR (101 MHz, $CDCl_3$): δ = 171.1, 159.9, 136.6, 129.8, 120.8, 114.1, 113.5, 95.0, 82.1, 74.3, 70.3, 55.4, 52.5, 32.3, 32.2, 23.7; IR (ATR): ṽ = 2955, 2869, 1753, 1600, 1585, 1490, 1446, 1345, 1261, 1138, 1094, 1042, 855, 793, 714, 571 cm^{-1}; HRMS (ESI^+) for $C_{17}H_{21}Cl_3O_4Na$ $[M+Na]^+$: calcd: 417.03976, found: 417.03927.

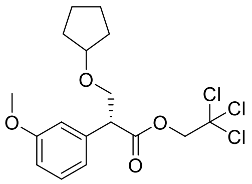

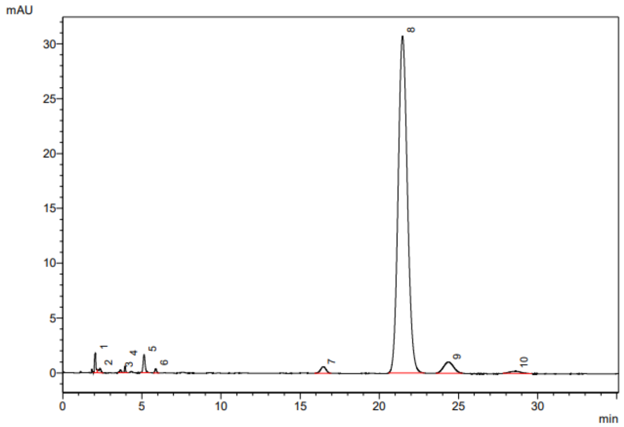

1 220nm,4nm

PDA Ch1 220nm

Peak #	Ret. Time	Area %	Name
1	2.07	0.85	
2	2.35	0.35	
3	3.65	0.15	
4	3.94	0.22	
5	5.14	1.07	
6	5.87	0.21	
7	16.45	1.07	
8	21.47	91.99	1st enantiomer
9	24.36	3.28	2nd enantiomer
10	28.59	0.82	
Total		100.00	

ee = 93.1%

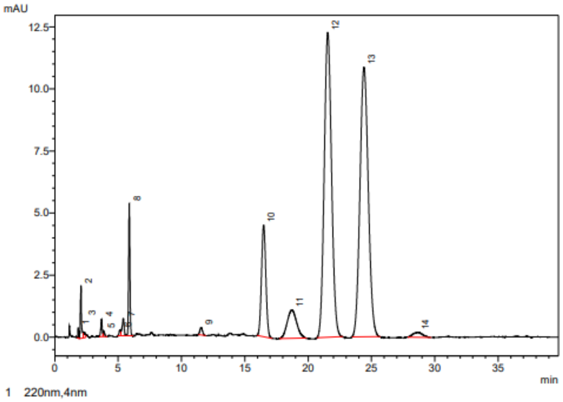

1 220nm,4nm

PDA Ch1 220nm

Peak #	Ret. Time	Area %	Name
1	1.84	0.13	
2	2.07	1.08	
3	2.34	0.17	
4	3.69	0.43	
5	3.86	0.13	
6	5.15	0.17	
7	5.41	0.62	
8	5.88	3.14	
9	11.56	0.34	
10	16.49	8.67	
11	18.71	4.80	
12	21.55	39.61	1st enantiomer
13	24.41	39.86	2nd enantiomer
14	28.65	0.83	
Total		100.00	

Additional Info : Peak(s) manually integrated

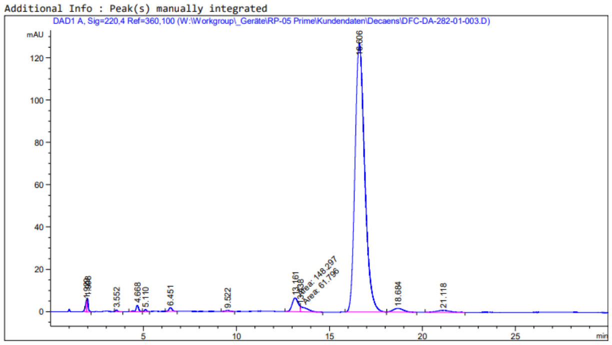

Additional Info : Peak(s) manually integrated

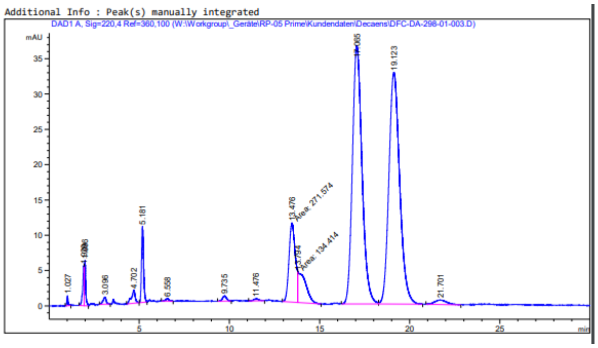

Peak #	RetTime [min]	Type	Width [min]	Area [mAU*s]	Height [mAU]	Area %	
1	1.929	VV	0.0588	23.00724	5.38780	0.4553	
2	1.998	VB	0.0726	34.47395	6.32086	0.6822	
3	3.552	BB	0.0859	5.11841	8.53781e-1	0.1013	
4	4.668	VV R	0.1423	31.73808	3.05966	0.6280	
5	5.110	VB	0.1203	8.21713	1.04981	0.1626	
6	6.451	BB	0.1754	21.14618	1.78316	0.4184	
7	9.522	BB	0.1941	8.09943	5.02313e-1	0.1603	
8	13.161	MF	0.3790	148.29694	6.52074	2.9345	
9	13.438	FM	0.3891	61.79604	2.64680	1.2228	
10	16.606	BB	0.5600	4601.81689	126.92947	91.0603	1st enantiomer
11	18.684	BB	0.4504	65.54720	1.72867	1.2970	2nd enantiomer
12	21.118	BB	0.5545	44.33377	9.39573e-1	0.8773	
Totals :				5053.59126	157.72264		

ee = 97.2 %

Peak #	RetTime [min]	Type	Width [min]	Area [mAU*s]	Height [mAU]	Area %	
1	1.027	BB	0.0561	6.11837	1.42404	0.1816	
2	1.929	BV	0.0712	29.14336	5.64860	0.8651	
3	1.996	VB	0.0736	34.89260	6.40390	1.0357	
4	3.096	BB	0.1452	12.40517	1.02508	0.3682	
5	4.702	BB	0.1843	27.04014	1.84124	0.8026	
6	5.181	BB	0.1228	86.31013	10.72962	2.5619	
7	6.558	BB	0.1645	4.99411	3.65408e-1	0.1482	
8	9.735	BB	0.1965	11.98189	7.37727e-1	0.3557	
9	11.476	BB	0.2458	5.93979	2.87748e-1	0.1763	
10	13.476	MF	0.4038	271.57382	11.21006	8.0611	
11	13.794	FM	0.5140	134.41391	4.35865	3.9898	
12	17.065	BV	0.5605	1351.14539	36.53251	40.1058	1st enantiomer
13	19.123	VB	0.6254	1362.60571	32.81698	40.4459	2nd enantiomer
14	21.701	BB	0.5674	30.39170	6.29387e-1	0.9021	
Totals :				3368.95610	114.01096		

Figure S19. HPLC traces of compound **21a**: following procedure **A** with complex **7b** (top left); the corresponding racemate (top right); following procedure **B** with complex **7b** (bottom left); the corresponding racemate (bottom right).

2,2,2-Trichloroethyl (*R*)-3-(cyclopentyloxy)-2-(3-fluorophenyl)propanoate (21b). Prepared according to the general procedure **A** as a colorless liquid; with complex **7b**: 71% yield, 99% ee. [The ee was determined by HPLC analysis: Daicel 150 mm Chiralpak AS-3R, Ø 4.6 mm, acetonitrile/H_2O = 50/50, v = 1.0 mL/min, λ = 220 nm, t(minor) = 22.65 min, t(major) = 25.02 min]. $[\alpha]_D^{20}$ = 15.0 (c = 0.5, $CHCl_3$); ^1H NMR (400 MHz, $CDCl_3$): 7.27 – 7.32 (m, 1H), 7.09 – 7.15 (m, 2H), 6.96 – 7.02 (m, 1H), 4.78 (d, *J* = 12 Hz, 1H), 4.73 (d, *J* = 12 Hz, 1H), 3.91 – 3.99 (m, 3H), 3.63 – 3.69 (m, 1H), 1.47 – 1.68 (m, 8H); ^{13}C NMR (101 MHz, $CDCl_3$): 170.6, 163.0 (d, *J* = 246.4 Hz), 137.6 (d, *J* = 7.7 Hz), 130.3 (d, *J* = 8.3 Hz), 124.3 (d, *J* = 3.0 Hz), 115.5 (d, *J* = 22.5 Hz), 115.0 (d, *J* = 21.0 Hz), 94.9, 82.2, 74.4, 70.0, 52.2 (d, *J* = 1.8 Hz), 32.3, 32.2, 23.6;^{19}F NMR (282 MHz, $CDCl_3$): δ = –112.5; IR (ATR): ṽ = 2957, 2870, 1754, 1614, 1591, 1488, 1449, 1373, 1346, 1263, 1136, 1096, 796, 715, 689, 574 cm^{-1}; HRMS (ESI^+) for $C_{16}H_{18}Cl_3FO_3Na$ $[M+Na]^+$: calcd: 405.01978, found: 405.01953.

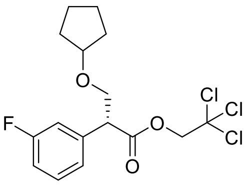

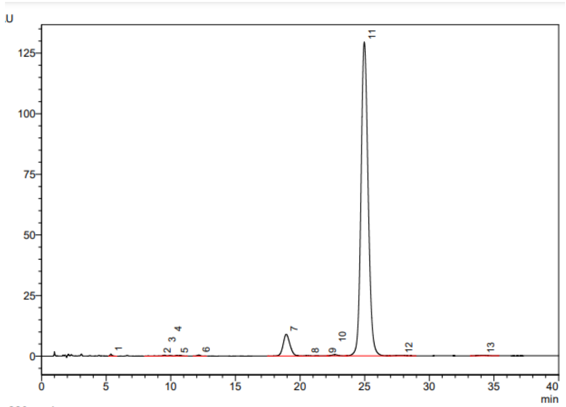

220nm,4nm

PDA Ch1 220nm

Peak #	Ret. Time	Area %	Name
1	5.37	0.14	
2	9.12	0.06	
3	9.51	0.10	
4	9.97	0.07	
5	10.46	0.10	
6	12.16	0.14	
7	18.93	6.10	
8	20.58	0.09	
9	21.91	0.02	
10	22.68	0.32	1st enantiomer
11	24.97	92.43	2nd enantiomer
12	27.81	0.19	
13	34.15	0.22	
Total		100.00	

ee = 99.3 %

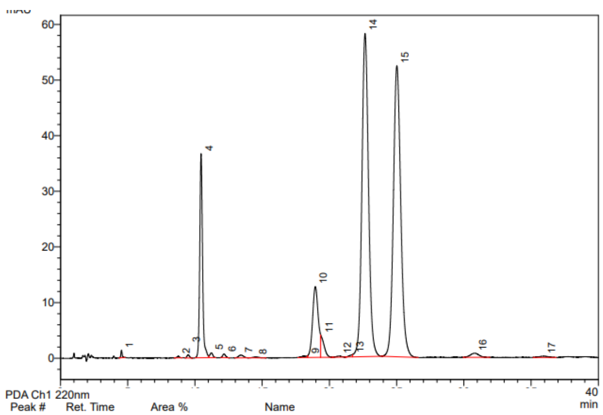

PDA Ch1 220nm

Peak #	Ret. Time	Area %	Name
1	4.54	0.17	
2	8.76	0.07	
3	9.50	0.14	
4	10.46	10.43	
5	11.22	0.25	
6	12.16	0.19	
7	13.42	0.25	
8	14.46	0.07	
9	18.37	0.15	
10	18.96	7.40	
11	19.38	1.36	
12	20.74	0.10	
13	21.68	0.12	
14	22.65	39.21	1st enantiomer
15	25.02	39.22	2nd enantiomer
16	30.81	0.65	
17	35.94	0.21	
Total		100.00	

Figure S20. HPLC traces of compound **21b**: with complex **7b** (left); the corresponding racemate (right).

2,2,2-Trichloroethyl (*R*)-3-(cyclopentyloxy)-2-(thiophen-3-yl)propanoate (22). Prepared according to the general procedure **A** as a colorless liquid; with complex **7b**: 51% yield, 98% ee. [The ee was determined by HPLC analysis: Daicel 150 mm Chiralpak OJ-3, Ø 4.6 mm, n-heptane/iso-propanol = 98/2, v = 1.0 mL/min, λ = 235 nm, t(major) = 3.60 min, t(minor) = 4.04 min]. $[\alpha]_D^{20}$ = −7.3 (c = 0.85, $CHCl_3$); ^1H NMR (400 MHz, $CDCl_3$): δ = 7.29 (dd, *J* = 5.0, 3.0 Hz, 1H), 7.24 (ddd, *J* = 3.0, 1.4, 0.6 Hz, 1H), 7.10 (dd, *J* = 5.0, 1.4 Hz, 1H), 4.82 – 4.71 (m, 2H), 4.14 (dd, *J* = 9.6, 4.9 Hz, 1H), 4.00 – 3.94 (m, 2H), 3.67 (dd, *J* = 9.0, 4.9 Hz, 1H), 1.73 – 1.59 (m, 6H), 1.53 – 1.47 (m, 2H); ^{13}C NMR (101 MHz, $CDCl_3$): δ = 170.8, 135.1, 127.6, 126.0, 123.0, 95.0, 82.1, 74.4, 70.0, 48.1, 32.4, 32.2, 23.7; IR (ATR): ṽ = 2955, 2869, 1753, 1448, 1204, 1136, 1095, 1045, 849, 791, 720, 570 cm^{-1}; HRMS (ESI^+) for $C_{14}H_{17}Cl_3O_3SNa$ $[M+Na]^+$: calcd: 392.98562, found: 392.98539.

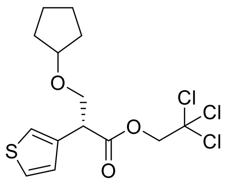

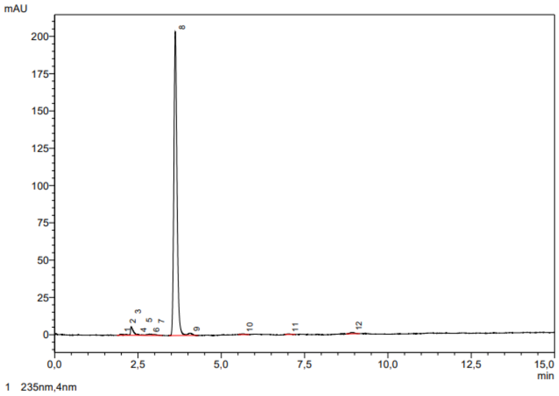

PDA Ch1 235nm

Peak #	Ret. Time	Area %	Name
1	1,99	0,29	
2	2,14	0,28	
3	2,30	2,35	
4	2,47	0,25	
5	2,63	0,17	
6	2,85	0,68	
7	3,00	0,32	
8	3,62	93,49	1. Enantiomer 97.8 % ee
9	4,06	1,05	2. Enantiomer
10	5,65	0,29	
11	7,02	0,22	
12	8,93	0,59	
Total		100,00	

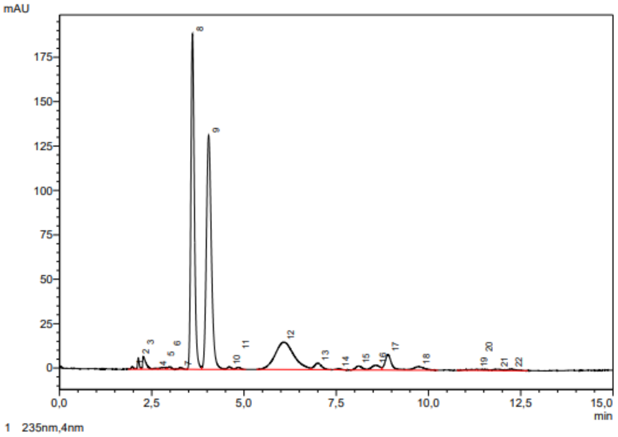

PDA Ch1 235nm

Peak #	Ret. Time	Area %	Name
1	1,98	0,21	
2	2,14	0,59	
3	2,27	1,33	
4	2,60	0,12	
5	2,82	0,36	
6	2,99	0,29	
7	3,28	0,20	
8	3,60	35,48	1. Enantiomer
9	4,04	35,64	2. Enantiomer

Figure S21. HPLC traces of compound **22**: with complex **7b** (left); the corresponding racemate (right).

2,2,2-Trichloroethyl (*S*)-2-(4-fluorophenyl)-3-phenylpropanoate (23a). Prepared according to the general

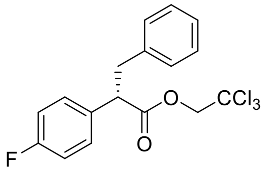

procedure **B** but using toluene as solvent and reagent; the title compound was obtained as a white solid; with complex **7b**: 80% yield, 95% ee. [The ee was determined by HPLC analysis: Daicel 150 mm Chiralpak IA-3, Ø 4.6 mm, n-heptane/i-propanol = 98/2, v = 1.0 mL/min, λ = 220 nm, t(minor) = 3.43 min, t(major) = 4.13 min]. $[\alpha]_D^{20}$ = 57.7 (c = 1.9, $CHCl_3$); ^1H NMR (400 MHz, $CDCl_3$): δ = 7.37 – 7.28 (m, 2H), 7.28 – 7.21 (m, 2H), 7.23 – 7.16 (m, 1H), 7.16 – 7.11 (m, 2H), 7.06 – 6.94 (m, 2H), 4.74 – 4.59 (m, 2H), 4.00 (dd, *J* = 8.7, 7.0 Hz, 1H), 3.46 (dd, *J* = 13.8, 8.7 Hz, 1H), 3.09 (dd, *J* = 13.8, 7.0 Hz, 1H); ^{13}C NMR (101 MHz, $CDCl_3$): δ = 171.7, 162.4 (d, *J* = 246.4 Hz), 138.3, 133.4 (d, *J* = 3.4 Hz), 129.9 (d, *J* = 8.1 Hz), 129.1, 128.6, 126.8, 115.7 (d, *J* = 21.6 Hz), 94.8, 74.2, 52.8, 39.6; ^{19}F NMR (282 MHz, $CDCl_3$): δ = −114.58; IR (ATR): ṽ = 1737, 1602, 1508, 1455 1377, 1222, 1204, 1175, 1147, 1077, 1046, 842, 794, 743, 718, 698, 569, 542, 522 cm^{-1}; HRMS (ESI^+) for $C_{17}H_{14}Cl_3FO_2Na$ $[M+Na]^+$: calcd: 396.99356, found: 396.99390.

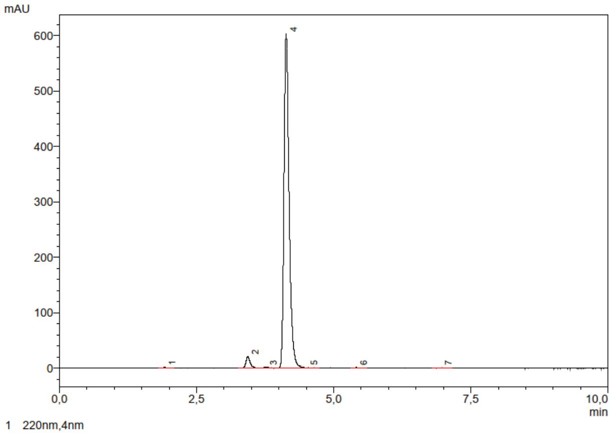

PDA Ch1 220nm

Peak #	Ret. Time	Area %	Name
1	1,92	0,14	
2	3,43	2,72	1. Enantiomer
3	3,77	0,24	
4	4,13	96,48	2. Enantiomer 94.5 % ee
5	4,51	0,15	
6	5,41	0,15	
7	6,95	0,11	
Total		100,00	

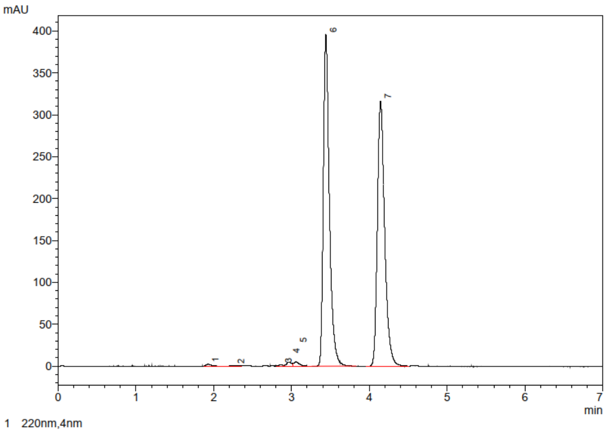

PDA Ch1 220nm

Peak #	Ret. Time	Area %	Name
1	1,93	0,28	
2	2,26	0,10	
3	2,87	0,14	
4	2,97	0,51	
5	3,06	0,64	
6	3,44	50,02	1. Enantiomer
7	4,14	48,30	2. Enantiomer
Total		100,00	

Figure S22. HPLC traces of compound **23a**: with complex **7b** (left); the corresponding racemate (right).

2,2,2-Trichloroethyl (*S*)-2-(4-fluorophenyl)-3-(4-isopropylphenyl)propanoate (23b). Prepared according

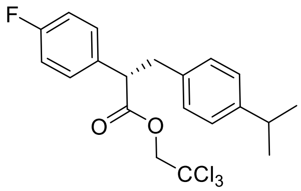

to the general procedure **A** as a white solid; with complex **7b**: 56% yield, 97% ee; following procedure **B:** with complex **7b**: 94% yield, 97% ee; with complex **7d**: 71% yield, 97% ee. [The ee was determined by HPLC analysis: Daicel 150 mm Chiralcel OJ-3R, Ø 4.6 mm, acetonitrile/water = 90/10, v = 0.5 mL/min, λ = 220 nm, t(minor) = 5.82 min, t(major) = 6.78 min]. $[\alpha]_D^{20}$ = 46.7 (c = 1.1, $CHCl_3$); ^1H NMR (400 MHz, $CDCl_3$): δ = 7.40 – 7.30 (m, 2H), 7.14 – 7.05 (m, 4H), 7.05 – 6.97 (m, 2H), 4.69 (d, *J* = 12.0 Hz, 1H), 4.61 (d, *J* = 12.0 Hz, 1H), 3.99 (dd, *J* = 9.2, 6.5 Hz, 1H), 3.42 (dd, *J* = 13.9, 9.2 Hz, 1H), 3.05 (dd, *J* = 13.9, 6.5 Hz, 1H), 2.85 (hept, *J* = 6. 9 Hz, 1H), 1.21 (d, *J* = 6.9 Hz, 6H); ^{13}C NMR (101 MHz, $CDCl_3$): δ = 171.8, 162.4 (d, *J* = 246.4 Hz), 147.4, 135.6, 133.6 (d, *J* = 3.3 Hz), 129.9 (d, *J* = 8.1 Hz), 129.0, 126.7, 115.7 (d, *J* = 21.2 Hz), 94.8, 74.2, 52.9, 39.3, 33.8, 24.1; ^{19}F NMR (282 MHz, $CDCl_3$): δ = −114.7; IR (ATR): ṽ = 2960, 1746, 1507, 1439, 1375, 1271, 1220, 1139, 1060, 842, 825, 799, 747, 719, 676, 578, 556, 522 cm^{-1}; HRMS (ESI^+) for $C_{20}H_{20}Cl_3FO_2Na$ $[M+Na]^+$: calcd: 439.04051, found: 439.04083.

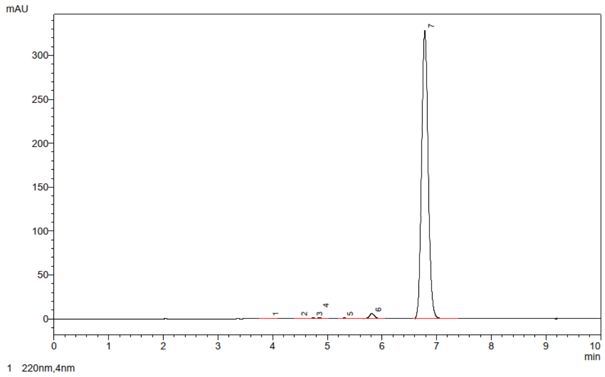

PDA Ch1 220nm

Peak #	Ret. Time	Area %	Name
1	3,94	0,12	
2	4,46	0,16	
3	4,74	0,22	
4	4,86	0,23	
5	5,30	0,33	
6	5,82	1,42	1. Enantiomer
7	6,78	97,52	2. Enantiomer 97.1 % ee
Total		100,00	

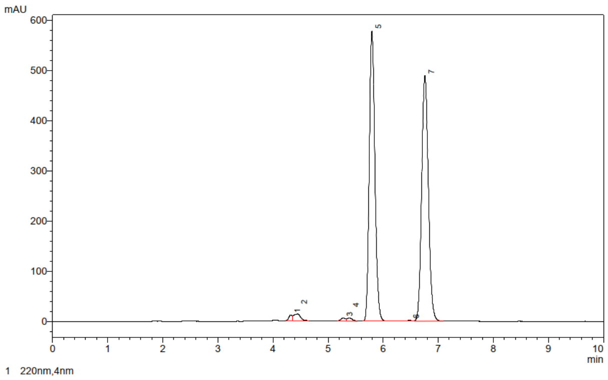

PDA Ch1 220nm

Peak #	Ret. Time	Area %	Name
1	4,32	0,60	
2	4,45	1,37	
3	5,27	0,42	
4	5,39	0,47	
5	5,80	48,32	1. Enantiomer
6	6,48	0,13	
7	6,76	48,69	2. Enantiomer
Total		100,00	

Figure S23. HPLC traces of compound **23b**: with complex **7b** (left); the corresponding racemate (right).

2,2,2-Trichloroethyl (*S,E*)-2-(4-fluorophenyl)oct-4-enoate (24). Prepared according to the general procedure **B** as a colorless liquid; with complex **7b**: 78% yield, 98% ee, 10:1 rr; with complex **7d**: 84% yield, 95% ee, 20:1 rr. [The ee was determined by HPLC analysis: Daicel 150 mm Chiralcel OJ-3R, Ø 4.6 mm, methanol/water = 85/15, v = 0.5 mL/min, λ = 220 nm, t(minor) = 23.02 min, t(major) = 24.08 min]. $[\alpha]_D^{20}$ = 25.8 (c = 2.8, $CHCl_3$); ^1H NMR (400 MHz, $CDCl_3$): δ = 7.39 – 7.24 (m, 2H), 7.08 – 6.93 (m, 2H), 5.50 (dtt, *J* = 14.8, 6.8, 1.3 Hz, 1H), 5.32 (dtt, *J* = 15.1, 6.8, 1.3 Hz, 1H), 4.79 – 4.61 (m, 2H), 3.73 (dd, *J* = 8.5, 7.1 Hz, 1H), 2.81 (dddq, *J* = 15.2, 8.1, 7.1, 1.0 Hz, 1H), 2.57 – 2.44 (m, 1H), 1.97 – 1.86 (m, 2H), 1.31 (h, *J* = 7.4 Hz, 2H), 0.82 (t, *J* = 7.4 Hz, 3H); ^{13}C NMR (101 MHz, $CDCl_3$): δ = 171.9, 162.3 (d, *J* = 246.1 Hz), 134.1, 133.6 (d, *J* = 3.3 Hz), 129.9 (d, *J* = 8.1 Hz), 125.9, 115.6 (d, *J* = 21.3 Hz), 94.9, 74.2, 51.3, 36.4, 34.7, 22.5, 13.7; ^{19}F NMR (282 MHz, $CDCl_3$): δ = −115.00; IR (ATR): ṽ = 2958, 752, 1605, 1509, 1438, 1226, 1137, 1124, 1043, 969, 837, 799, 716, 573, 519 cm^{-1}; HRMS (EI^+) for $C_{16}H_{18}Cl_3FO_2$ $[M]^+$: calcd: 366.03509, found: 366.03523.

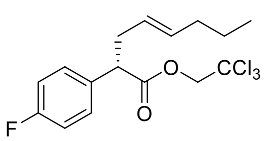

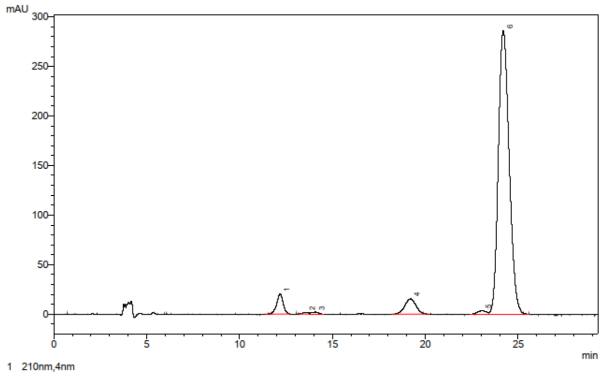

1 210nm,4nm

PDA Ch1 210nm

Peak #	Ret. Time	Area %	Name
1	12,19	3,92	
2	13,55	0,26	
3	14,07	0,43	
4	19,19	5,01	
5	23,04	0,94	1. Enantiomer
6	24,20	89,43	2. Enantiomer 97.9 % ee
Total		100,00	

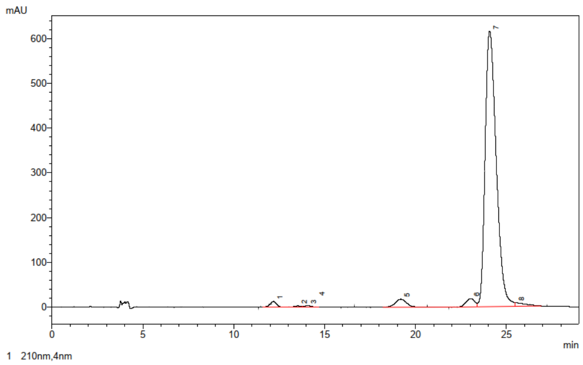

1 210nm,4nm

PDA Ch1 210nm

Peak #	Ret. Time	Area %	Name
1	12,19	1,17	
2	13,53	0,29	
3	14,04	0,36	
4	14,51	0,03	
5	19,18	2,80	
6	23,02	2,19	1. Enantiomer
7	24,08	92,00	2. Enantiomer 95.3 % ee
8	25,47	1,15	
Total		100,00	

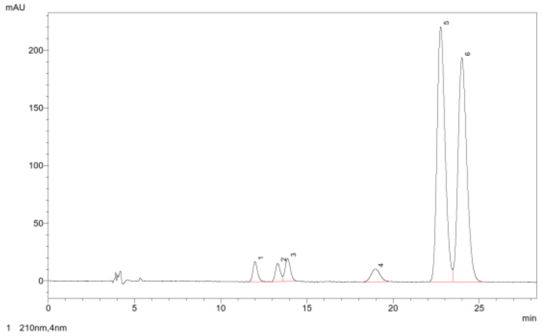

1 210nm,4nm

PDA Ch1 210nm

Peak #	Ret. Time	Area %	Name
1	11,98	2,09	
2	13,31	1,99	
3	13,88	2,75	
4	18,95	2,63	
5	22,75	46,27	1. Enantiomer
6	23,99	44,26	2. Enantiomer
Total		100,00	

Figure S24. HPLC traces of compound **24**: with complex **7b** (top, left); with complex **7d** (top, right); the corresponding racemate (bottom).

2,2,2-Trichloroethyl (*S*,*E*)-6-((*tert*-butyldimethylsilyl)oxy)-2-(4-fluorophenyl)hex-4-enoate (25). Prepared

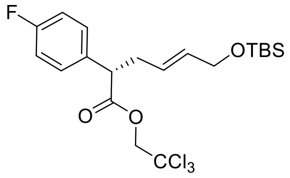

according to the general procedure **A** as a colorless oil; with catalyst **7b**: 59%, 96% ee; with catalyst **7d**: 62%, 99% ee. [The ee was determined by HPLC analysis: Daicel 150 mm Chiralcel OJ-3R, Ø 4.6 mm, MeCN/water = 65/35, v = 1.0 mL/min, λ = 210 nm, t(minor) = 18.14 min, t(major) = 19.14 min.] $[\alpha]_D^{20}$ = +13.2 (c = 0.9, $CHCl_3$); ^{1}H NMR (400 MHz, $CDCl_3$): δ = 7.37 – 7.27 (m, 2H), 7.07 – 6.97 (m, 2H), 5.68 – 5.50 (m, 2H), 4.70 (d, *J* = 1.3 Hz, 2H), 4.06 (dt, *J* = 4.7, 1.3 Hz, 2H), 3.78 – 3.73 (m, 1H), 2.92 – 2.80 (m, 1H), 2.62 – 2.50 (m, 1H), 0.87 (s, 9H), 0.02 (d, *J* = 0.6 Hz, 6H); ^{13}C NMR (101 MHz, $CDCl_3$): δ = 171.8, 162.4 (d, *J* = 246.0 Hz), 133.4 (d, *J* = 3.3 Hz), 132.7, 129.9 (d, *J* = 8.1 Hz), 126.3, 115.7 (d, *J* = 21.5 Hz), 94.9, 74.2, 63.5, 50.9, 35.8, 26.1, 18.5, −5.1; ^{19}F NMR (282 MHz, $CDCl_3$): δ = −114.9; IR (ATR): ṽ = 2954, 2930, 2857, 1752, 1696, 1605, 1510, 1254, 1227, 1136, 834, 807, 776, 717, 573, 518 cm^{-1}; HRMS (ESI$^+$) for $C_{20}H_{28}O_3FCl_3SiNa$ $[M+Na^+]^+$: calcd: 491.07496, found: 491.07535.

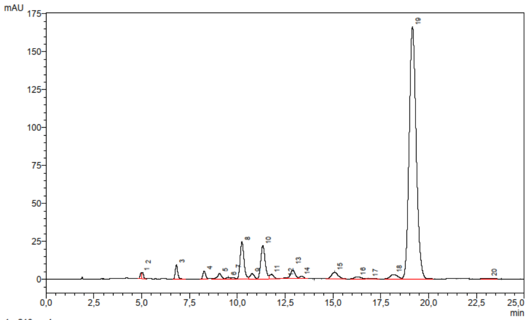

1 210nm,4nm

PDA Ch1 210nm

Peak #	Ret. Time	Area %	Name
1	4,97	0,39	
2	5,05	0,45	
3	6,81	1,45	
4	8,26	0,96	
5	9,07	1,02	
6	9,52	0,27	
7	9,77	0,23	
8	10,24	6,03	
9	10,77	0,94	
10	11,33	6,17	
11	11,78	0,83	
12	12,50	0,22	
13	12,90	1,74	
14	13,36	0,48	
15	15,08	1,84	
16	16,27	0,67	
17	16,91	0,19	
18	18,17	1,39	1. Enantiomer
19	19,15	74,49	2. Enantiomer 96.3 % ee
20	23,13	0,24	
Total		100,00	

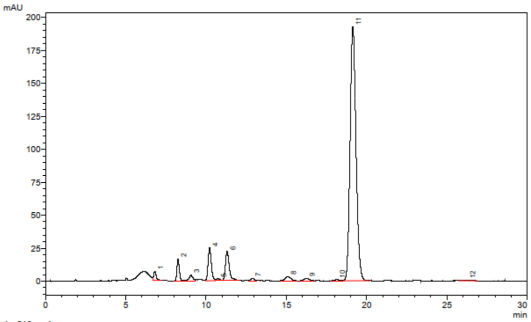

1 210nm,4nm

PDA Ch1 210nm

Peak #	Ret. Time	Area %	Name
1	6,81	1,27	
2	8,26	2,77	
3	9,06	1,19	
4	10,22	5,61	
5	10,75	0,37	
6	11,32	6,02	
7	12,89	0,50	
8	15,09	1,36	
9	16,25	0,70	
10	18,14	0,34	1. Enantiomer
11	19,14	79,71	2. Enantiomer 99.2 % ee
12	26,25	0,18	
Total		100,00	

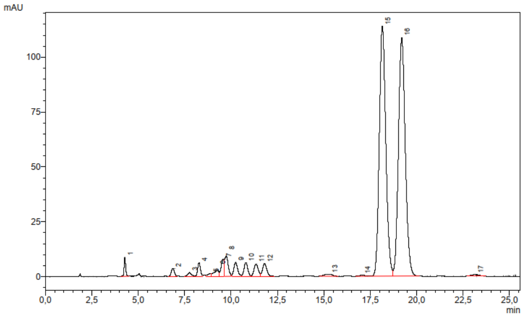

Figure S25. HPLC traces of compound **25**: with catalyst **7b** (top, left); with catalyst **7d** (top, right); the corresponding racemate (bottom).

2,2,2-Trichloroethyl (*S*,*E*)-2-(4-fluorophenyl)-5-(4-methoxyphenyl)pent-4-enoate (26). Prepared according to the general procedure **B** as a colorless oil; with catalyst **7b**: 71%, 98% ee. [The ee was determined by HPLC analysis: Daicel 150 mm Chiralcel OD-3, Ø 4.6 mm, *n*-heptane/*iso*-propanol = 99/1, v = 1.0 mL/min, λ = 220 nm, t(minor) = 6.84 min, t(major) = 7.88 min.] $[\alpha]_D^{20}$ = +42.3 (c = 1.1, $CHCl_3$); ^1H NMR (400 MHz, $CDCl_3$): δ = 7.40 – 7.29 (m, 2H), 7.24 – 7.18 (m, 2H), 7.08 – 6.97 (m, 2H), 6.85 – 6.78 (m, 2H), 6.41 (dt, *J* = 15.6, 1.4 Hz, 1H), 5.96 (ddd, *J* = 15.8, 7.6, 6.7 Hz, 1H), 4.78 – 4.64 (m, 2H), 3.87 – 3.80 (m, 1H), 3.79 (s, 3H), 3.01 (dddd, *J* = 14.3, 8.7, 7.5, 1.3 Hz, 1H), 2.76 – 2.64 (m, 1H); ^{13}C NMR (101 MHz, $CDCl_3$): δ = 171.8, 162.4 (d, *J* = 246.4 Hz), 159.2, 133.4 (d, *J* = 3.1 Hz), 132.4, 130.0, 129.9 (d, *J* = 8.1 Hz), 127.4, 123.8, 115.8 (d, *J* = 21.5 Hz), 114.1, 94.9, 74.3, 55.4, 51.2, 36.9; ^{19}F NMR (282 MHz, $CDCl_3$): δ = −114.7; IR (ATR): ṽ = 2935, 1749, 1606, 1508, 1245, 1174, 1160, 1133, 1034, 966, 837, 791, 759, 716, 572, 520, 436 cm^{-1}; HRMS (ESI^+) for $C_{20}H_{18}O_3FCl_3Na$ $[M+Na^+]^+$: calcd: 453.01978, found: 453.01974.

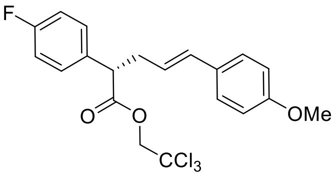

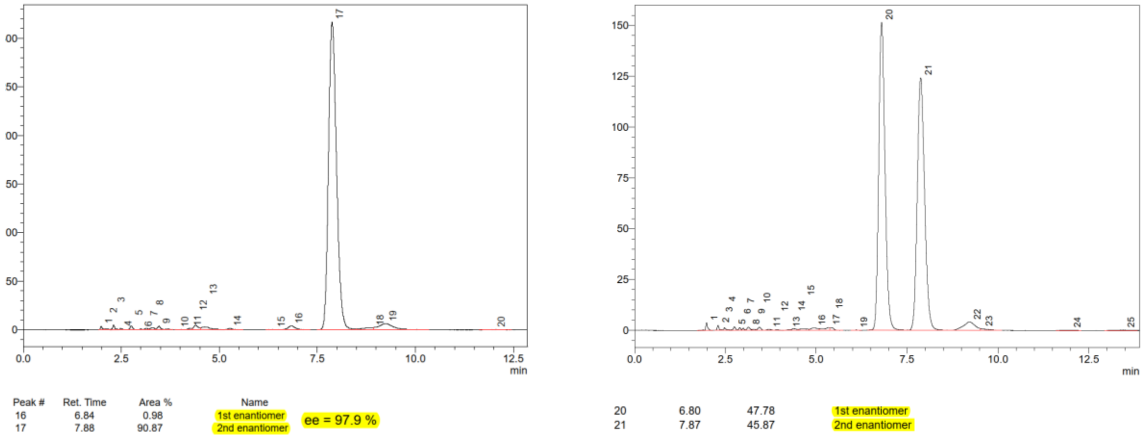

Figure S26. HPLC traces of compound **26**: with catalyst **7b** (left); the corresponding racemate (right).

2,2,2-Trichloroethyl (*S*,*E*)-5-bromo-2-(4-fluorophenyl)pent-4-enoate (27). Prepared according to the general procedure **B** as a colorless oil; with catalyst **7b**: 80%, 96% ee. [The ee was determined by HPLC analysis: Daicel 150 mm Chiralcel OD-3, Ø 4.6 mm, *n*-heptane/*iso*-propanol = 99/1, v = 1.0 mL/min, λ = 220 nm, t(minor) = 4.45 min, t(major) = 5.09 min.] $[\alpha]_D^{20}$ = +3.7 (c = 1.2, $CHCl_3$); ^1H NMR (400 MHz, $CDCl_3$): δ = 7.33 – 7.27 (m, 2H), 7.08 – 7.00 (m, 2H), 6.20 – 6.05 (m, 2H), 4.81 – 4.64 (m, 2H), 3.77 (dd, *J* = 8.5, 6.9 Hz, 1H), 2.93 – 2.80 (m, 1H), 2.60 – 2.49 (m, 1H); ^{13}C NMR (101 MHz, $CDCl_3$): δ = 171.2, 162.5 (d, *J* = 246.9 Hz), 133.7, 132.6 (d, *J* = 3.2 Hz), 129.8 (d, *J* = 8.1 Hz), 116.0 (d, *J* = 21.7 Hz), 108.0, 94.8, 74.3, 50.1, 36.5; ^{19}F NMR (282 MHz, $CDCl_3$): δ = -114.13; IR (ATR): ṽ = 2929, 1750, 1605, 1509, 1372, 1225, 1199, 1161, 1134, 1063, 932, 837, 794, 745, 715, 574, 555, 518, 409 cm^{-1}; HRMS (ESI^+) for $C_{13}H_{12}O_2BrFCl_3$ $[M+H]^+$: calcd: 402.90649, found: 402.90682.

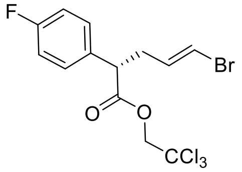

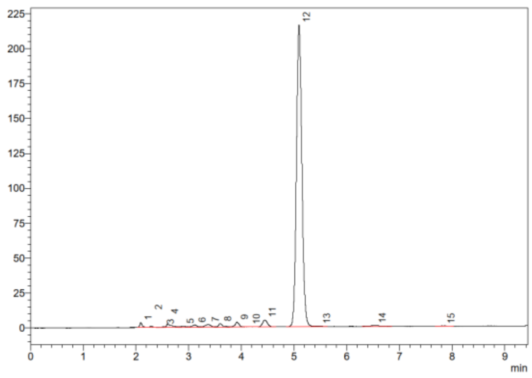

11 4.45 1.85 1st enantiomer
12 5.09 91.45 2nd enantiomer ee = 96.0 %

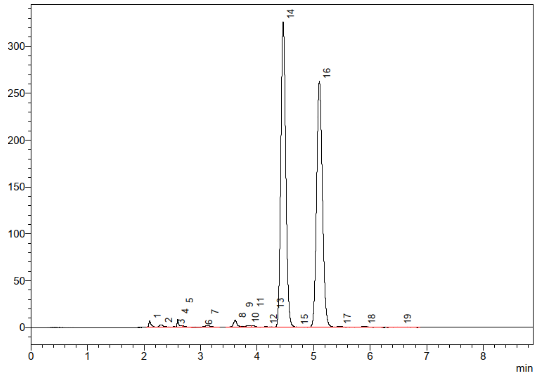

14 4.46 49.10 1st enantiomer

16 5.10 46.39 2nd enantiomer

Figure S27. HPLC traces of compound **27**: with catalyst **7b** (left); the corresponding racemate (right).

2,2,2-Trichloroethyl (*S*,*E*)-2-(4-fluorophenyl)-5-(4,4,5,5-tetramethyl-1,3,2-dioxaborolan-2-yl)pent-4-enoate (28).

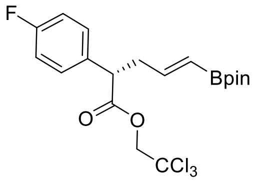

Prepared according to the general procedure **B** as a colorless oil; with catalyst **7b**: 55%, 97% ee. [The ee was determined by HPLC analysis: Daicel 150 mm Chiralcel ID-3, Ø 4.6 mm, MeCN/water = 50/50, v = 1.0 mL/min, λ = 220 nm, t(minor) = 14.91 min, t(major) = 16.22 min.] $[\alpha]_D^{20}$ = +28.7 (c = 0.9, $CHCl_3$); ^{1}H NMR (400 MHz, $CDCl_3$) δ 7.30 – 7.20 (m, 2H), 7.03 – 6.89 (m, 2H), 6.46 (dt, *J* = 18.0, 6.4 Hz, 1H), 5.46 (dt, *J* = 17.9, 1.5 Hz, 1H), 4.71 – 4.57 (m, 2H), 3.76 (dd, *J* = 8.9, 6.5 Hz, 1H), 2.93 (dddd, *J* = 15.4, 8.9, 6.6, 1.5 Hz, 1H), 2.58 (dtd, *J* = 14.8, 6.4, 1.6 Hz, 1H), 1.17 (s, 12H); ^{13}C NMR (101 MHz, $CDCl_3$): δ = 171.7, 162.4 (d, *J* = 246.0 Hz), 149.2, 133.3 (d, *J* = 3.4 Hz), 129.8 (d, *J* = 8.1 Hz), 115.8 (d, *J* = 21.6 Hz), 122.0, 94.8, 83.4, 74.2, 49.9, 39.1, 24.9 (2x); ^{11}B NMR (128 MHz, $CDCl_3$): δ = −14.6; ^{19}F NMR (282 MHz, $CDCl_3$): δ = −114.7; IR (ATR): ṽ = 2930, 1753, 1692, 1640, 1509, 1362, 1324, 1225, 1141, 1003, 972, 838, 803, 718, 574, 517, 441 cm^{-1}; HRMS (ESI$^+$) for $C_{19}H_{23}O_4BFCl_3Na$ [M+Na$^+$]$^+$: calcd: 473.06312, found: 473.06331.

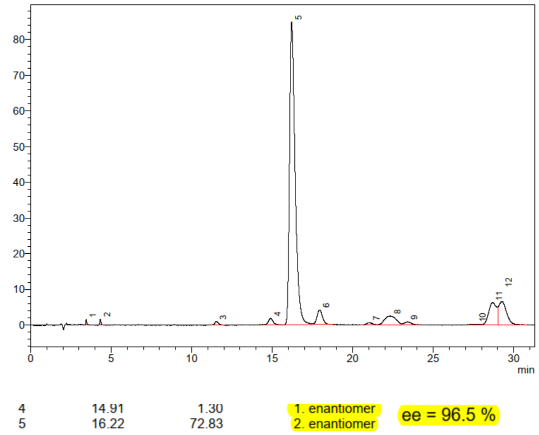

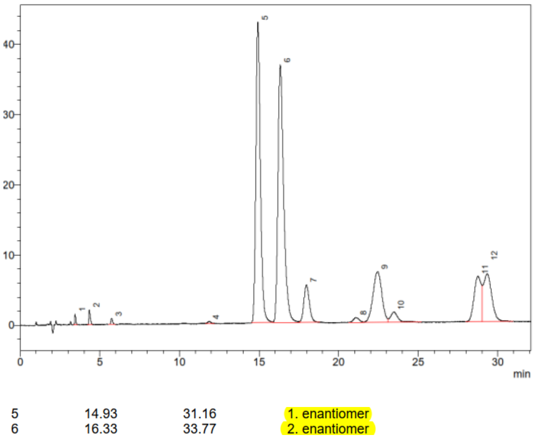

Figure S28. HPLC traces of compound **28**: with catalyst **7b** (left); the corresponding racemate (right).

1-Ethyl 6-(2,2,2-trichloroethyl) (*S*,*E*)-5-(4-fluorophenyl)hex-2-enedioate (29). Prepared according to the general procedure **B** as a colorless liquid; with complex **7b**: 50% yield, 94% ee. [The ee was determined by HPLC analysis: Daicel 150 mm Chiralpak IB-N-3, Ø 4.6 mm, n-heptane/i-propanol = 98/2, v = 1.0 mL/min, λ = 220 nm, t(major) = 3.09 min, t(minor) = 7.56 min]. $[\alpha]_D^{20}$ = 40.8 (c = 1.7, $CHCl_3$); ^{1}H NMR (400 MHz, $CDCl_3$): δ = 7.36 – 7.24 (m, 2H), 7.03 (t, *J* = 8.6 Hz, 2H), 6.84 (dt, *J* = 15.8, 7.03 Hz, 1H), 5.88 (dd, *J* = 15.6, 1.7 Hz, 1H), 4.78 – 4.65 (m, 2H), 4.16 (q, *J* = 7.1 Hz, 2H), 3.84 (t, *J* = 7.7 Hz, 1H), 3.10 – 2.97 (m, 1H), 2.71 (dtd, *J* = 15.3, 6.9, 1.6 Hz, 1H), 1.26 (t, *J* = 7.1 Hz, 3H); ^{13}C NMR (101 MHz, $CDCl_3$): δ = 171.2, 166.1, 162.5 (d, *J* = 247.0 Hz), 144.1, 132.6 (d, *J* = 3.1 Hz), 129.8 (d, *J* = 8.1 Hz), 124.2, 116.0 (d, *J* = 21.7 Hz), 94.7, 74.3, 60.5, 49.6, 35.5, 14.3; ^{19}F NMR (282 MHz, $CDCl_3$): δ = −114.07; IR (ATR): ṽ = 1751, 1716, 1657, 1509, 1369, 1265, 1224, 1192, 1137, 1037, 838, 805, 744, 716, 574, 518 cm^{-1}; HRMS (ESI$^+$) for $C_{16}H_{17}Cl_3FO_4$ [M+H]$^+$: calcd: 397.01710, found: 397.01742.

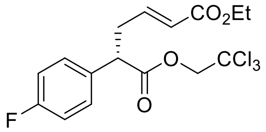

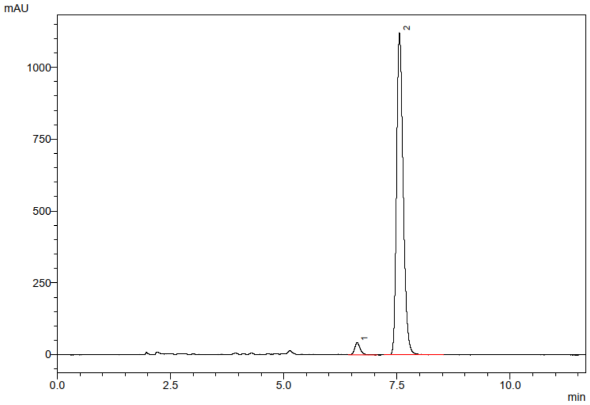

1 210nm,4nm

PDA Ch1 210nm

Peak #	Ret. Time	Area %	Name
1	6.62	3.09	1st enantiomer
2	7.56	96.91	2nd enantiomer, 94% ee
Total		100.00	

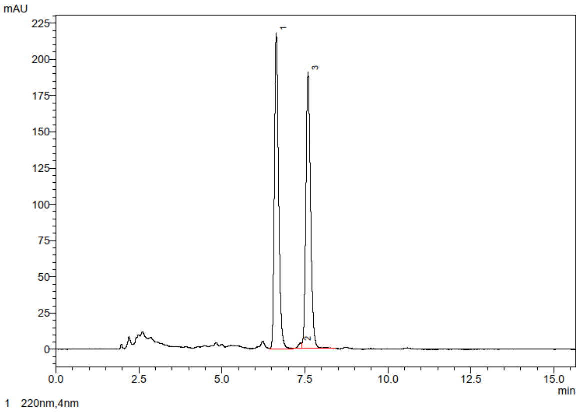

1 220nm,4nm

PDA Ch1 220nm

Peak #	Ret. Time	Area %	Name
1	6.64	50.31	1st enantiomer
2	7.37	0.69	
3	7.59	49.00	2nd enantiomer
Total		100.00	

Figure S29. HPLC traces of compound **29**: with complex **7b** (left); the corresponding racemate (right).

1-Methyl 8-(2,2,2-trichloroethyl) (*S*,2*E*,4*E*)-7-(4-fluorophenyl)octa-2,4-dienedioate (30). Prepared according to the general procedure **B** as a colorless liquid; with complex **7b**: 68% yield, >99% ee. [The ee was determined by HPLC analysis: Daicel 150 mm Chiralpak OJ-3R, Ø 4.6 mm, methanol/water = 90/10, v = 0.5 mL/min, λ = 254 nm, t(major) = 17.21 min, t(minor) = 19.14 min]. $[\alpha]_D^{20}$ = 68.3 (c = 0.46, $CHCl_3$); ^1H NMR (400 MHz, $CDCl_3$): δ = 7.39 (ddd, *J* = 15.4, 11.1, 0.8 Hz, 1H), 7.11 – 7.00 (m, 2H), 6.90 – 6.77 (m, 2H), 6.01 – 5.86 (m, 2H), 5.69 – 5.56 (m, 1H), 4.46 (d, *J* = 12.1 Hz, 1H), 4.37 (d, *J* = 12.1 Hz, 1H), 3.52 (s, 3H), 3.50 – 3.45 (m, 1H), 2.74 (dtd, *J* = 14.6, 8.1, 1.1 Hz, 1H), 2.35 (dtd, *J* = 14.4, 7.1, 1.2 Hz, 1H); ^{13}C NMR (101 MHz, $CDCl_3$): δ = 171.3, 167.4, 162.4 (d, *J* = 246.9 Hz), 144.2, 138.9, 132.7 (d, *J* = 3.5 Hz), 131.0, 129.7 (d, *J* = 8.1 Hz), 120.4, 115.8 (d, *J* = 6.4 Hz), 94.6, 74.1, 51.6, 50.2, 36.3; ^{19}F NMR (282 MHz, $CDCl_3$): δ = –114.2; IR (ATR): $\tilde{\nu}$ = 2995, 1723, 1604, 1509, 1437, 1224, 1143, 1035, 980, 839, 805, 716, 572, 518, 425 cm^{-1}; HRMS (ESI^+) for $C_{17}H_{16}Cl_3FO_4Na$ $[M+Na]^+$: calcd: 430.99904, found: 430.99875.

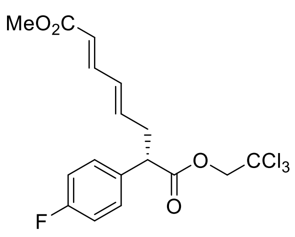

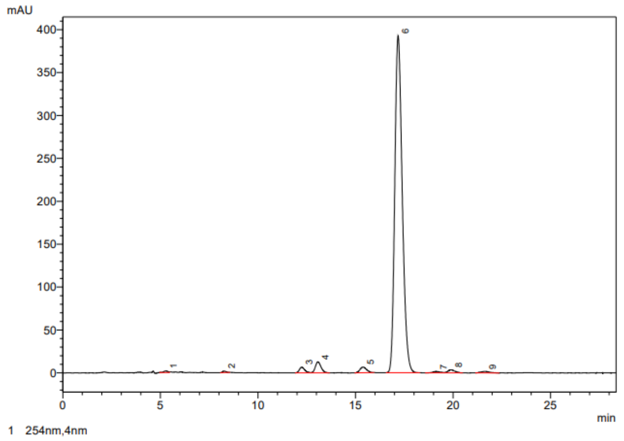

PDA Ch1 254nm

Peak #	Ret. Time	Area %	Name
1	5,28	0,26	
2	8,26	0,19	
3	12,24	1,11	
4	13,08	2,28	
5	15,39	1,40	
6	17,18	93,10	1. Enantiomer > 99 % ee
7	19,14	0,38	2. Enantiomer
8	19,90	0,79	
9	21,65	0,49	
Total		100,00	

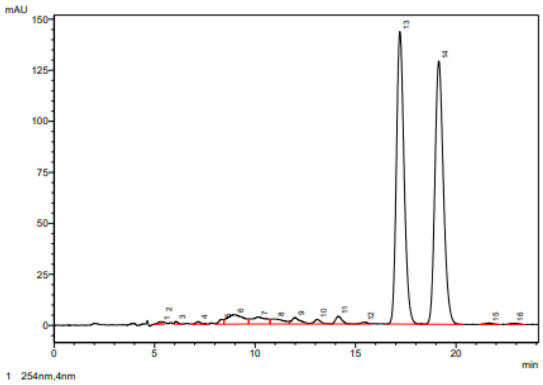

PDA Ch1 254nm

Peak #	Ret. Time	Area %	Name
1	5,28	0,16	
2	5,40	0,13	
3	6,07	0,19	
4	7,17	0,23	
5	8,33	0,45	
6	8,96	3,04	
7	10,17	2,08	
8	10,97	1,33	
9	11,98	1,11	
10	13,10	0,61	
11	14,14	0,97	
12	15,44	0,23	
13	17,21	44,56	1. Enantiomer
14	19,14	44,48	2. Enantiomer
15	21,65	0,26	
16	22,87	0,17	
Total		100,00	

Figure S30. HPLC traces of compound **30**: with complex **7b** (left); the corresponding racemate (right).

2,2,2-Trichloroethyl (*S*)-2-(4-fluorophenyl)-5-(trimethylsilyl)pent-4-ynoate (31a). Prepared according to

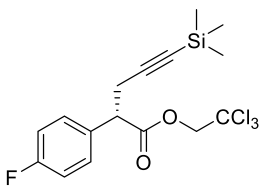

the general procedure **B** as a colorless liquid; with complex **7b**: <51% yield (the product contained compound **11** as inseparable impurity, ca. 25 %), 95% ee. [The ee was determined by HPLC analysis: Daicel 150 mm Chiralpak IG-G, Ø 4.6 mm, acetonitrile/water = 60/40, v = 1.0 mL/min, λ = 220 nm, t(minor) = 8.79 min, t(major) = 10.75 min]. $[\alpha]_D^{20}$ = 26.0 (c = 1.10, $CHCl_3$); ^1H NMR (400 MHz, $CDCl_3$): δ = 7.36 – 7.27 (m, 2H), 7.05 – 6.98 (m, 2H), 4.78 (d, *J* = 11.9 Hz, 1H), 4.71 (d, *J* = 11.9 Hz, 1H), 3.93 (t, *J* = 7.7 Hz, 1H), 2.99 (dd, *J* = 16.9, 8.0 Hz, 1H), 2.72 (dd, *J* = 16.9, 7.4 Hz, 1H), 0.08 (s, 9H); ^{13}C NMR (101 MHz, $CDCl_3$): δ = 170.9, 162.6 (d, *J* = 246.5 Hz), 132.5 (d, *J* = 3.4 Hz), 129.8 (d, *J* = 8.1 Hz), 115.7 (d, *J* = 21.5 Hz), 94.7, 87.5, 81.5, 74.3, 50.2, 24.5, 0.1; ^{19}F NMR (282 MHz, $CDCl_3$): δ = –114.3; IR (ATR): ṽ = 2959, 2179, 1754, 1605, 1509, 1422, 1250, 1226, 1137, 1030, 837, 759, 717, 574, 516 cm^{-1}; HRMS (ESI^+) for $C_{16}H_{18}Cl_3FO_2SiNa$ $[M+Na]^+$: calcd: 417.00179, found: 417.00140.

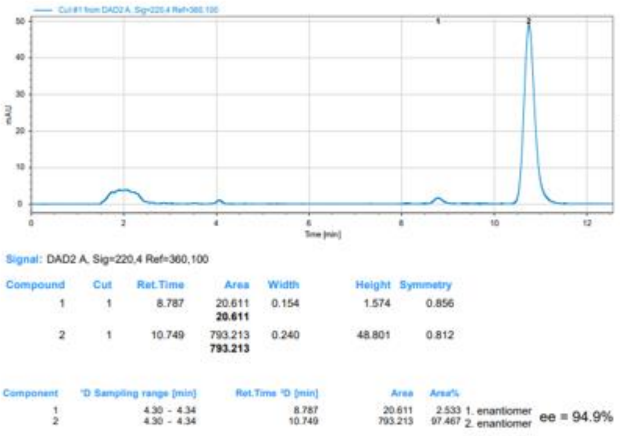

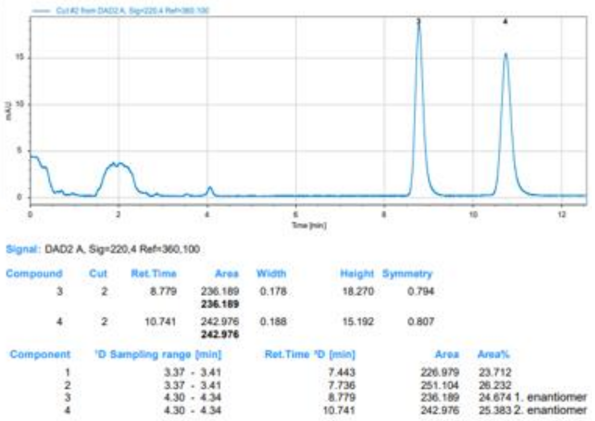

Figure S31. HPLC traces of compound **31a**: with complex **7b** (left); the corresponding racemate (right).

2,2,2-Trichloroethyl (*S*)-2-(4-fluorophenyl)-5-phenylpent-4-ynoate (31b). Prepared according to the general procedure **B** as a colorless liquid; with complex **7b**: <55% yield (the sample contained compound **11** as inseparable impurity, ca. 10%), 96% ee. [The ee was determined by HPLC analysis: Daicel 150 mm Chiralpak OD-3, Ø 4.6 mm, n-heptane/iso-propanol = 99/1, v = 1.0 mL/min, λ = 220 nm, t(minor) = 5.20 min, t(major) = 6.81 min]. $[\alpha]_D^{20}$ = 36.5 (c = 1.15, $CHCl_3$); ^{1}H NMR (400 MHz, $CDCl_3$): δ = 7.45 – 7.33 (m, 2H), 7.33 – 7.23 (m, 5H), 7.12 – 7.01 (m, 2H), 4.80 (d, *J* = 11.9 Hz, 1H), 4.74 (d, *J* = 11.9 Hz, 1H), 4.04 (t, *J* = 7.7 Hz, 1H), 3.20 (dd, *J* = 16.8, 8.0 Hz, 1H), 2.93 (dd, *J* = 16.8, 7.3 Hz, 1H); ^{13}C NMR (101 MHz, $CDCl_3$): δ = 171.0, 162.6 (d, *J* = 246.9 Hz), 132.6 (d, *J* = 3.2 Hz), 131.7, 129.9 (d, *J* = 8.4 Hz), 128.4, 128.2, 123.3, 115.8 (d, *J* = 21.6 Hz), 94.8, 86.2, 82.9, 74.3, 50.3, 24.1; ^{19}F NMR (282 MHz, $CDCl_3$) δ = –114.1; IR (ATR): ṽ = 2957, 1753, 1604, 1509, 1142, 1372, 1224, 1136, 1060, 837, 806, 755, 717, 691, 574, 518 cm^{-1}; HRMS (EI$^+$) for $C_{19}H_{14}Cl_3FO_2$ [M]$^+$: calcd: 398.00379, found: 398.00461.

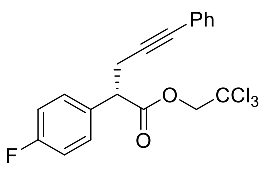

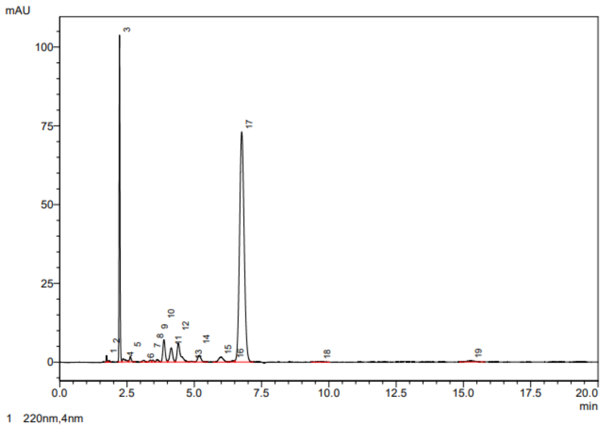

1 220nm,4nm

PDA Ch1 220nm

Peak #	Ret. Time	Area %	Name
1	1.74	0.35	
2	1.84	0.10	
3	2.22	18.14	
4	2.35	0.67	
5	2.63	0.57	
6	3.11	0.25	
7	3.37	0.25	
8	3.47	0.22	
9	3.62	0.39	
10	3.87	3.24	
11	4.14	2.52	
12	4.41	4.14	
13	4.89	0.14	
14	5.18	1.35	1st enantiomer
15	5.99	1.57	
16	6.44	0.29	
17	6.76	65.01	2nd enantiomer

ee = 95.9 %

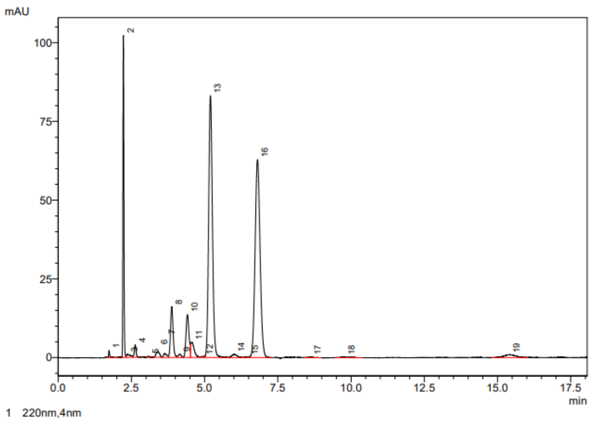

1 220nm,4nm

PDA Ch1 220nm

Peak #	Ret. Time	Area %	Name
1	1.74	0.29	
2	2.23	11.41	
3	2.35	0.44	
4	2.63	0.79	
5	3.08	0.07	
6	3.38	0.75	
7	3.64	0.46	
8	3.88	4.77	
9	4.15	0.36	
10	4.41	4.68	
11	4.57	1.93	
12	4.94	0.14	
13	5.20	35.82	1st enantiomer
14	6.01	0.65	
15	6.47	0.10	
16	6.81	35.82	2nd enantiomer
17	8.62	0.09	

Figure S32. HPLC traces of compound **31b**: with complex **7b** (left); the corresponding racemate (right).

5-Methyl 1-(2,2,2-trichloroethyl) (2*S*)-2-(4-fluorophenyl)-4-methylpentanedioate (33). Prepared

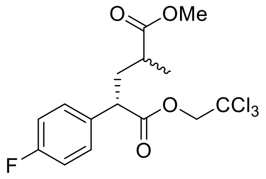

according to the general procedure **B** as a colorless liquid; with complex **7b**: 47% yield, dr = 1:1, 92% ee/93% ee for the two diastereomers [The ee's were determined by HPLC analysis: Daicel 150 mm Chiralpak OJ-3, Ø 4.6 mm, n-heptane/ethanol = 99/1, v = 1.0 mL/min, λ = 210 nm, t(minor diastereomer 1) = 6.90 min, t(major diastereomer 1) = 9.77 min; t(major diastereomer 2) = 7.67 min, t(minor diastereomer 2) = 8.39 min]. $[\alpha]_D^{20}$ = 39.5 (c = 0.95, $CHCl_3$); ^{1}H NMR (600 MHz, $CDCl_3$) δ = 7.31 (m, 2H), 7.29 (m, 2H), 7.02 (t, *J* = 8.7 Hz, 2H), 7.02 (m, 2H), 4.71 (m, 4H), 3.79 (dd, *J* = 9.0, 6.7 Hz, 1H), 3.76 (t, *J* = 7.8 Hz, 1H), 3.67 (s, 3H), 3.64 (s, 3H), 2.49 (ddd, *J* = 14.0, 8.9, 6.7 Hz, 1H), 2.45 (p, *J* = 7.1 Hz, 1H), 2.34 (dqd, *J* = 8.9, 7.1, 5.5 Hz, 1H), 2.21 (d, *J* = 7.4 Hz, 1H), 2.20 (d, *J* = 7.0 Hz, 1H), 1.95 (ddd, *J* = 13.9, 9.0, 5.5 Hz, 1H), 1.20 (d, *J* = 7.1 Hz, 3H), 1.18 (d, *J* = 7.0 Hz, 3H); ^{13}C NMR (151 MHz, $CDCl_3$) δ = 176.3, 176.2, 171.88 (d, *J* = 0.8 Hz), 171.87, 171.85, 171.84 (d, *J* = 0.8 Hz), 162.47 (d, *J* = 246.7 Hz), 162.45 (d, *J* = 246.4 Hz), 133.22 (d, *J* = 3.2 Hz), 133.16 (d, *J* = 3.2 Hz), 129.97 (d, *J* = 8.2 Hz), 129.84 (d, *J* = 8.1 Hz), 115.90, 115.86 (d, *J* = 21.7 Hz), 115.82 (d, *J* = 21.5 Hz), 115.75, 94.8, 74.23, 74.21, 51.92, 51.89, 48.8, 48.5, 37.6, 37.0, 36.8, 36.6, 17.7, 17.6; ^{19}F NMR (565 MHz, $CDCl_3$) δ = –114.46, –114.54; IR (ATR): ṽ = 2954, 1733, 1604, 1509, 1459, 1436, 1375, 1264, 1224, 1140, 1059, 839, 803, 716, 572, 519 cm^{-1}; HRMS (ESI$^+$) for $C_{15}H_{16}Cl_3FO_4Na$ $[M+Na]^+$: calcd: 406.99905, found: 406.99904.

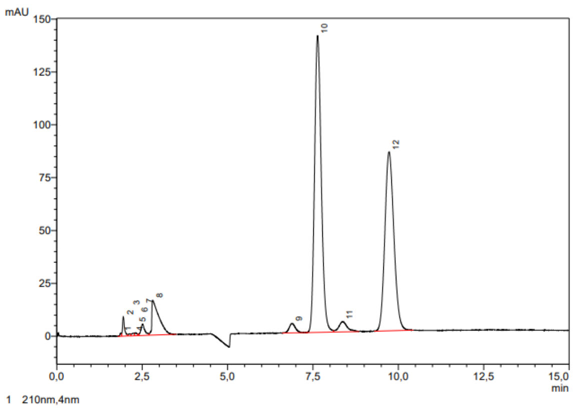

PDA Ch1 210nm

Peak #	Ret. Time	Area %	Name
1	1,88	0,11	
2	1,95	0,92	
3	2,14	0,13	
4	2,23	0,16	
5	2,31	0,16	
6	2,37	0,04	
7	2,51	1,09	
8	2,81	6,09	
9	6,88	1,43	1. Enantiomer Diastereomer 1
10	7,64	48,43	1. Enantiomer Diastereomer 2 91.6 % ee
11	8,37	2,13	2. Enantiomer Diastereomer 2
12	9,73	39,30	2. Enantiomer Diastereomer 1 93.0 % ee
Total		100,00	

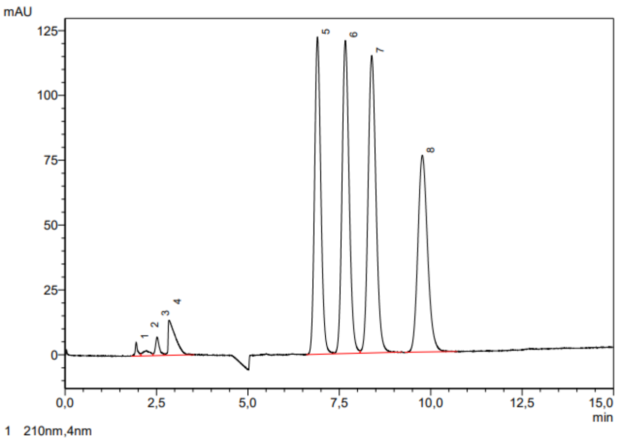

PDA Ch1 210nm

Peak #	Ret. Time	Area %	Name
1	1,95	0,38	
2	2,22	0,42	
3	2,52	0,81	
4	2,85	2,89	
5	6,90	22,45	1. Enantiomer Diastereomer 1
6	7,67	25,03	1. Enantiomer Diastereomer 2
7	8,39	26,49	2. Enantiomer Diastereomer 2
8	9,77	21,53	2. Enantiomer Diastereomer 1
Total		100,00	

Figure S33. HPLC traces of compound **33**: with complex **7b** (left); the corresponding racemate (right).

Reaction with Gaseous Substrates

Representative Procedure for C–H Insertion into Ethane. 2,2,2-Trichloroethyl (*S*)-2-(4-fluorophenyl)butanoate (34a).

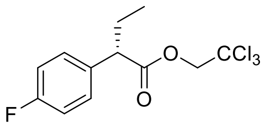

A 45 mL stainless steel autoclave equipped with a magnetic stir bar was charged with the catalyst (0.001 mmol, 1 mol%). The autoclave was evacuated and backfilled with argon 3 times and then purged with ethane. C_6F_6 (1 mL) was added and the autoclave was pressurized with ethane to ≈ 25 bar. A solution of the diazo derivative **8c** (31.2 mg, 0.1 mmol) in C_6F_6 (3 mL) was added dropwise over 30 min to the pressurized autoclave with the help of an hplc pump. After the addition was complete, the mixture was left stirring at room temperature for 2 h. The pressure was carefully released and the mixture was absorbed on silica, which was loaded on top of a silica column. Purification by flash chromatography (hexanes/EtOAc) afforded the title compound; with complex **7b**: 80% yield, 90% ee; with complex **7d**: 61% yield, 95% ee. [The ee was determined by HPLC analysis: Daicel 150 mm Chiralpak IB-N-3, Ø 4.6 mm, n-heptane2-propanol = 99.9/0.1, v = 1.0 mL/min, λ = 220 nm, t(minor) = 4.72 min, t(major) = 5.00 min]. $[\alpha]_D^{20}$ = 24.2 (c = 1.2, $CHCl_3$); ^1H NMR (400 MHz, $CDCl_3$): δ = 7.36 – 7.22 (m, 2H), 7.07 – 6.97 (m, 2H), 4.78 – 4.63 (m, 2H), 3.59 (t, *J* = 7.7 Hz, 1H), 2.25 – 2.09 (m, 1H), 1.94 – 1.78 (m, 1H), 0.93 (t, *J* = 7.4 Hz, 3H); ^{13}C NMR (101 MHz, $CDCl_3$): δ = 172.4, 162.3 (d, *J* = 245.9 Hz), 133.8 (d, *J* = 3.1 Hz), 129.9 (d, *J* = 8.0 Hz), 115.6 (d, *J* = 21.2 Hz), 95.0, 74.1, 52.6, 26.6, 12.2; ^{19}F NMR (282 MHz, $CDCl_3$): δ = −115.07; IR (ATR): ṽ = 1749, 1604, 1509, 1224, 1161, 1138, 1089, 836, 804, 785, 770, 715, 573, 519 cm^{-1}; HRMS (EI^+) for $C_{12}H_{12}Cl_3FO_2$ $[M]^+$: calcd: 311.98814, found: 311.98807.

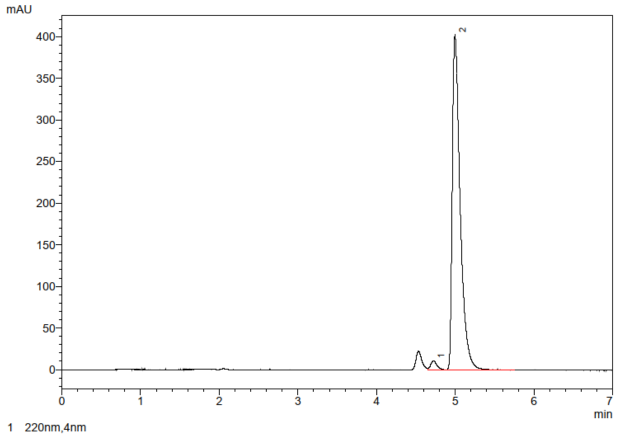

PDA Ch1 220nm

Peak #	Ret. Time	Area %
1	4.72	2.26
2	5.00	97.74
Total		100.00

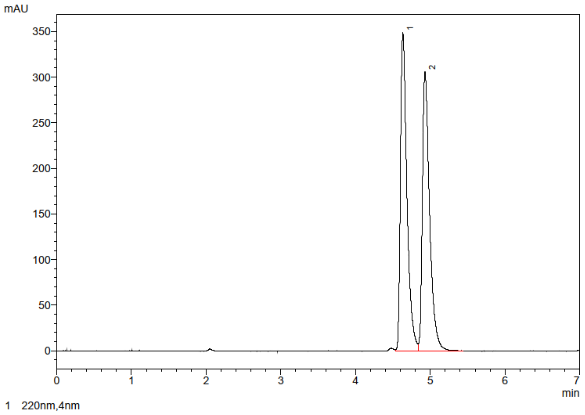

PDA Ch1 220nm

Peak #	Ret. Time	Area %
1	4.63	50.74
2	4.93	49.26
Total		100.00

Figure S34. HPLC traces of compound **34a**: with complex **7d** (left); the corresponding racemate (right).

2,2,2-Trichloroethyl (*S*)-2-(4-bromophenyl)butanoate (34b). Prepared analogously as a colorless liquid;

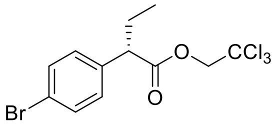

with complex **7b**: 80% yield, 94% ee; [The ee was determined by HPLC analysis: Daicel 150 mm Chiralpak IB-N-3, Ø 4.6 mm, n-heptane/2-propanol = 99.9/0.1, v = 1.0 mL/min, λ = 220 nm, t(minor) = 5.52 min, t(major) = 5.89 min]. $[\alpha]_D^{20}$ = 14.3 (c = 2.5, $CHCl_3$); ^1H NMR (400 MHz, $CDCl_3$): δ = 7.49 – 7.42 (m, 2H), 7.27 – 7.19 (m, 2H), 4.75 (d, *J* = 12.0 Hz, 1H), 4.69 (d, *J* = 12.0 Hz, 1H), 3.57 (t, *J* = 7.7 Hz, 1H), 2.24 – 2.09 (m, 1H), 1.86 (dt, *J* = 13.6, 7.4 Hz, 1H), 0.93 (t, *J* = 7.4 Hz, 3H); ^{13}C NMR (101 MHz, $CDCl_3$): δ = 172.0, 137.1, 131.9, 130.0, 121.7, 94.9, 74.2, 52.8, 26.5, 12.2; IR (ATR): ṽ = 1749, 1488, 1458, 1408, 1371, 1265, 1511, 1193, 1139, 1091, 1073, 1011, 826, 785, 716, 571, 515 cm^{-1}; HRMS (EI^+) for $C_{12}H_{12}BrCl_3O_2$ $[M]^+$: calcd: 371.90809, found: 371.90832.

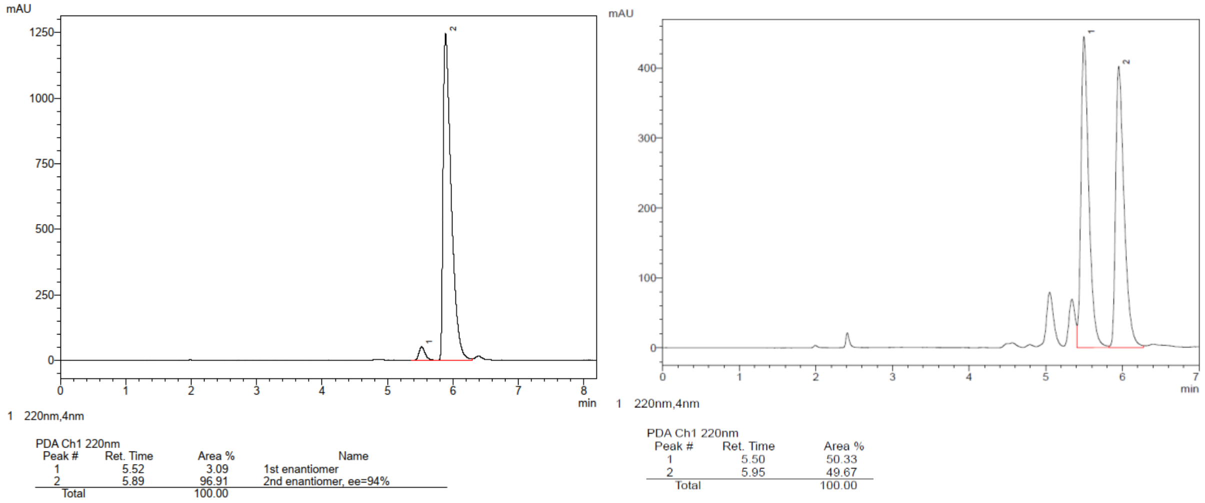

Figure S35. HPLC traces of compound **34b**: with complex **7b** (left); the corresponding racemate (right).

Methyl (*S*)-4-(1-oxo-1-(2,2,2-trichloroethoxy)butan-2-yl)benzoate (34c). A 45 mL stainless steel autoclave

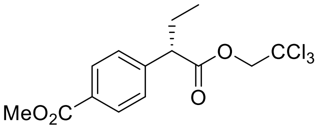

equipped with a magnetic stir bar was charged with catalyst **7b** (0.001 mmol, 1 mol%) and the diazo compound (0.1 mmol). The autoclave was evacuated, backfilled with argon 3 times, and then purged with ethane. Next, the autoclave was cooled with dry ice, C_6F_6 (3 mL) was added. The autoclave was pressurized with ethane to 25 bar and the mixture was left stirring for 2 h while slowly reaching room temperature. After that, the pressure was released and the mixture was absorbed on silica, which was loaded on top of a silica column. Purification by flash chromatography (hexanes/EtOAc) afforded the title compound as a colorless liquid (15.2 mg, 43% yield, 96% ee). [The ee was determined by HPLC analysis: Daicel 150 mm Chiralpak IB-N-3, Ø 4.6 mm, n-heptane/2-propanol = 98/2, v = 1.0 mL/min, λ = 220 nm, t(minor) = 5.50 min, t(major) = 6.60 min]. $[\alpha]_D^{20}$ = 20.5 (c = 1.4, $CHCl_3$); ^1H NMR (400 MHz, $CDCl_3$): δ = 8.05 – 7.96 (m, 2H), 7.46 – 7.38 (m, 2H), 4.78 – 4.65 (m, 2H), 3.91 (s, 3H), 3.67 (t, *J* = 7.7 Hz, 1H), 2.28 – 2.11 (m, 1H), 1.98 – 1.82 (m, 1H), 0.94 (t, *J* = 7.4 Hz, 3H); ^{13}C NMR (101 MHz, $CDCl_3$): δ = 171.8, 166.9, 143.2, 130.1, 129.6, 128.4, 94.9, 74.2, 53.4, 52.3, 26.5, 12.2; IR (ATR): ṽ = 1751, 1721, 1611, 1435, 1276, 1182, 1141, 1109, 1019, 857, 815, 786, 717, 572 cm^{-1}; HRMS (ESI^+) for $C_{14}H_{15}Cl_3O_4Na$ $[M+Na]^+$: calcd: 374.99281, found: 374.99305.

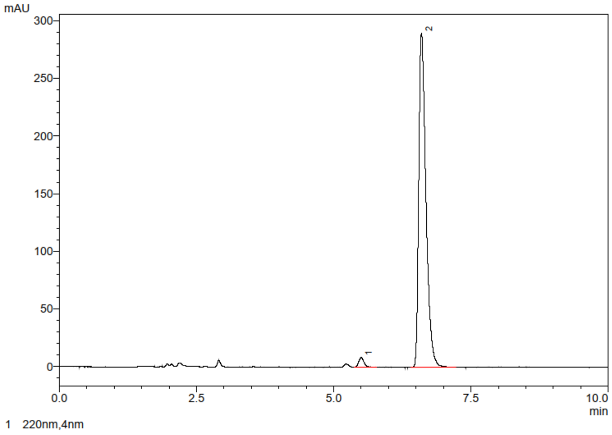

PDA Ch1 220nm

Peak #	Ret. Time	Area %	Name
1	5.50	2.12	
2	6.60	97.88	
Total		100.00	

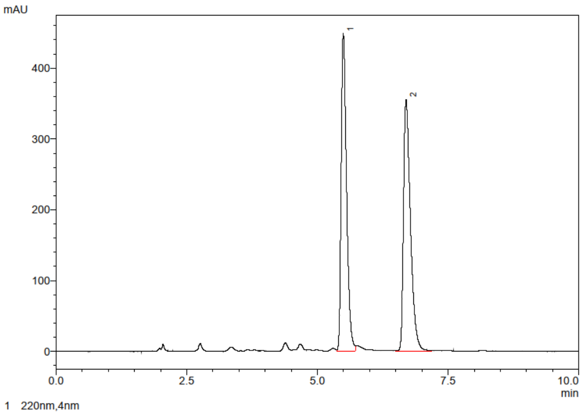

PDA Ch1 220nm

Peak #	Ret. Time	Area %	Name
1	5.50	49.30	
2	6.70	50.70	
Total		100.00	

Figure S36. HPLC traces of compound **34c**: with complex **7b** (left); the corresponding racemate (right).

Cyclopropanation of Propene. 2,2,2-Trichloroethyl (1*S*,2*S*)-1-(4-fluorophenyl)-2-methylcyclopropane-1-carboxylate (35).

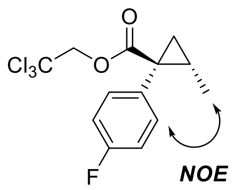

A 45 mL stainless steel autoclave equipped with a magnetic stir bar was charged with catalyst **7b** (1.45 mg, 0.0005 mmol, 0.5 mol%). The autoclave was evacuated and backfilled with argon 3 times and purged with propene. Pentane (1 mL) was added and the autoclave was pressurized with propene to 9 bar. A solution of the diazo compound **8c** (31.2 mg, 0.1 mmol) in pentane (3 mL) was added over 30 min into the pressurized autoclave with the help of an hplc pump. After the addition was complete, the reaction mixture was left stirring at room temperature for 2 h before the pressure was carefully released and the reaction mixture was absorbed on silica, which was loaded on top of a silica column. Purification by flash chromatography (hexanes/EtOAc) afforded the title compound as a colorless liquid (28.0 mg, 86% yield, 94% ee). [The ee was determined by HPLC analysis: Daicel 150 mm Chiralcel OJ-3R, Ø 4.6 mm, acetonitrile/water = 60/40, v = 0.5 mL/min, λ = 220 nm, t(minor) = 23.42 min, t(major) = 25.67 min]. $[\alpha]_D^{20}$ = −4.9 (c = 2.2, $CHCl_3$); ^1H NMR (400 MHz, $CDCl_3$): δ = 7.31 – 7.22 (m, 2H), 7.08 – 6.97 (m, 2H), 4.76 (d, *J* = 11.9 Hz, 1H), 4.56 (d, *J* = 11.9 Hz, 1H), 1.99 (dp, *J* = 9.1, 6.3 Hz, 1H), 1.89 (dd, *J* = 9.0, 4.2 Hz, 1H), 1.15 (dd, *J* = 6.8, 4.2 Hz, 1H), 0.87 (d, *J* = 6.2 Hz, 3H); ^{13}C NMR (101 MHz, $CDCl_3$): δ = 173.0, 162.2 (d, *J* = 246.0 Hz), 133.3 (d, *J* = 8.2 Hz), 131.0 (d, *J* = 3.2 Hz), 115.1 (d, *J* = 21.4 Hz), 95.2, 74.4, 33.0, 23.9, 23.4, 15.5; ^{19}F NMR (282 MHz, $CDCl_3$): δ = −115.05; IR (ATR): ṽ = 1731, 1512, 1369, 1246, 1222, 1158, 1115, 1090, 1046, 884, 839, 803, 752, 719, 590, 571, 543 cm^{-1}; HRMS (EI^+) for $C_{13}H_{12}Cl_3FO_2$ $[M]^+$: calcd: 323.98814, found: 323.98815.

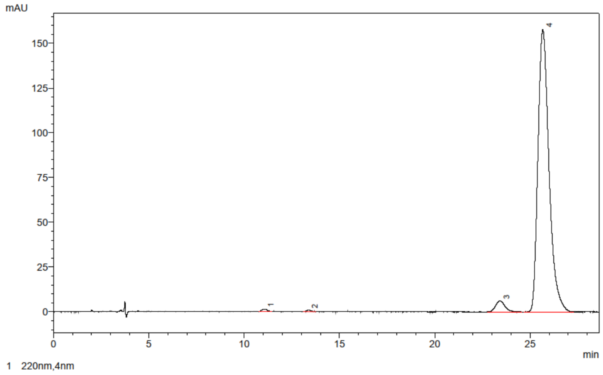

1 220nm,4nm

PDA Ch1 220nm

Peak #	Ret. Time	Area %	Name
1	11,06	0,42	
2	13,37	0,23	
3	23,42	3,18	1. Enantiomer
4	25,67	96,18	2. Enantiomer 93.6 % ee
Total		100,00	

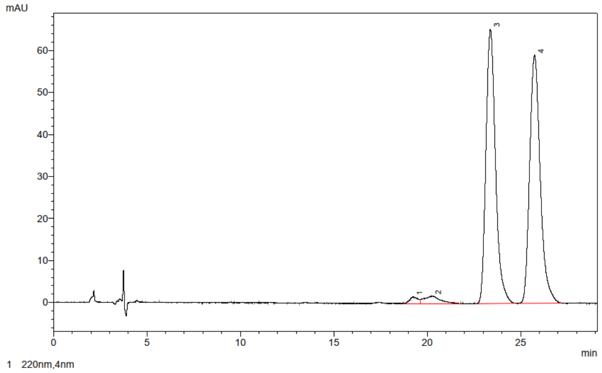

1 220nm,4nm

PDA Ch1 220nm

Peak #	Ret. Time	Area %	Name
1	19,25	1,03	
2	20,25	2,26	
3	23,38	48,37	1. Enantiomer
4	25,75	48,35	2. Enantiomer
Total		100,00	

Figure S37. HPLC traces of compound **35**: with complex **7b** (left); the corresponding racemate (right).

Further Reactions

2,2,2-Trichloroethyl (1*R*,2*R*)-2-bromo-1-(4-fluorophenyl)-2-methylcyclopropane-1-carboxylate (37a).

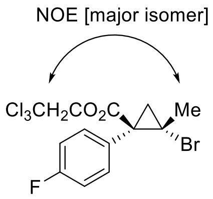

Prepared according to the general procedure **B** as a colorless liquid; with complex **7b**: 69% yield, dr = 95:5, 92% ee. [The ee was determined by HPLC analysis: Daicel 150 mm Chiralcel OJ-3R, Ø 4.6 mm, methanol/water = 85/15, v = 0.5 mL/min, λ = 220 nm, t(minor) = 19.13 min, t(major) = 21.51 min]. $[\alpha]_D^{20}$= 26.2 (c = 0.7, $CHCl_3$); ^1H NMR (400 MHz, $CDCl_3$): δ = 7.49 – 7.40 (m, 2H), 7.10 – 6.99 (m, 2H), 4.80 (d, *J* = 11.9 Hz, 1H), 4.60 (d, *J* = 11.9 Hz, 1H), 2.20 (d, *J* = 6.8 Hz, 1H), 2.02 (s, 3H), 1.85 (d, *J* = 6.8 Hz, 1H); ^{13}C NMR (101 MHz, $CDCl_3$): δ = 168.2, 162.6 (d, *J* = 247.0 Hz), 133.4 (d, *J* = 8.5 Hz), 132.9 (d, *J* = 3.4 Hz), 114.9 (d, *J* = 21.6 Hz), 94.5, 75.0, 40.6, 39.8, 28.8, 26.6; ^{19}F NMR (282 MHz, $CDCl_3$): δ = –113.6; IR (ATR): ṽ = 2962, 2927, 1729, 1602, 1509, 1424, 1371, 1291, 1265, 1217, 1155, 1111, 1054, 851, 806, 713, 600, 567, 529 cm^{-1}; HRMS (EI^+) for $C_{13}H_{11}BrCl_3FO_2$ $[M]^+$: calcd: 401.89867, found: 401.89908.

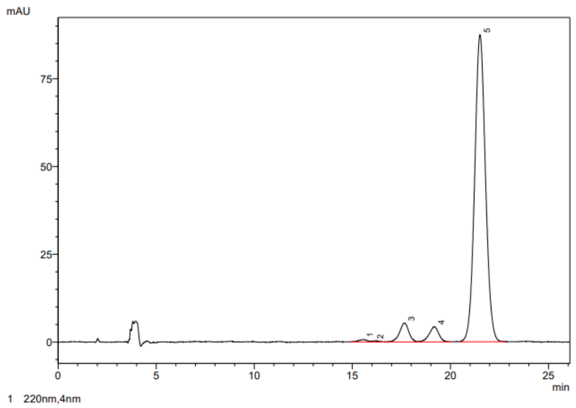

PDA Ch1 220nm

Peak #	Ret. Time	Area %	Name
1	15,55	0,55	
2	16,07	0,24	1. Enantiomer Diastereomer 1
3	17,65	4,79	2. Enantiomer Diastereomer 1
4	19,18	4,01	1. Enantiomer Diastereomer 2
5	21,51	90,41	2. Enantiomer Diastereomer 2 91.5 % ee
Total		100,00	

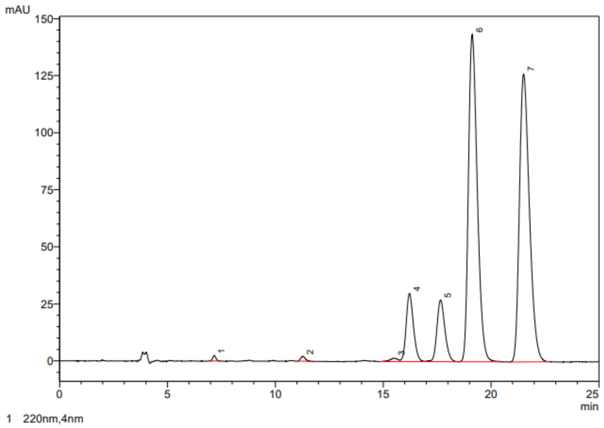

PDA Ch1 220nm

Peak #	Ret. Time	Area %	Name
1	7,17	0,25	
2	11,27	0,33	
3	15,51	0,40	
4	16,22	7,29	1. Enantiomer Diastereomer 1
5	17,66	7,23	2. Enantiomer Diastereomer 1
6	19,13	42,36	1. Enantiomer Diastereomer 2
7	21,51	42,13	2. Enantiomer Diastereomer 2
Total		100,00	

Figure S38. HPLC traces of compound **37a**: with complex **7b** (left); the corresponding racemate (right).

2,2,2-Trichloroethyl (1*R*,2*S*)-1-(4-fluorophenyl)-2-methyl-2-((trimethylsilyl)methyl)cyclopropane-1-carboxylate (37b).

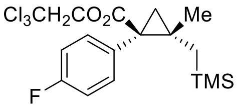

Prepared according to the general procedure **B** as a colorless liquid; with complex **7b**: 68% yield, dr = 63:37, major 90% ee, minor 95% ee. [The ee was determined by HPLC analysis: Daicel 150 mm Chiralpak IG-G, Ø 4.6 mm, Acetonitrile/water = 65/35, v = 1.0 mL/min, λ = 220 nm, major diastereomer t(minor) = 10.38 min, t(major) = 11.95 min; diastereoisomer t(major) = 9.43 min, t(minor) = 10.12 min]. $[\alpha]_D^{20}$ = –32.1 (c = 0.6, $CHCl_3$); ^1H NMR (400 MHz, $CDCl_3$): δ = 7.33 (ddd, *J* = 8.5, 5.3, 2.5 Hz, 2.80H), 7.04 – 6.93 (m, 2.64H), 4.78 (d, *J* = 12.0 Hz, 0.36H), 4.70 (d, *J* = 12.0 Hz, 1H), 4.62 (d, *J* = 12.0 Hz, 1H), 4.54 (d, *J* = 12.0 Hz, 0.35H), 1.84 (dd, *J* = 5.1, 1.5 Hz, 0.34H), 1.79 (d, *J* = 5.0 Hz, 1H), 1.33 (d, *J* = 1.1 Hz, 1H), 1.27 – 1.24 (m, 1H), 1.15 (d, *J* = 1.1 Hz, 0.38H) 0.88 (s, 3H), 0.09 (s, 9H), -0.00 (s, 3.12H).; ^{13}C NMR (101 MHz, $CDCl_3$): δ = 170.7 (2C), 162.1 (d, *J* = 245.7 Hz), 162.0 (d, *J* = 245.5 Hz), 133.6 (d, *J* = 8.1 Hz), 133.2 (d, *J* = 8.0 Hz), 133.1 (m), 129.6 (d, *J* = 8.4 Hz), 114.9 (d, *J* = 21.2 Hz), 114.8 (d, *J* = 21.5 Hz), 95.02, 94.97, 74.8, 74.7, 39.6, 39.4, 30.9, 30.6, 28.1, 27.2, 26.5, 25.9, 24.2, 22.4, 21.6, 21.1, –0.01, –0.03; ^{19}F NMR (282 MHz, $CDCl_3$): δ = –115.3, –115.4; IR (ATR): ṽ = 2954, 1732, 1603, 1510, 1302, 1248, 1222, 1181, 1130, 1050, 835, 806, 755, 718, 570, 546 cm^{-1}; HRMS (EI^+) for $C_{17}H_{22}Cl_3FO_2Si$ $[M]^+$: calcd: 410.04332, found: 410.04372.

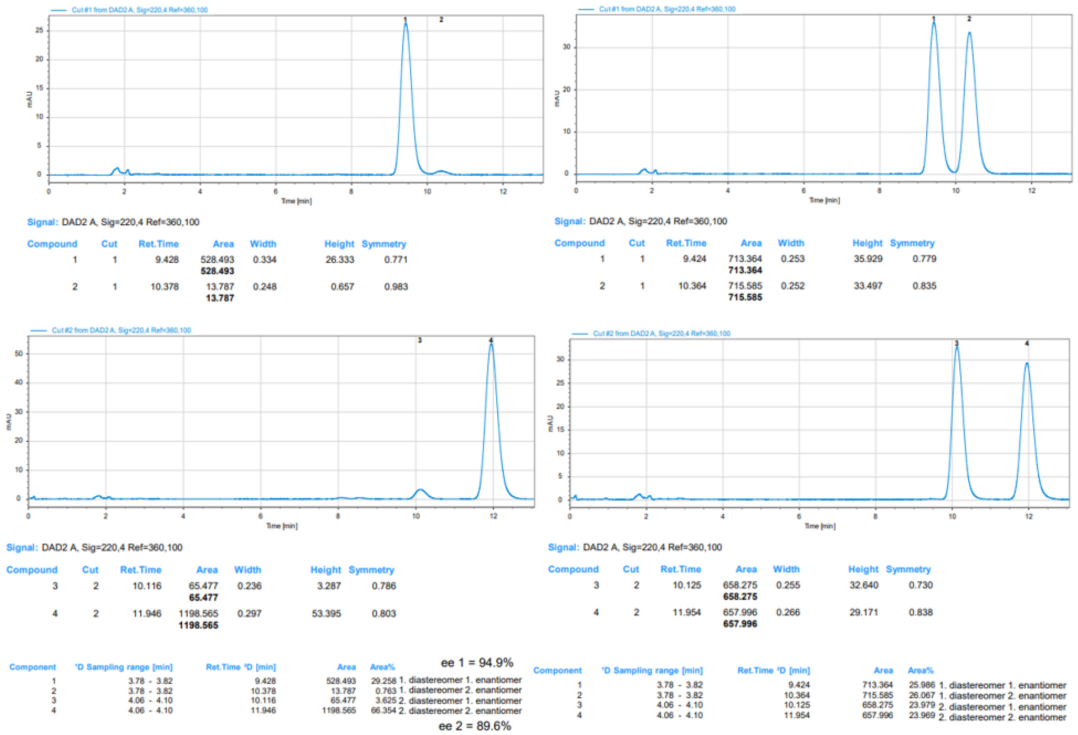

Figure S39. HPLC traces of compound **37b** with complex **7b**: minor diastereomer (top left); the corresponding racemate (top right); major diastereomer (bottom left); the corresponding racemate (bottom right).

S2: ^{1}H NMR (400 MHz, $CDCl_3$):

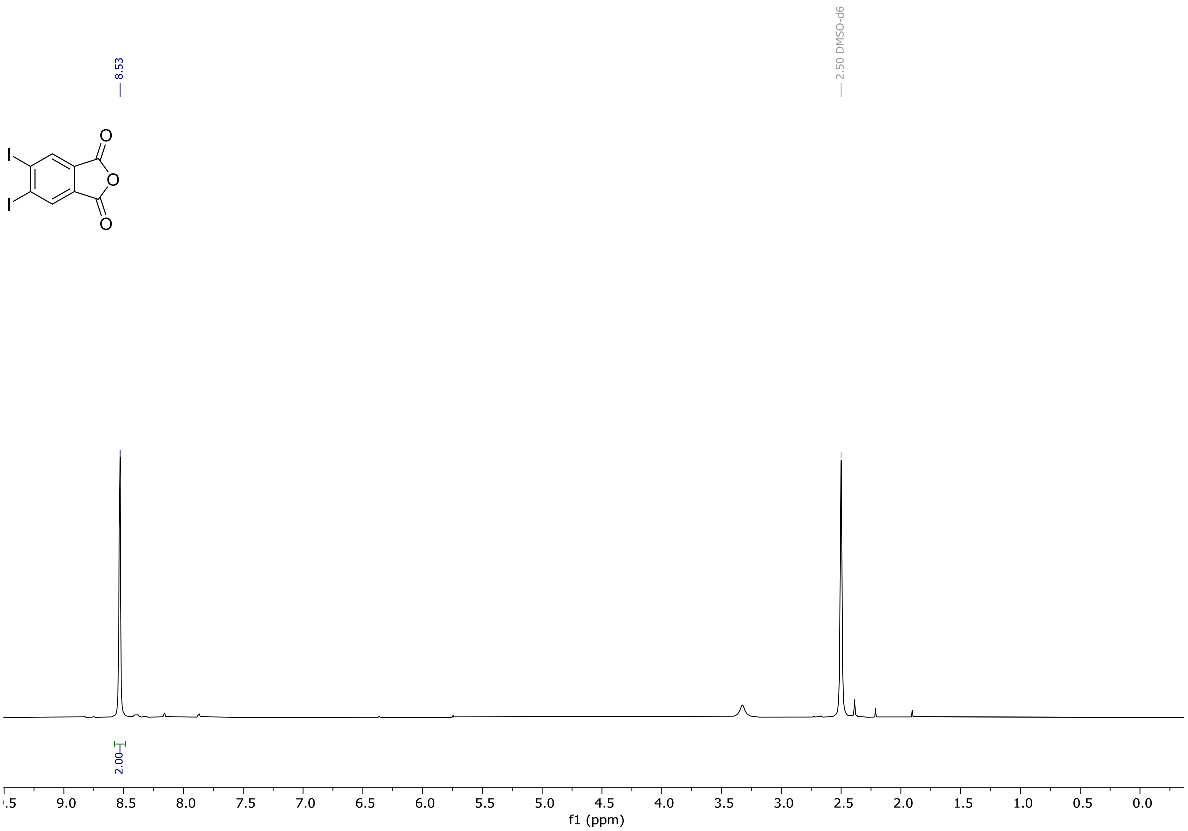

S2: ^{13}C NMR (101 MHz, $CDCl_3$):

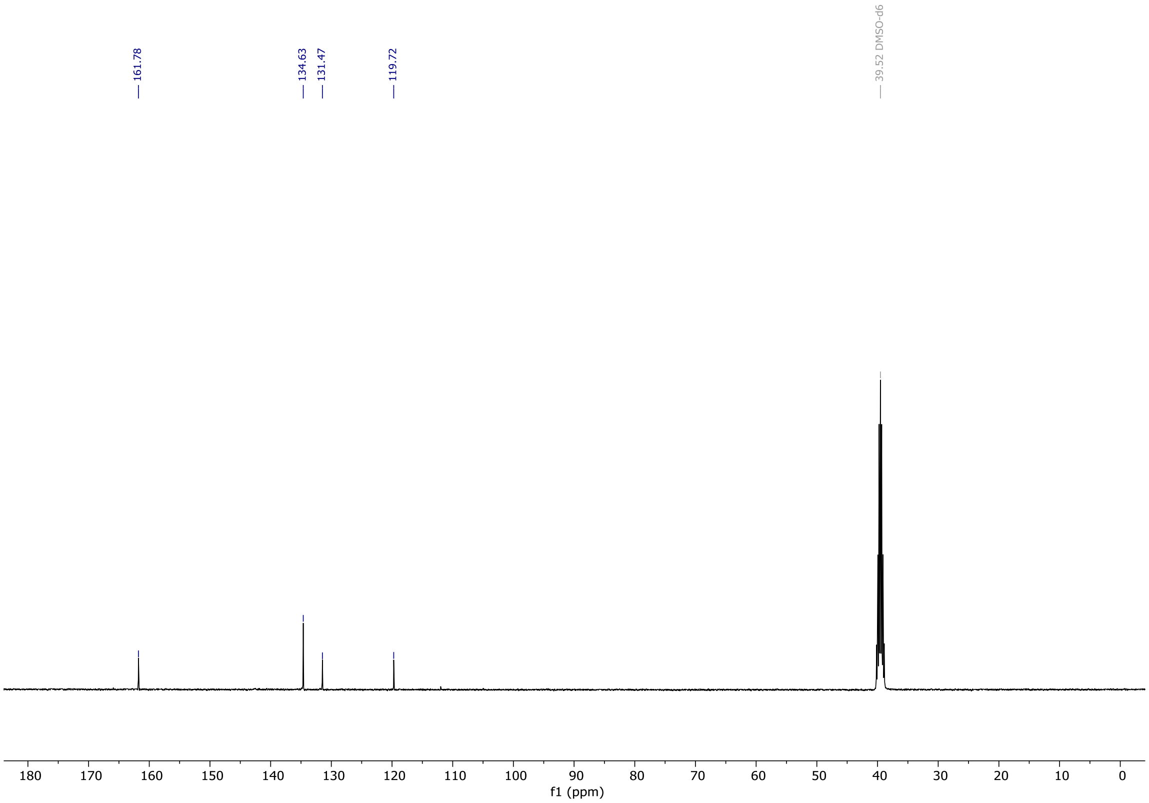

S4: ^{1}H NMR (400 MHz, CDCl$_3$):

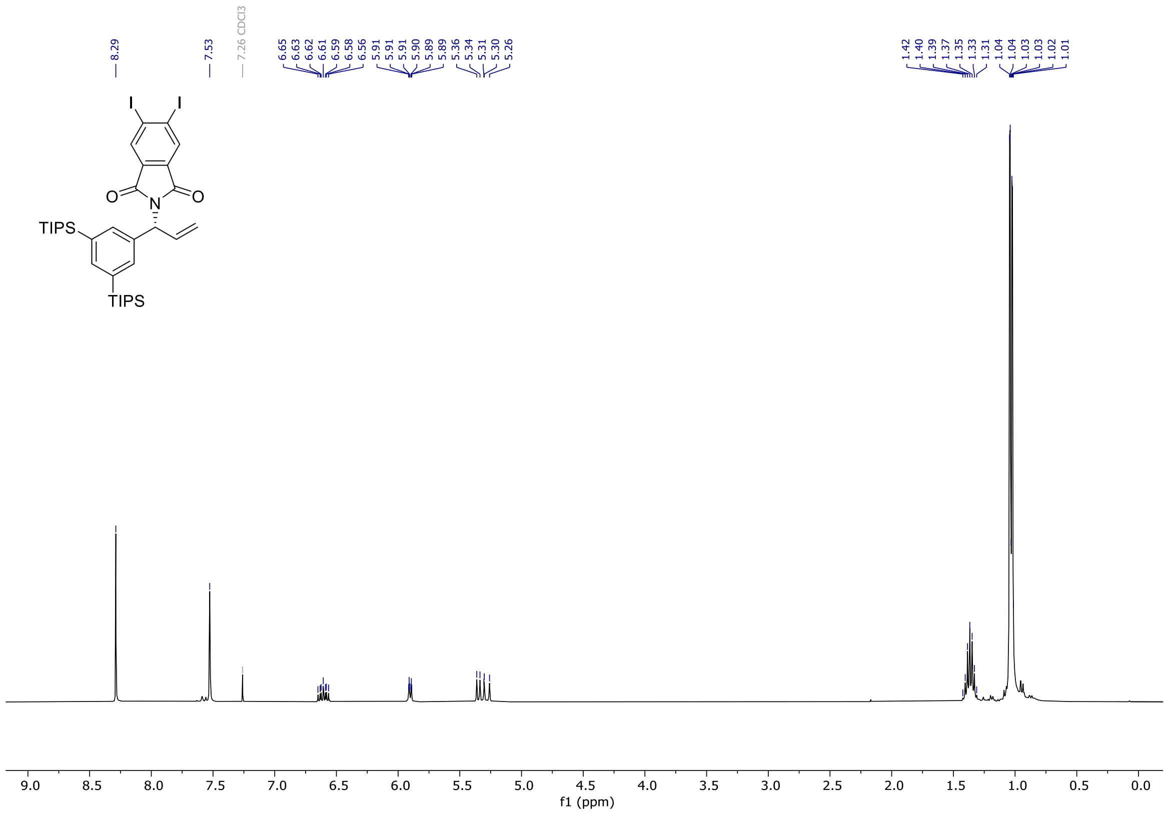

S4: ^{13}C NMR (101 MHz, CDCl$_3$):

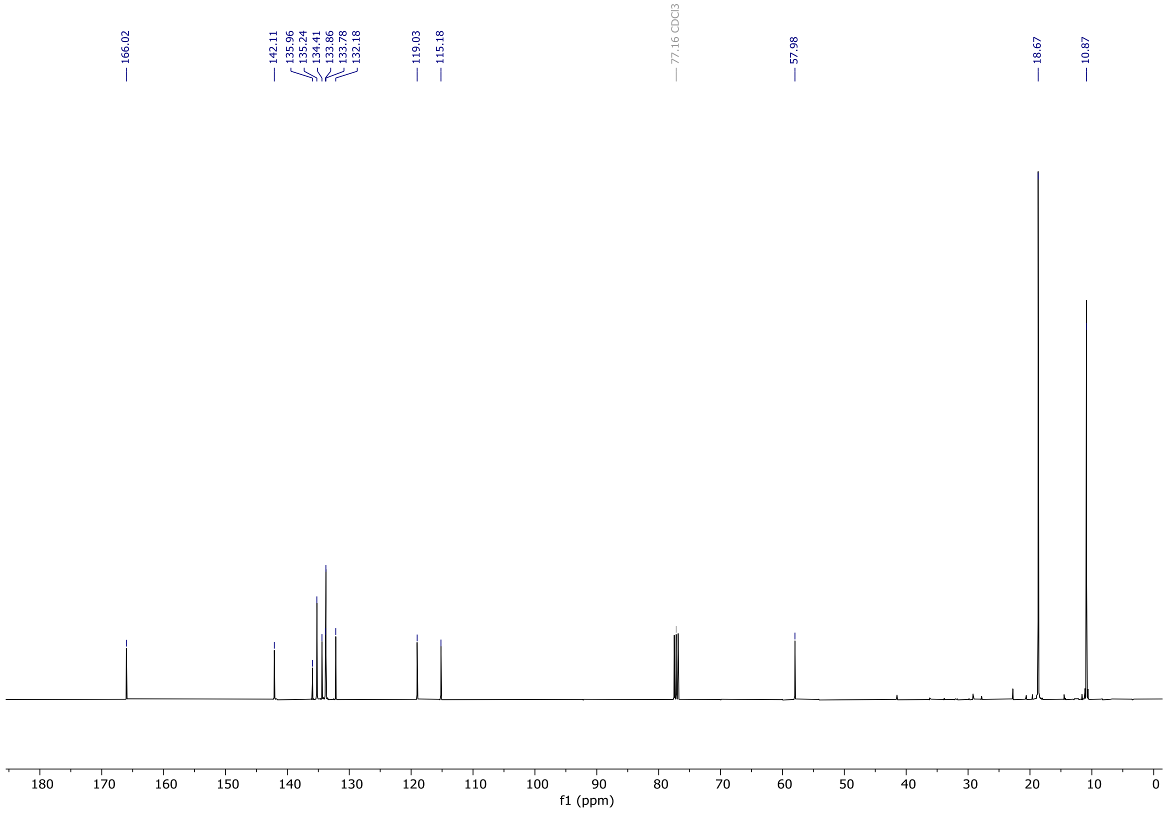

S5: ^{1}H NMR (400 MHz, $CDCl_3$)

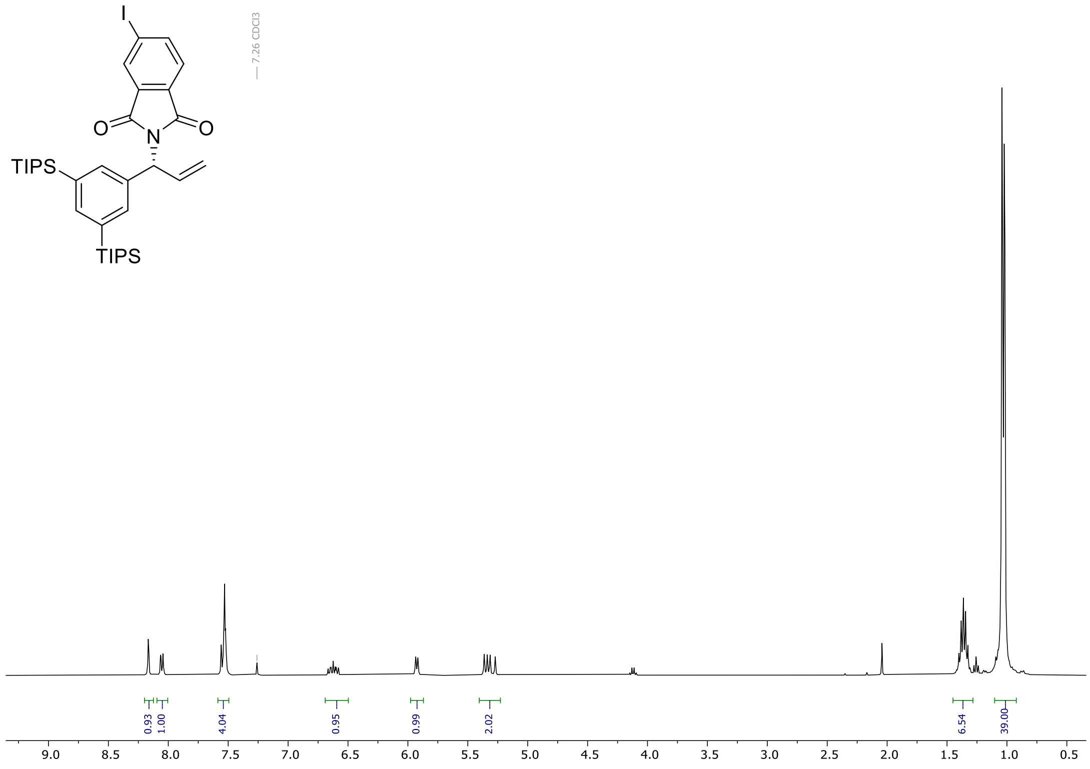

S5: ^{13}C NMR (101 MHz, $CDCl_3$)

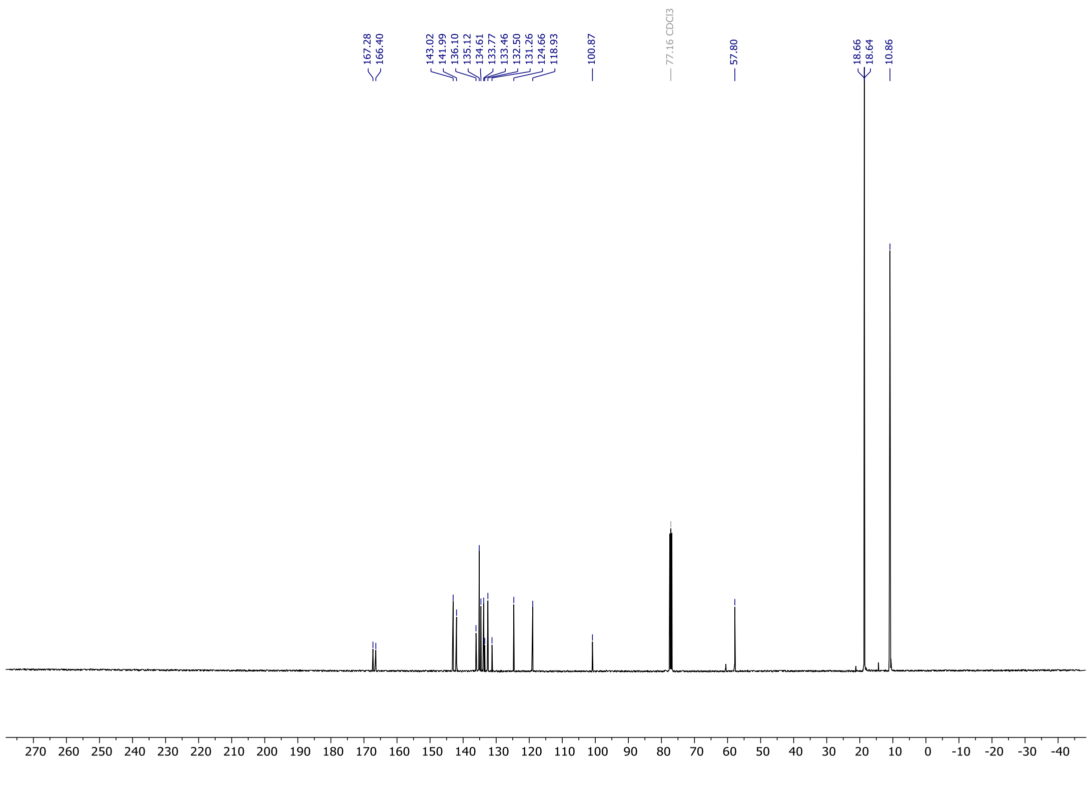

S6: ^{1}H NMR (400 MHz, $CDCl_3$):

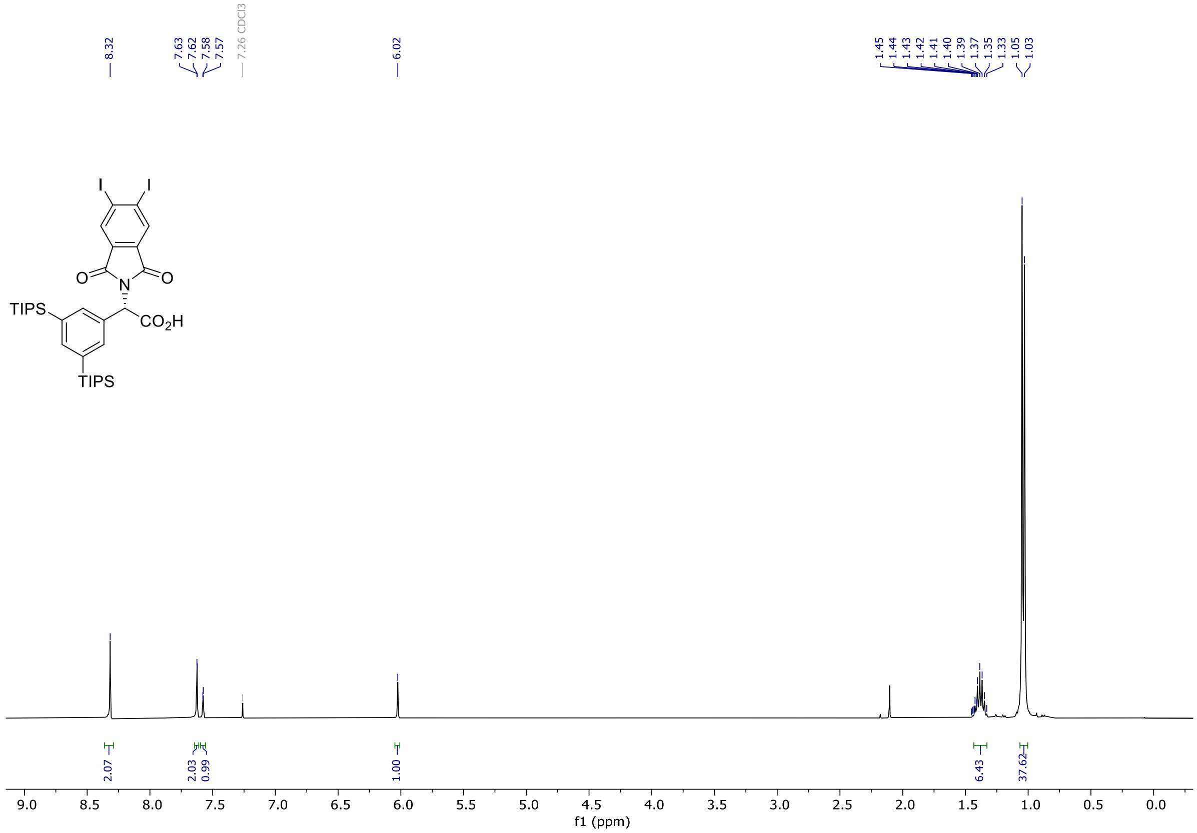

S6: ^{13}C NMR (101 MHz, $CDCl_3$):

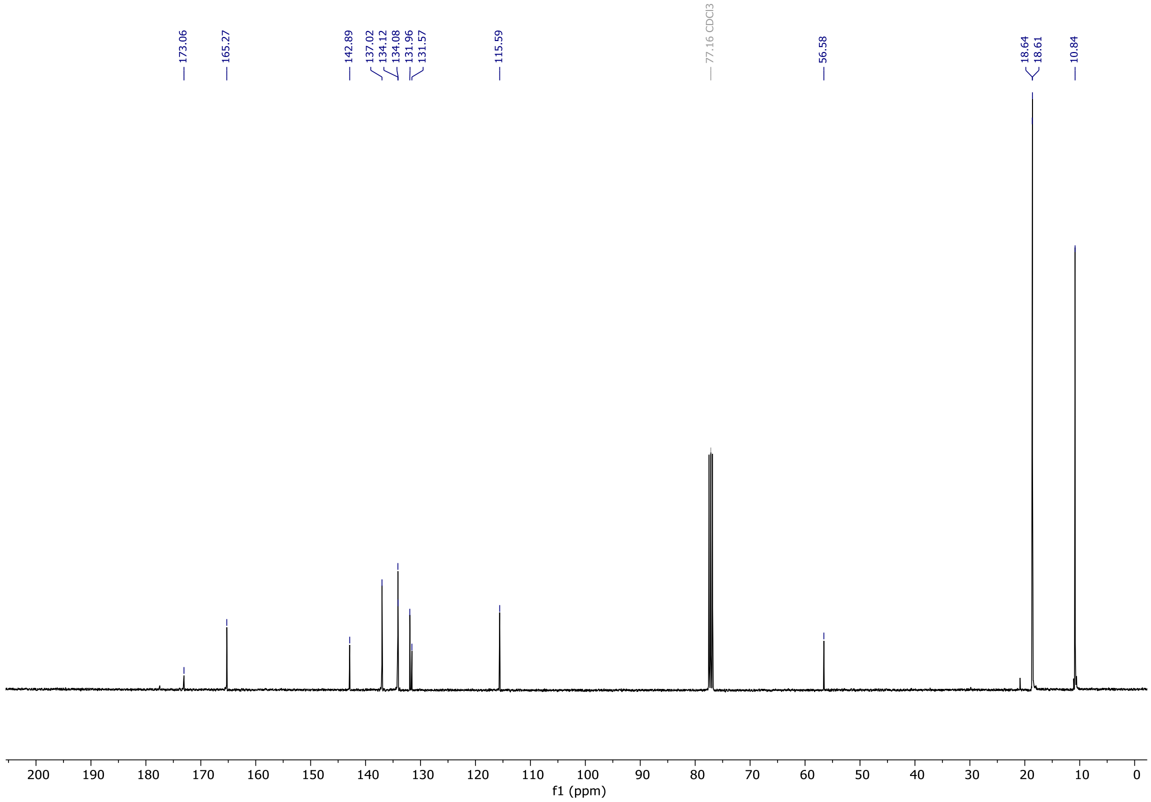

S7: ^{1}H NMR (400 MHz, $CDCl_3$)

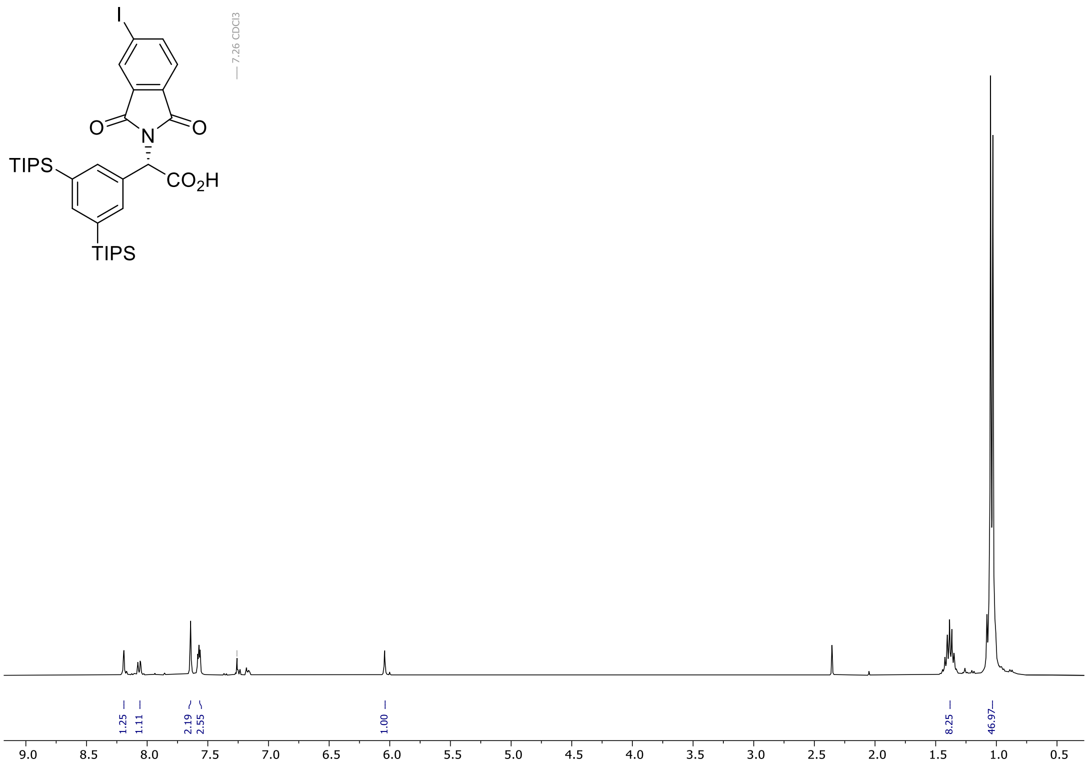

S7: ^{13}C NMR (101 MHz, $CDCl_3$)

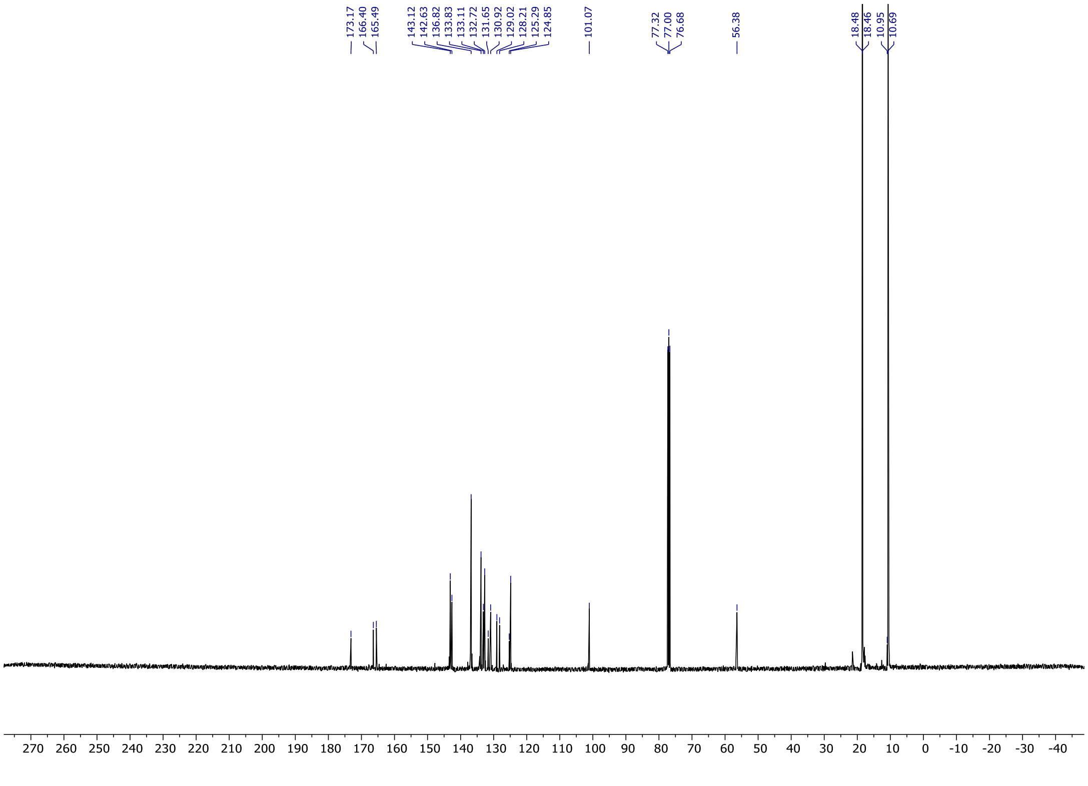

7b: ^{1}H NMR (600 MHz, $CDCl_3$):

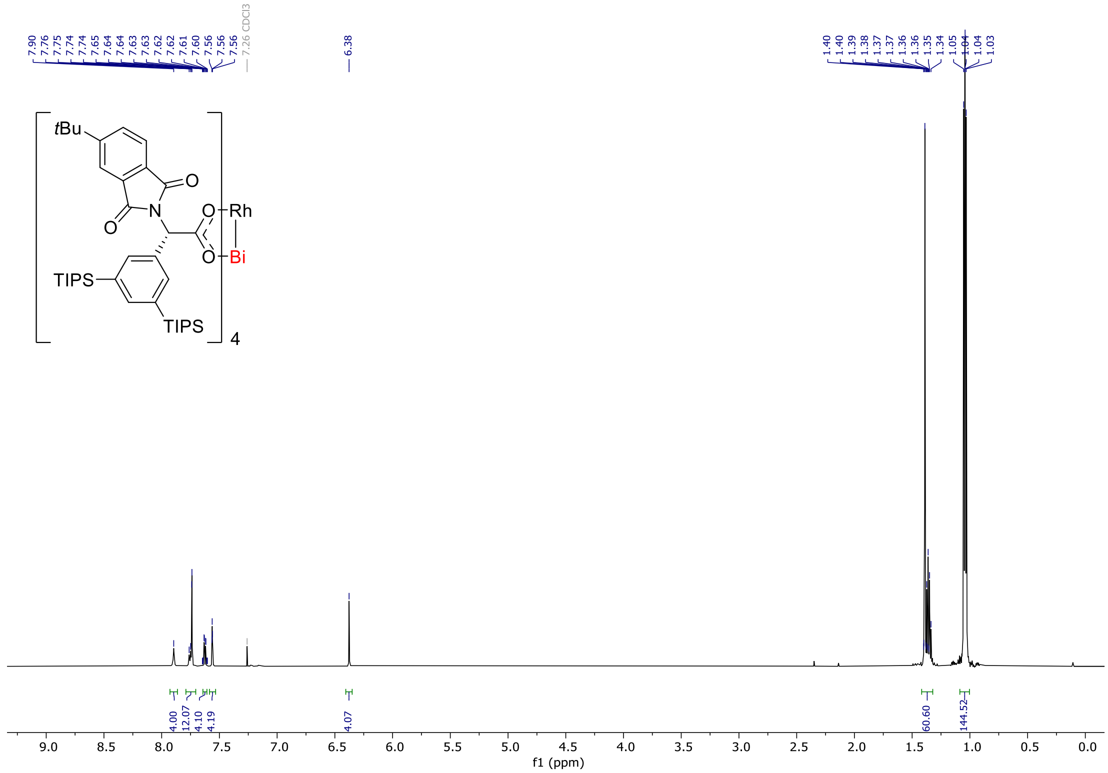

7b: ^{13}C NMR (151 MHz, $CDCl_3$):

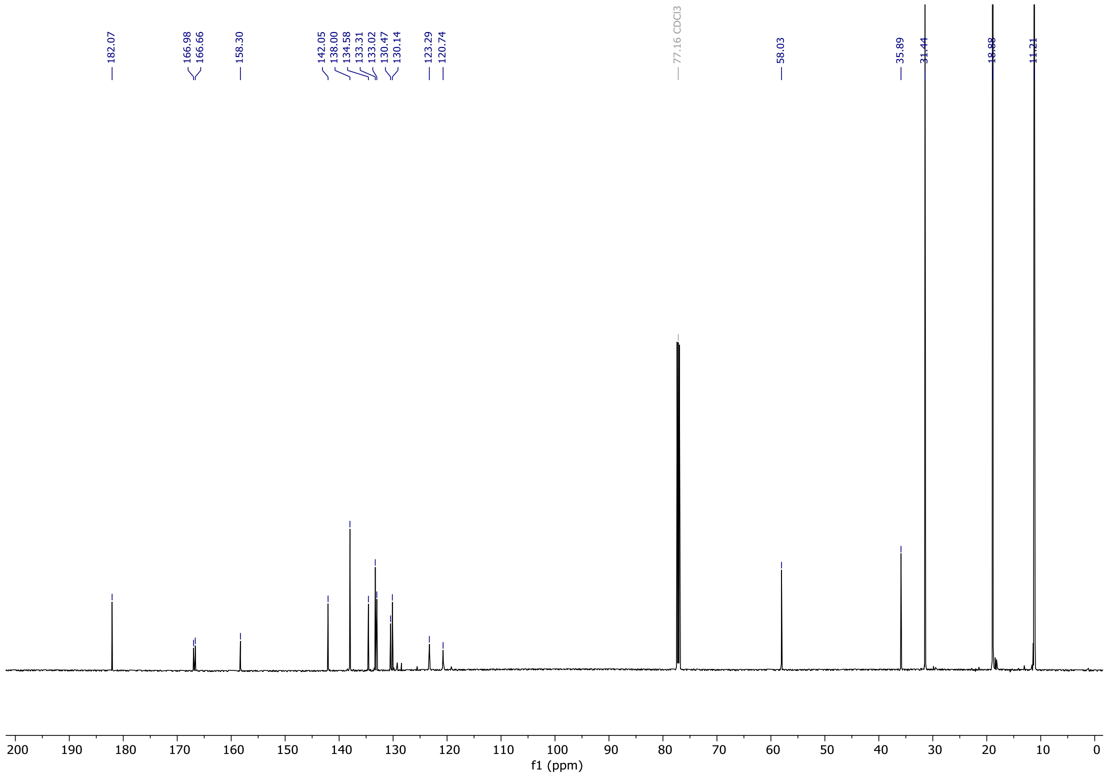

7c: ^{1}H NMR (600 MHz, $CDCl_3$, 353K)

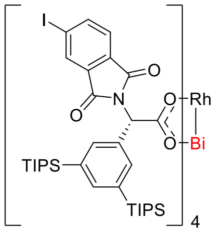

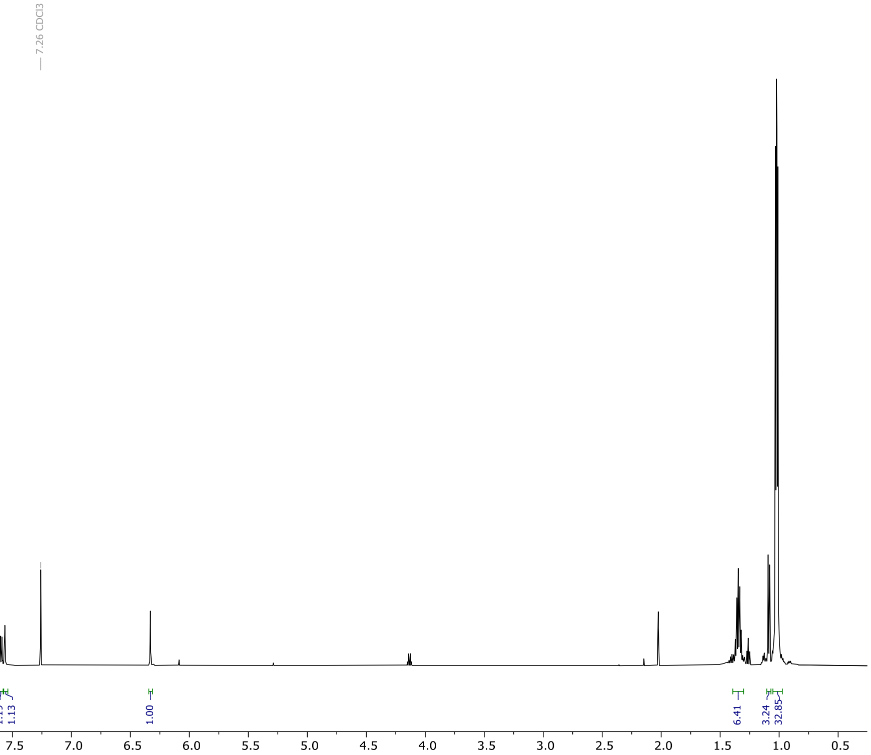

7c: ^{13}C NMR (151 MHz, $CDCl_3$, 353K)

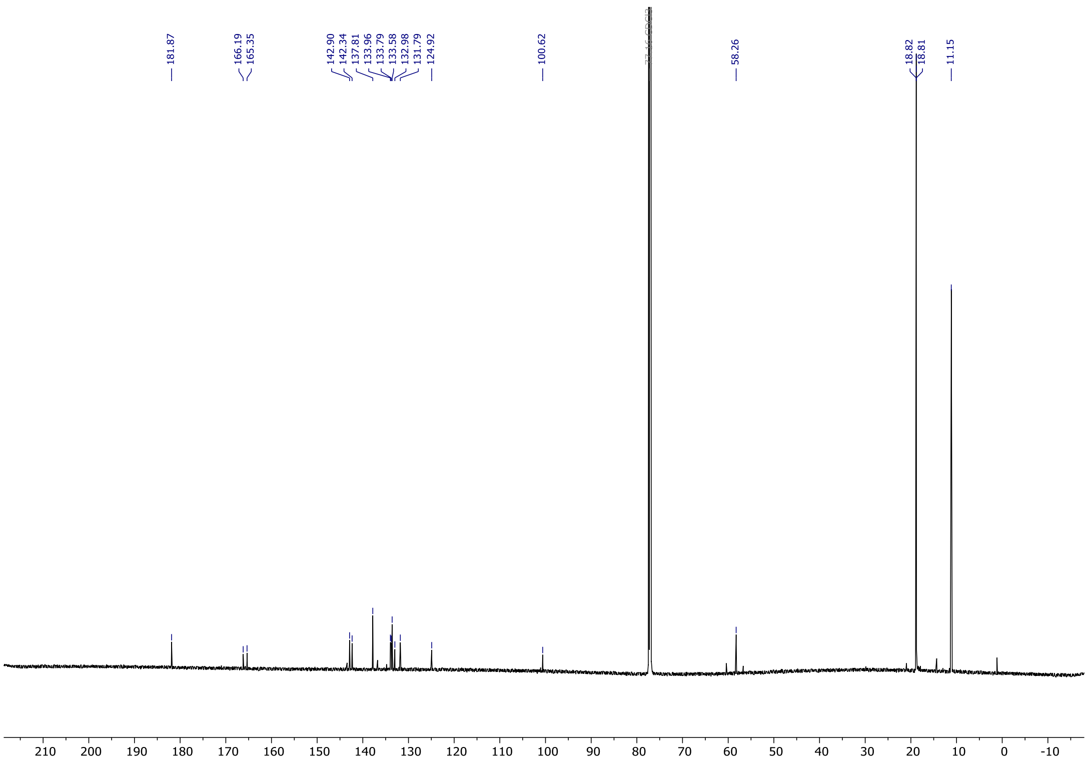

7d: ^{1}H NMR (600 MHz, $CDCl_3$, 353K):

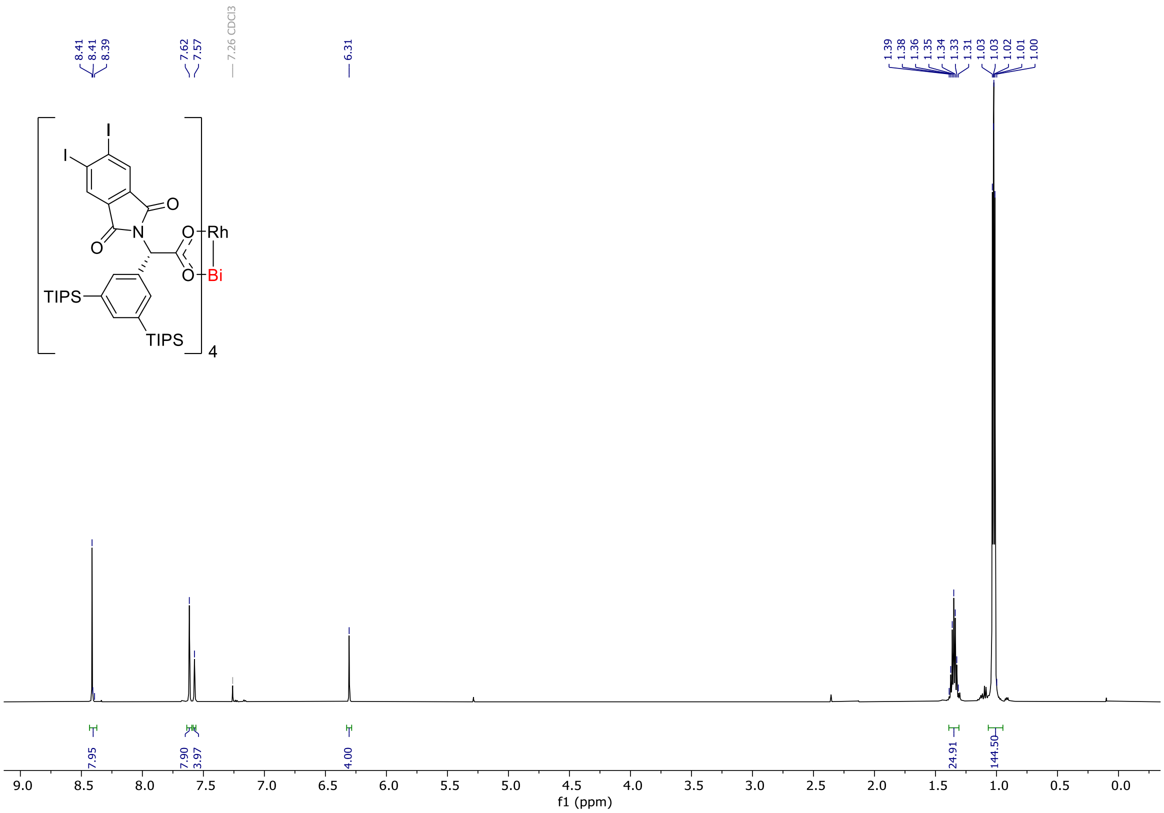

7d: ^{13}C NMR (151 MHz, $CDCl_3$, 353K):

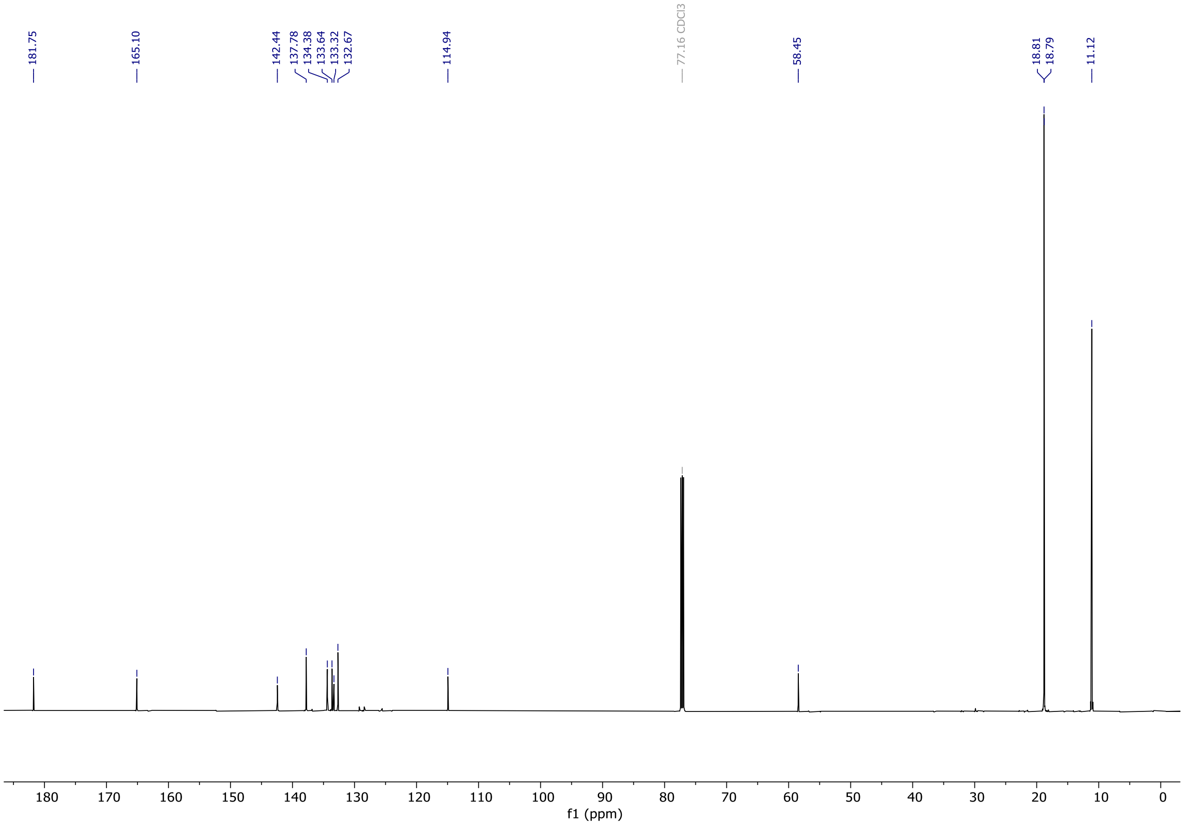

9a: ^{1}H NMR (400 MHz, $CDCl_3$):

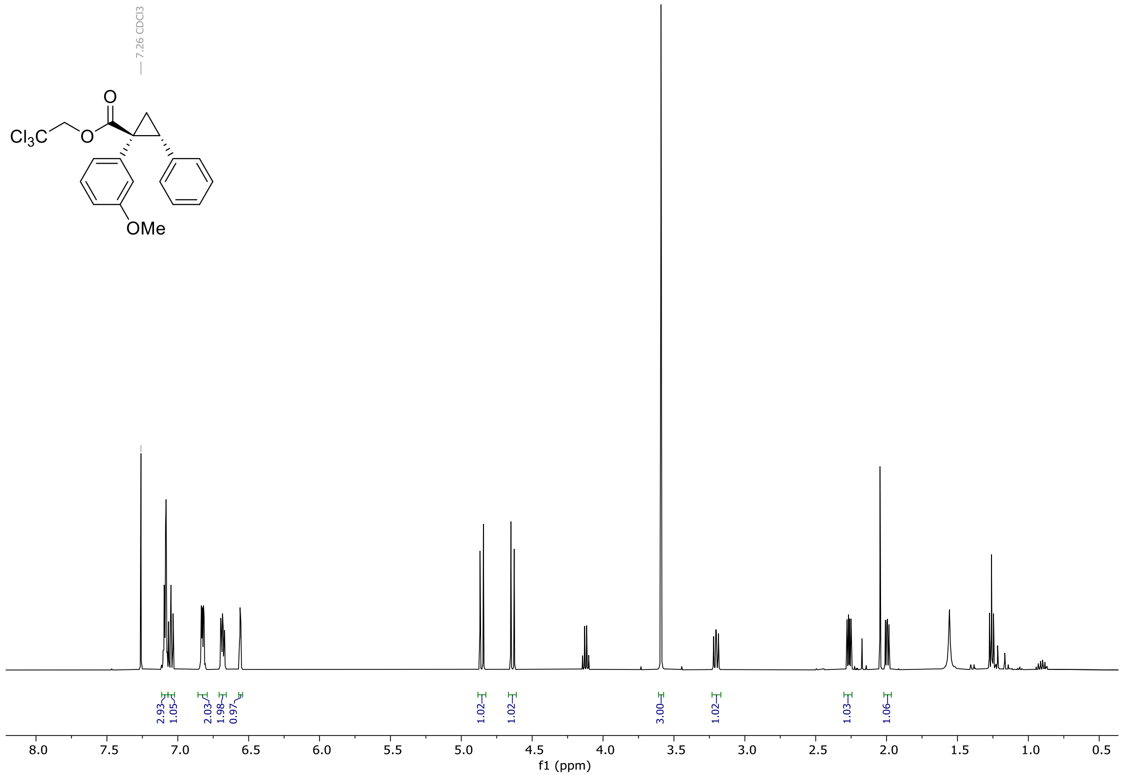

9a: ^{13}C NMR (101 MHz, $CDCl_3$):

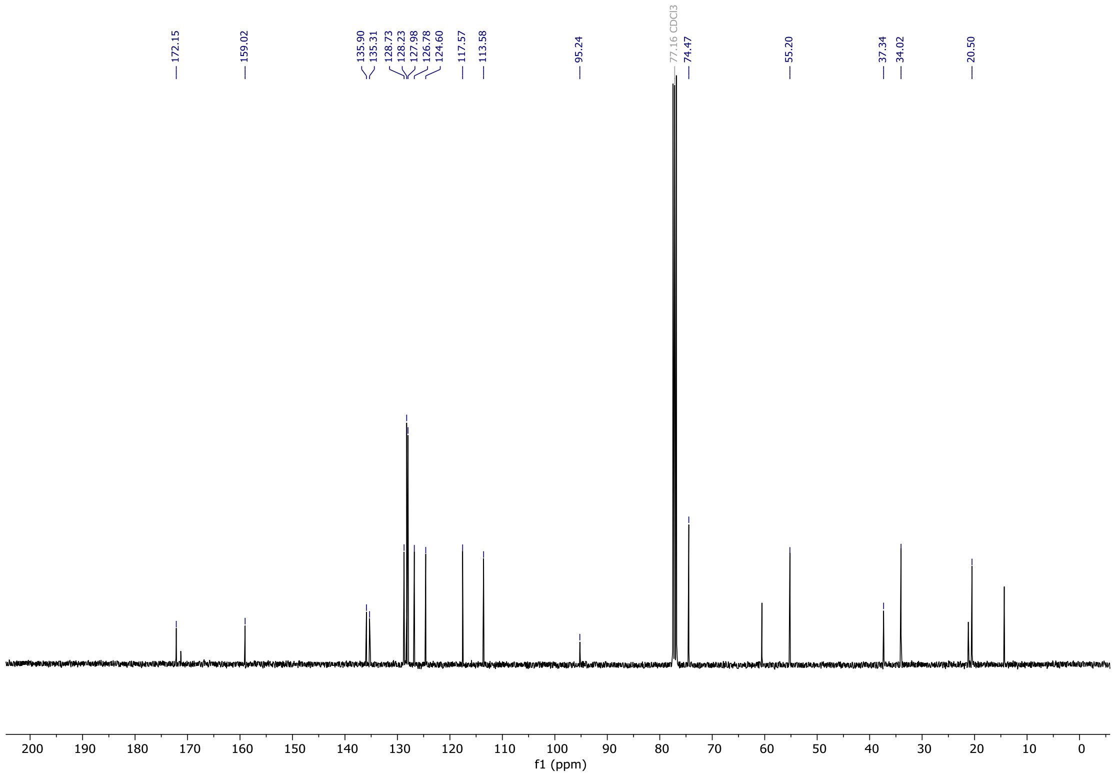

9a: HSQC NMR (400 MHz, 101 MHz, $CDCl_3$):

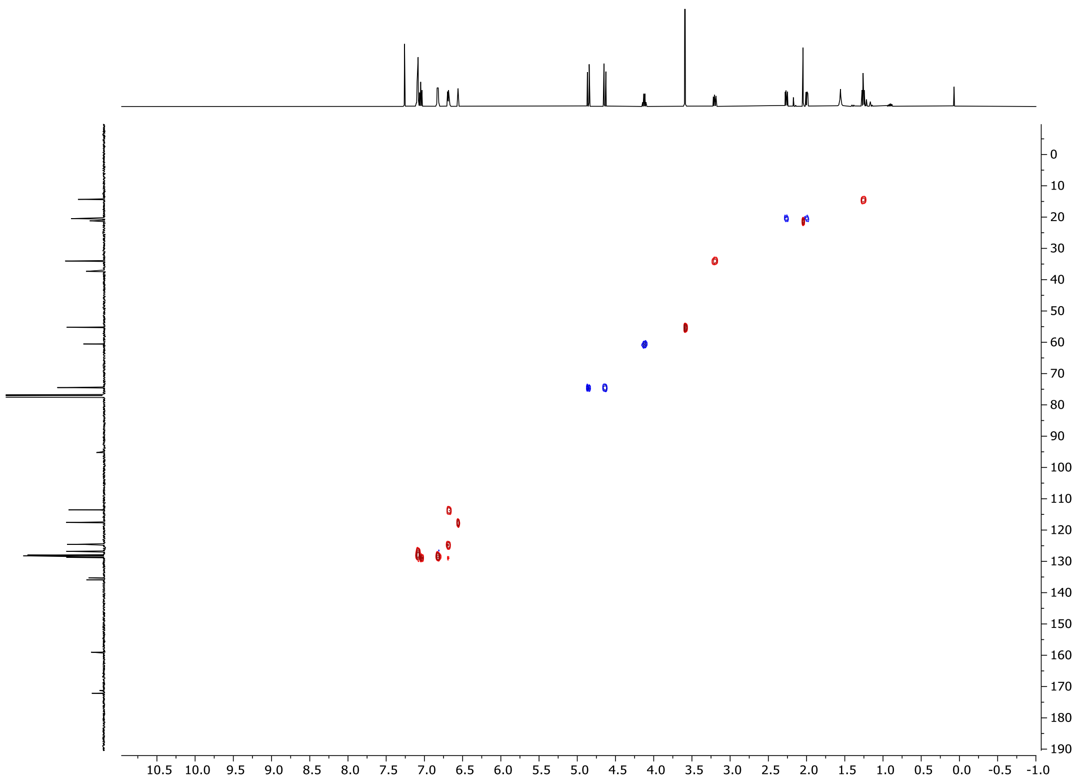

9a: HMBC NMR (400MHz, 101 MHz, $CDCl_3$):

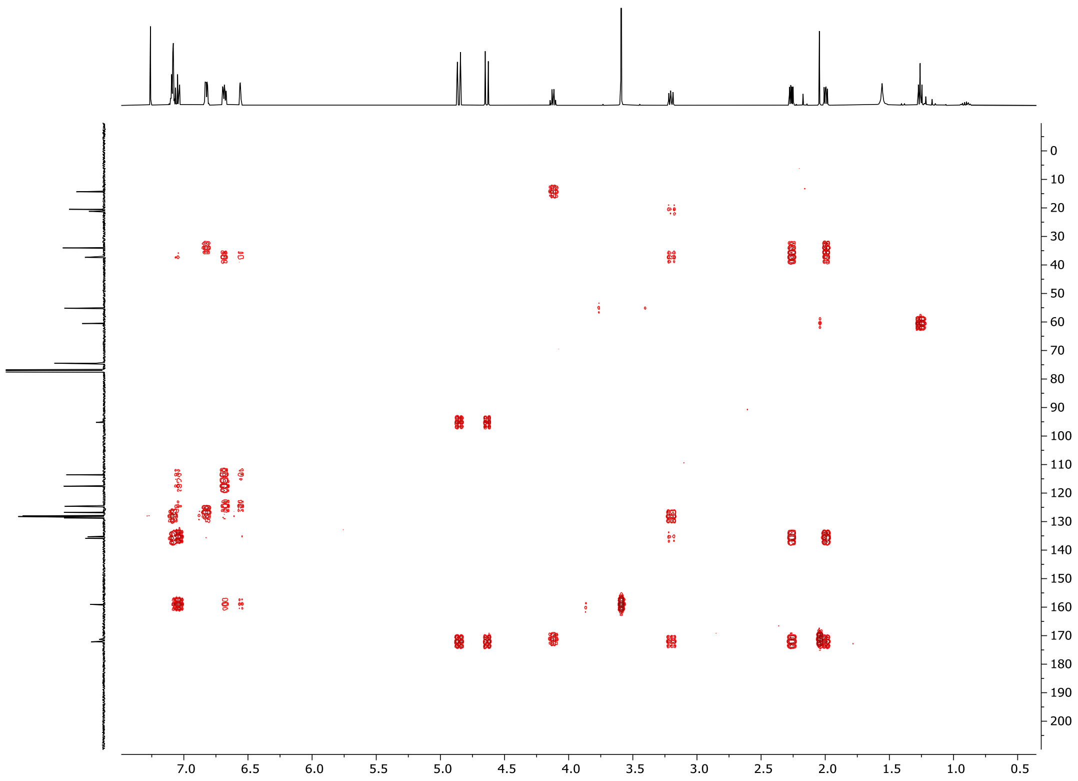

9a: NOESY NMR (500 MHz, $CDCl_3$):

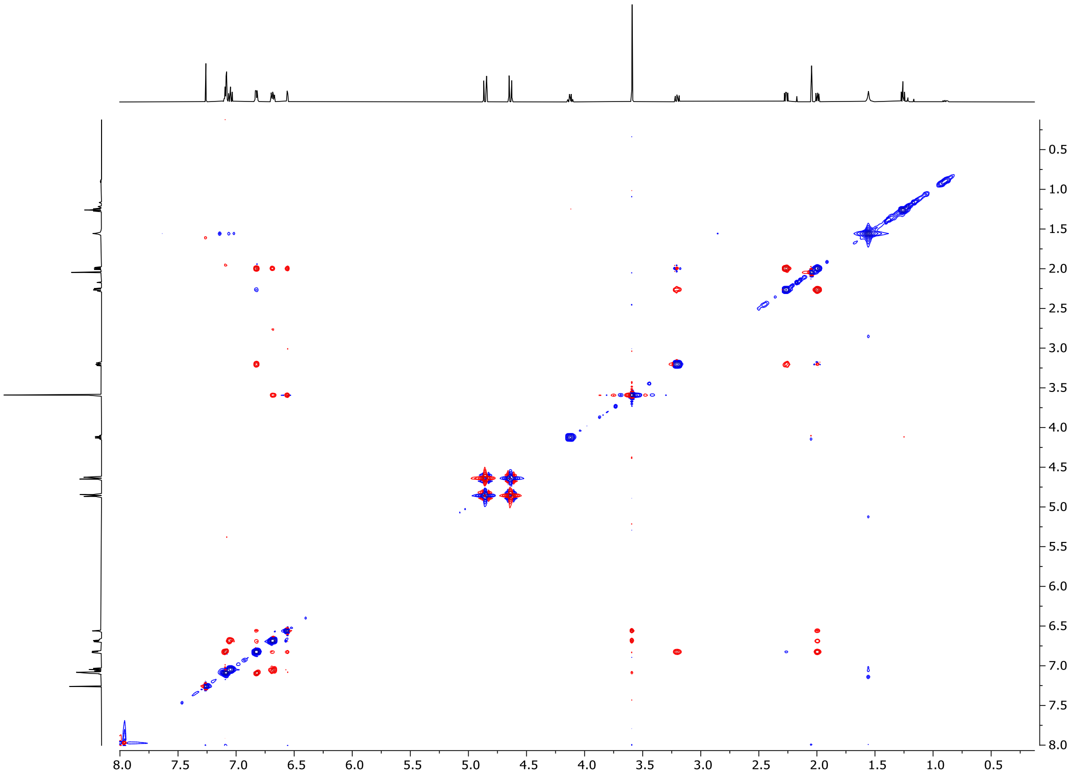

10: ^{1}H NMR (400 MHz, $CDCl_3$):

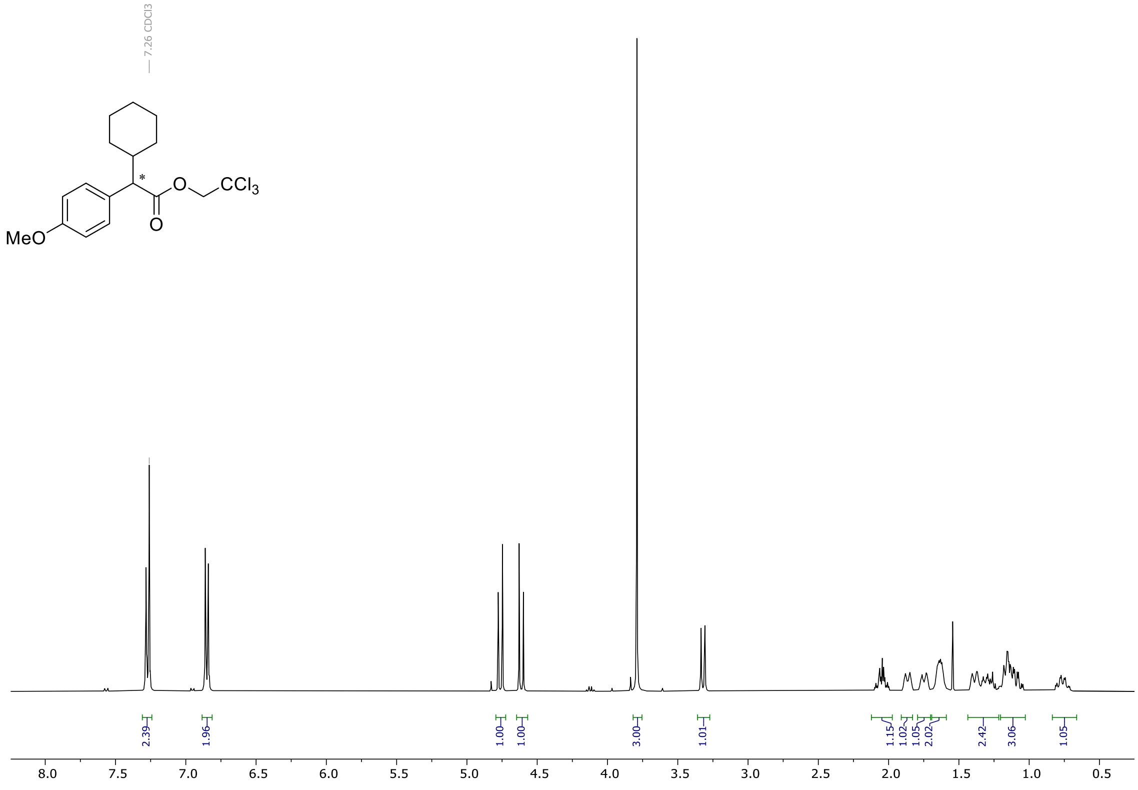

10: ^{13}C NMR (101 MHz, $CDCl_3$):

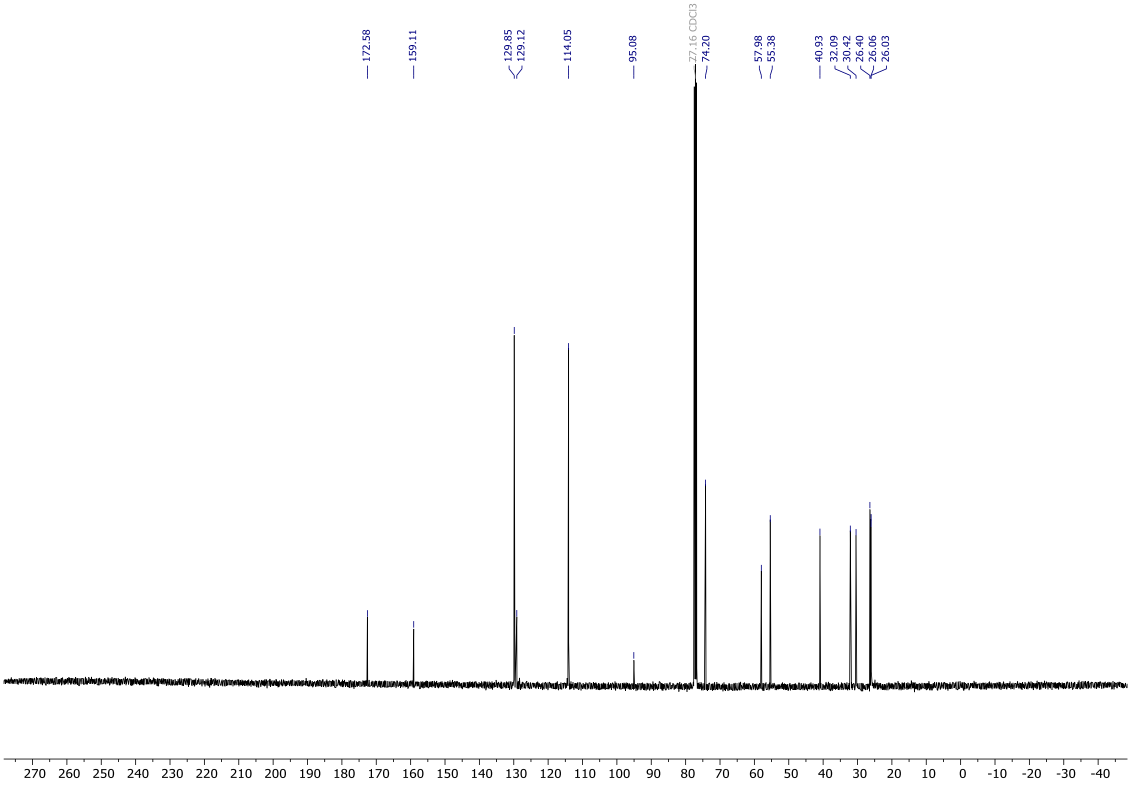

11: ^{1}H NMR (600 MHz, $CDCl_3$)

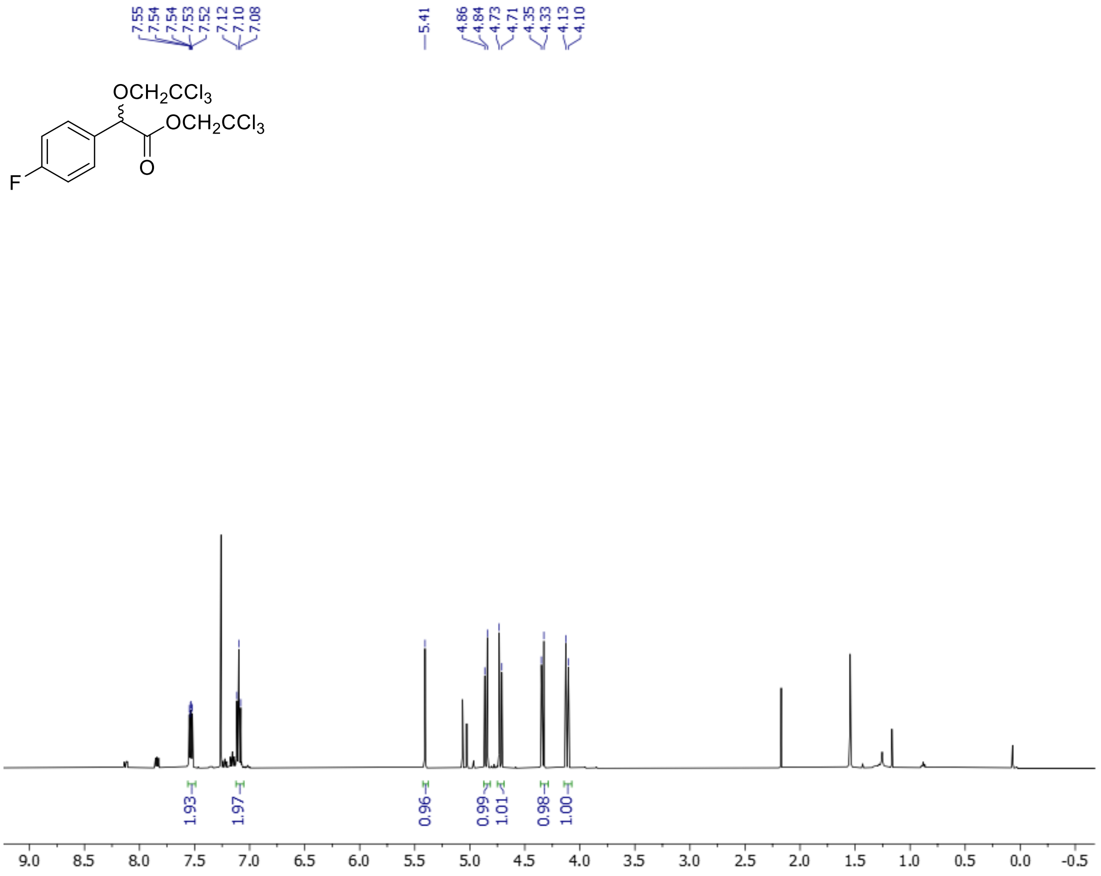

11: ^{13}C NMR (151 MHz, $CDCl_3$)

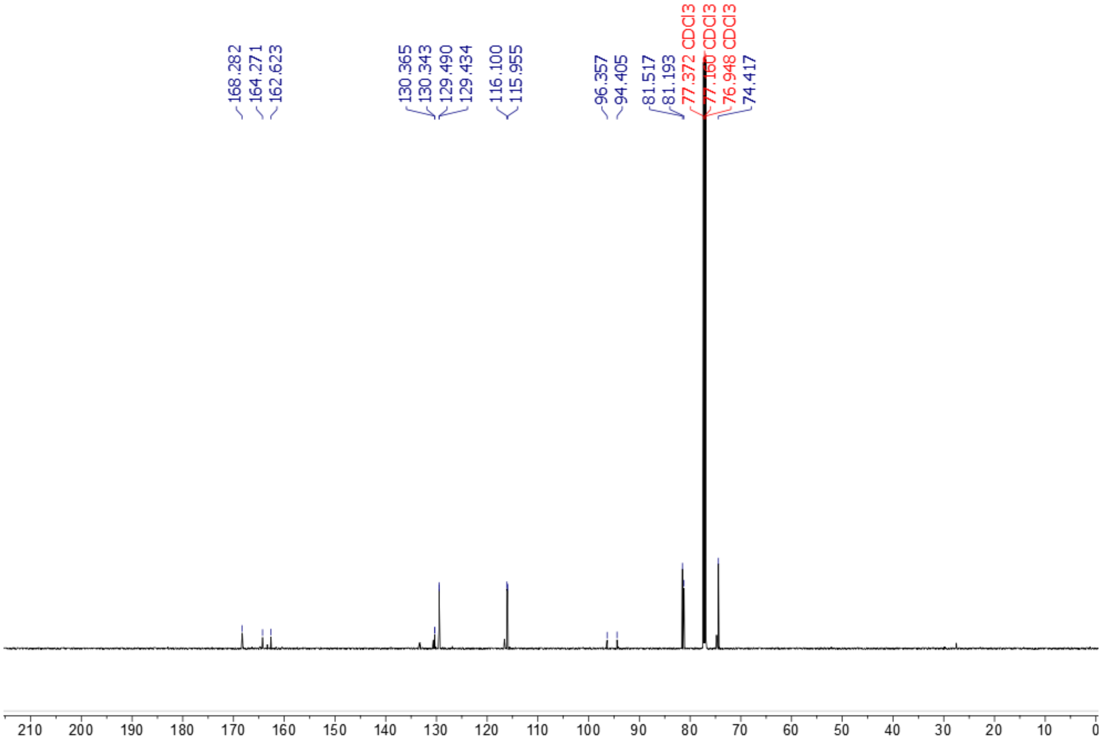

11: ^{19}F NMR (470 MHz, $CDCl_3$)

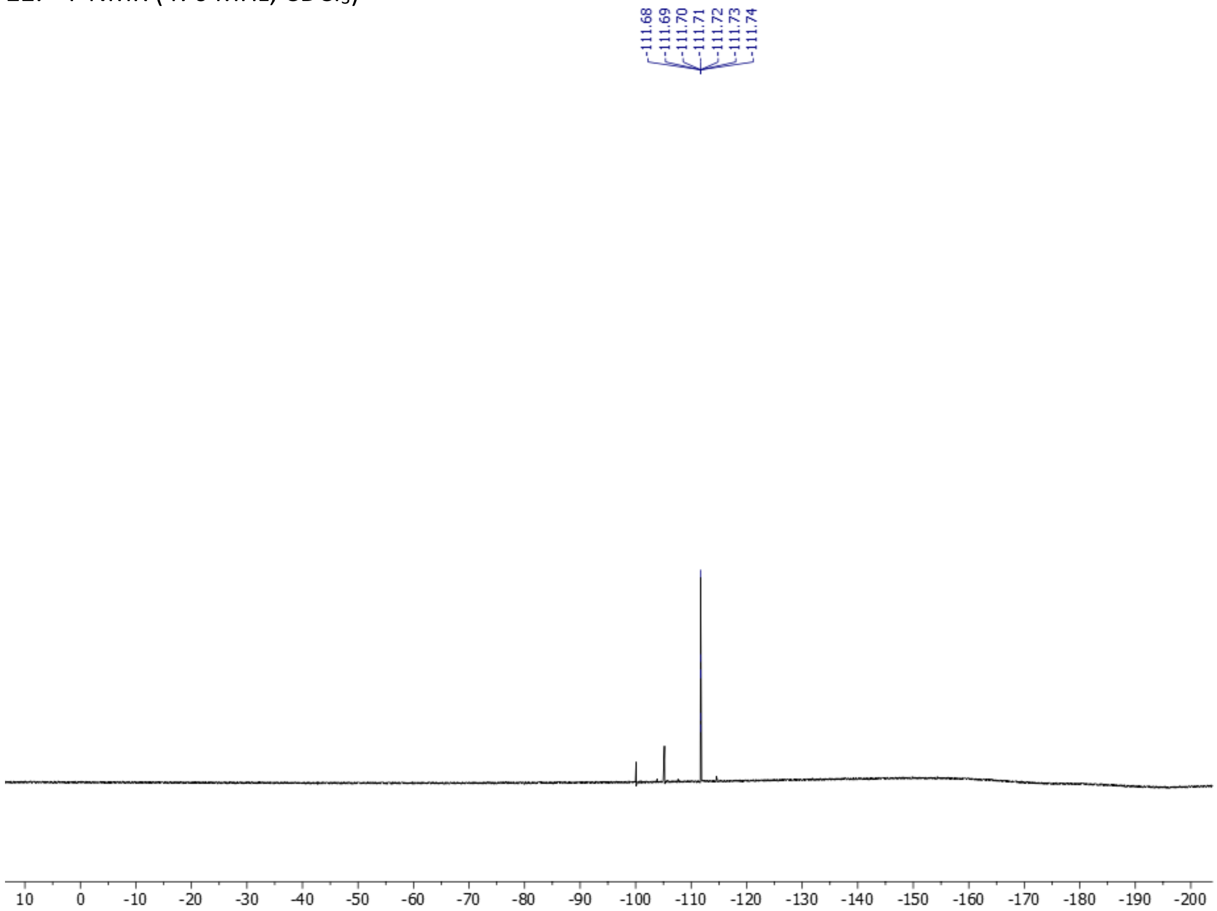

12: ^{1}H NMR (400 MHz, $CDCl_3$)

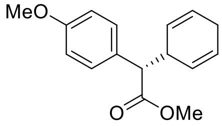

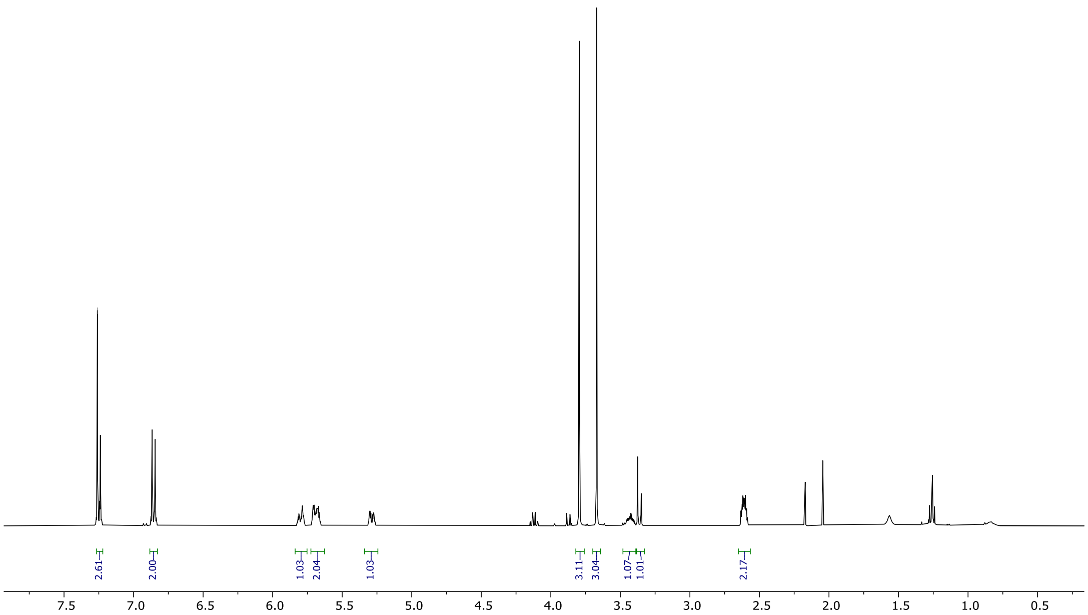

12: ^{13}C NMR (101 MHz, $CDCl_3$)

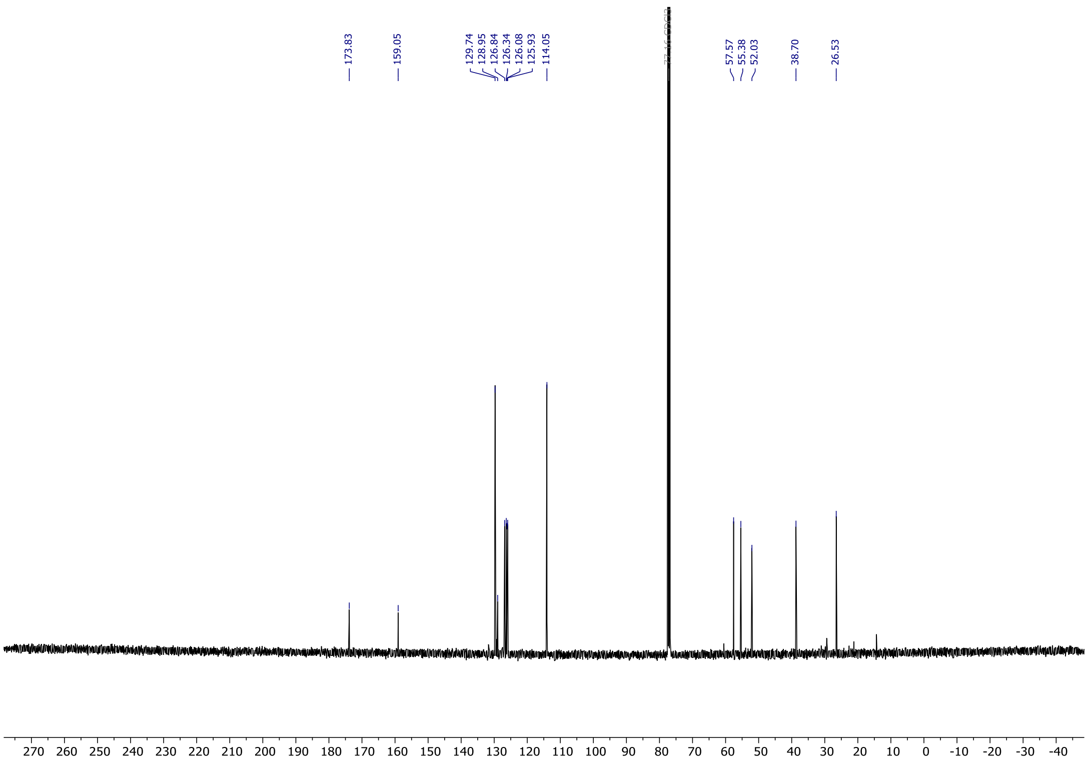

13: ^{1}H NMR (400 MHz, $CDCl_3$)

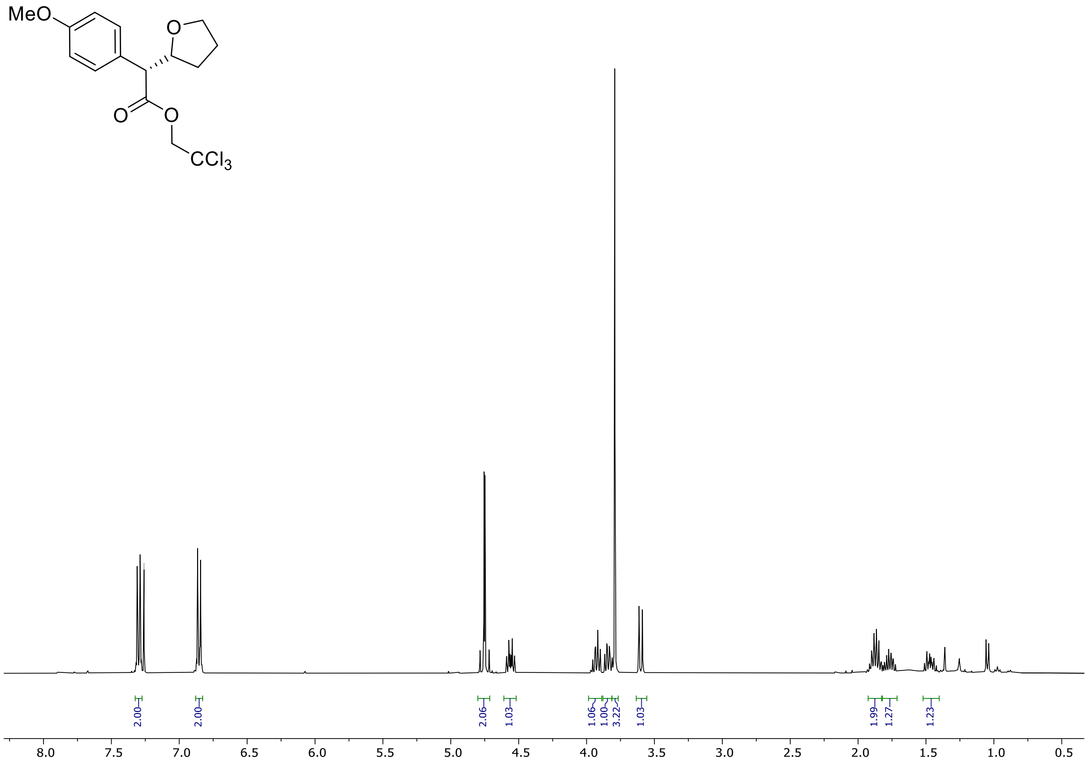

13: ^{13}C NMR (101 MHz, $CDCl_3$)

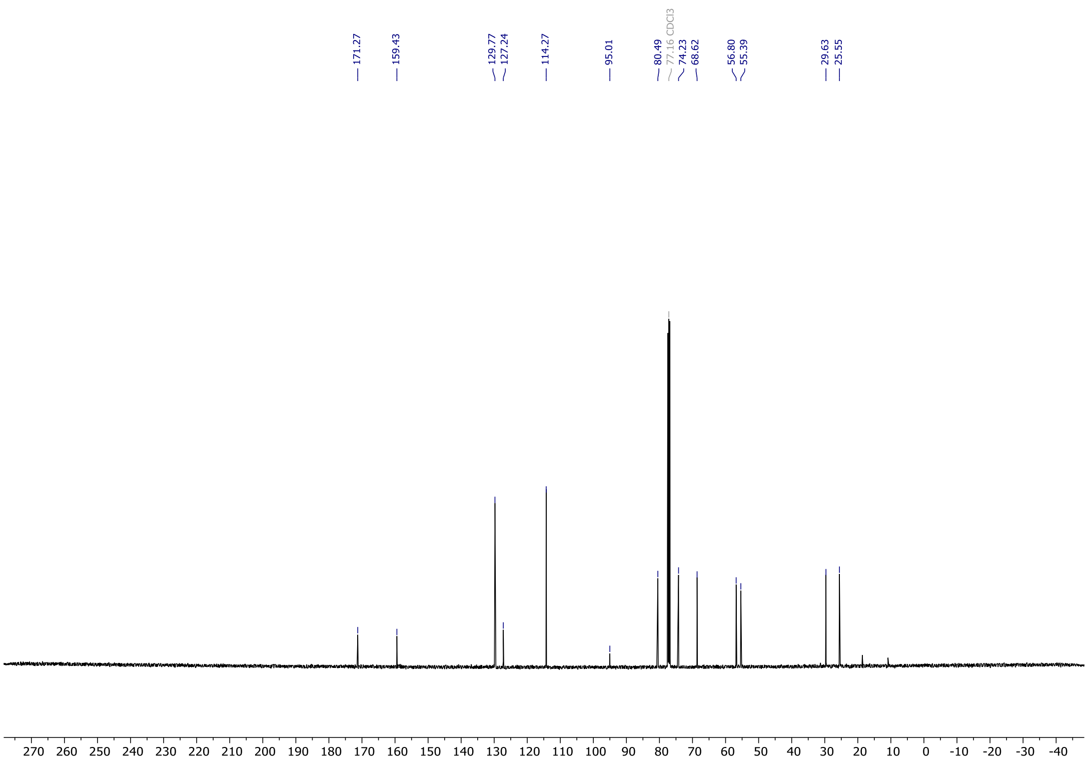

14: ^{1}H NMR (400 MHz, $CDCl_3$)

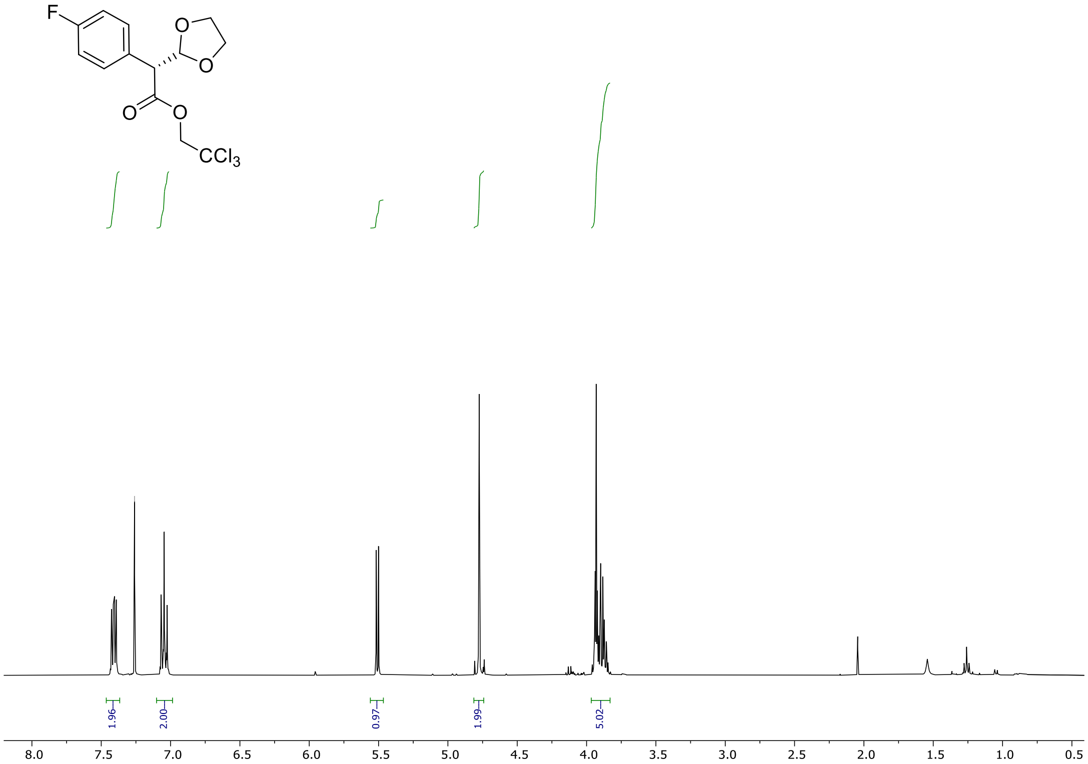

14: ^{13}C NMR (101 MHz, $CDCl_3$)

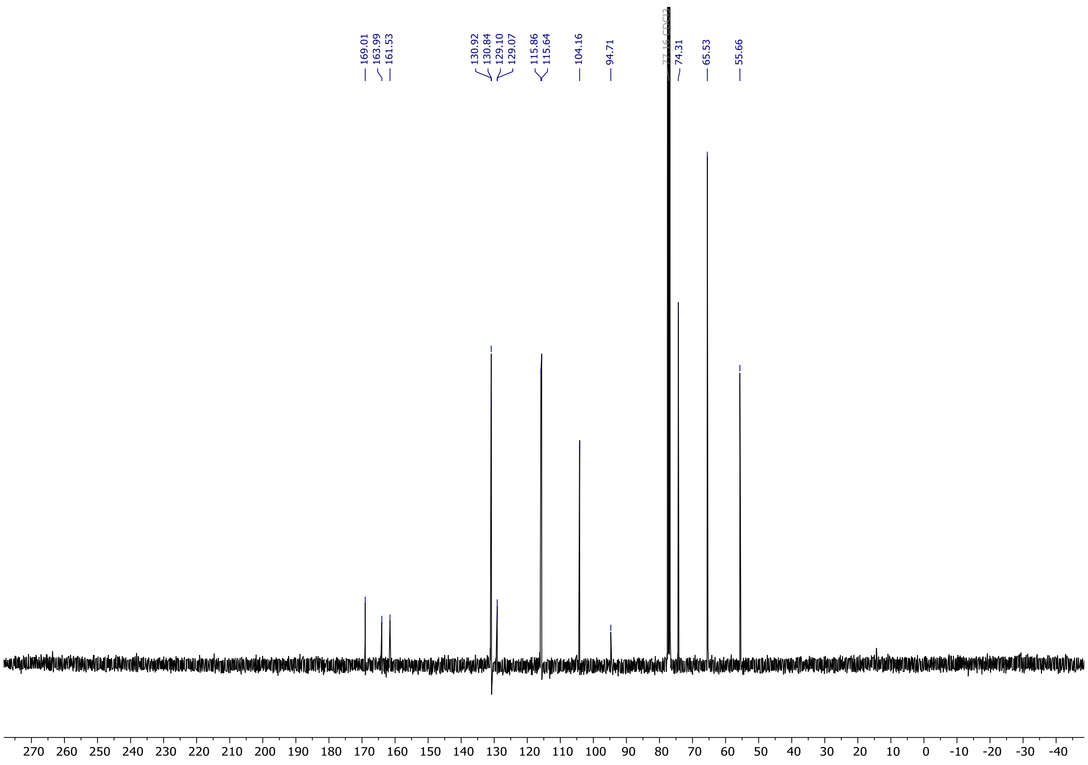

14: ^{19}F NMR (282 MHz, $CDCl_3$)

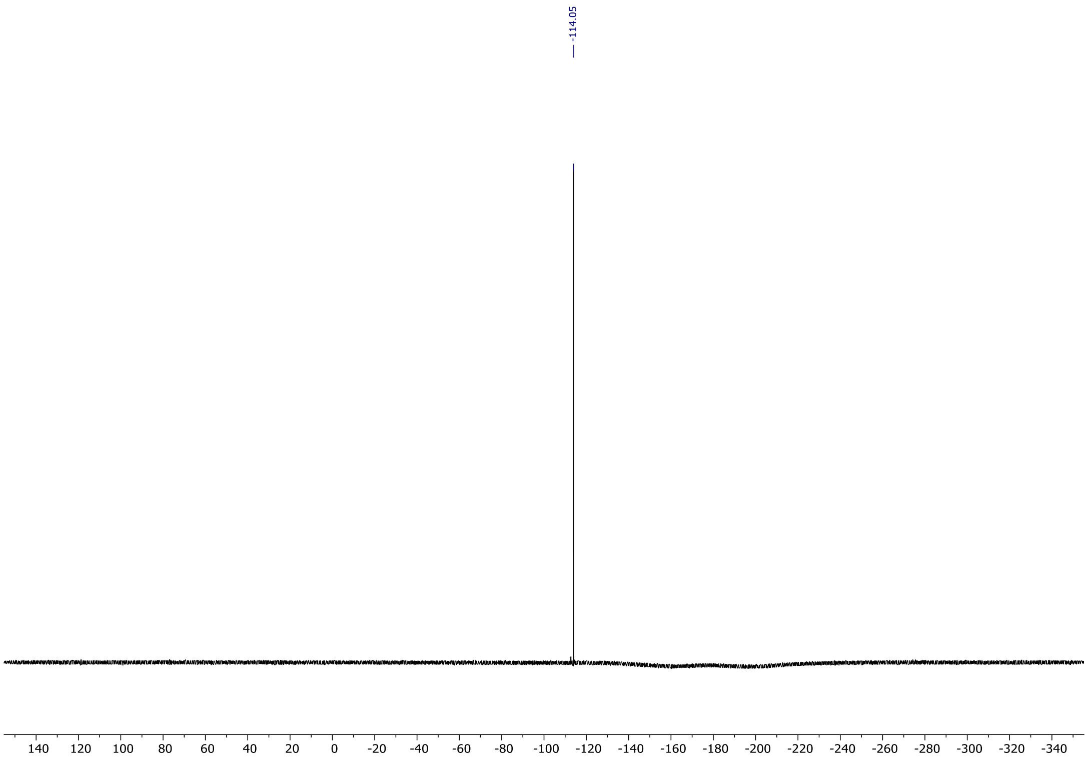

15: ^{1}H NMR (400 MHz, $CDCl_3$):

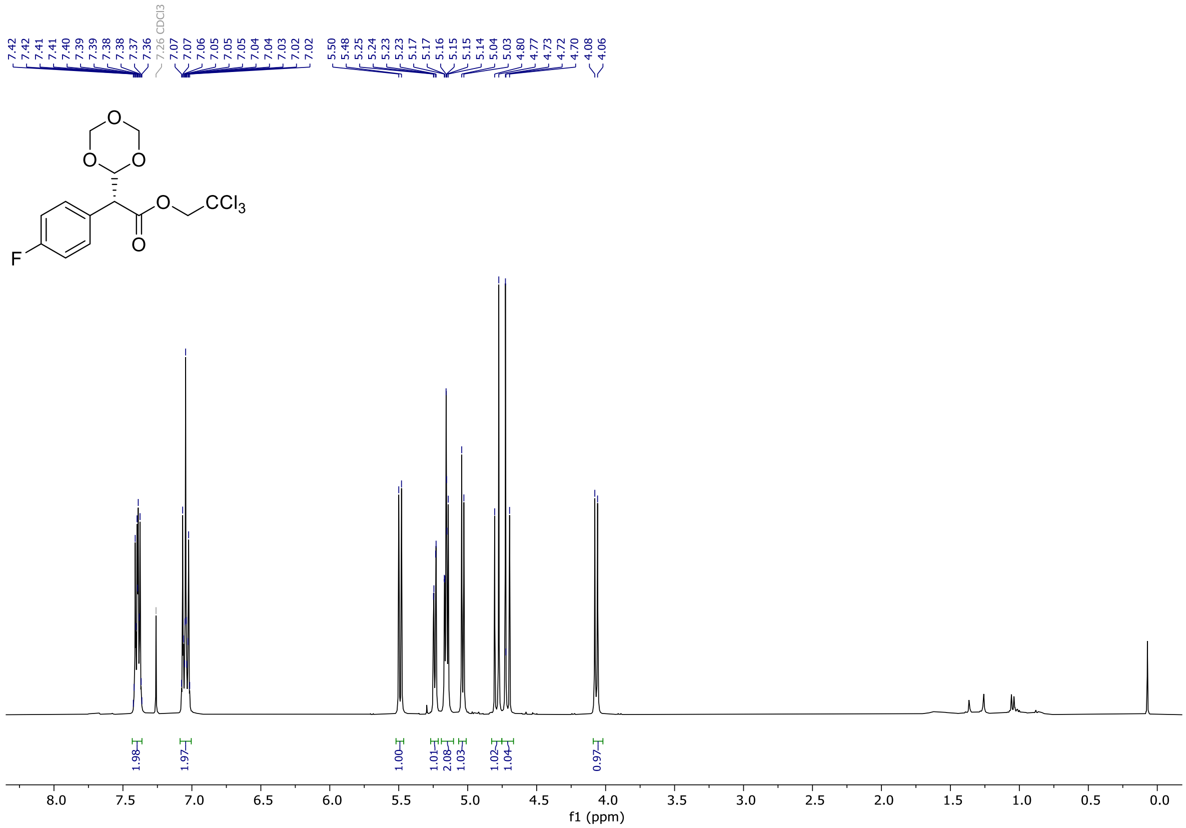

15: ^{13}C NMR (101 MHz, $CDCl_3$):

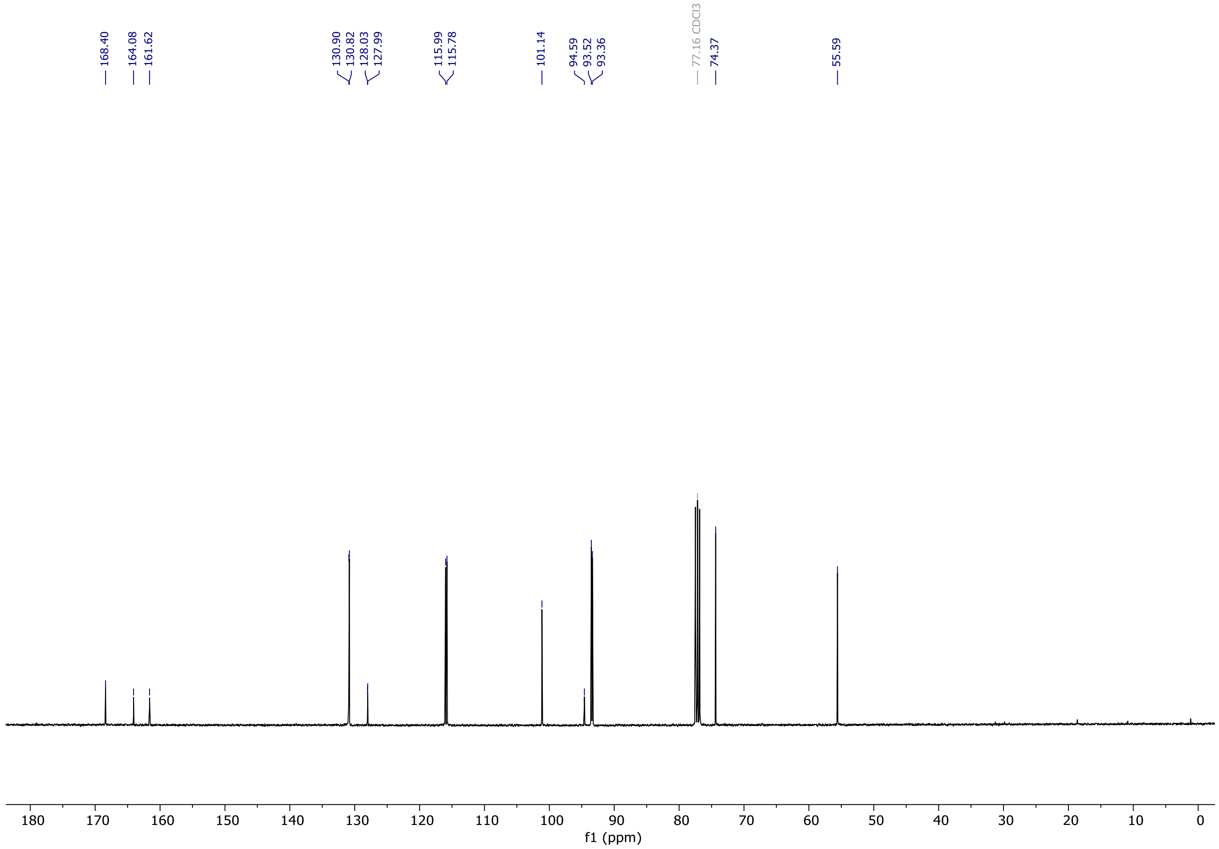

15: ^{19}F NMR (282 MHz, $CDCl_3$):

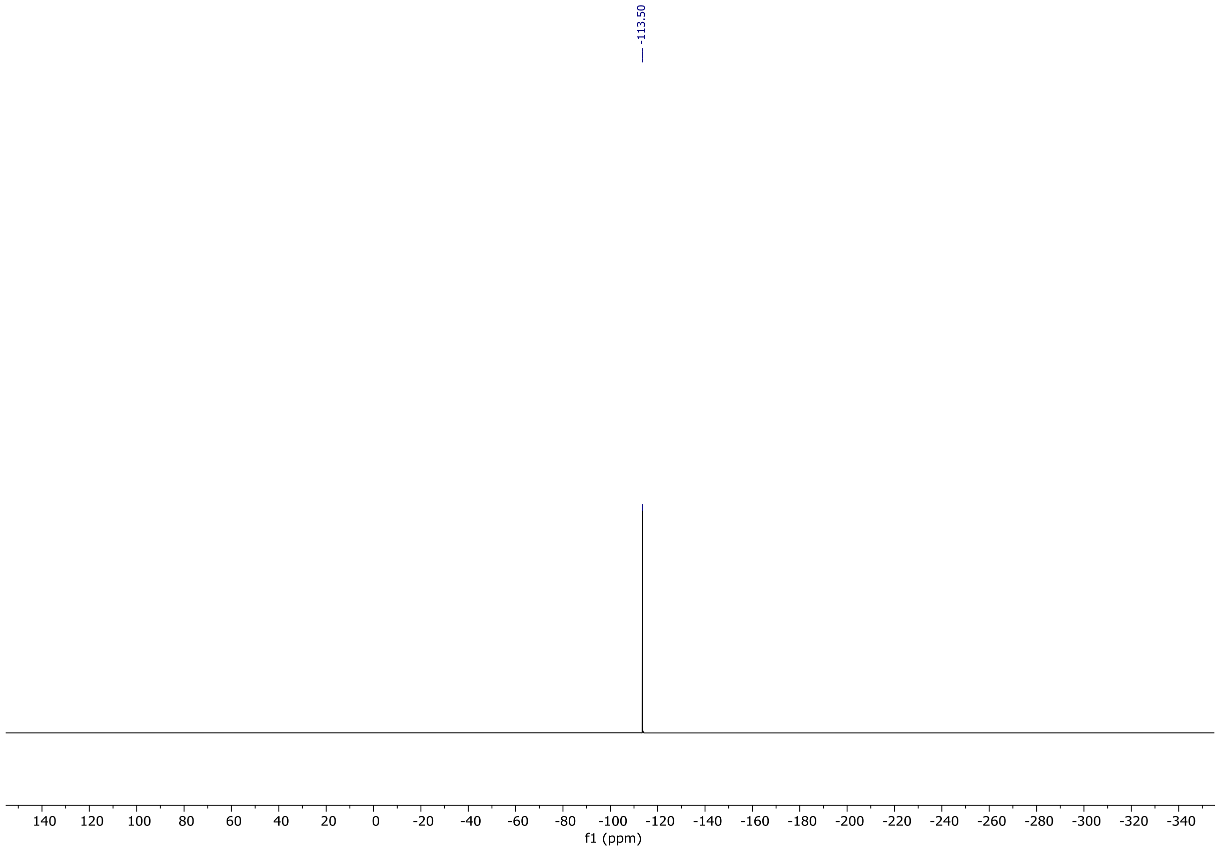

16: ^{1}H NMR (400 MHz, $CDCl_3$)

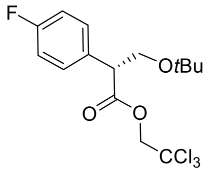

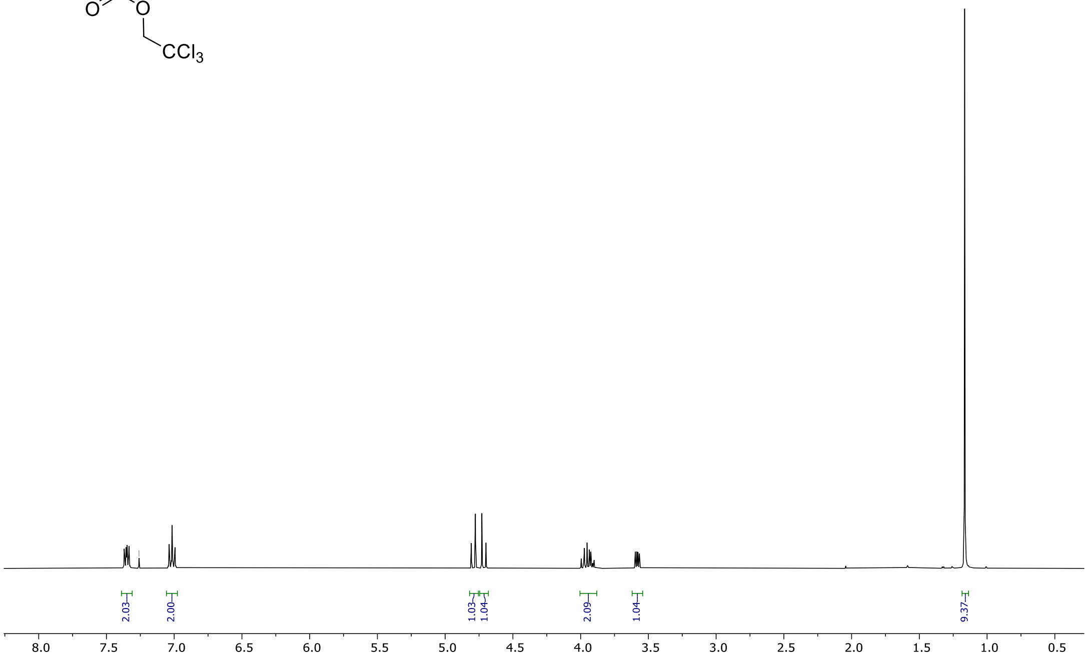

16: ^{13}C NMR (101 MHz, $CDCl_3$)

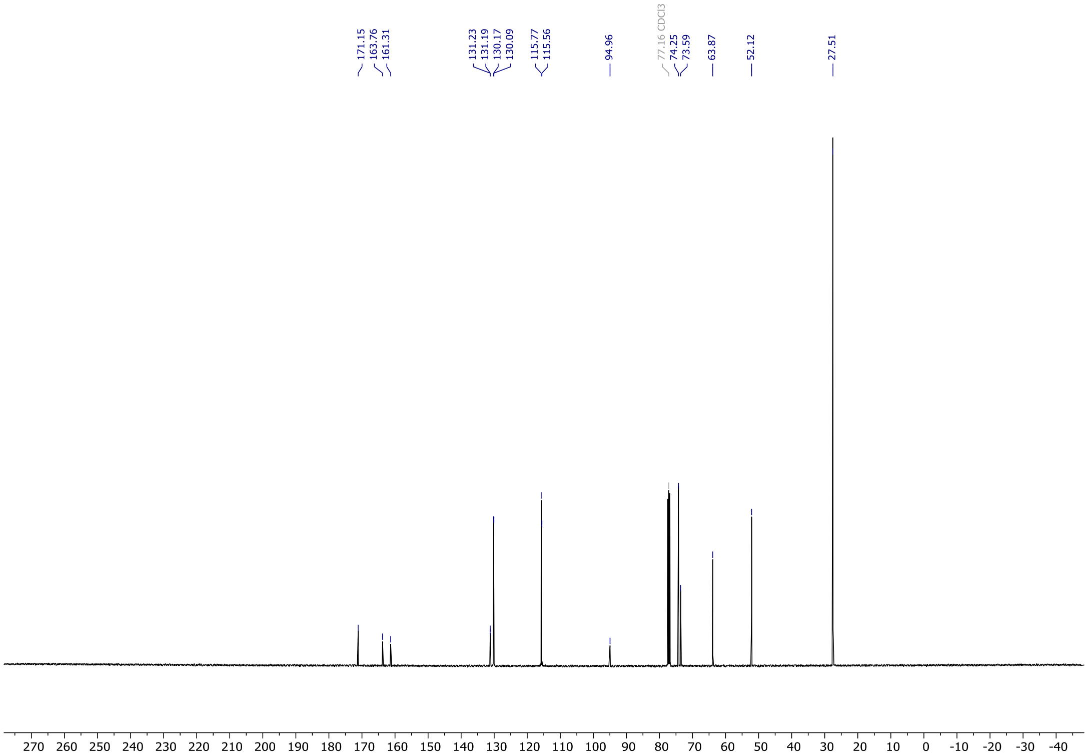

16: ^{19}F NMR (282 MHz, $CDCl_3$)

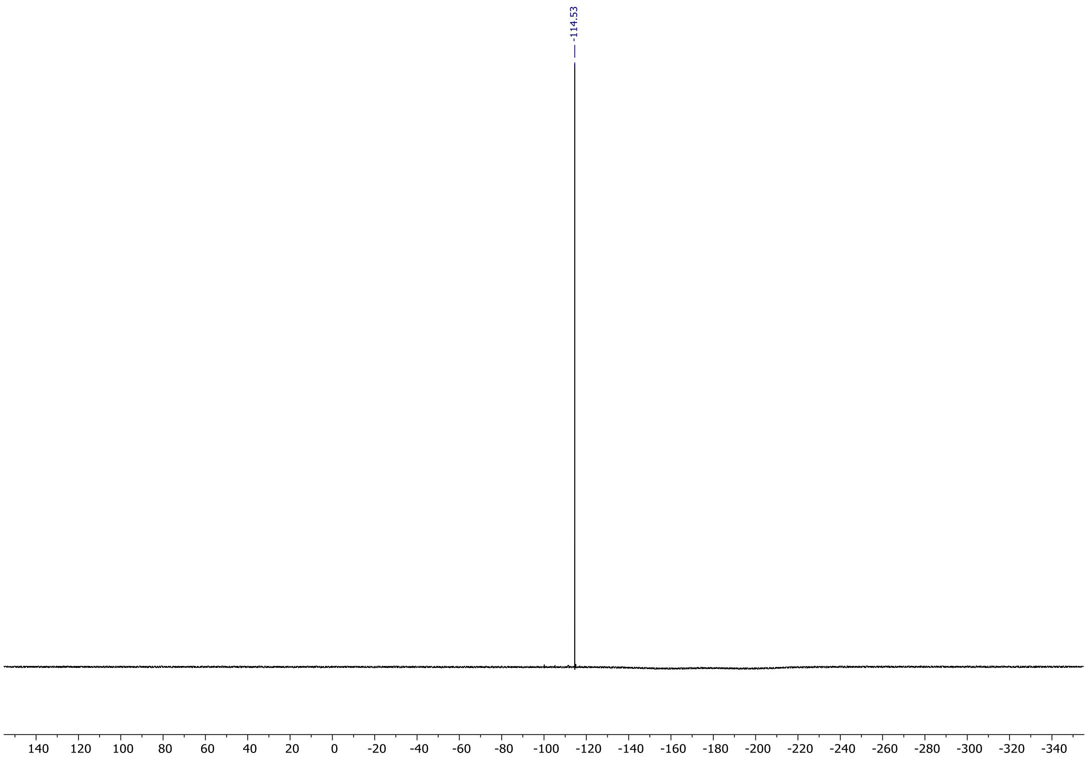

17: ^{1}H NMR (400 MHz, $CDCl_3$)

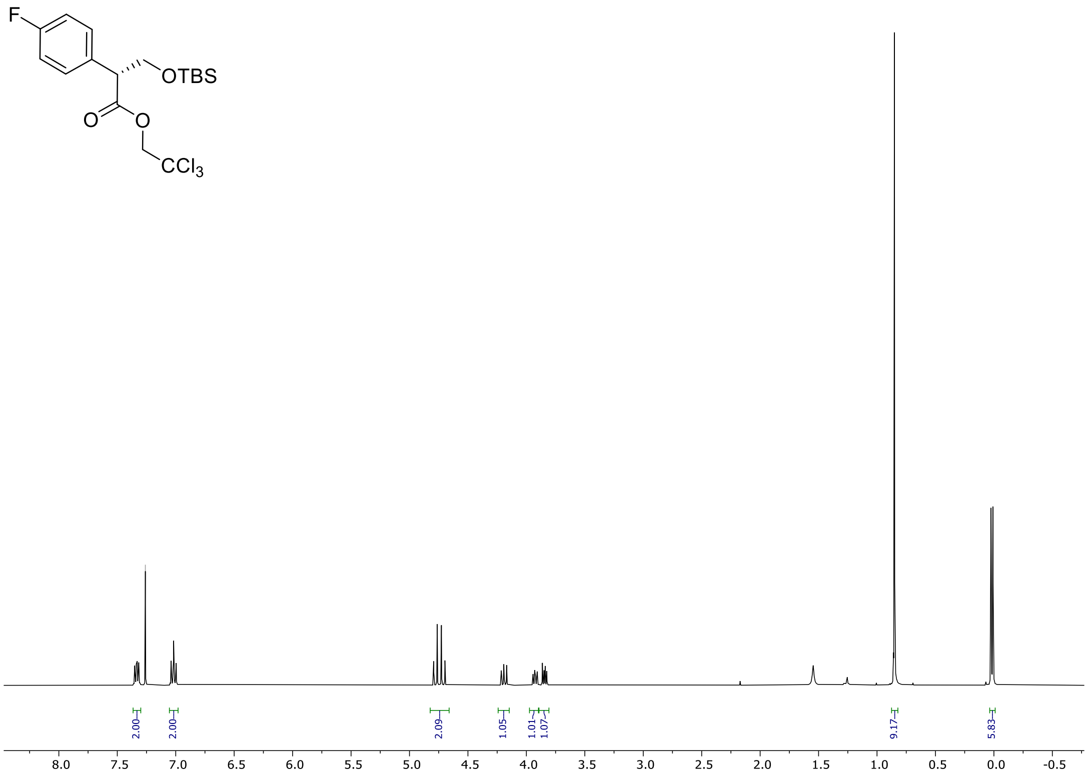

17: ^{13}C NMR (101 MHz, $CDCl_3$)

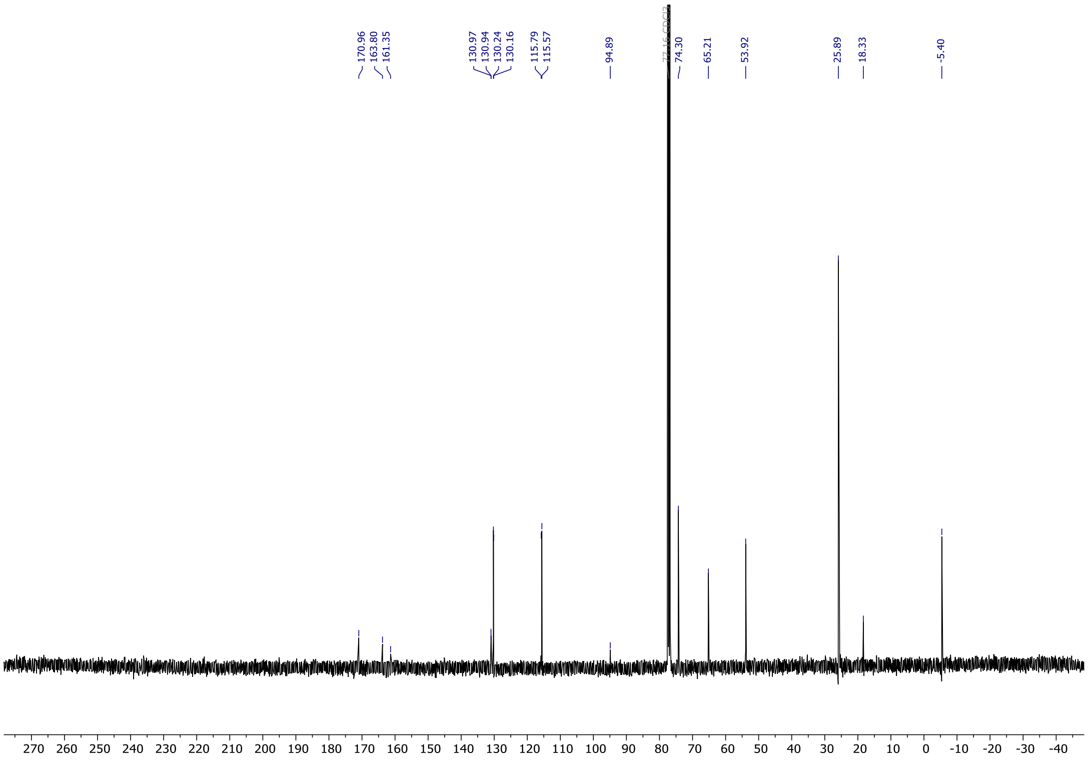

17: ^{19}F NMR (282 MHz, $CDCl_3$)

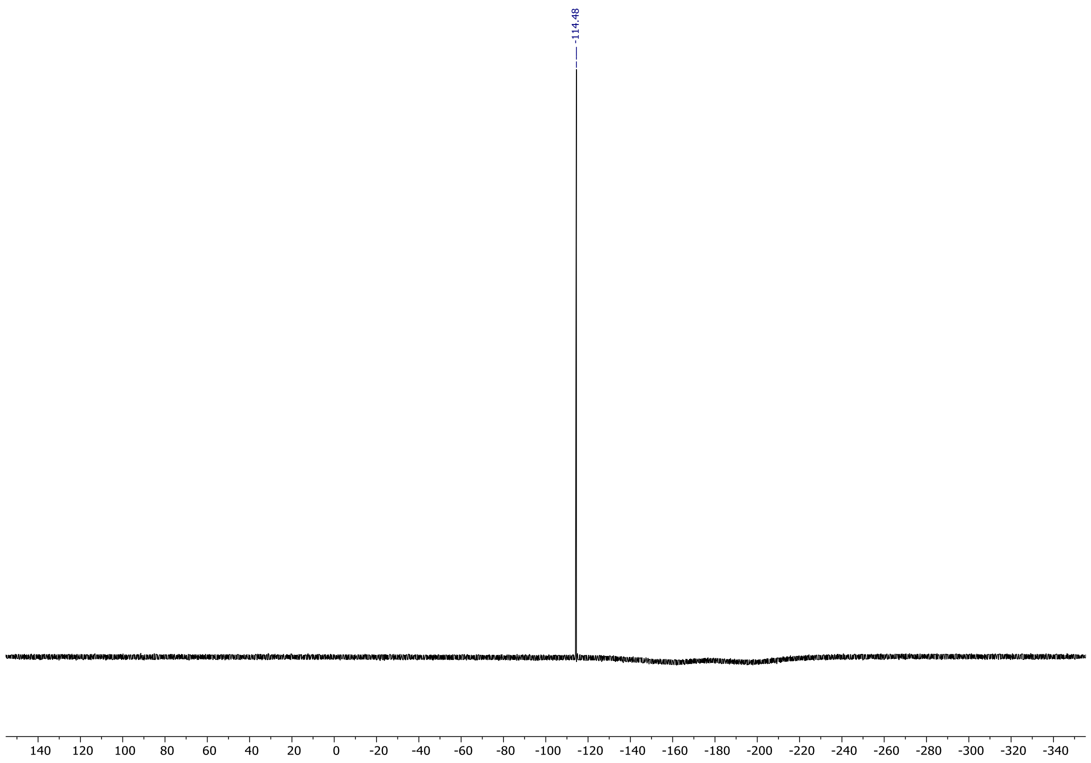

18: ^{1}H NMR (400 MHz, $CDCl_3$)

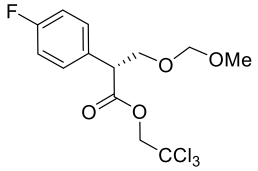

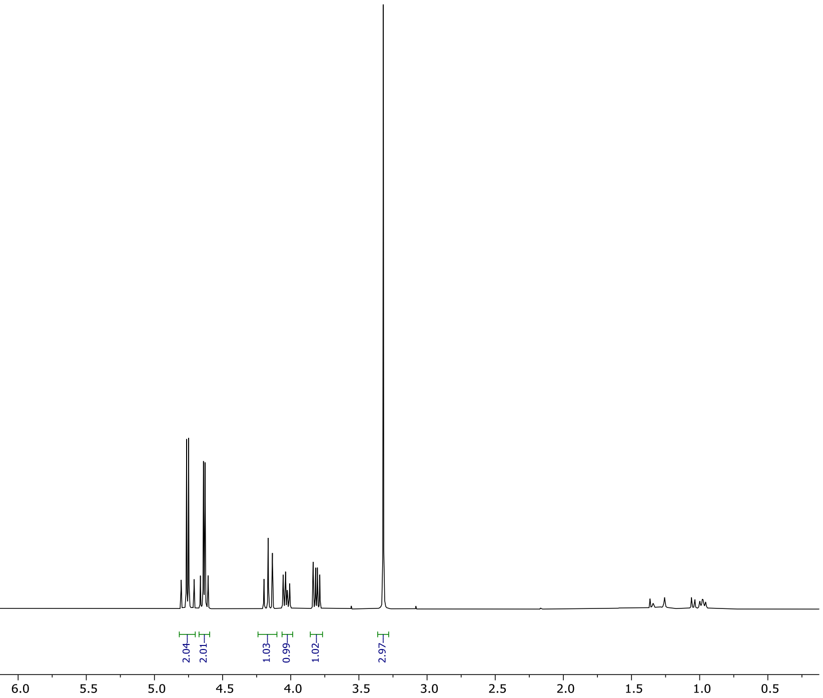

18: ^{13}C NMR (101 MHz, $CDCl_3$)

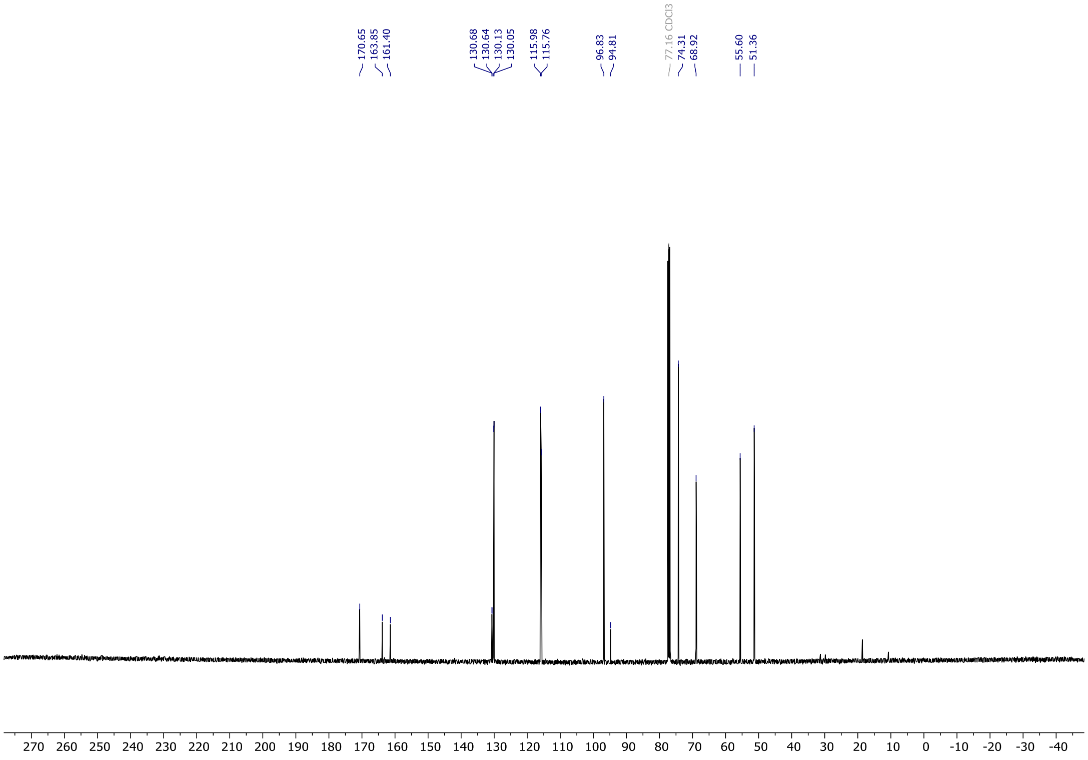

18: ^{19}F NMR (282 MHz, $CDCl_3$)

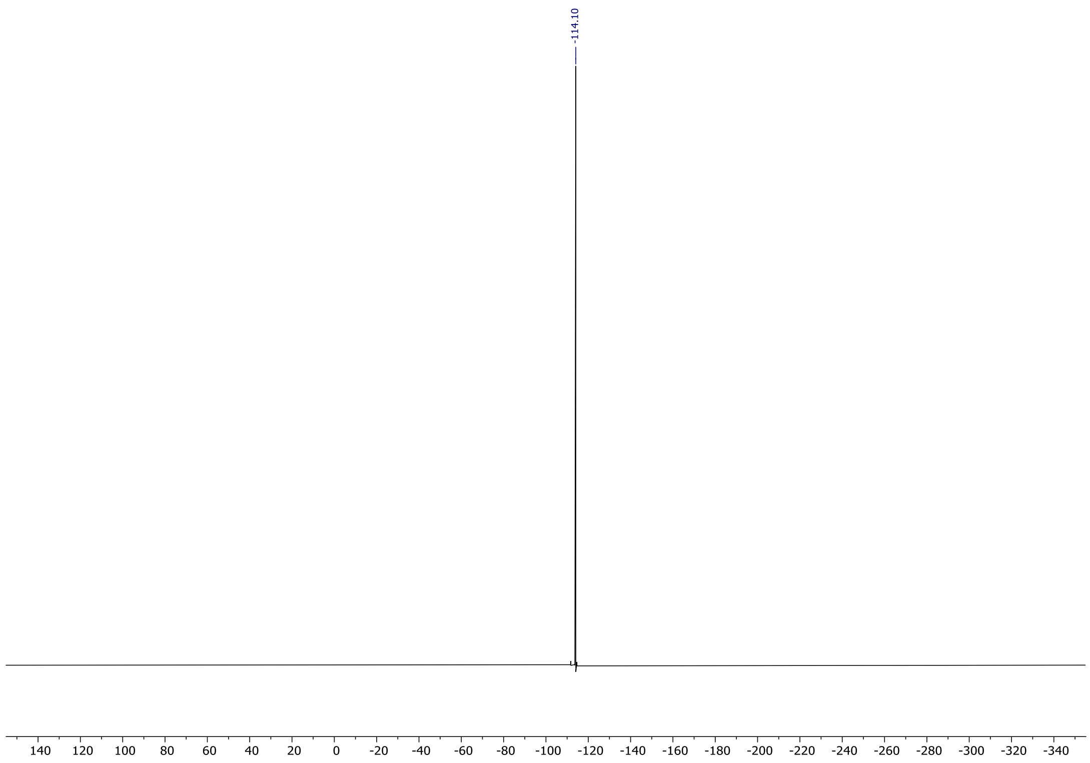

19: ^{1}H NMR (400 MHz, $CDCl_3$)

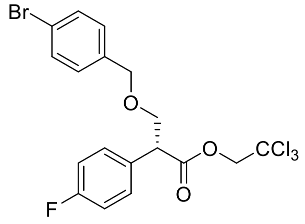

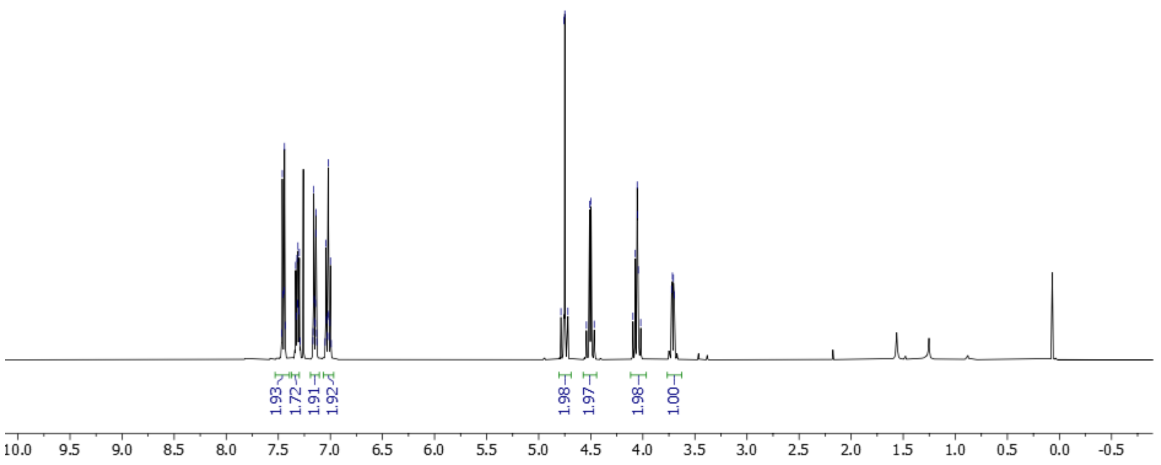

19: ^{13}C NMR (101 MHz, $CDCl_3$)

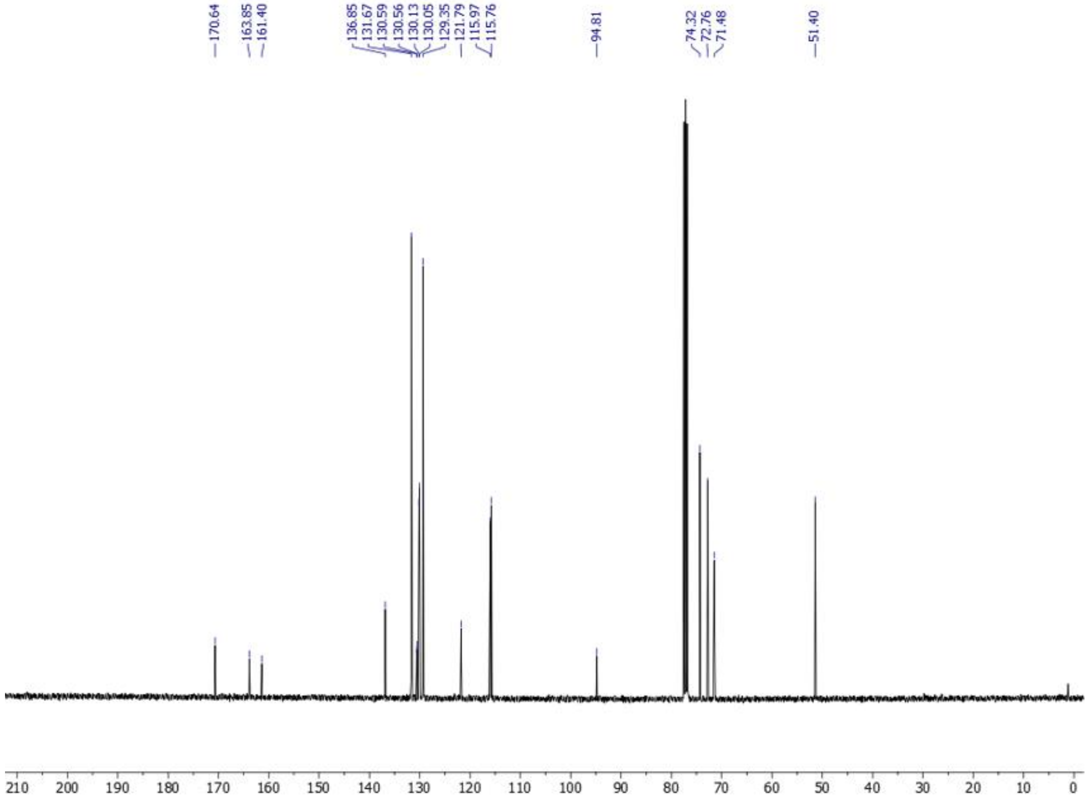

19: ^{19}F NMR (282 MHz, $CDCl_3$)

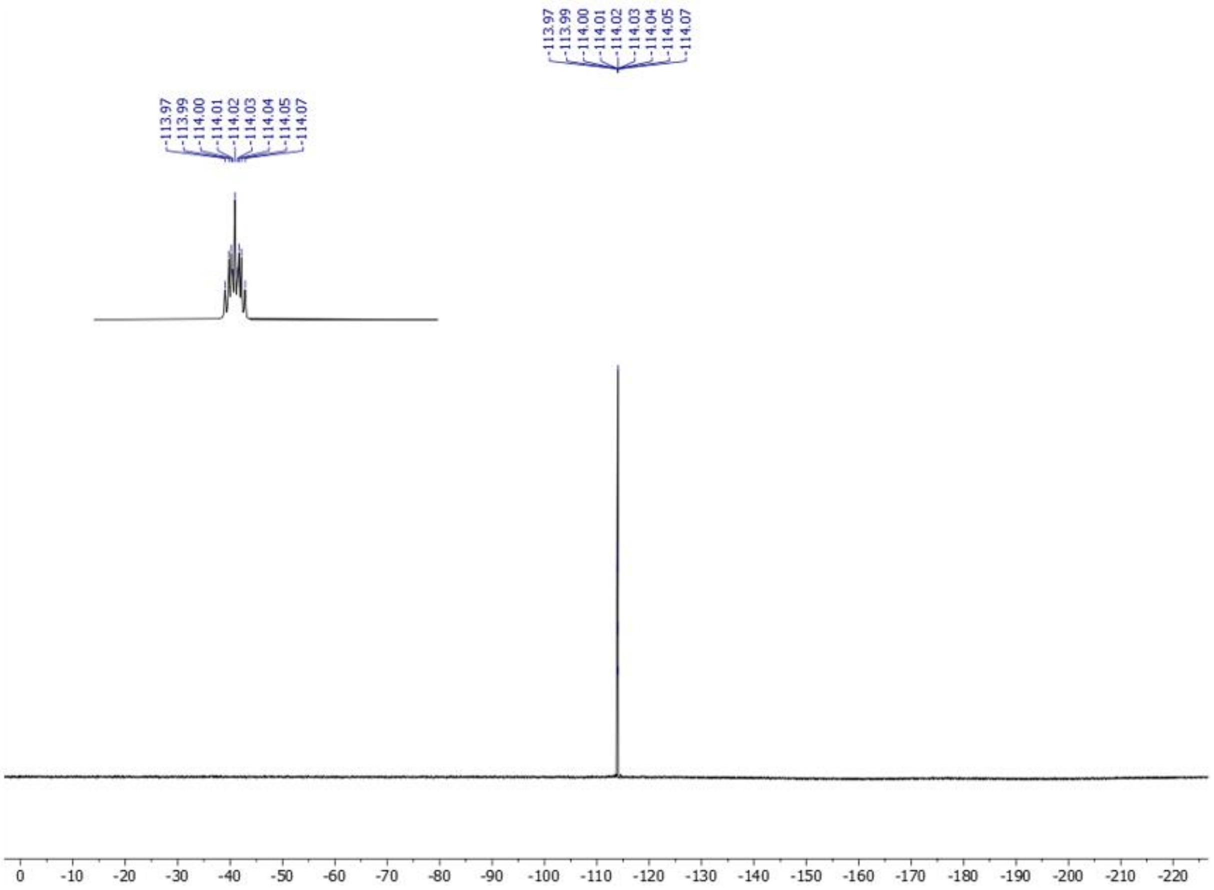

20a: ^{1}H NMR (400 MHz, $CDCl_3$)

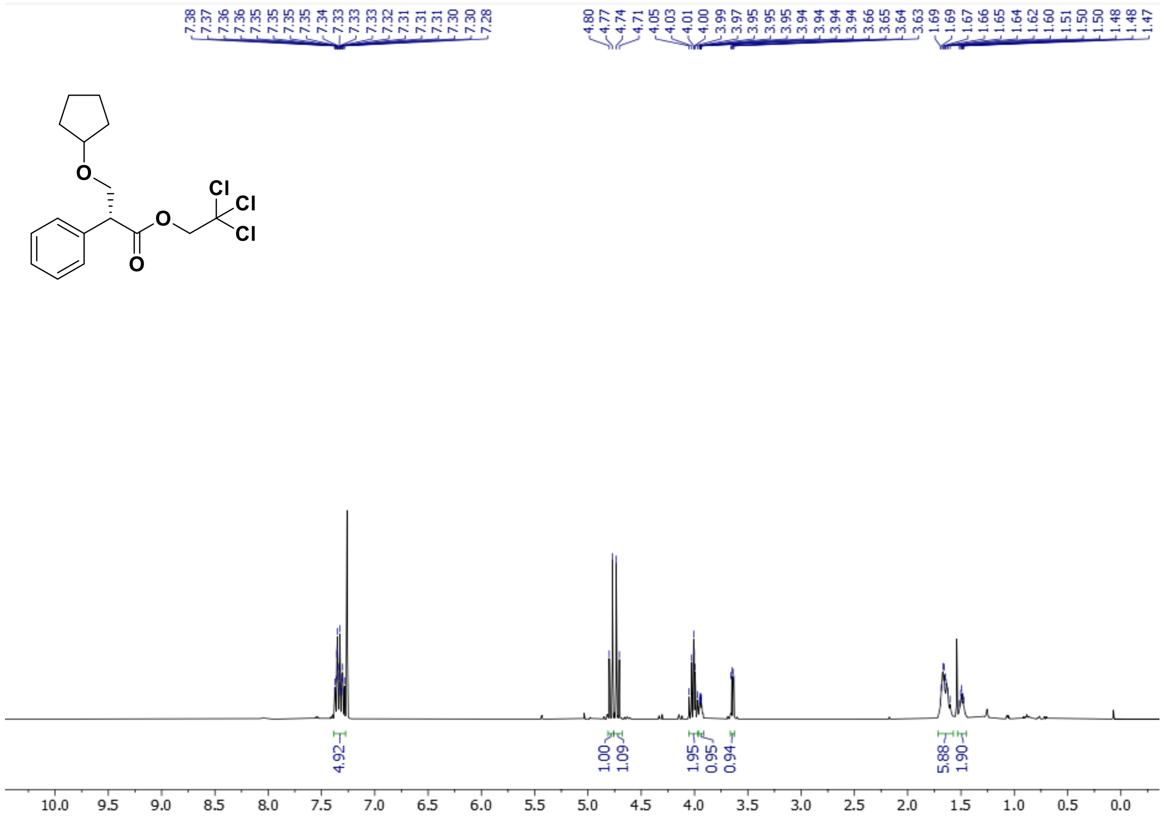

20a: ^{13}C NMR (101 MHz, $CDCl_3$)

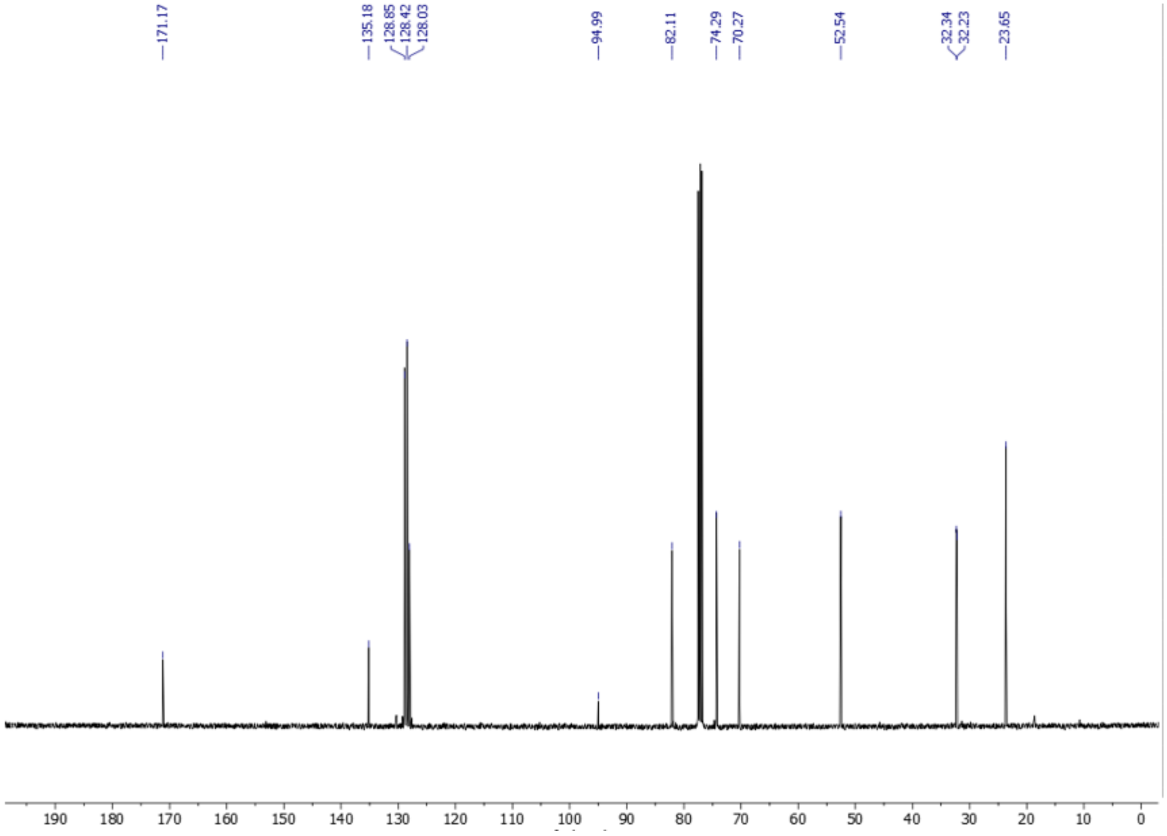

20b: ^{1}H NMR (400 MHz, $CDCl_3$)

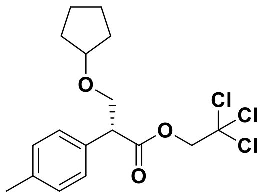

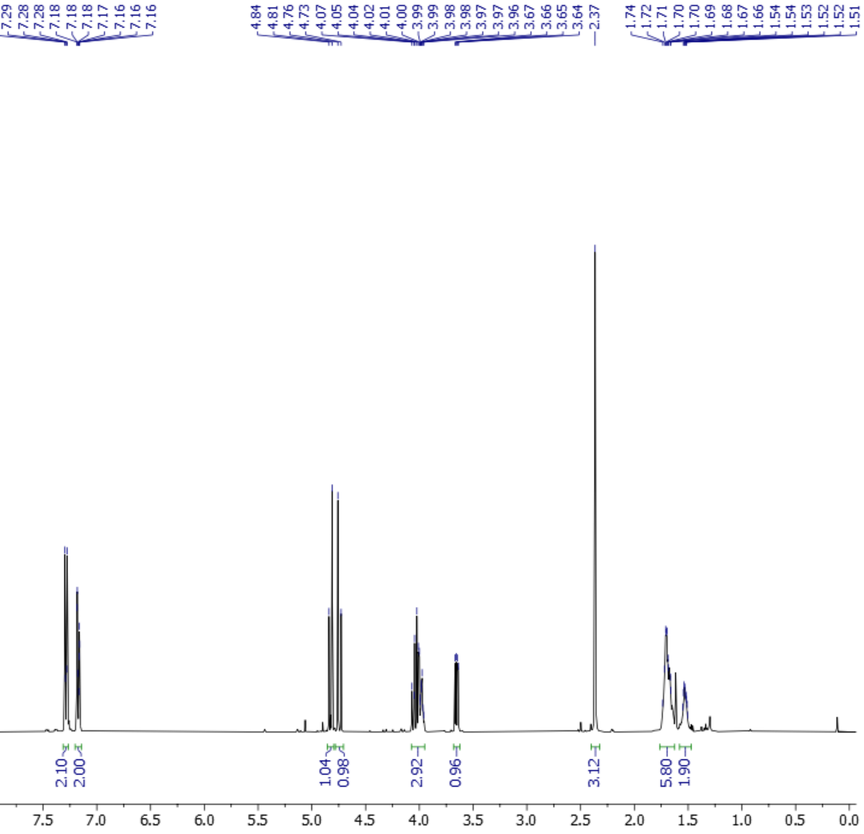

20b: ^{13}C NMR (101 MHz, $CDCl_3$)

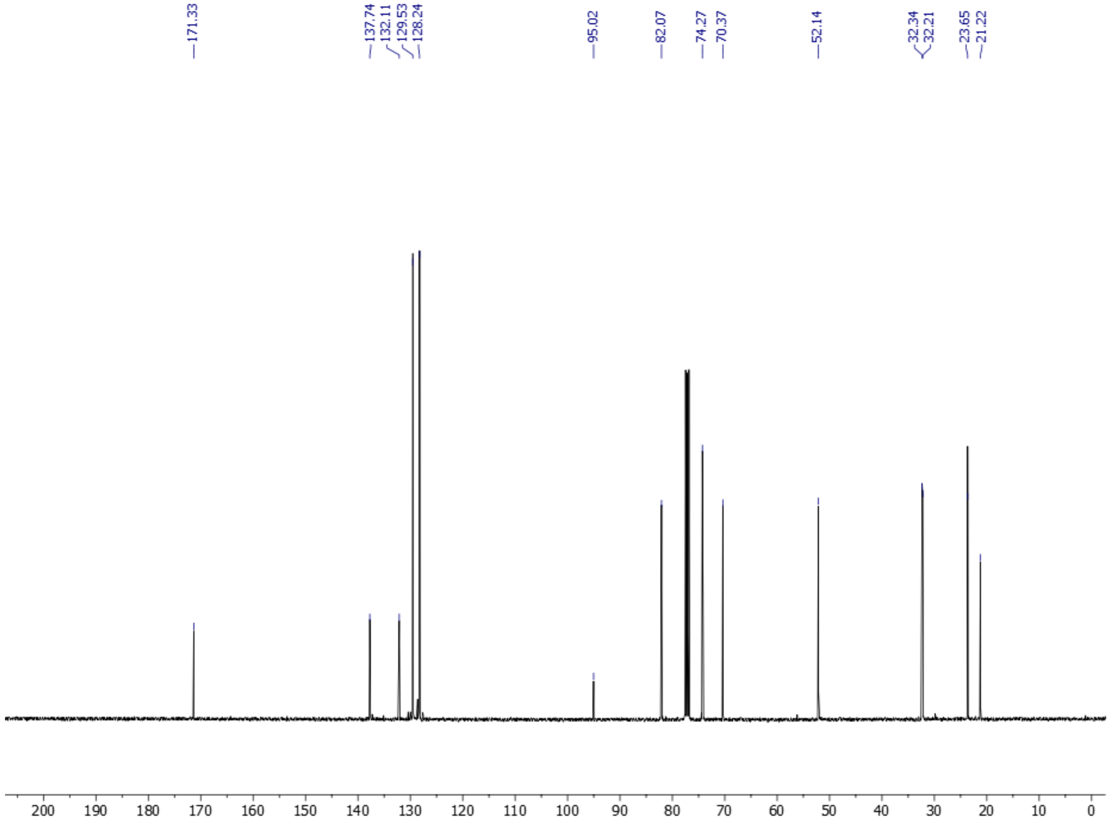

20c: ^{1}H NMR (400 MHz, $CDCl_3$)

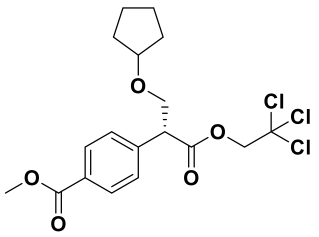

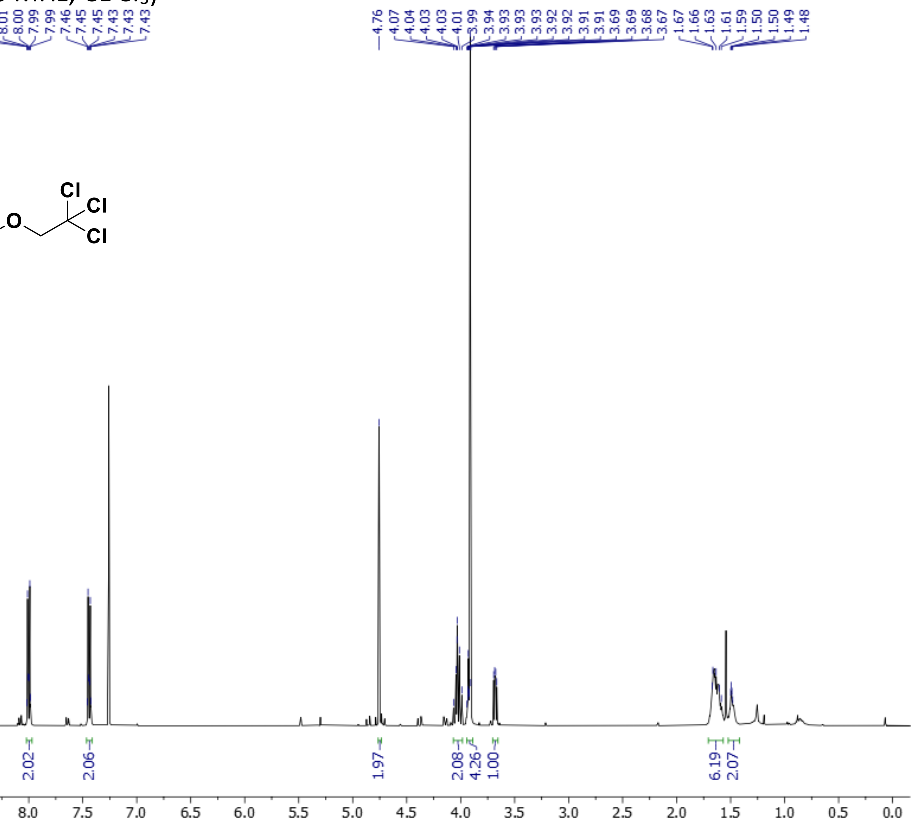

20c: ^{13}C NMR (101 MHz, $CDCl_3$)

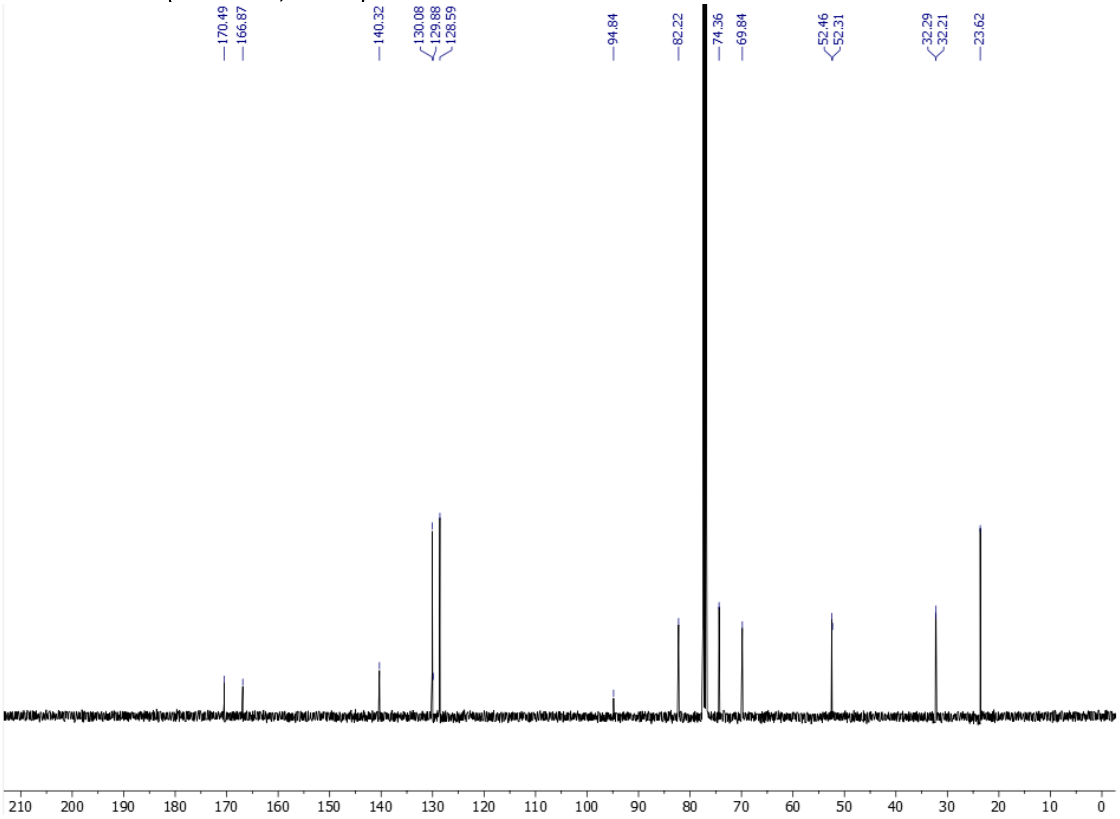

20d: ^{1}H NMR (400 MHz, $CDCl_3$)

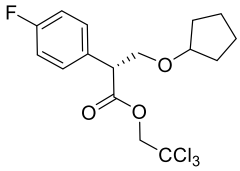

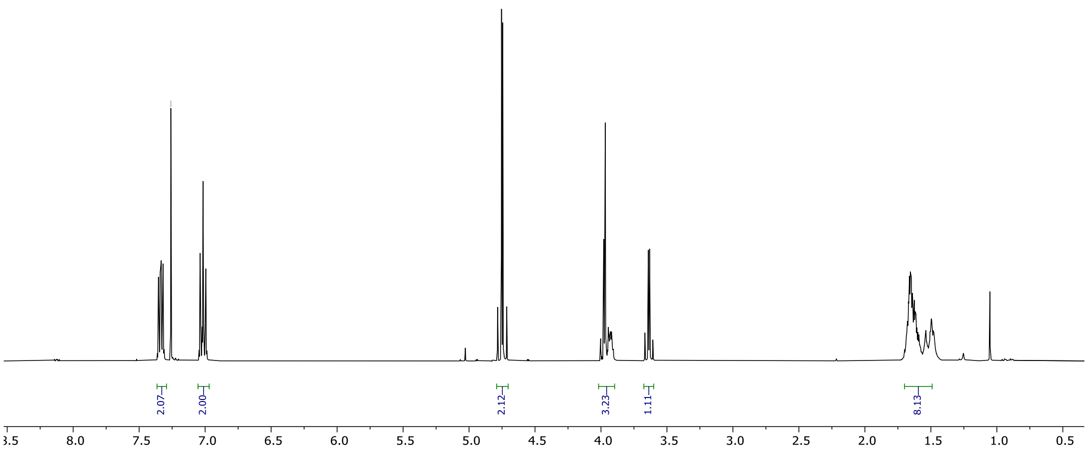

20d: ^{13}C NMR (101 MHz, $CDCl_3$)

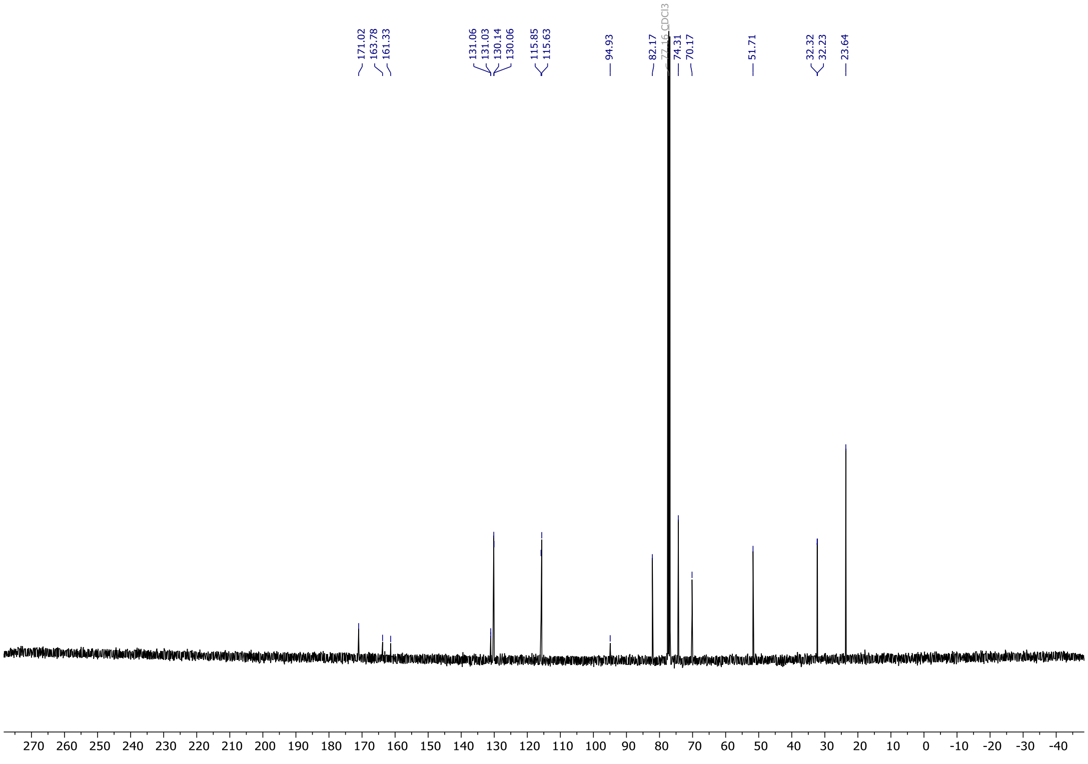

20d: ^{19}F NMR (282 MHz, $CDCl_3$)

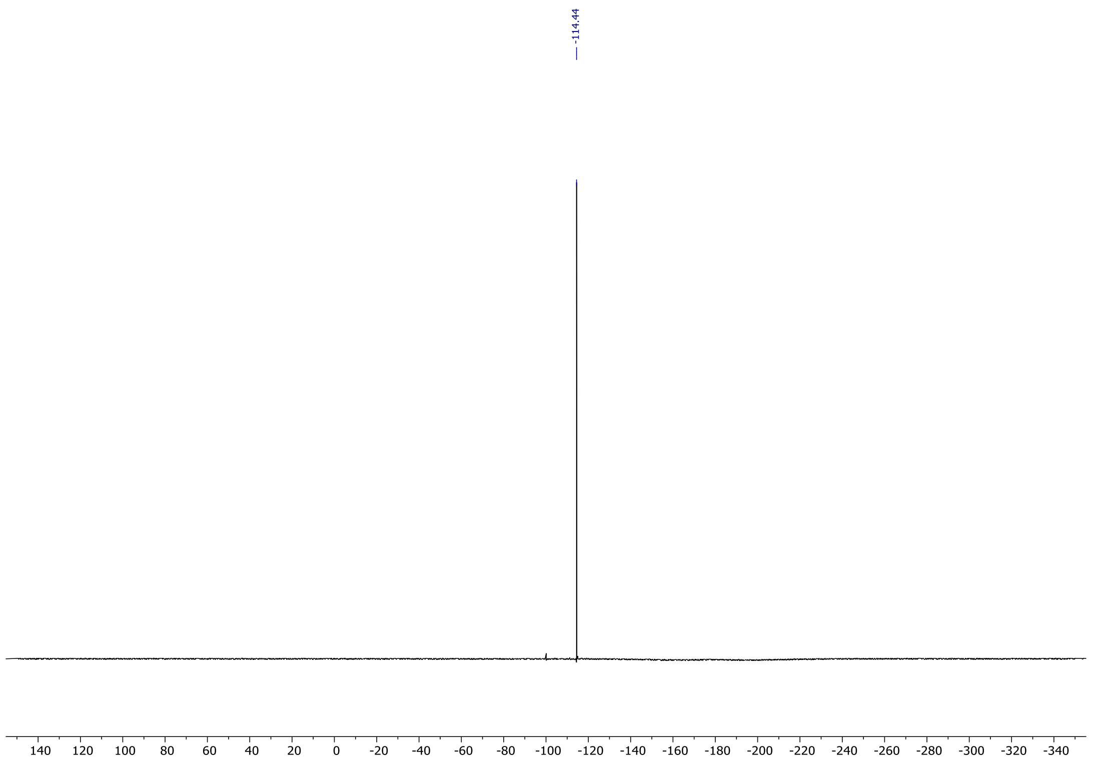

20e: ^{1}H NMR (400 MHz, $CDCl_3$)

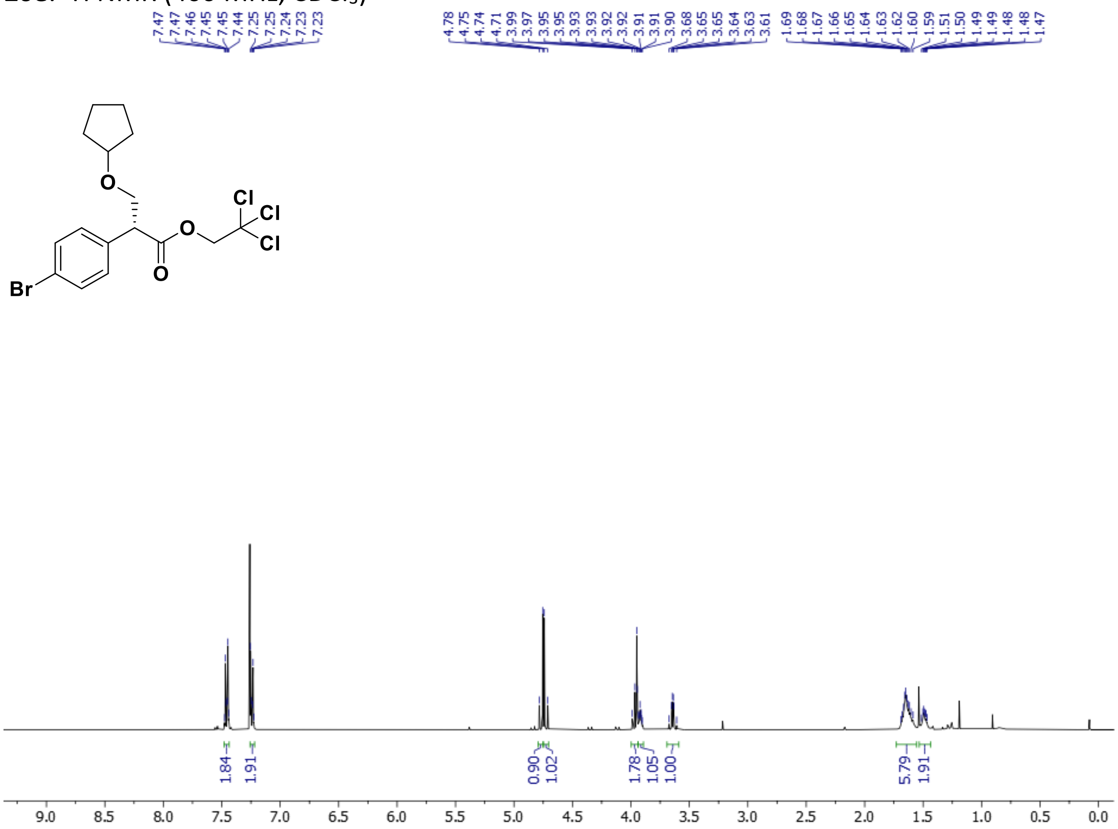

20e: ^{13}C NMR (101 MHz, $CDCl_3$)

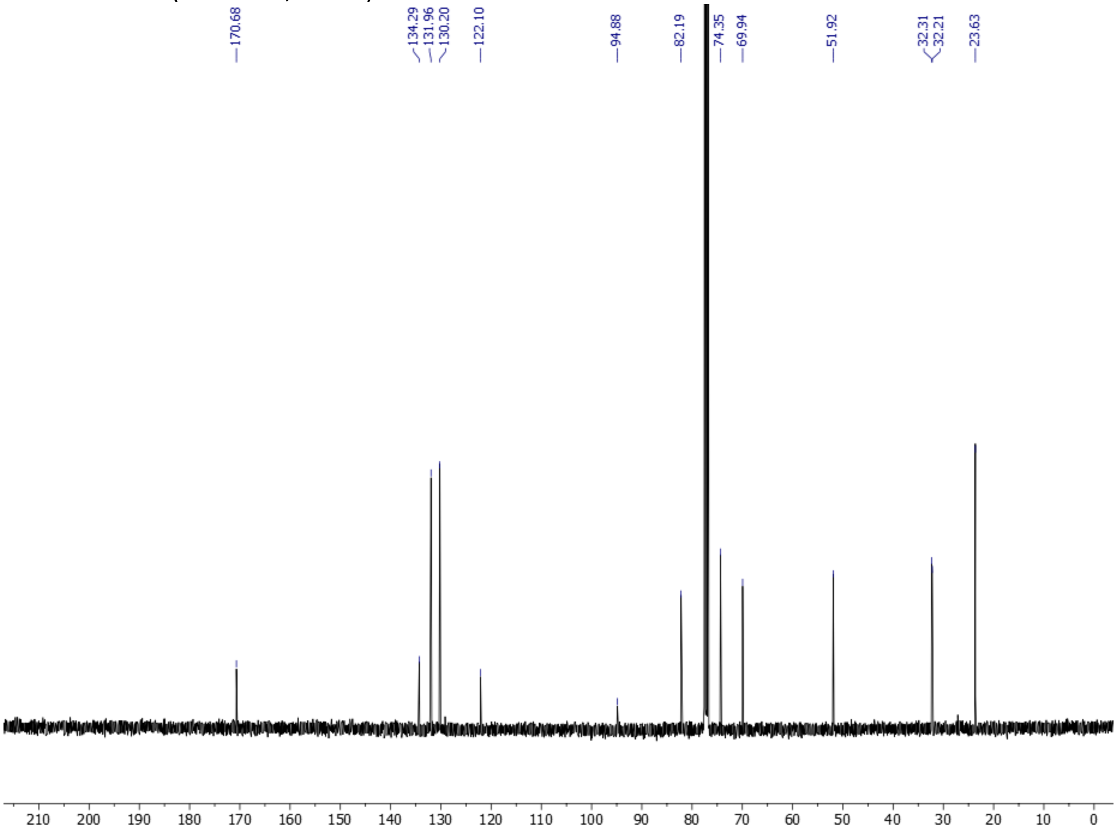

20f: ^{1}H NMR (400 MHz, $CDCl_3$)

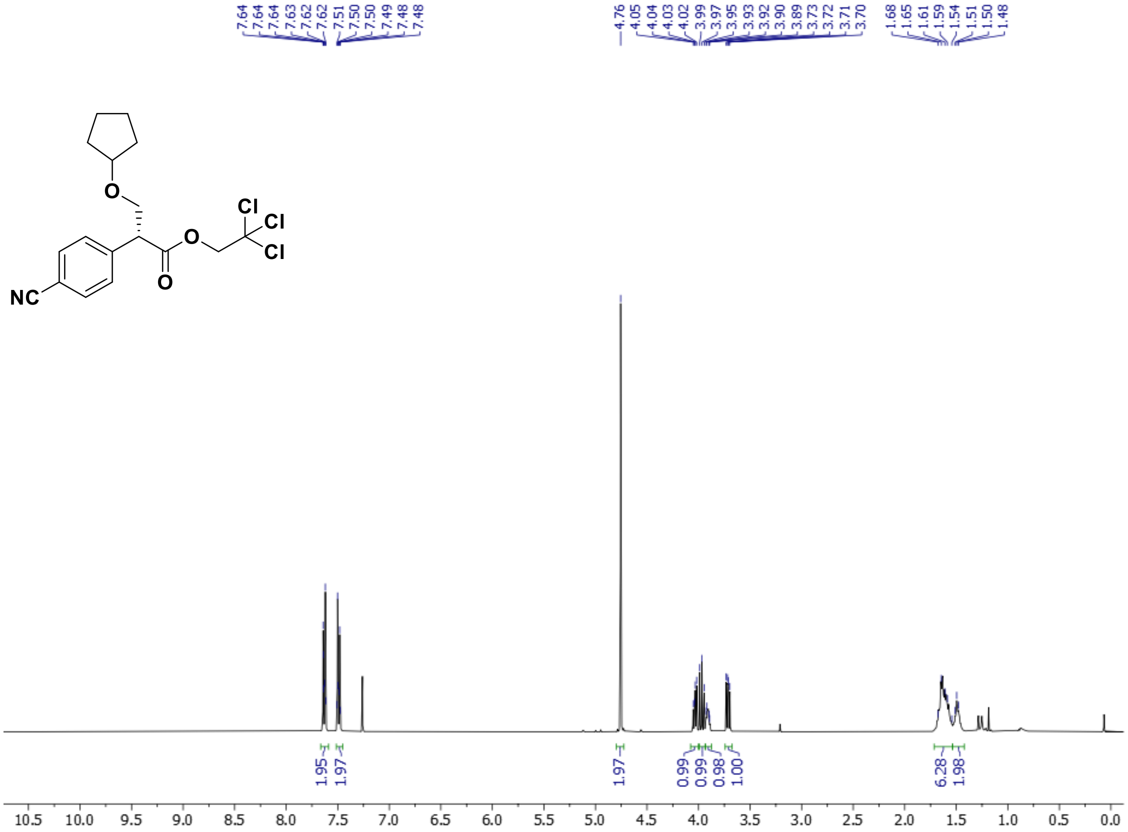

20f: ^{13}C NMR (101 MHz, $CDCl_3$)

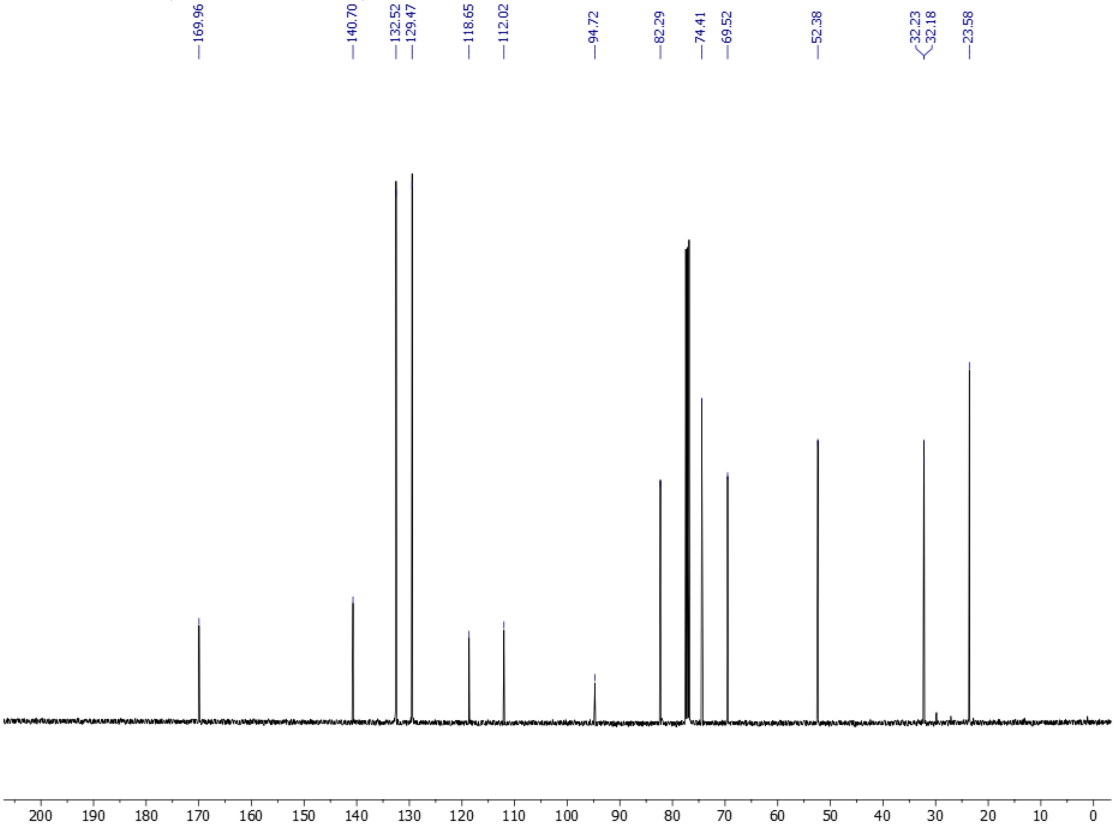

20g: ^{1}H NMR (400 MHz, $CDCl_3$)

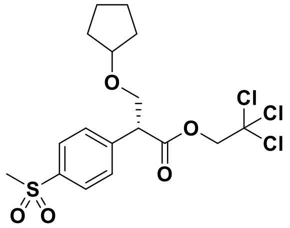

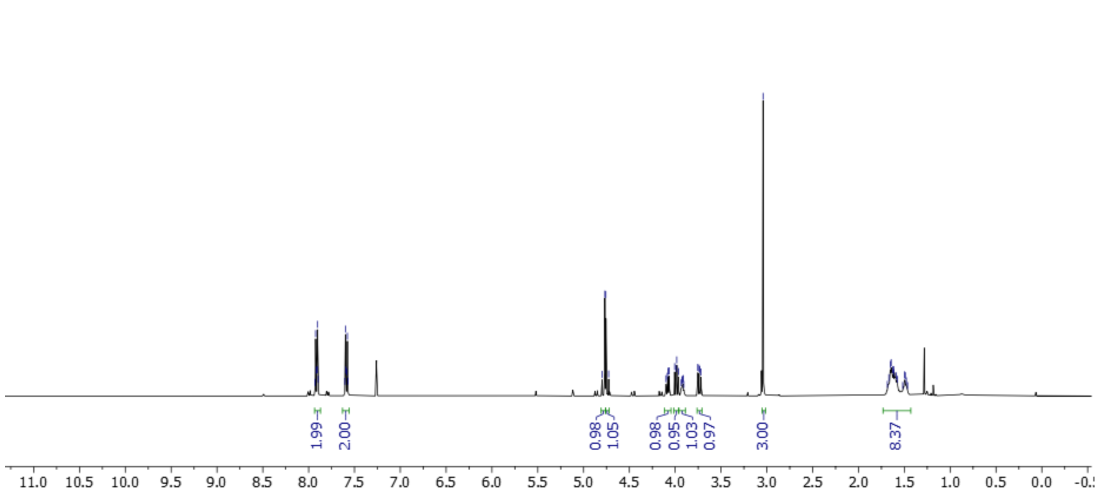

20g: ^{13}C NMR (101 MHz, $CDCl_3$)

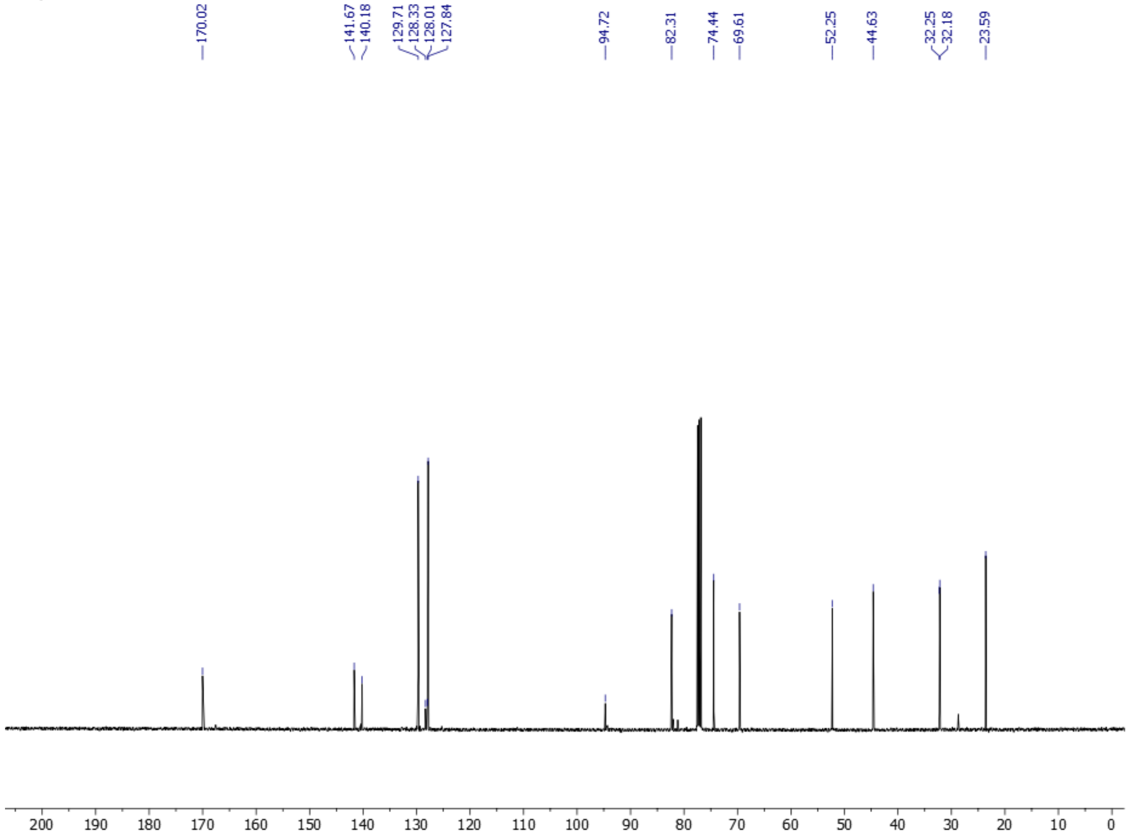

20h: ^{1}H NMR (400 MHz, $CDCl_3$)

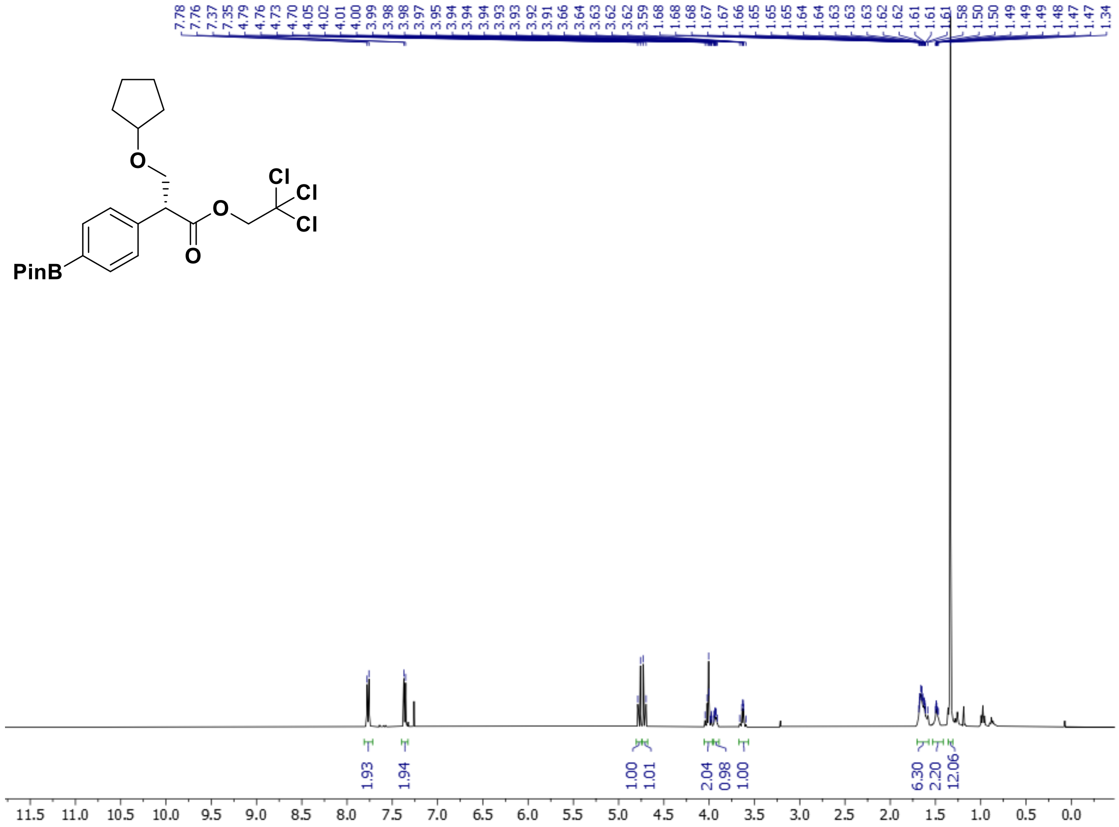

20h: ^{13}C NMR (101 MHz, $CDCl_3$)

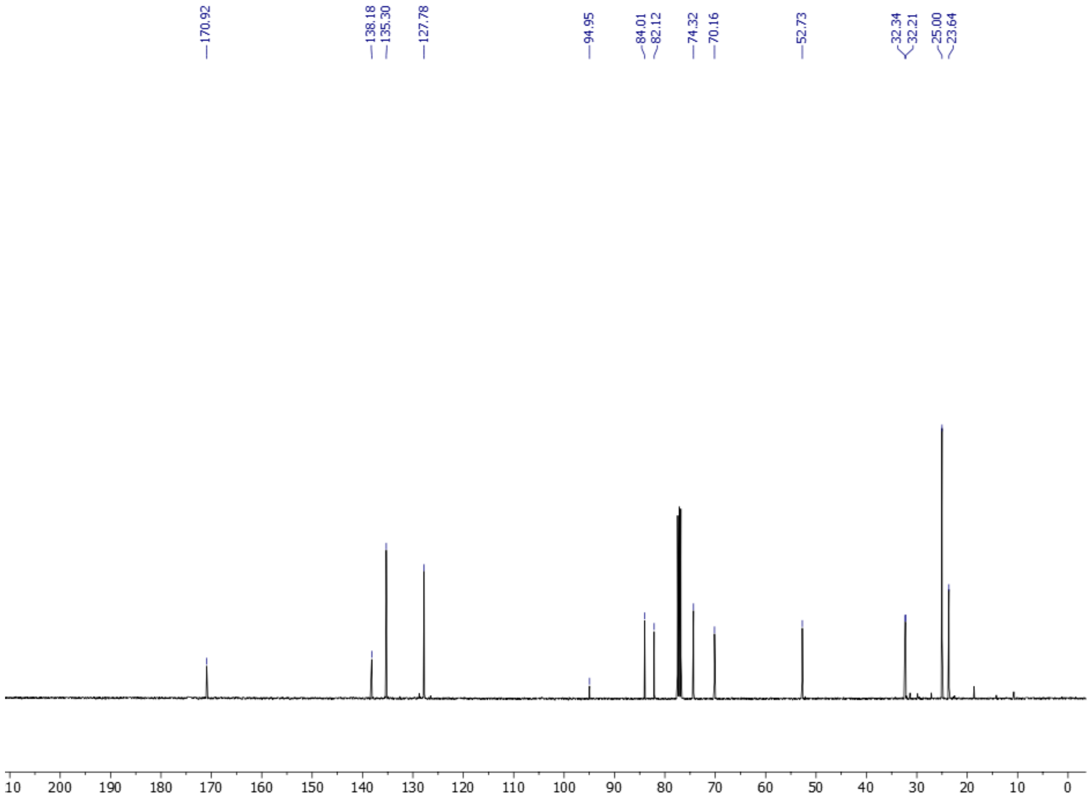

21a: ^{1}H NMR (400 MHz, $CDCl_3$)

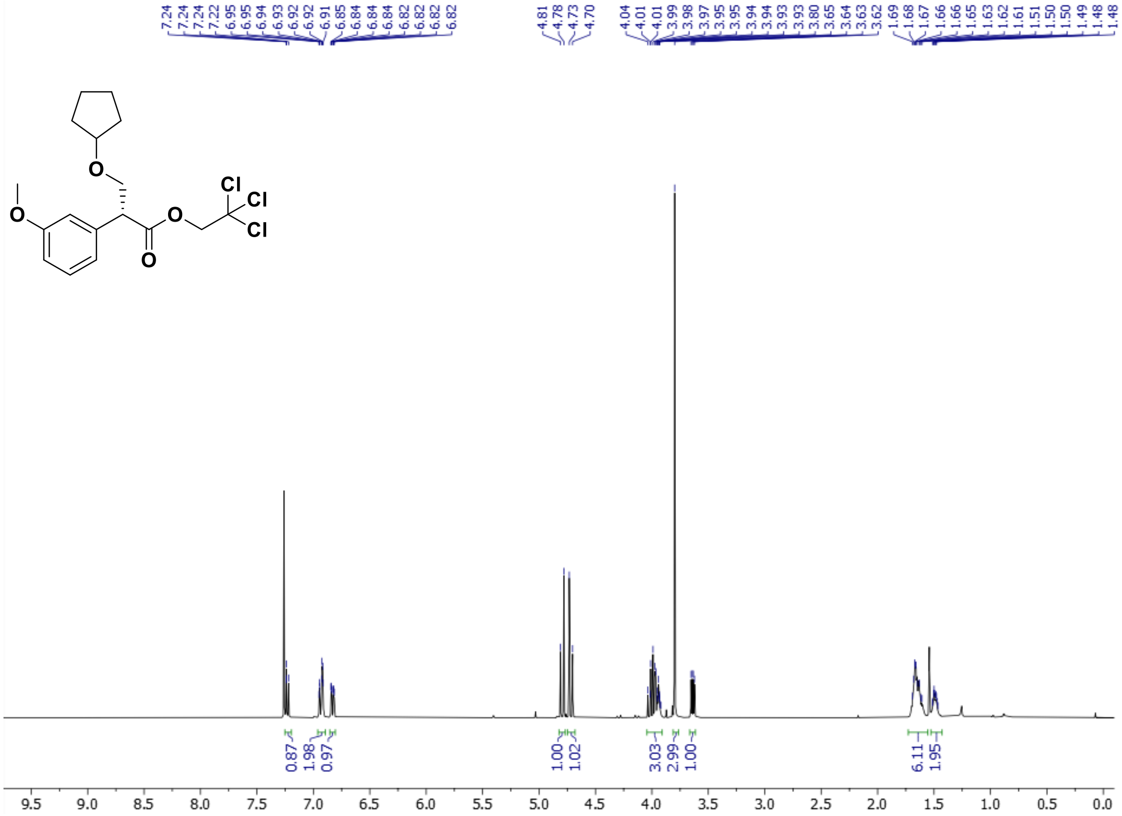

21a: ^{13}C NMR (101 MHz, $CDCl_3$)

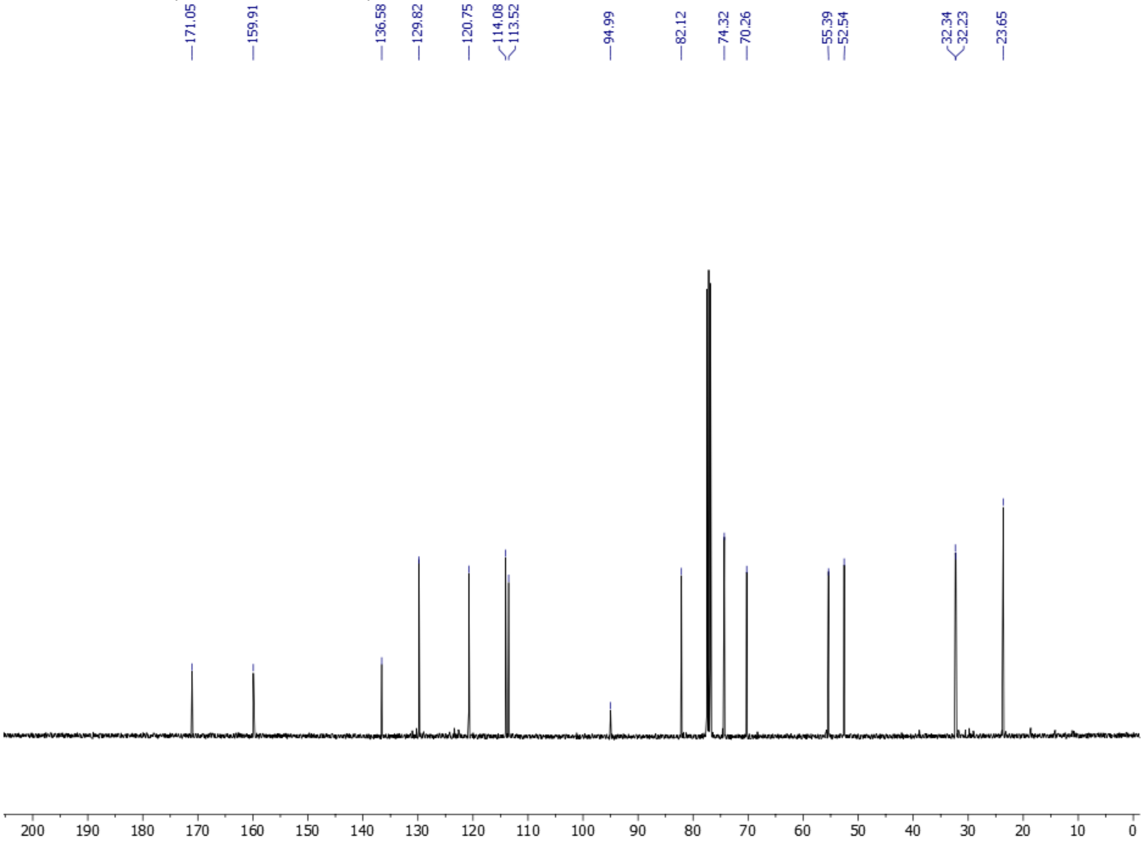

21b: ^{1}H NMR (400 MHz, $CDCl_3$)

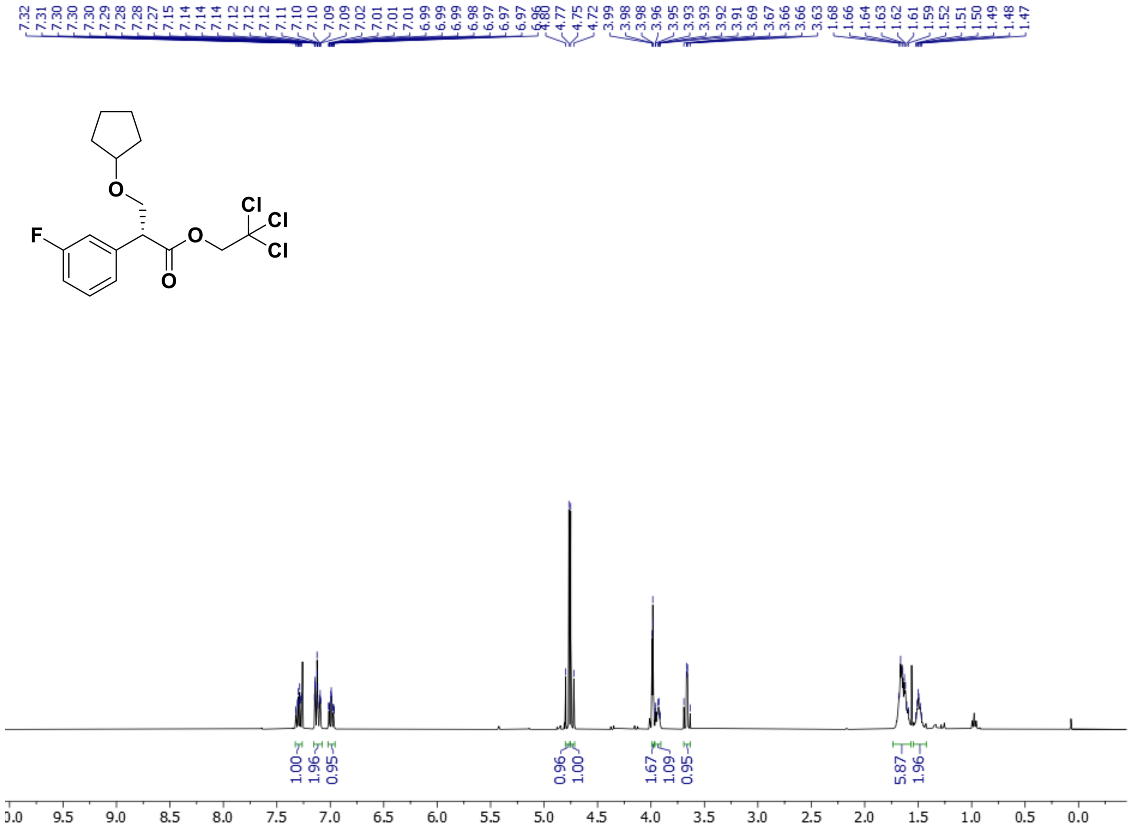

21b: ^{13}C NMR (101 MHz, $CDCl_3$)

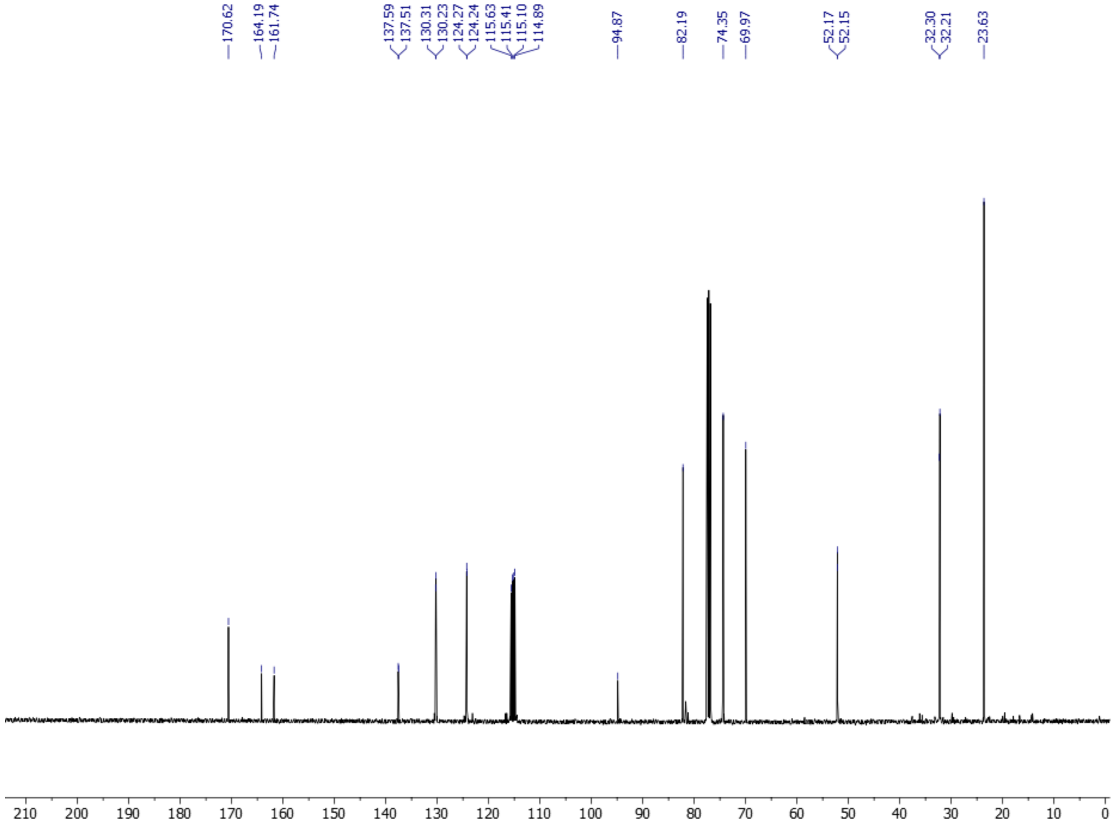

21b: ^{19}F NMR (282 MHz, $CDCl_3$)

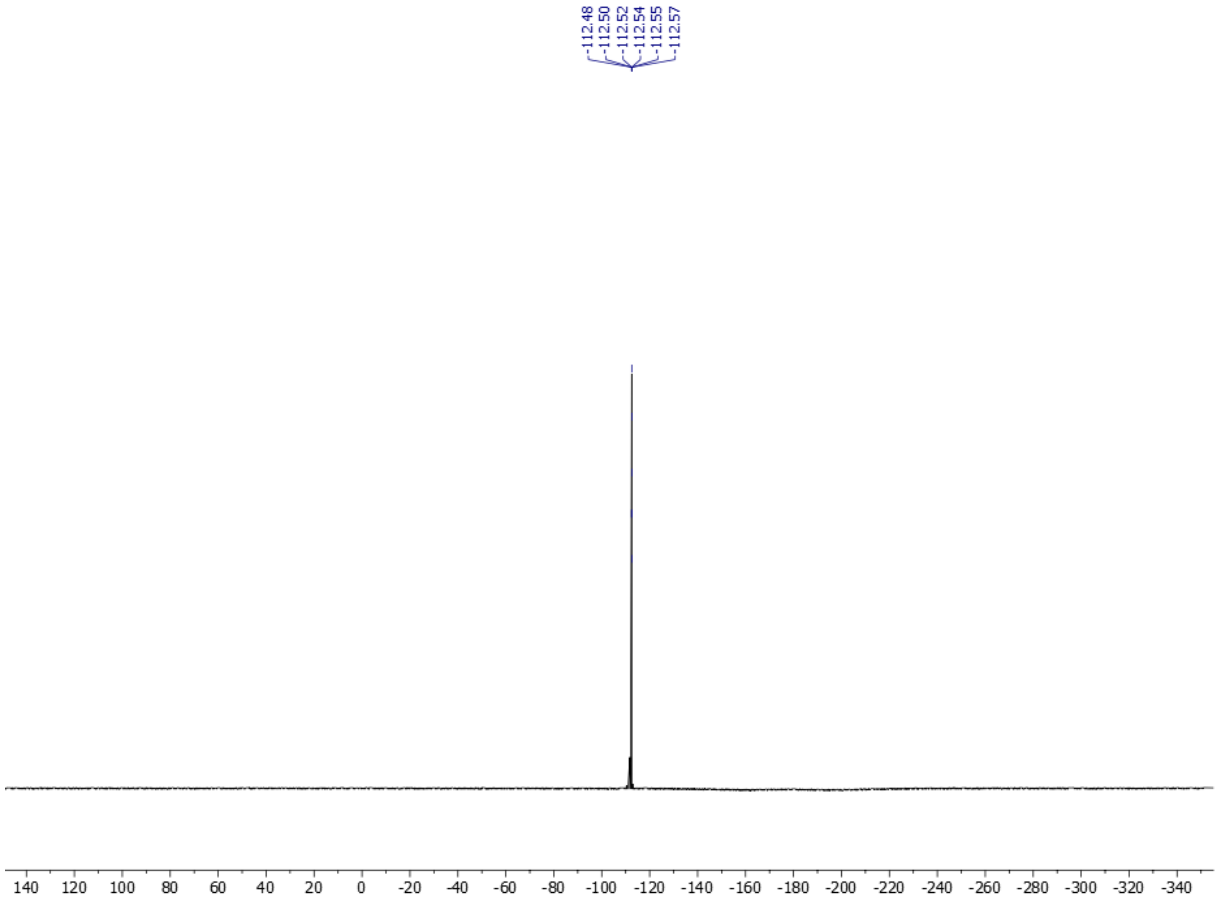

22: ^{1}H NMR (400 MHz, $CDCl_3$)

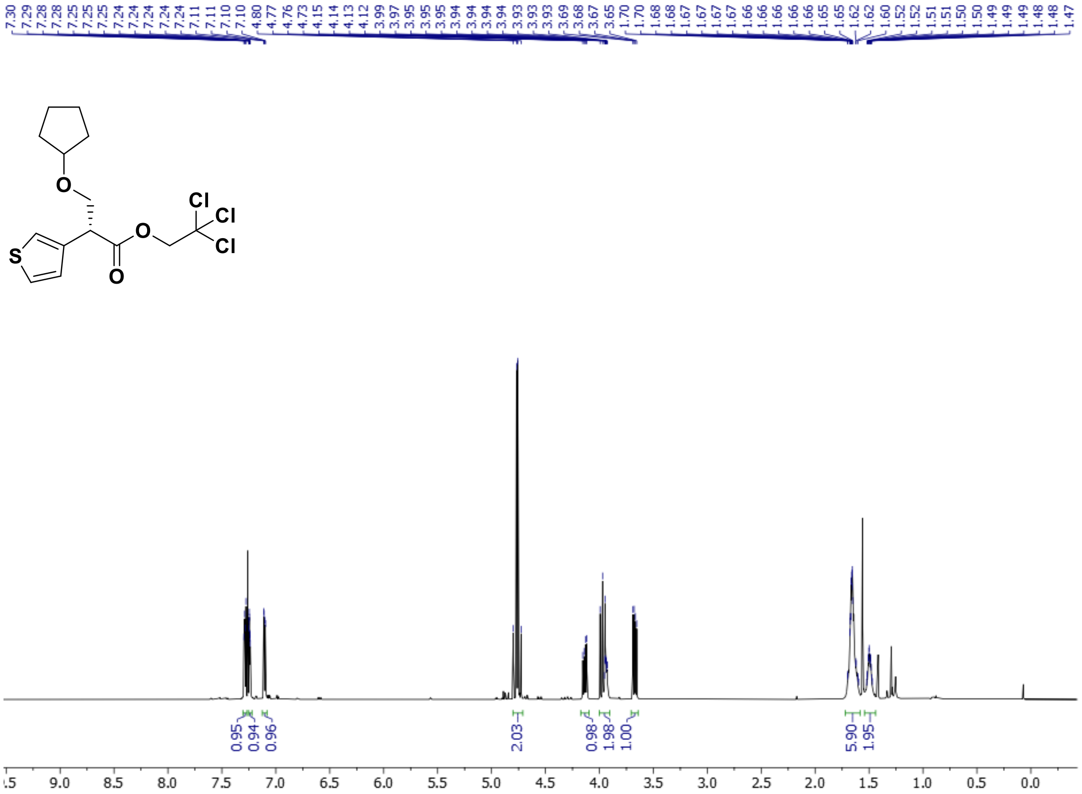

22: ^{13}C NMR (101 MHz, $CDCl_3$)

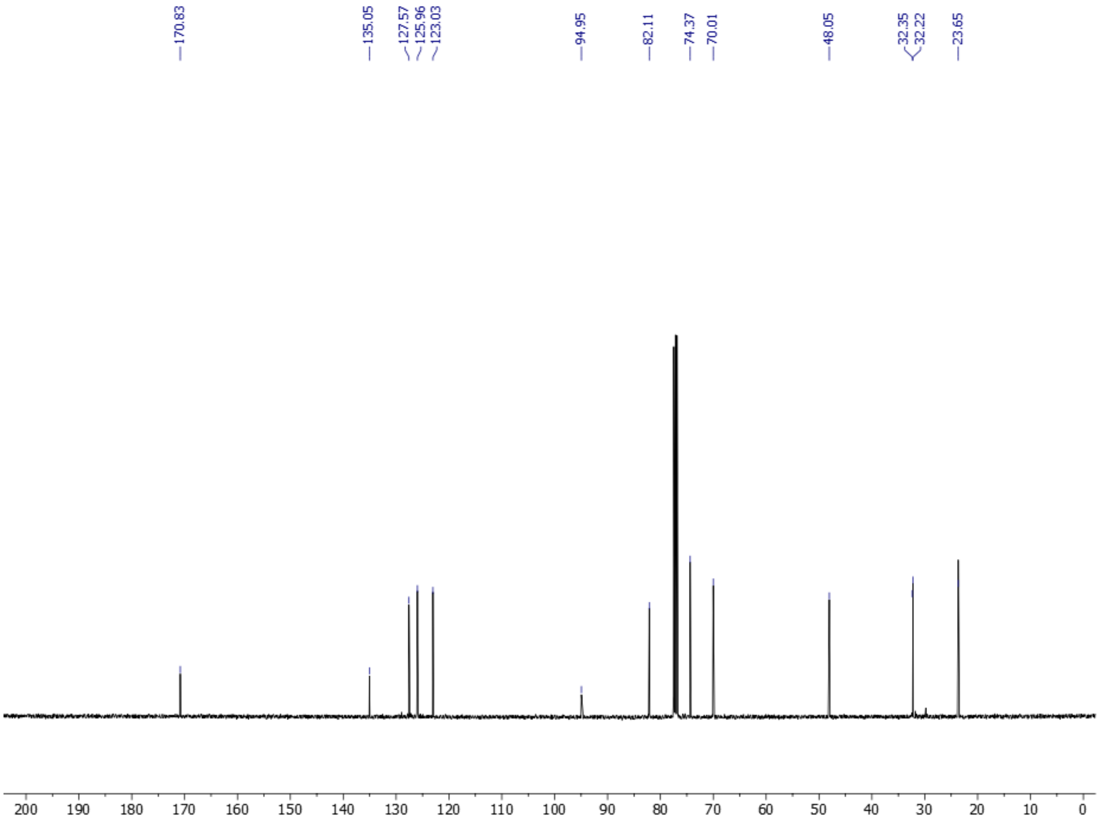

23a: ^{1}H NMR (400 MHz, $CDCl_3$):

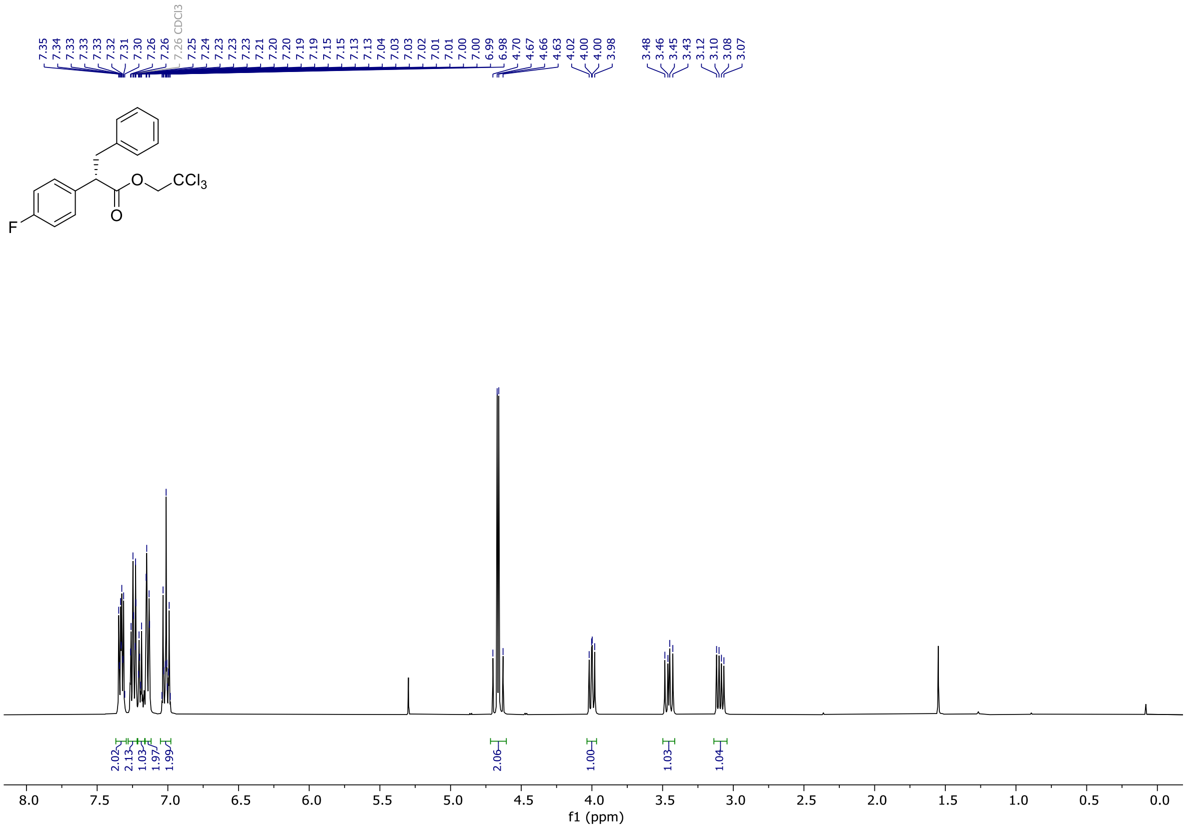

23a: ^{13}C NMR (101 MHz, $CDCl_3$):

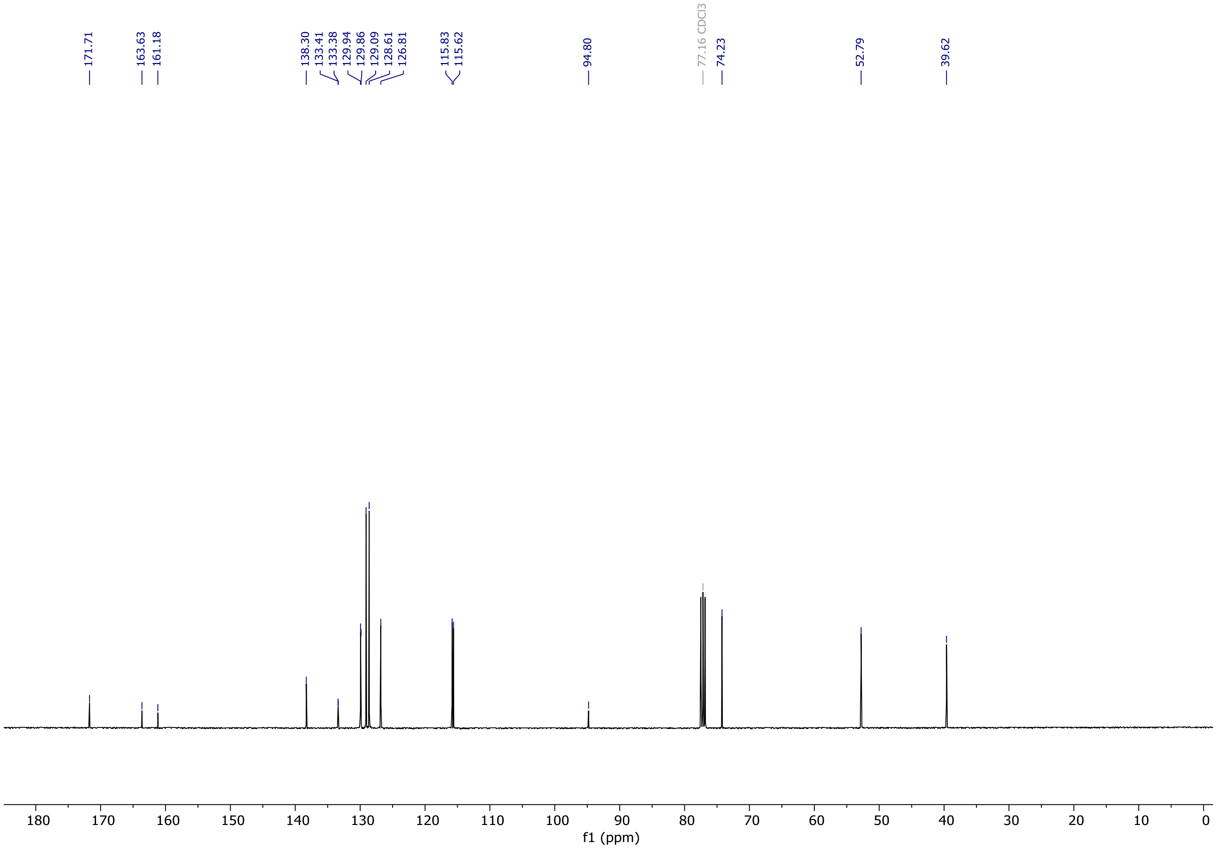

23a: ^{19}F NMR (282 MHz, $CDCl_3$):

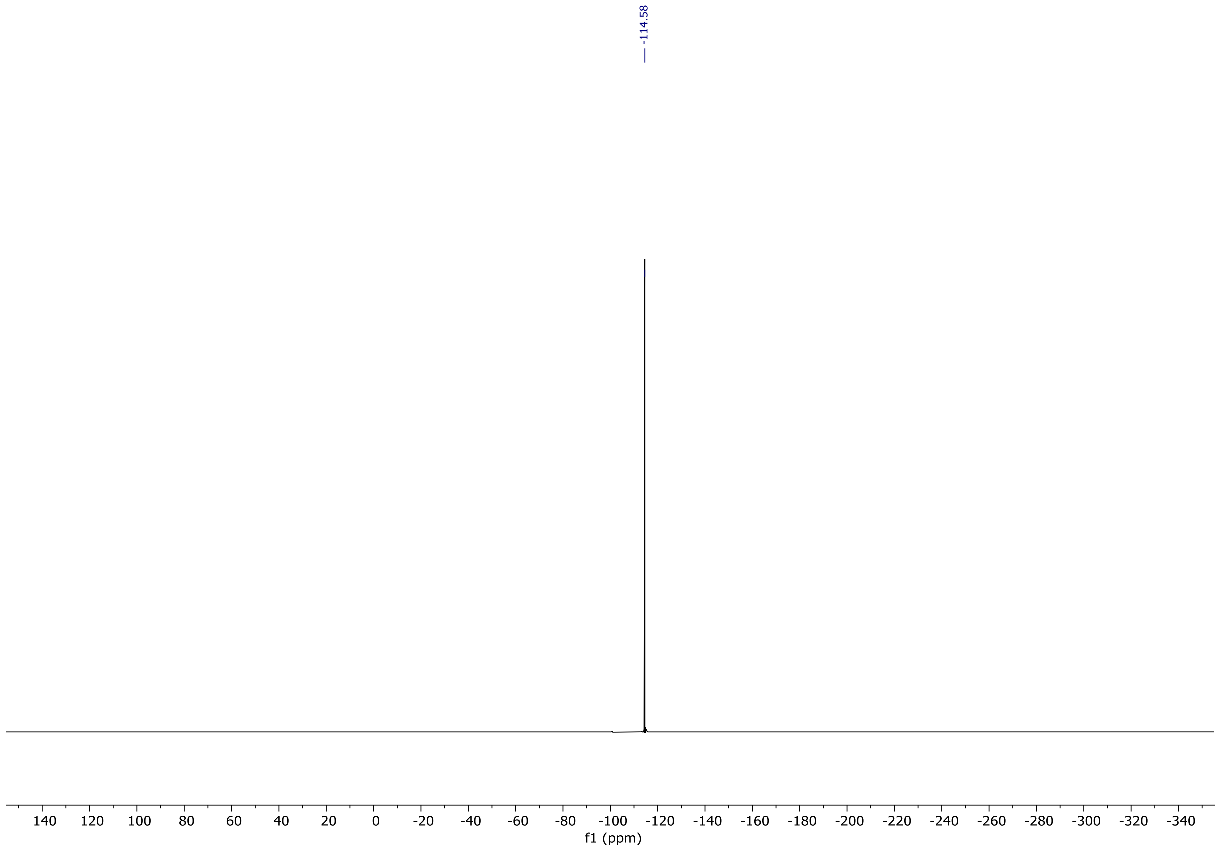

23b: ^{1}H NMR (400 MHz, CDCl$_3$):

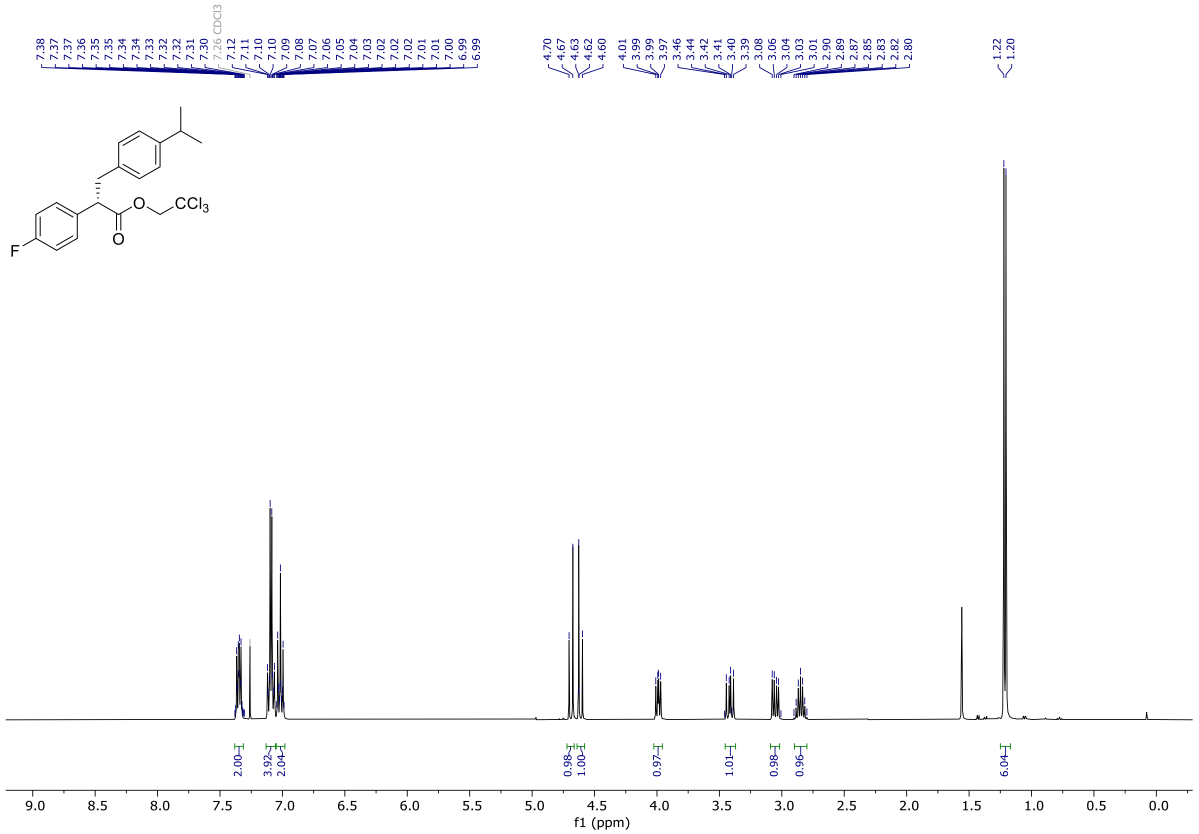

23b: ^{13}C NMR (101 MHz, CDCl$_3$):

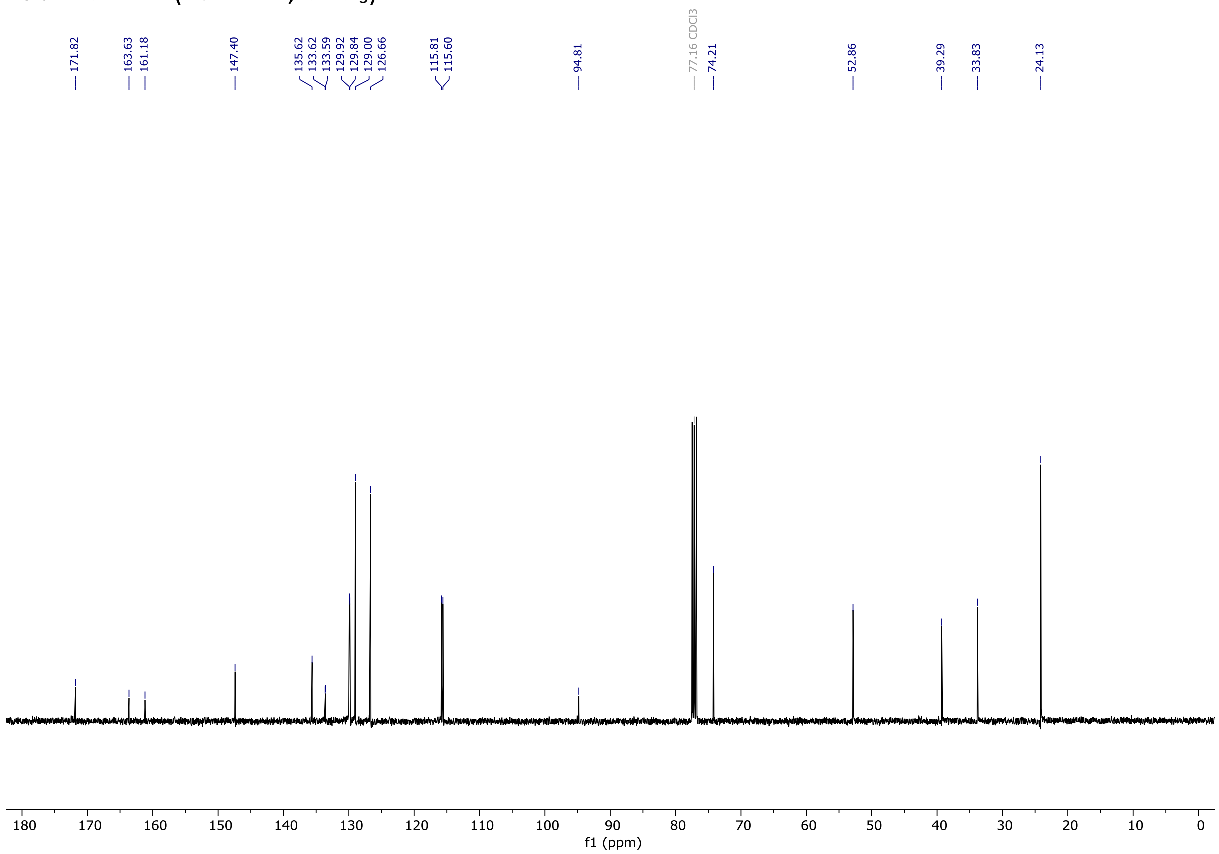

23b: ^{19}F NMR (282 MHz, $CDCl_3$):

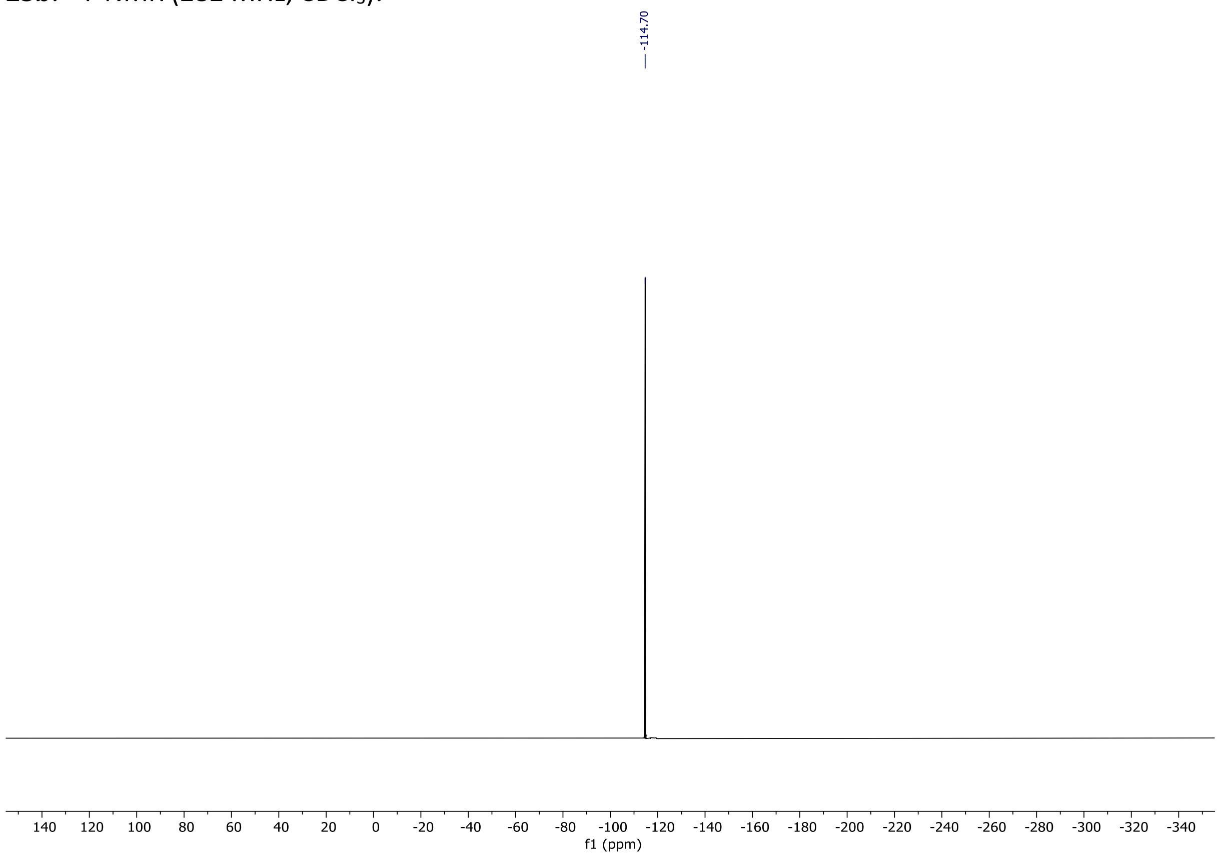

24: ^{1}H NMR (400 MHz, $CDCl_3$):

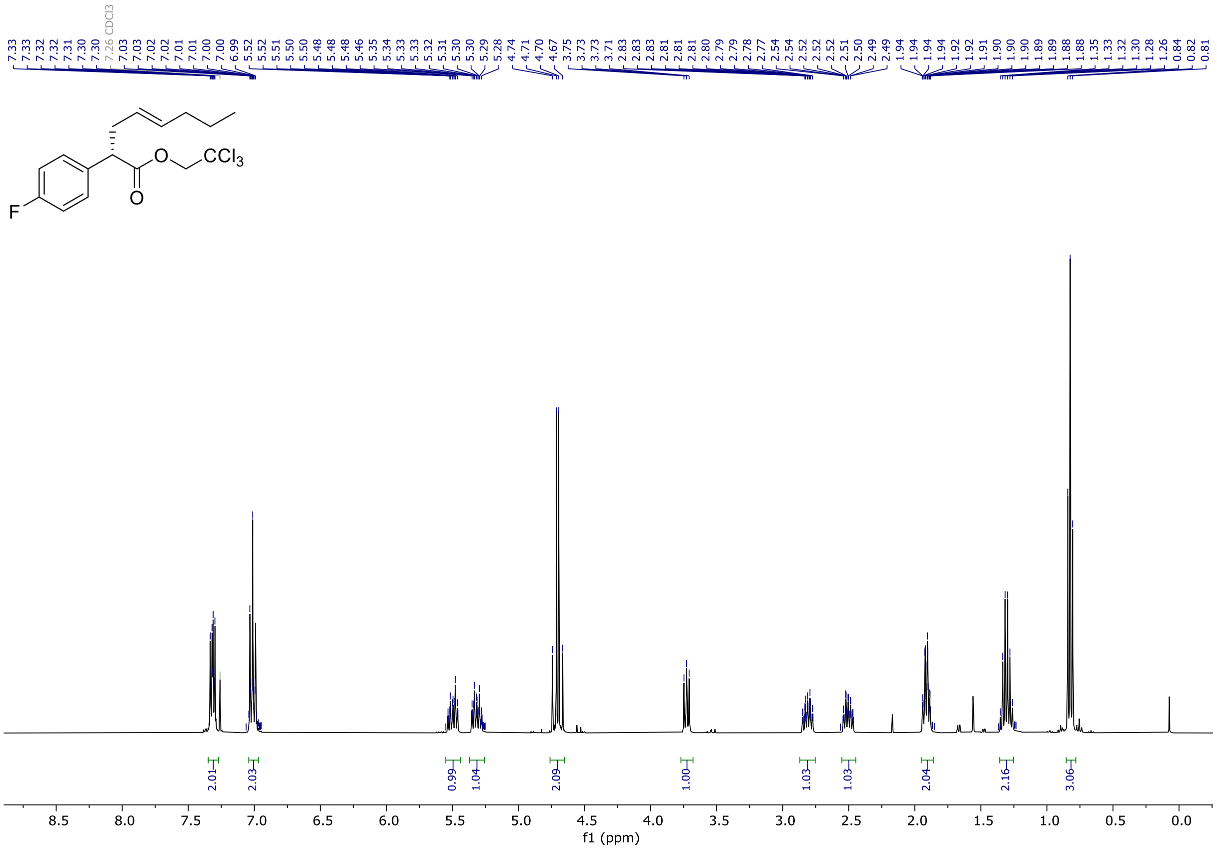

24: ^{13}C NMR (101 MHz, $CDCl_3$):

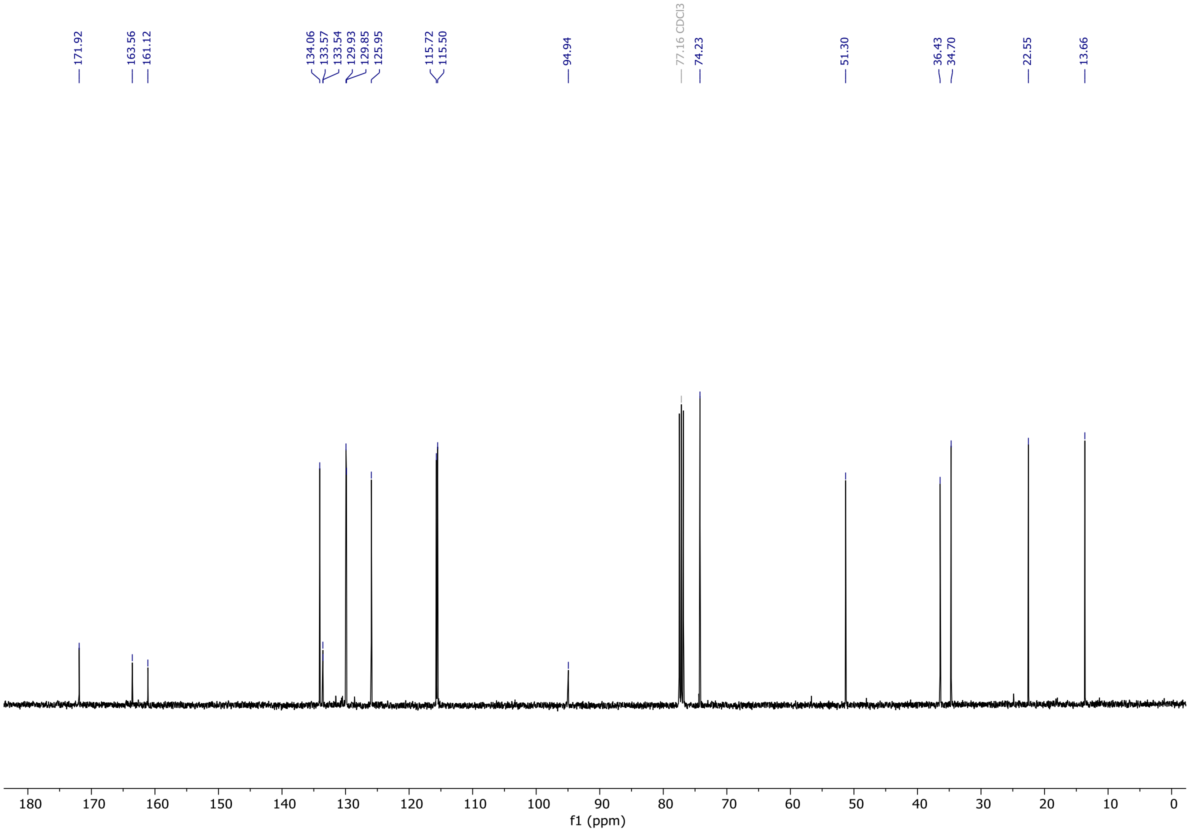

24: ^{19}F NMR (282 MHz, $CDCl_3$):

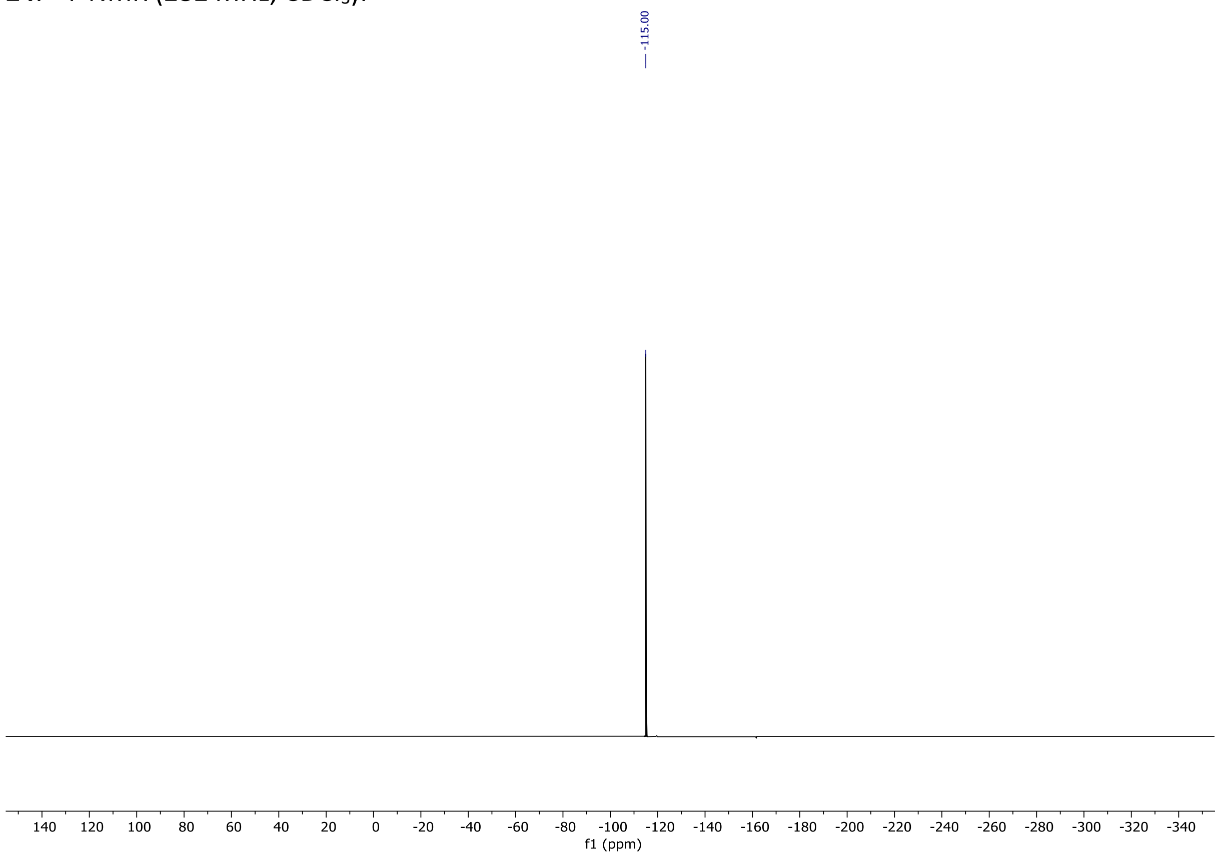

25: ^{1}H NMR (400 MHz, $CDCl_3$)

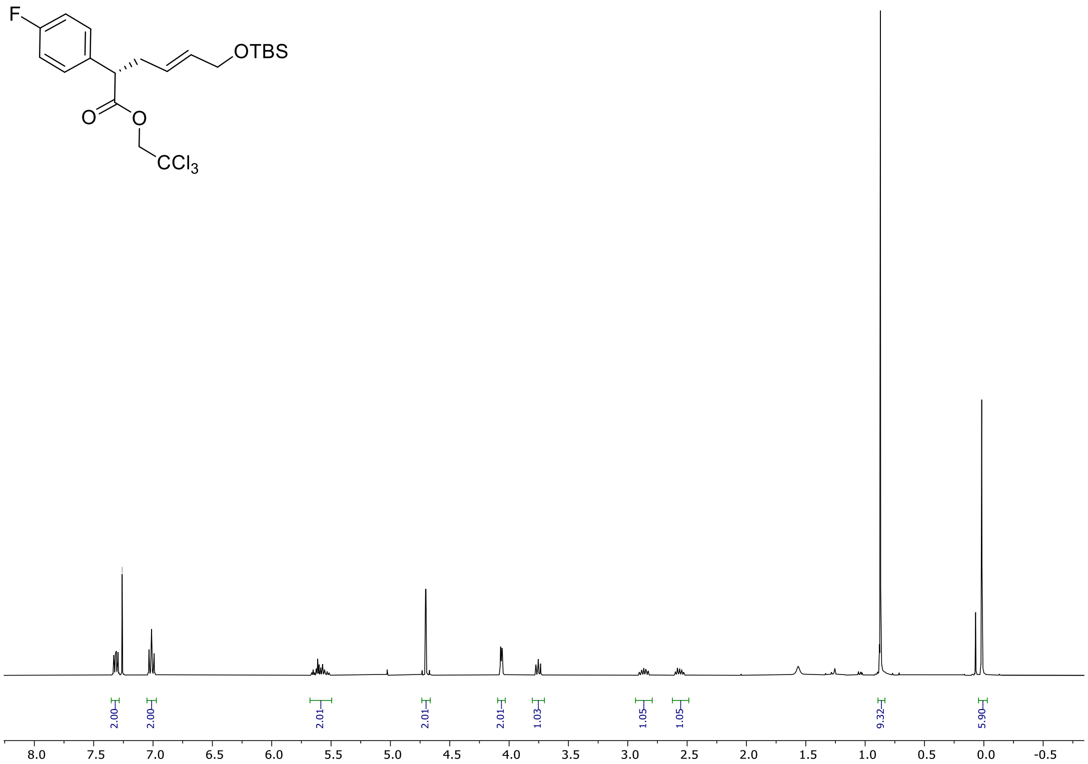

25: ^{13}C NMR (101 MHz, $CDCl_3$)

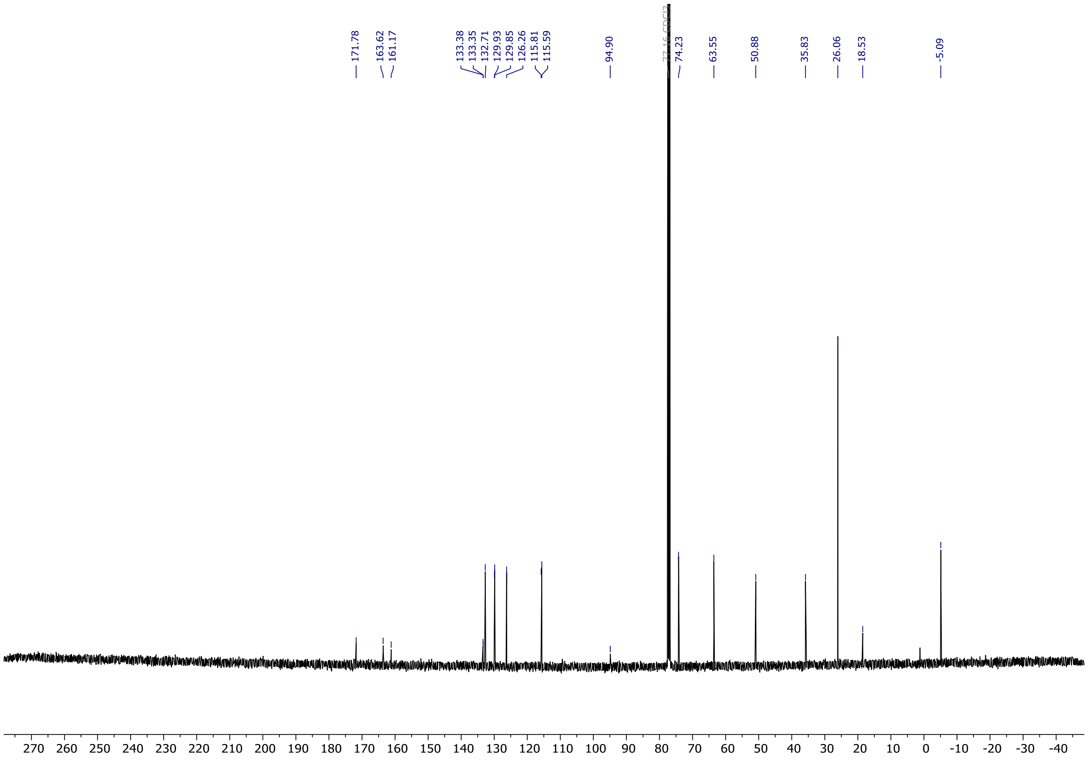

25: ^{19}F NMR (282 MHz, $CDCl_3$)

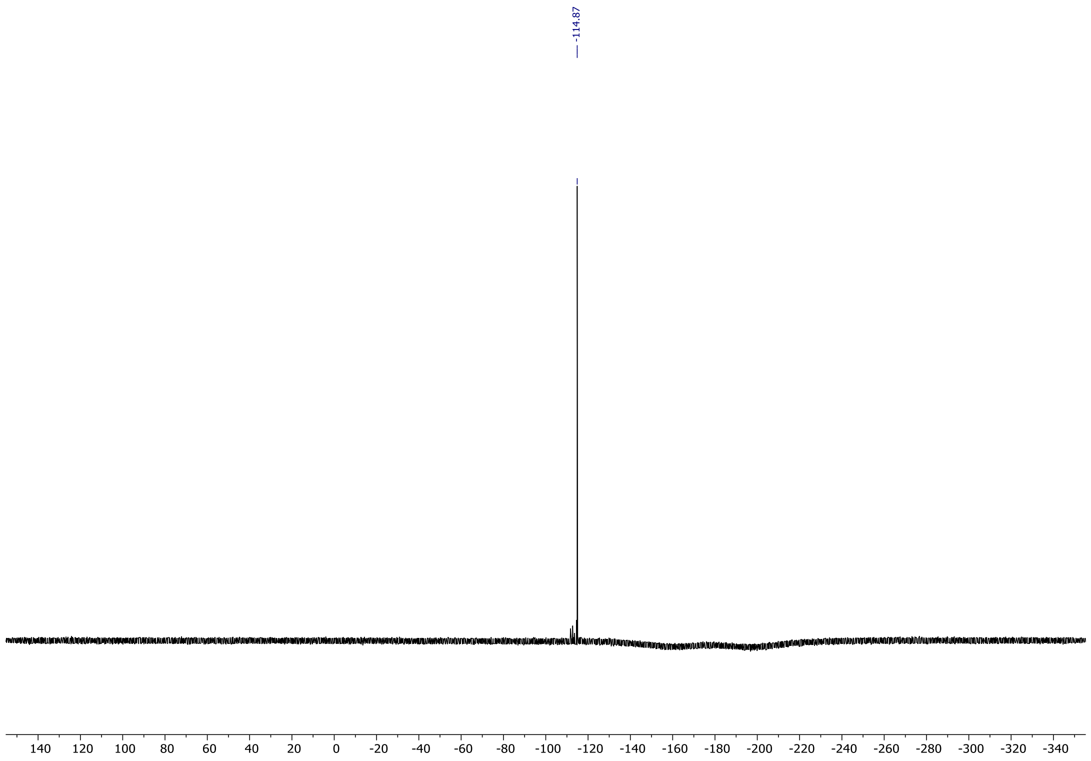

26: ^{1}H NMR (400 MHz, $CDCl_3$)

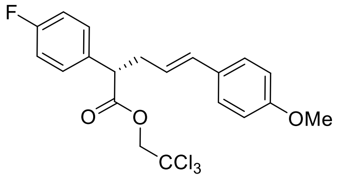

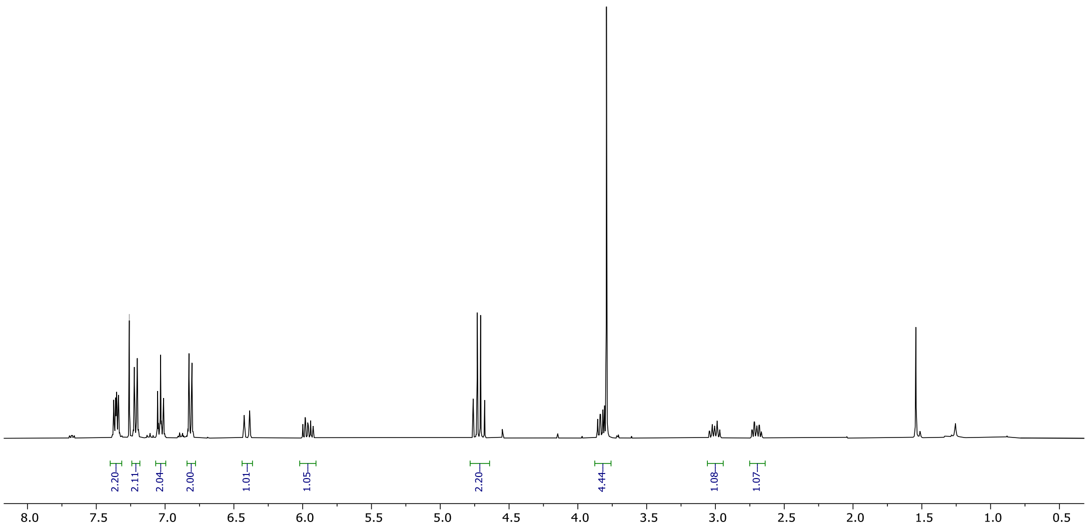

26: ^{13}C NMR (101 MHz, $CDCl_3$)

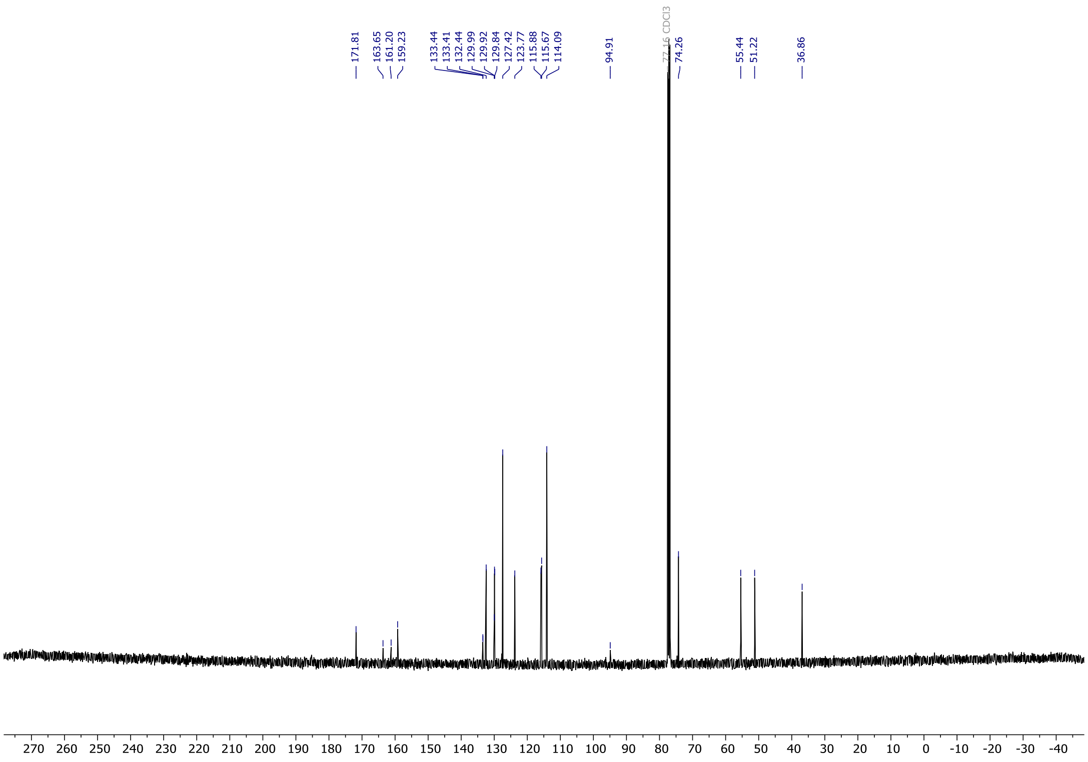

26: ^{19}F NMR (282 MHz, $CDCl_3$)

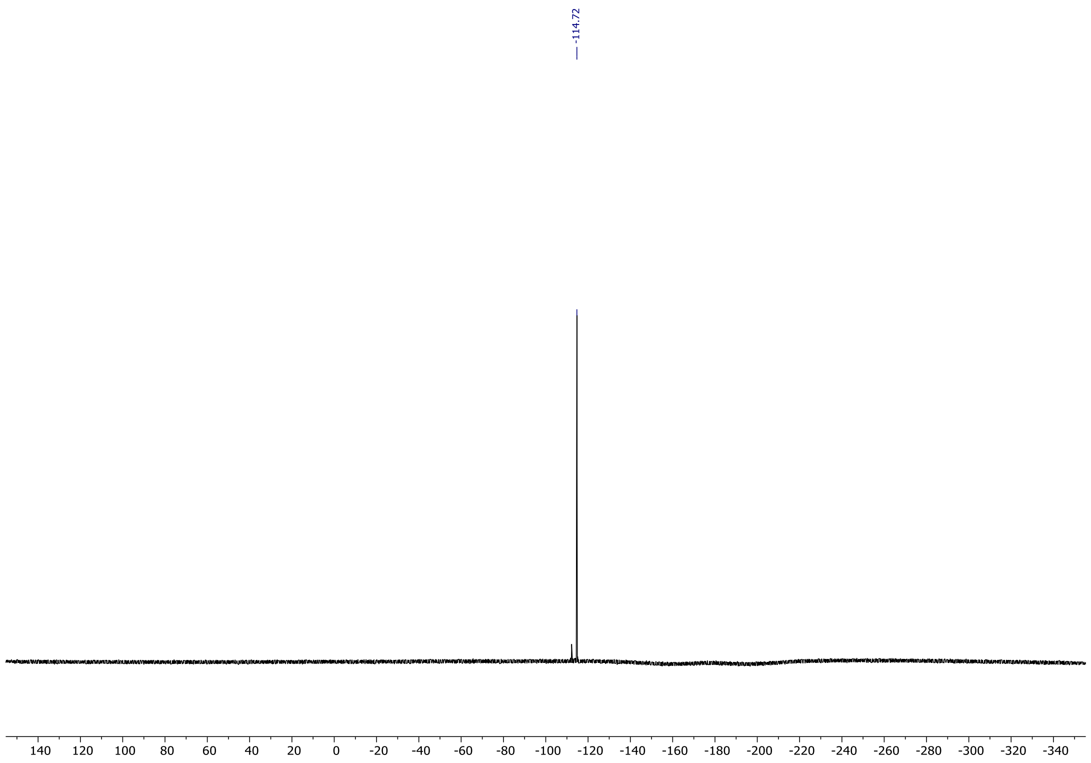

27: ^{1}H NMR (400 MHz, $CDCl_3$)

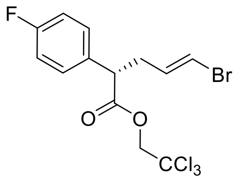

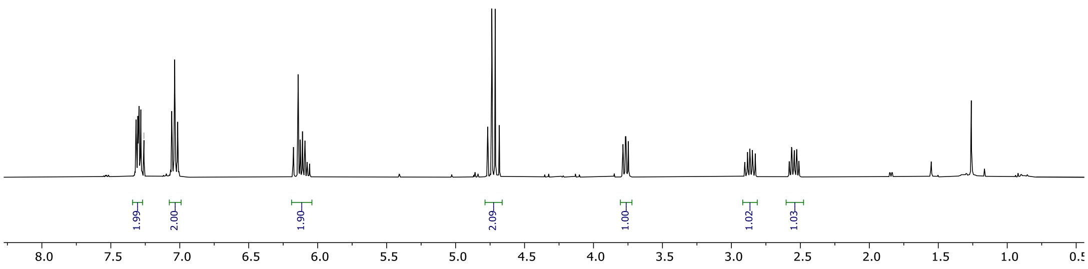

27: ^{13}C NMR (101 MHz, $CDCl_3$)

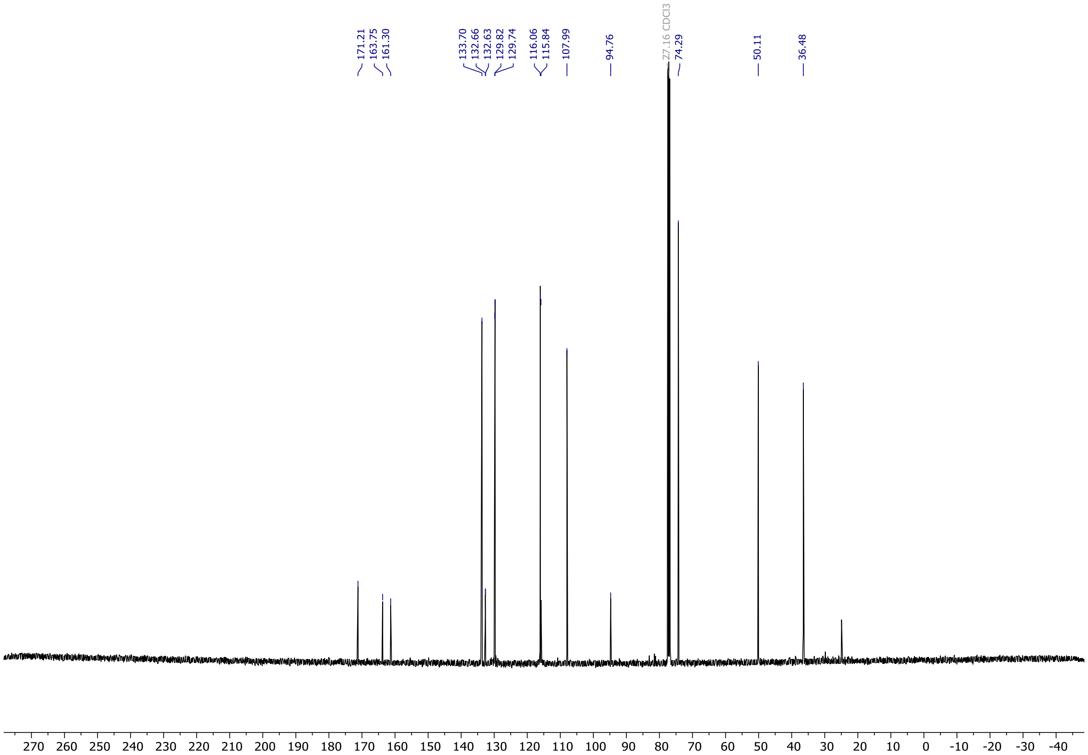

27: ^{19}F NMR (282 MHz, $CDCl_3$)

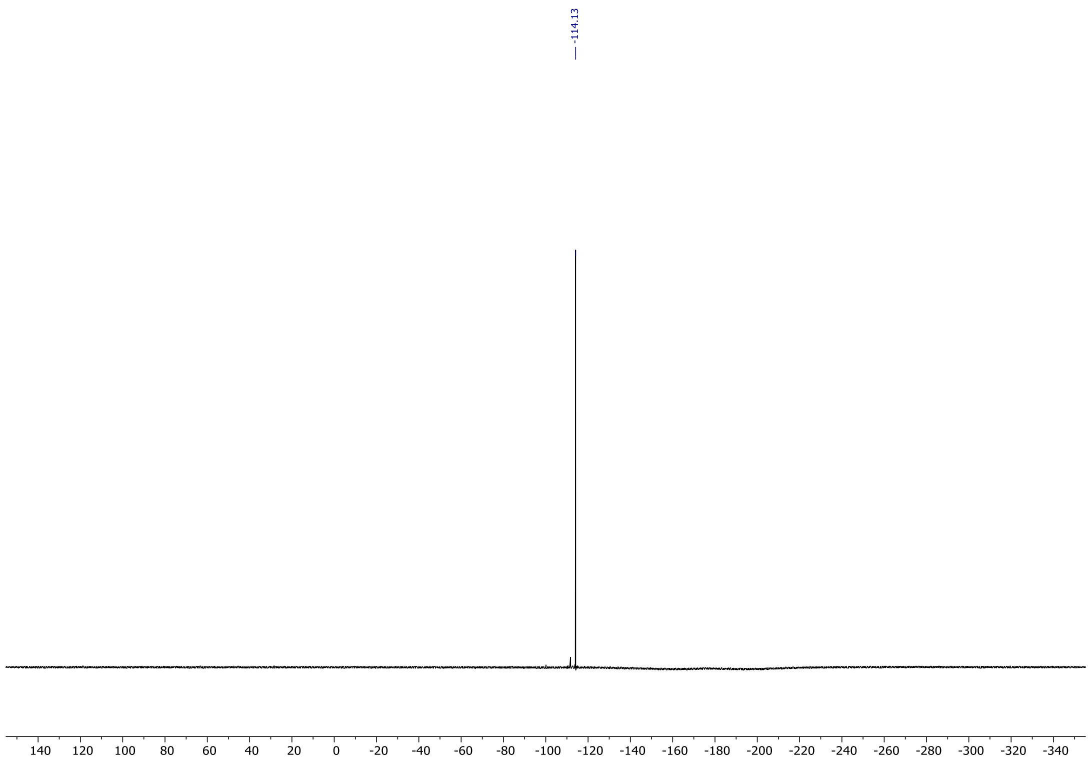

28: ^{1}H NMR (400 MHz, $CDCl_3$)

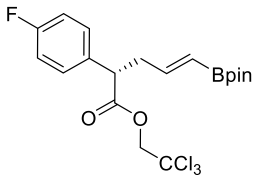

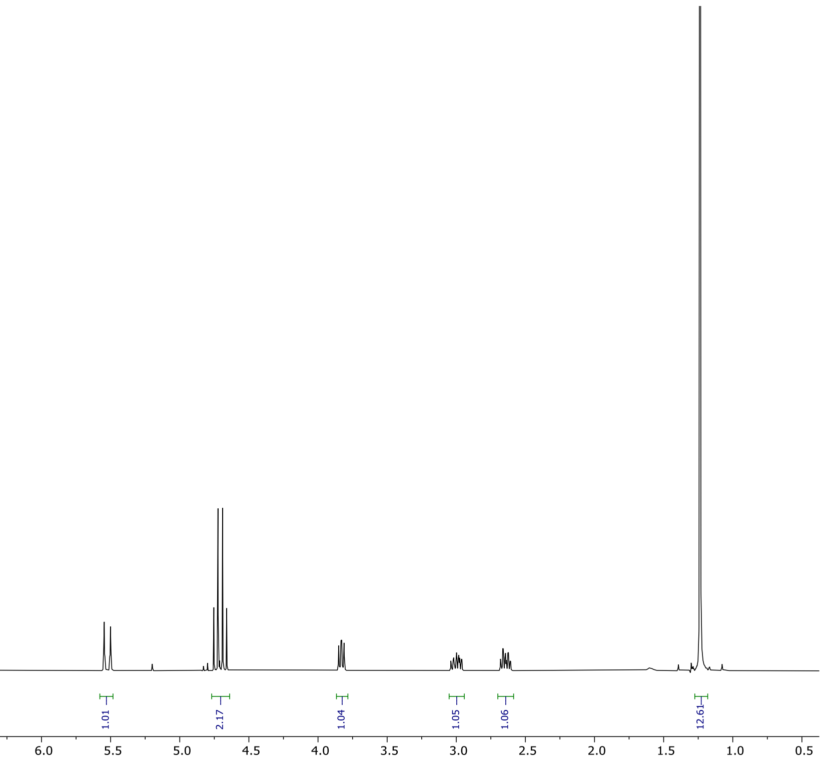

28: ^{13}C NMR (101 MHz, $CDCl_3$)

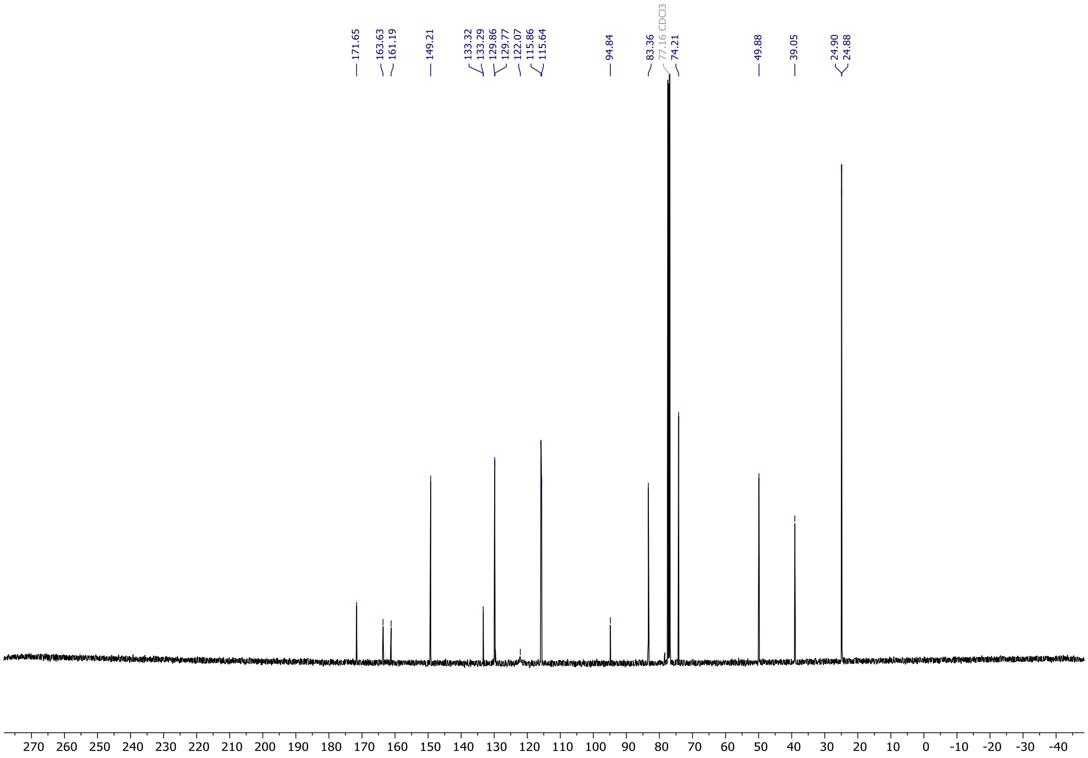

28: ^{19}F NMR (282 MHz, $CDCl_3$)

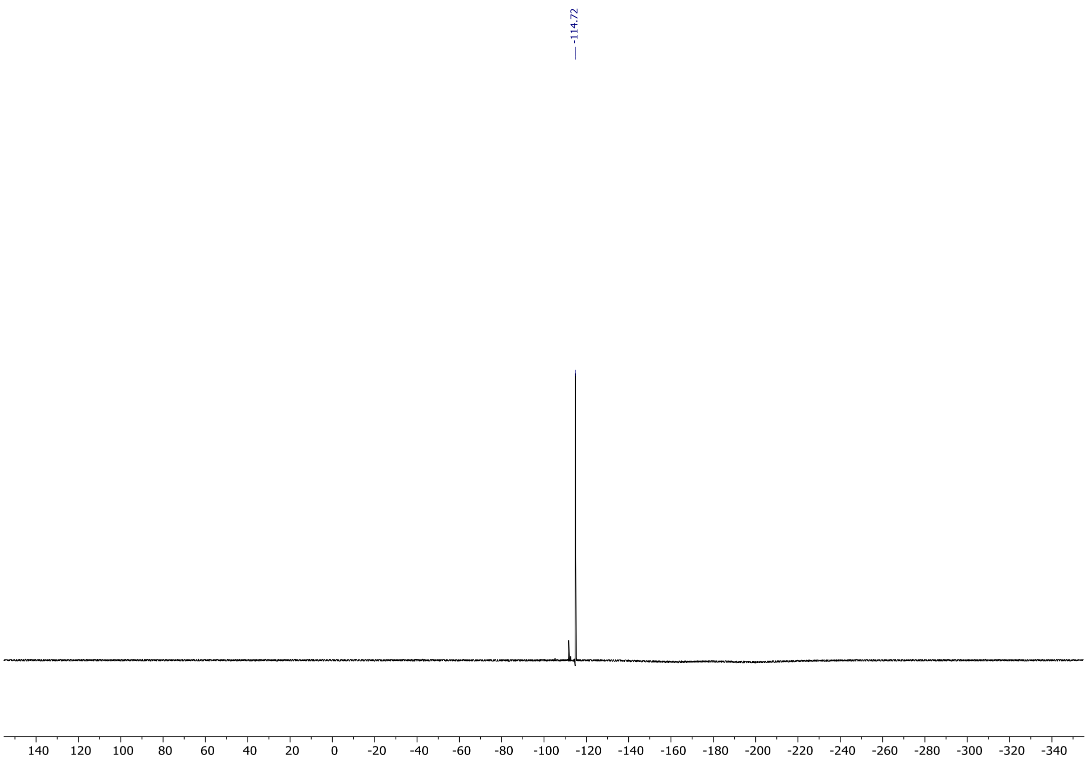

28: ^{11}B NMR (128 MHz, $CDCl_3$)

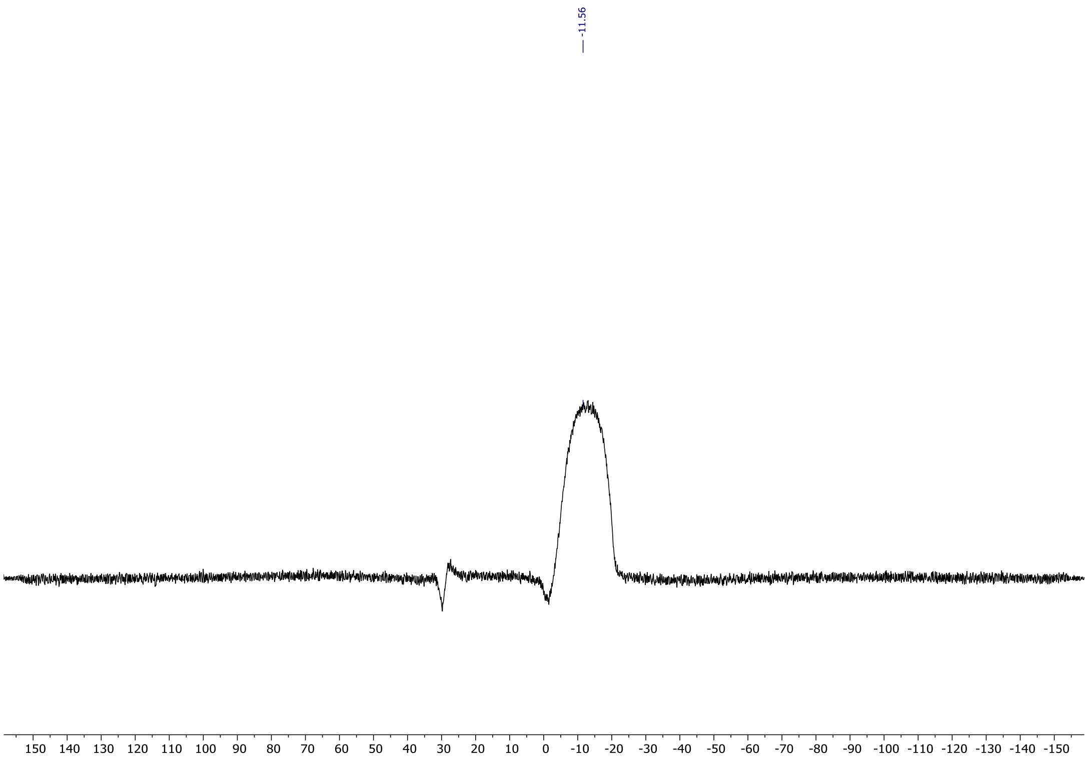

29: ^{1}H NMR (400 MHz, $CDCl_3$):

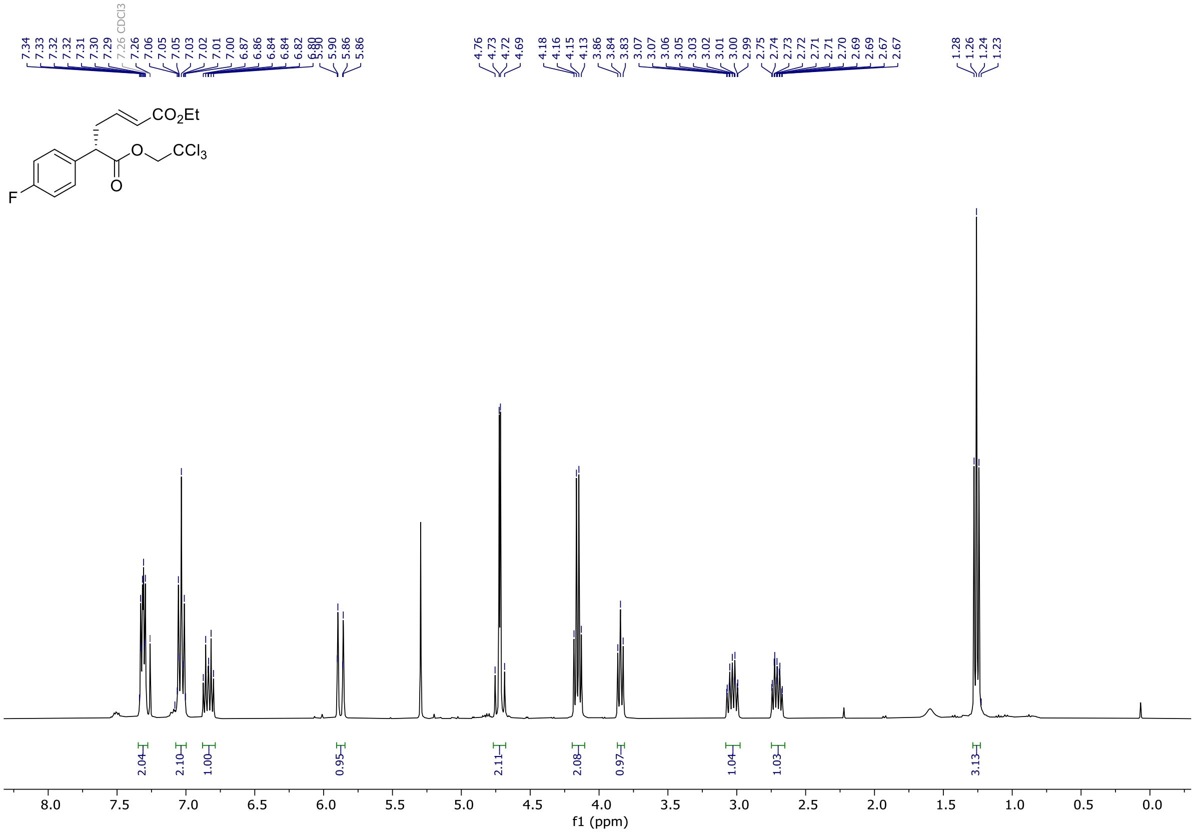

29: ^{13}C NMR (101 MHz, $CDCl_3$):

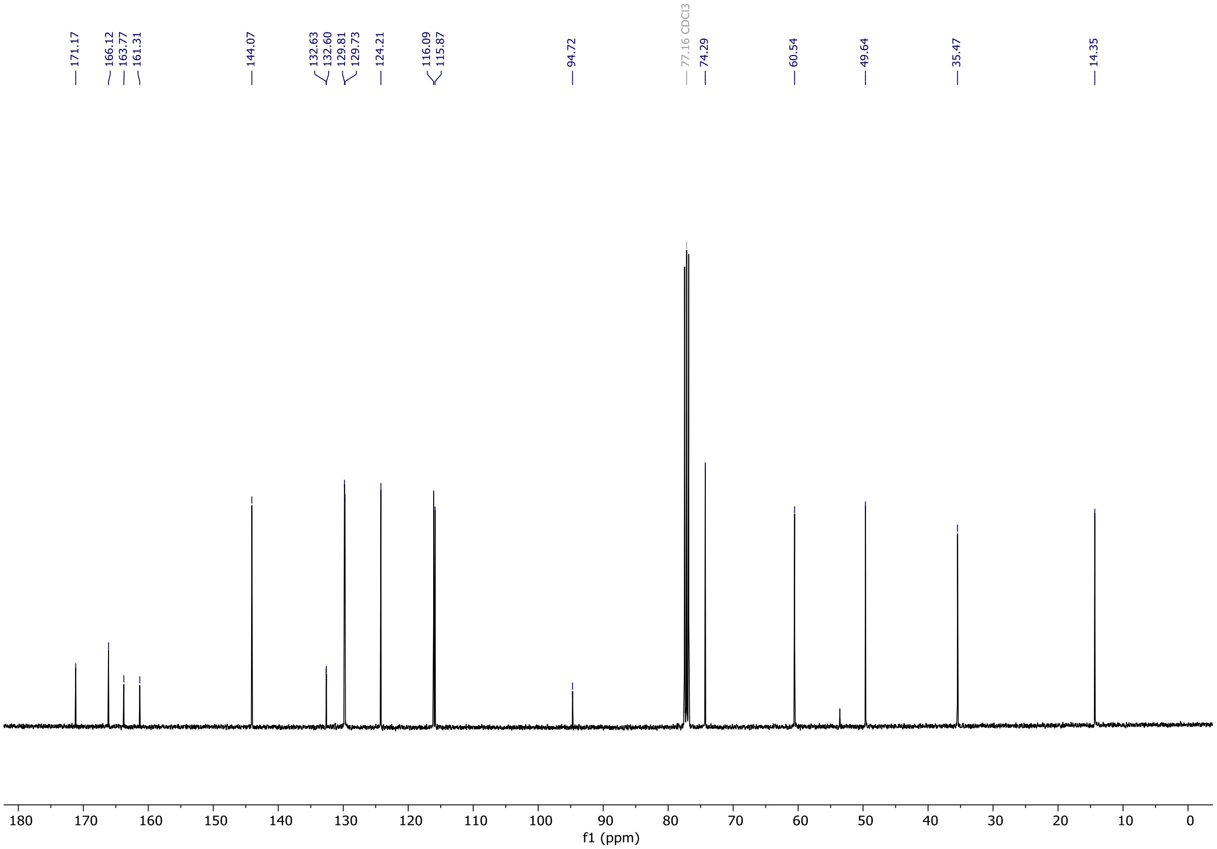

29: ^{19}F NMR (282 MHz, $CDCl_3$):

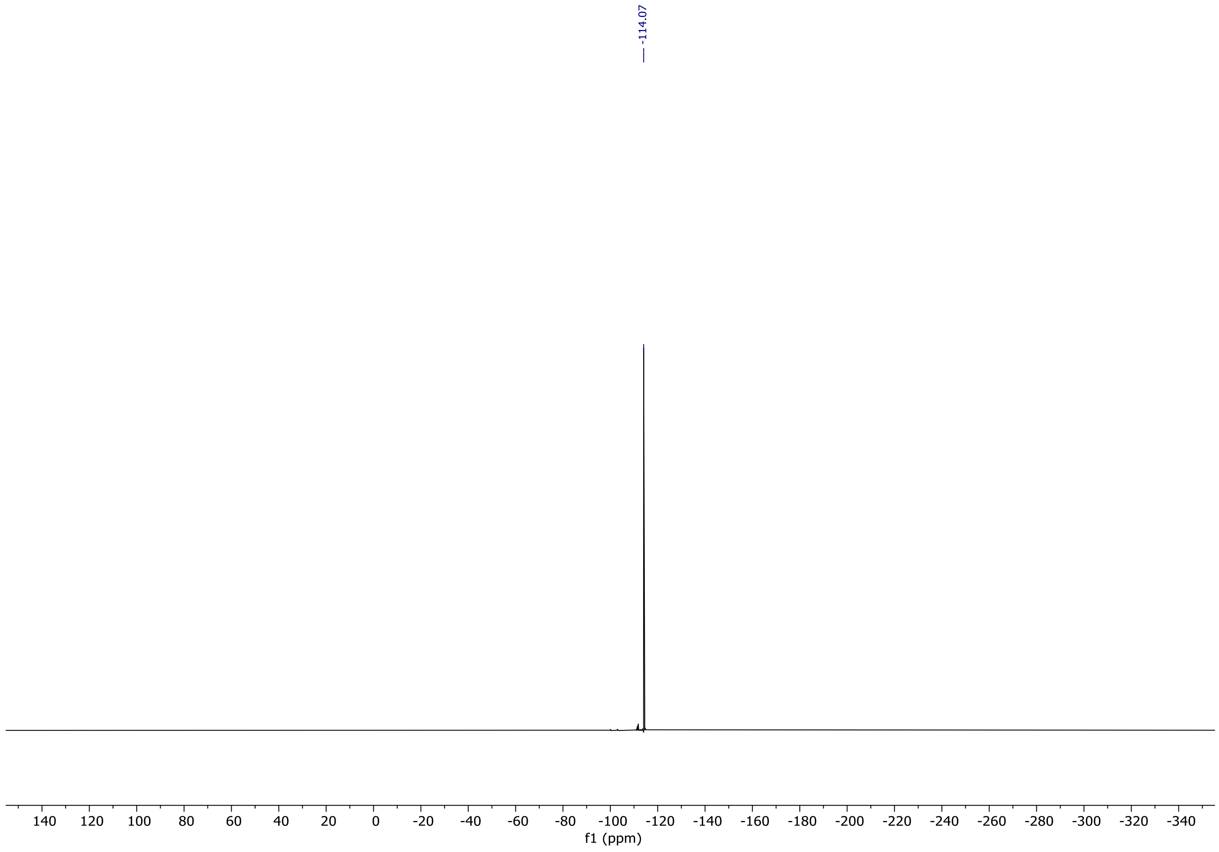

30: ^{1}H NMR (400 MHz, $CDCl_3$)

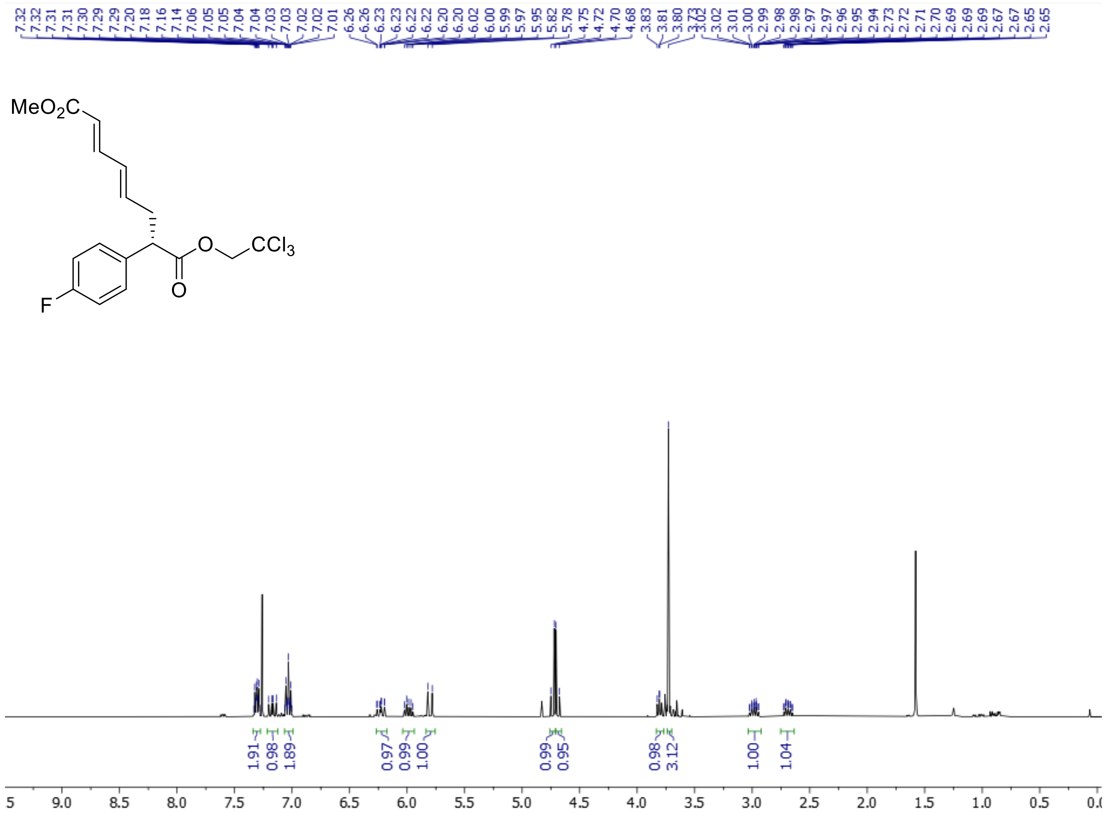

30: ^{13}C NMR (101 MHz, $CDCl_3$)

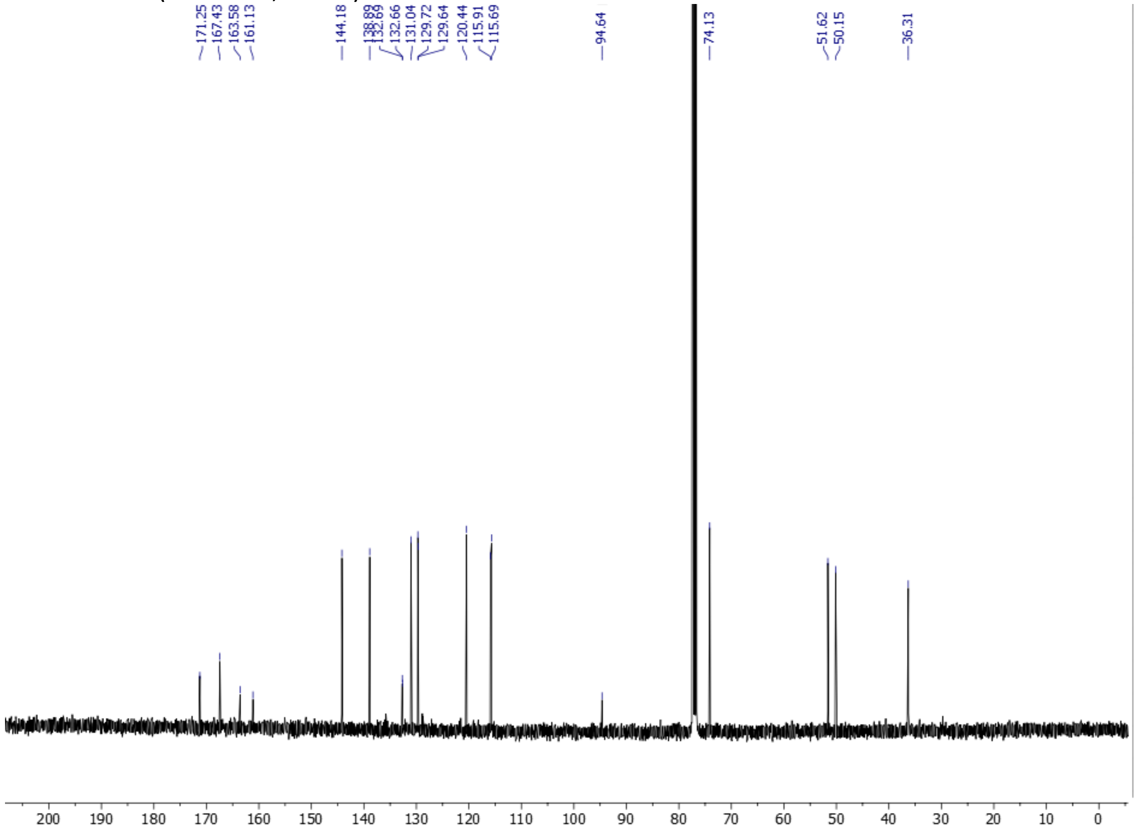

30: ^{19}F NMR (282 MHz, $CDCl_3$)

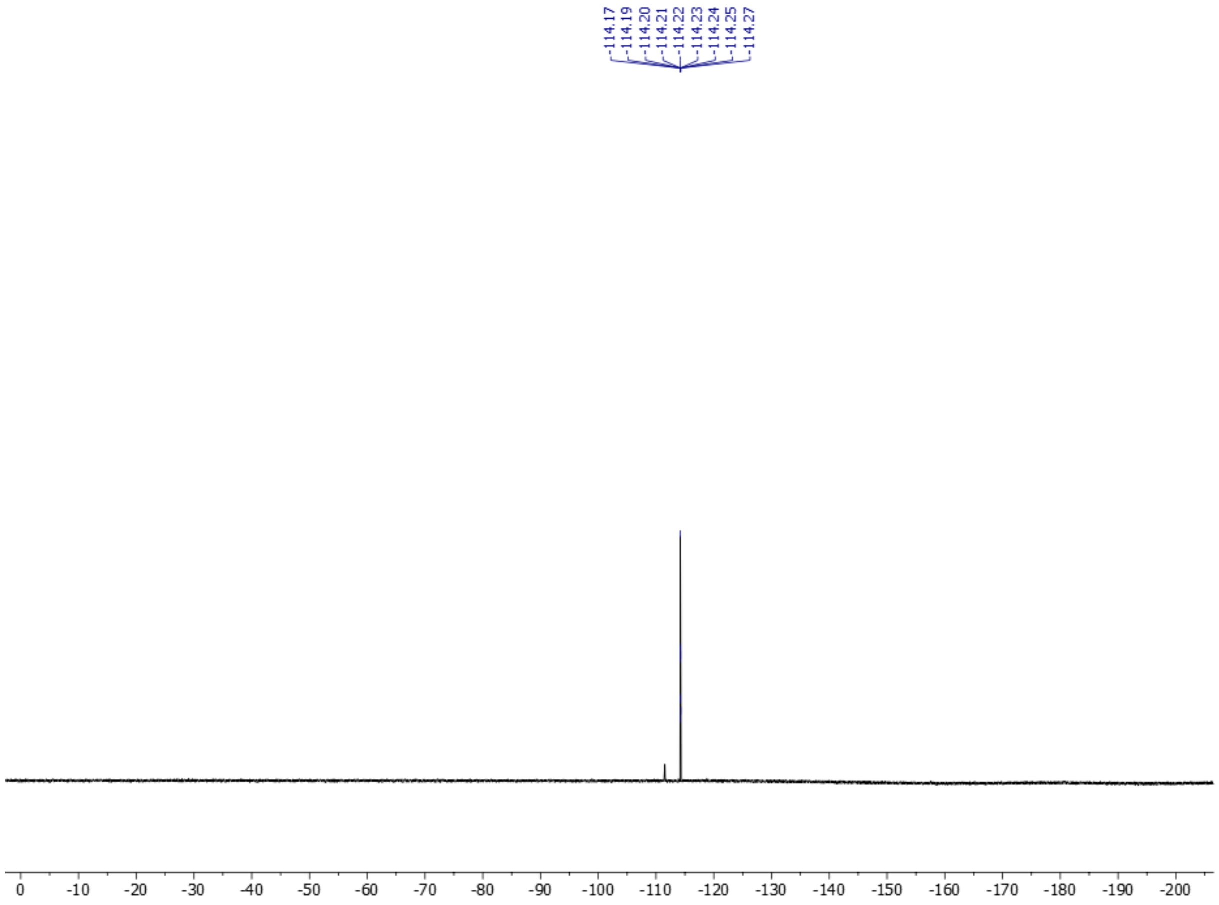

31a: ^{1}H NMR (400 MHz, $CDCl_3$) (the sample contained compound **11** as inseparable impurity, ca. 25%)

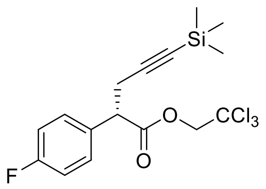

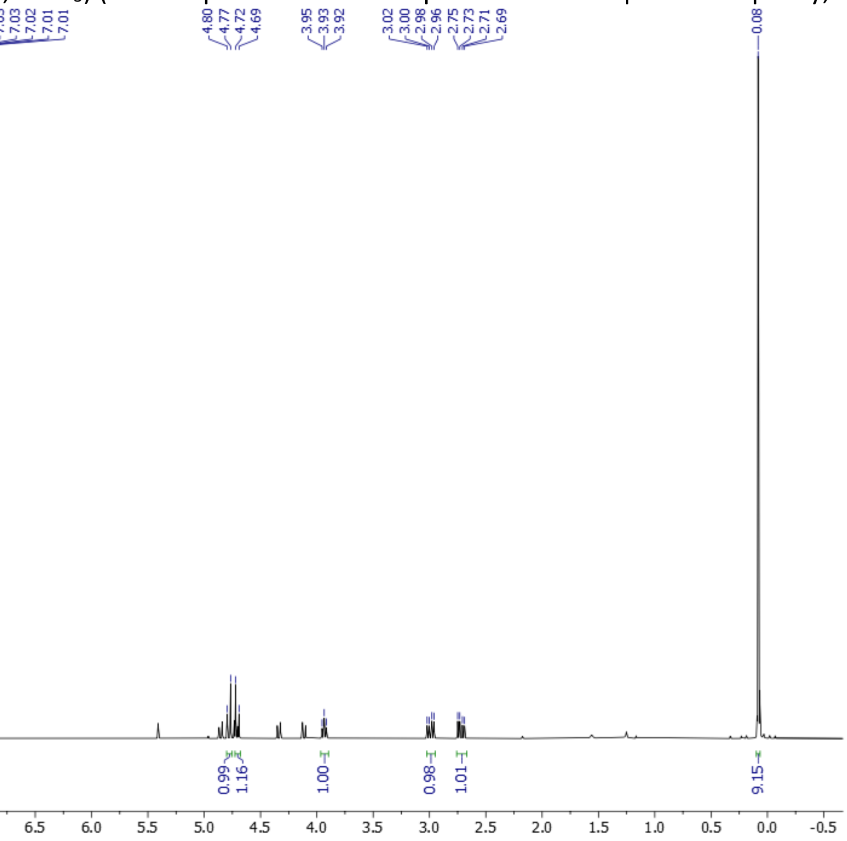

31a: ^{13}C NMR (101 MHz, $CDCl_3$) (the sample contained compound **11** as inseparable impurity, ca. 25%)

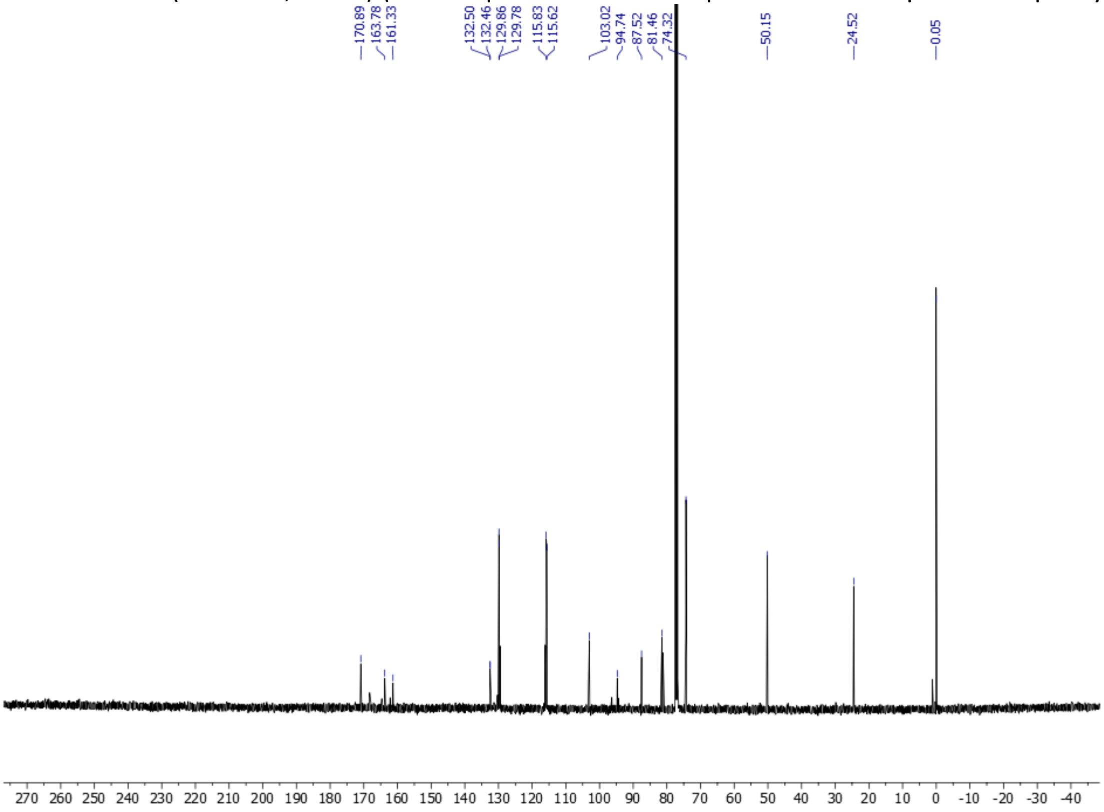

31a: ^{19}F NMR (282 MHz, $CDCl_3$) (the sample contained compound **11** as inseparable impurity, ca. 25%)

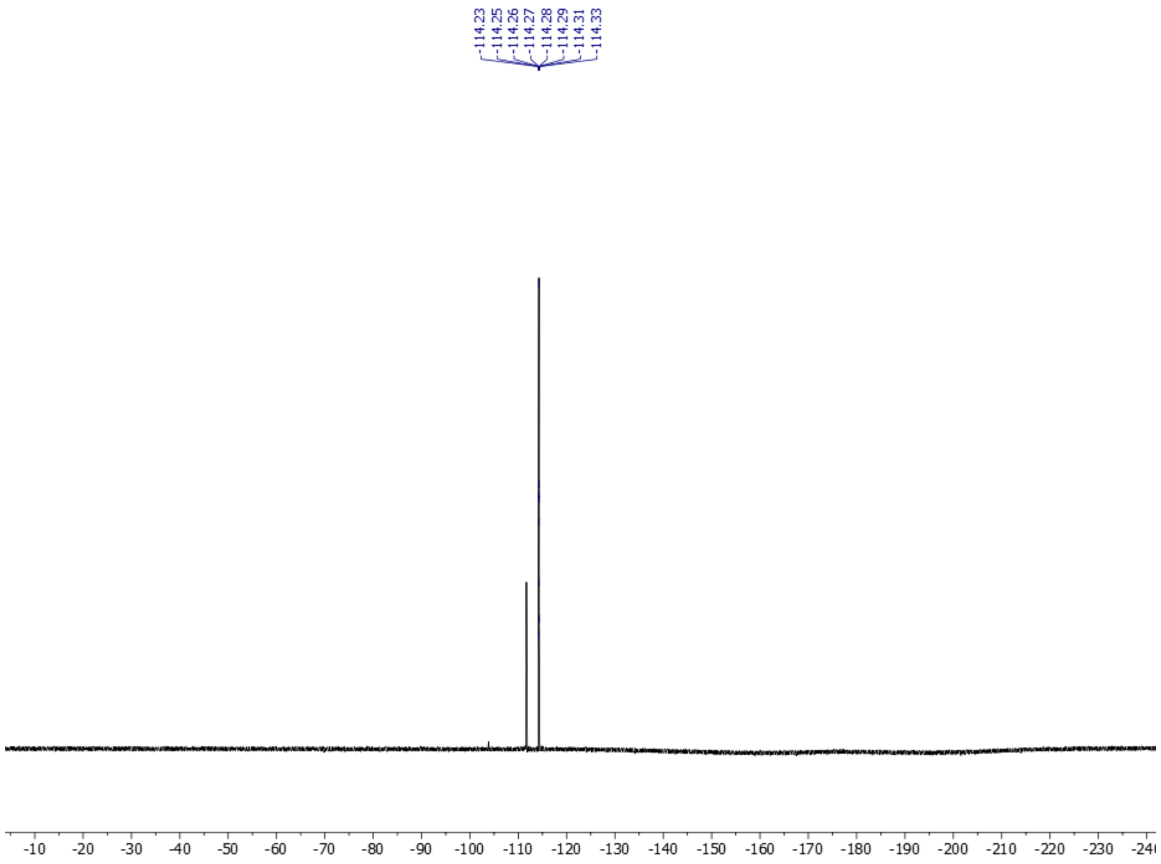

31b: ^{1}H NMR (400 MHz, $CDCl_3$) (the sample contained compound **11** as inseparable impurity, ca. 10%)

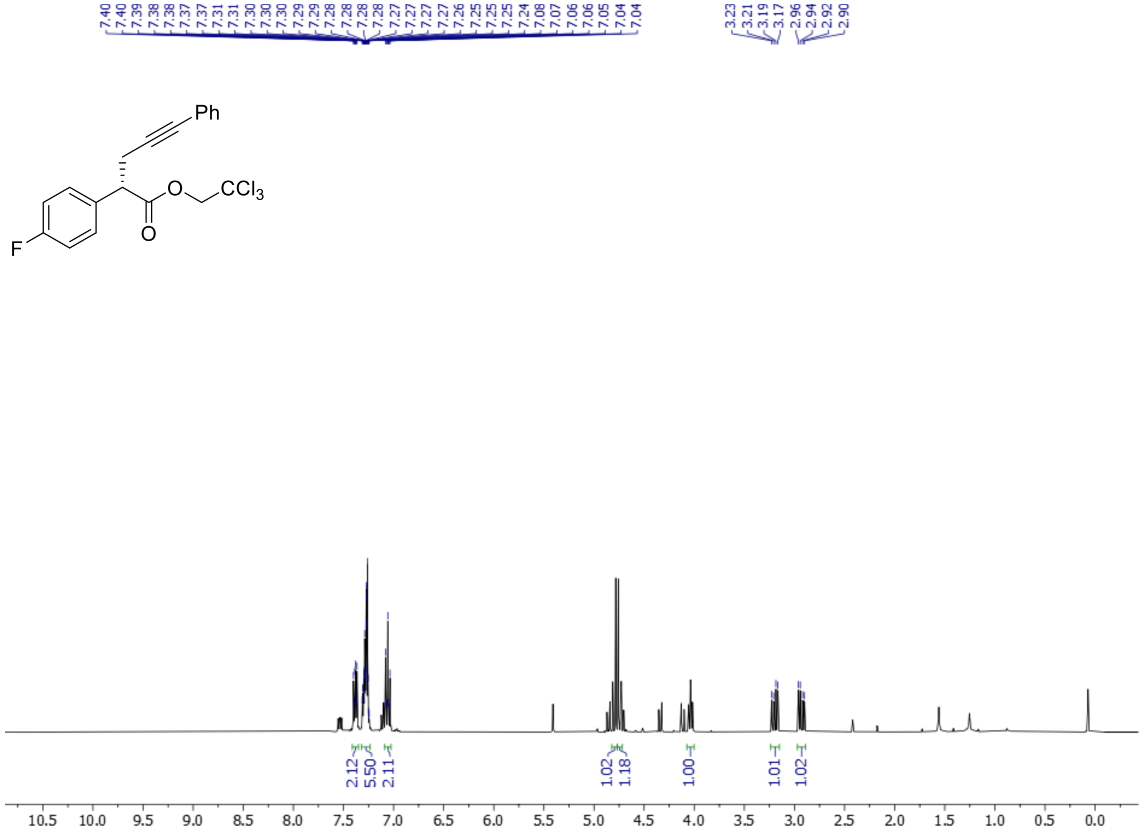

31b: ^{13}C NMR (101 MHz, $CDCl_3$) (the sample contained compound **11** as inseparable impurity, ca. 10%)

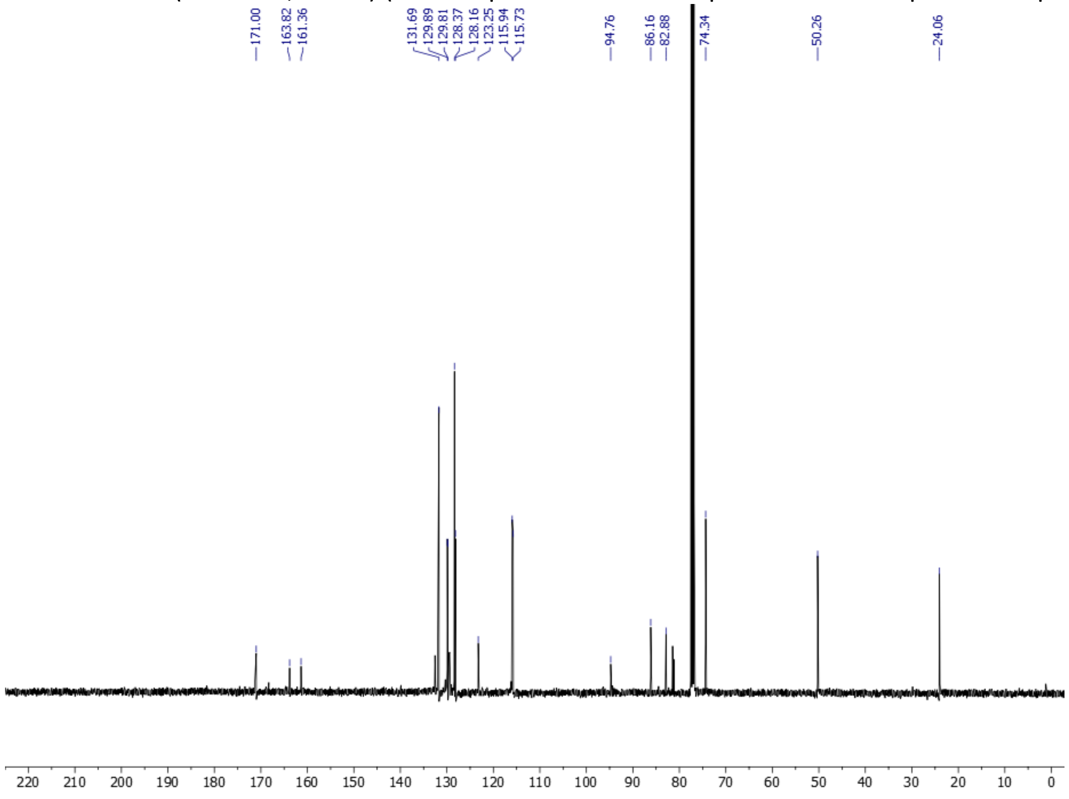

31b: ^{19}F NMR (282 MHz, $CDCl_3$) (the sample contained compound **11** as inseparable impurity, ca. 10%)

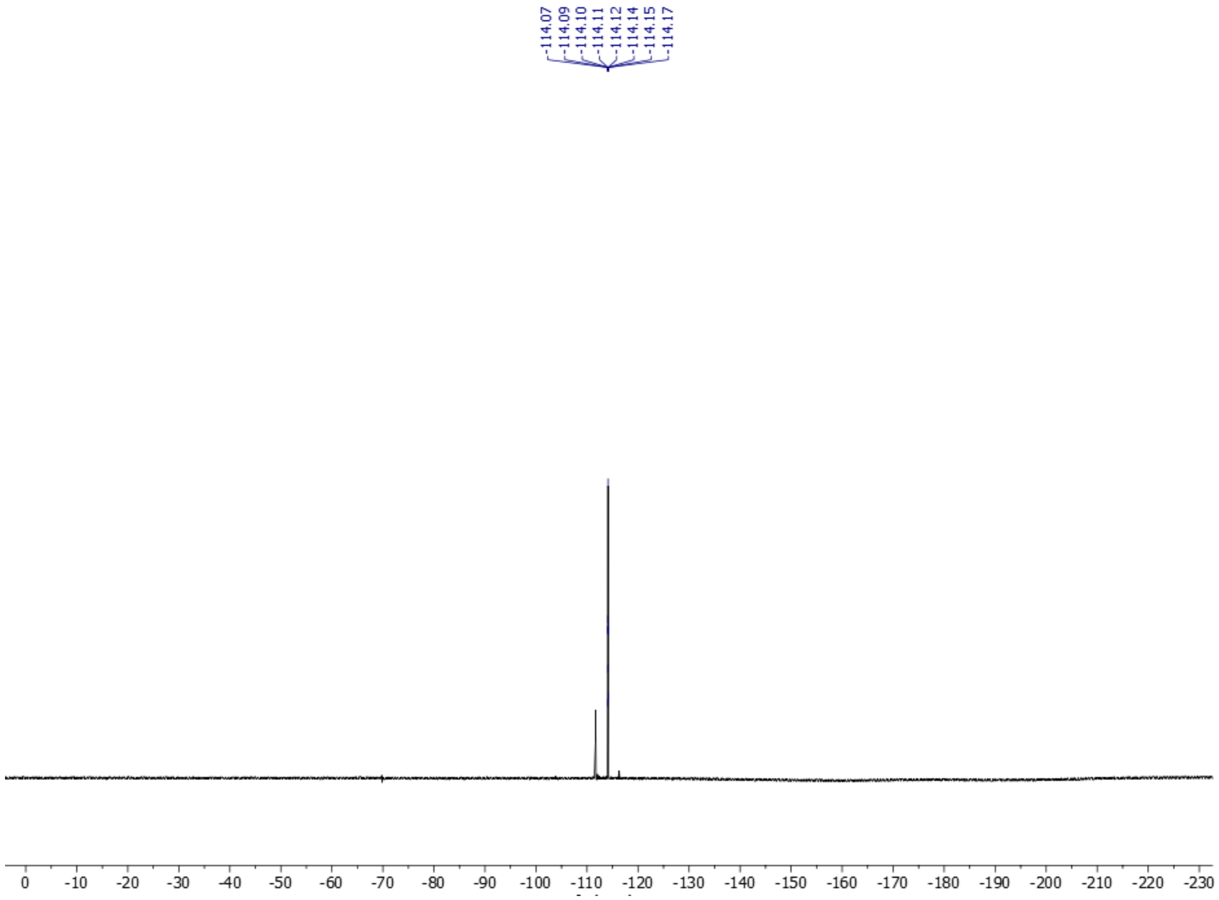

33: ^{1}H NMR (600 MHz, $CDCl_3$); mixture of diastereomers

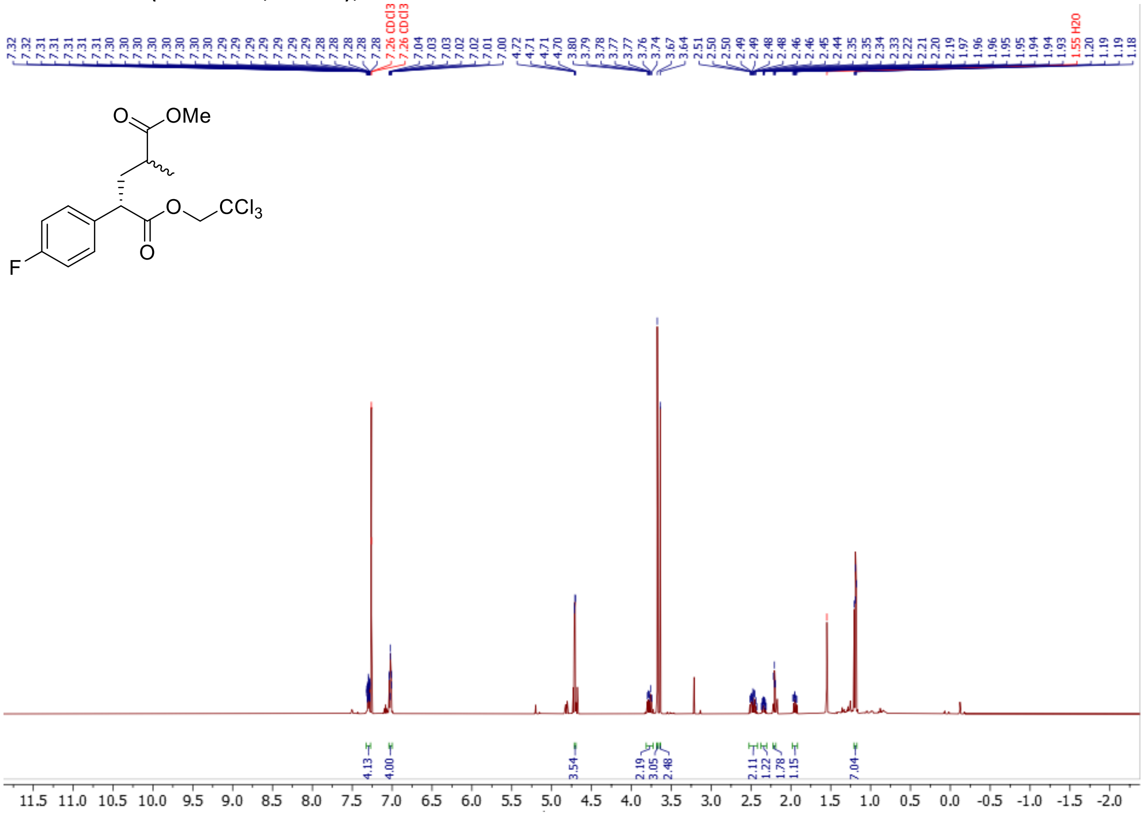

33: ^{13}C NMR (151 MHz, $CDCl_3$); mixture of diastereomers

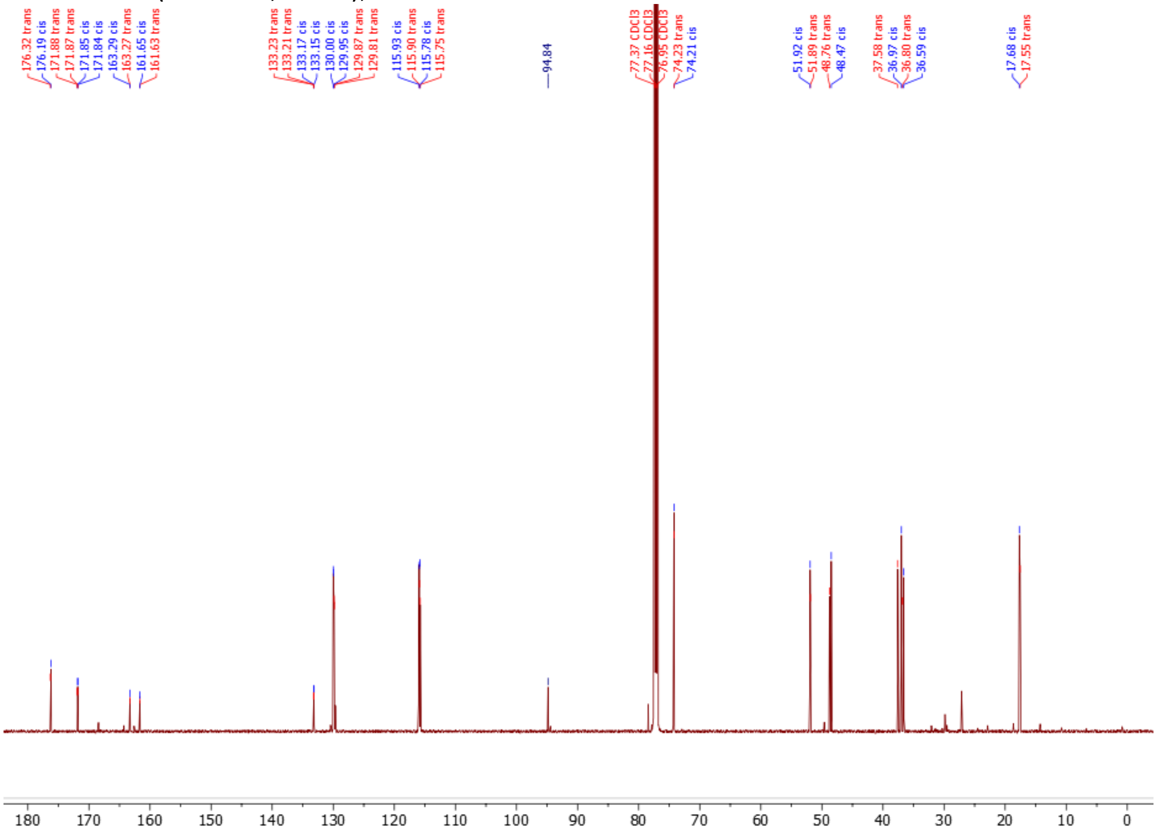

33: ^{19}F NMR (565 MHz, $CDCl_3$)

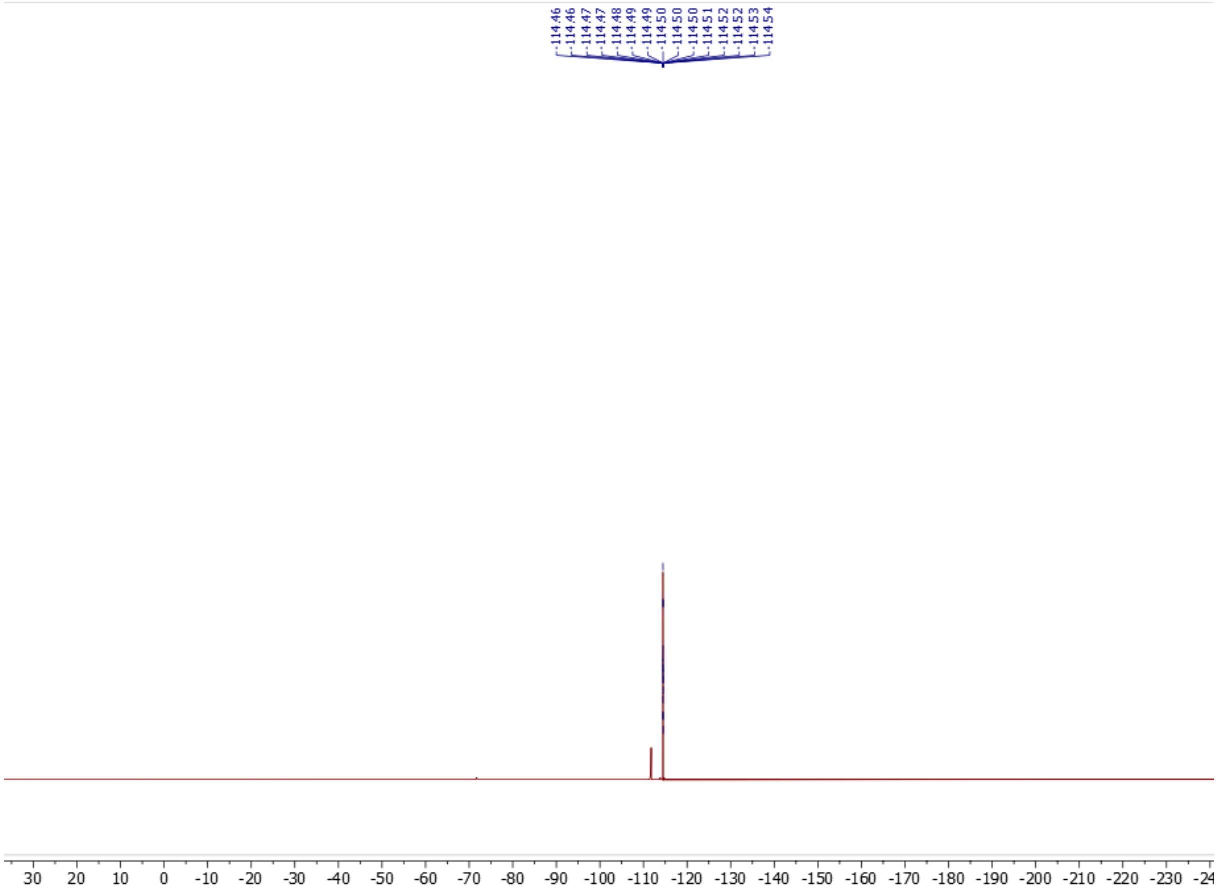

34a: ^{1}H NMR (400 MHz, $CDCl_3$):

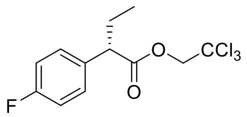

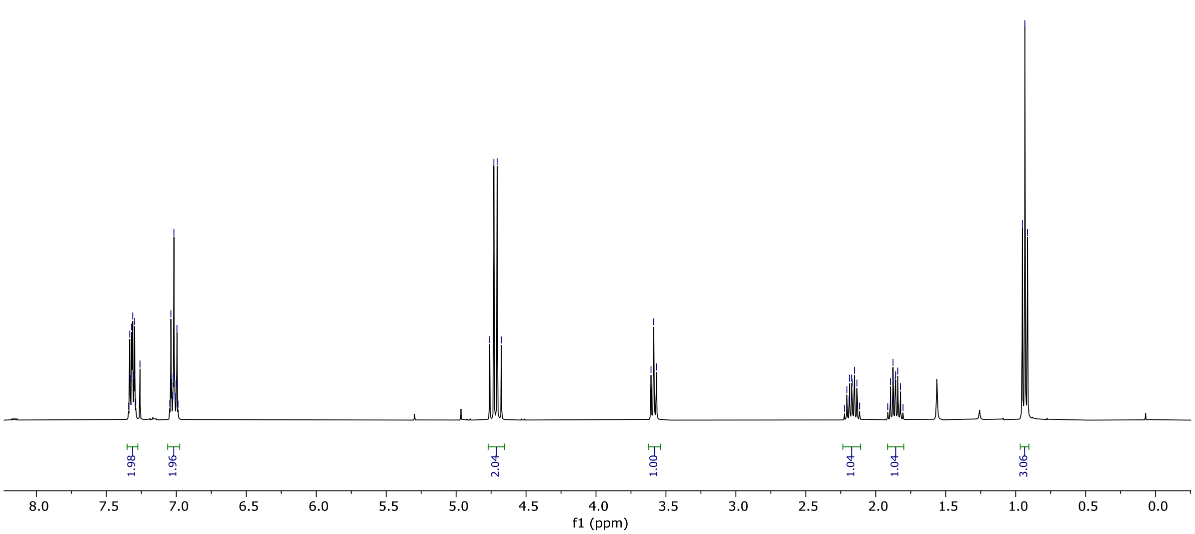

34a: ^{13}C NMR (101 MHz, $CDCl_3$):

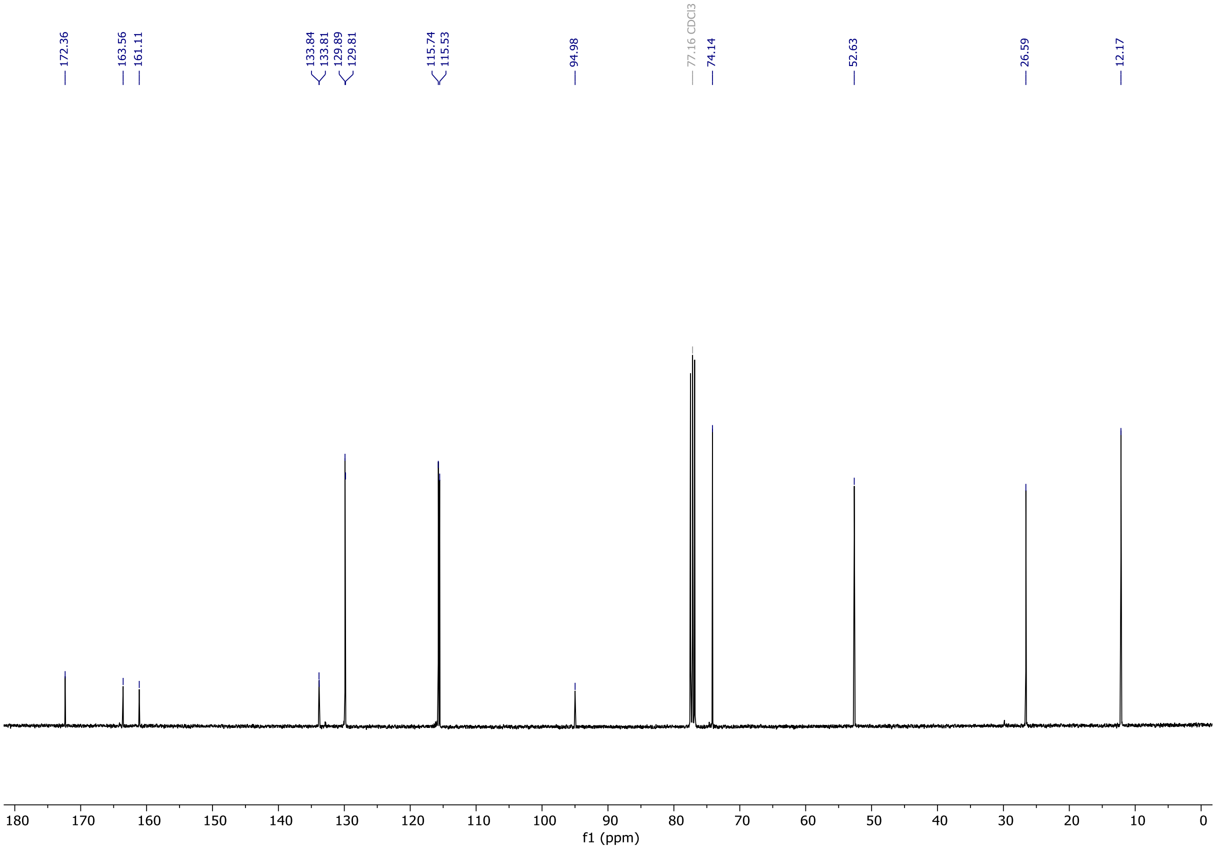

34a: ^{19}F NMR (282 MHz, $CDCl_3$):

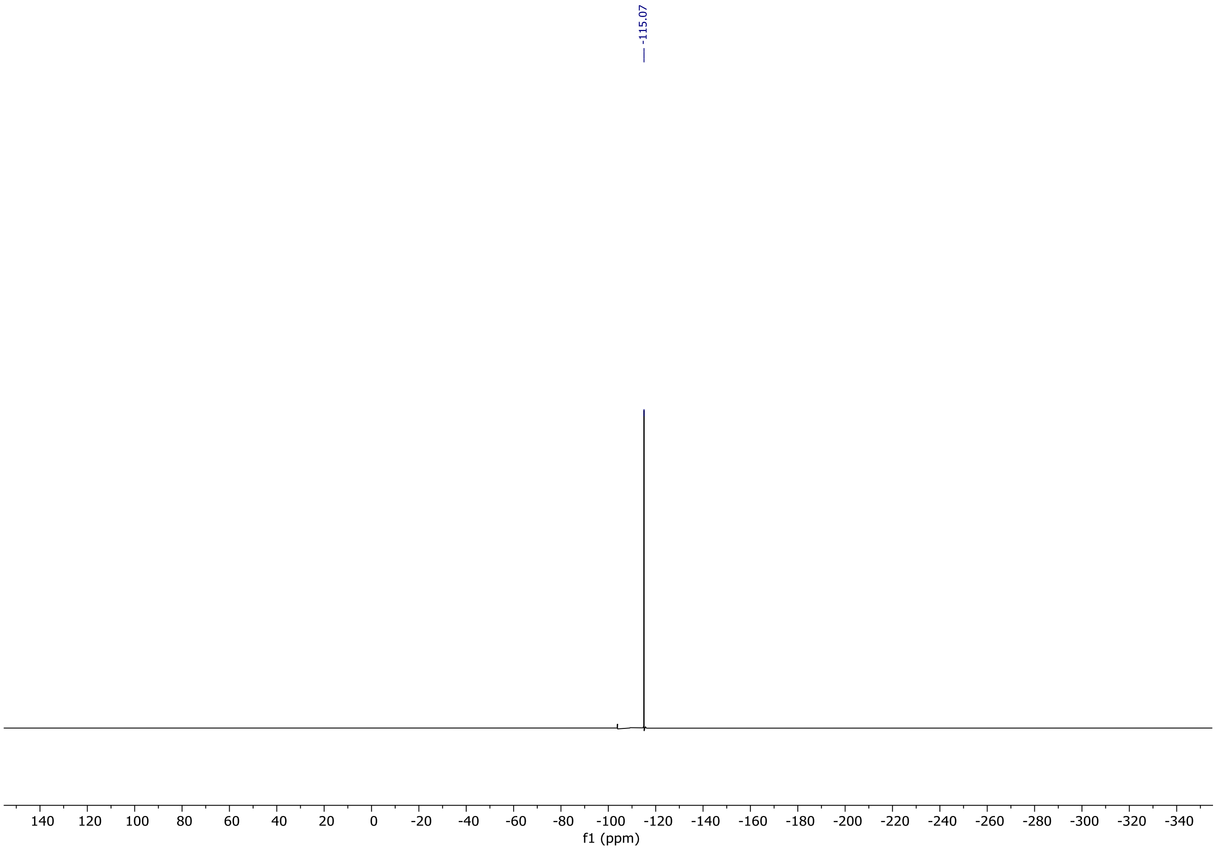

34b: ^{1}H NMR (400 MHz, $CDCl_3$):

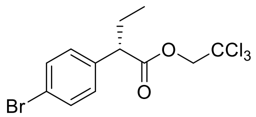

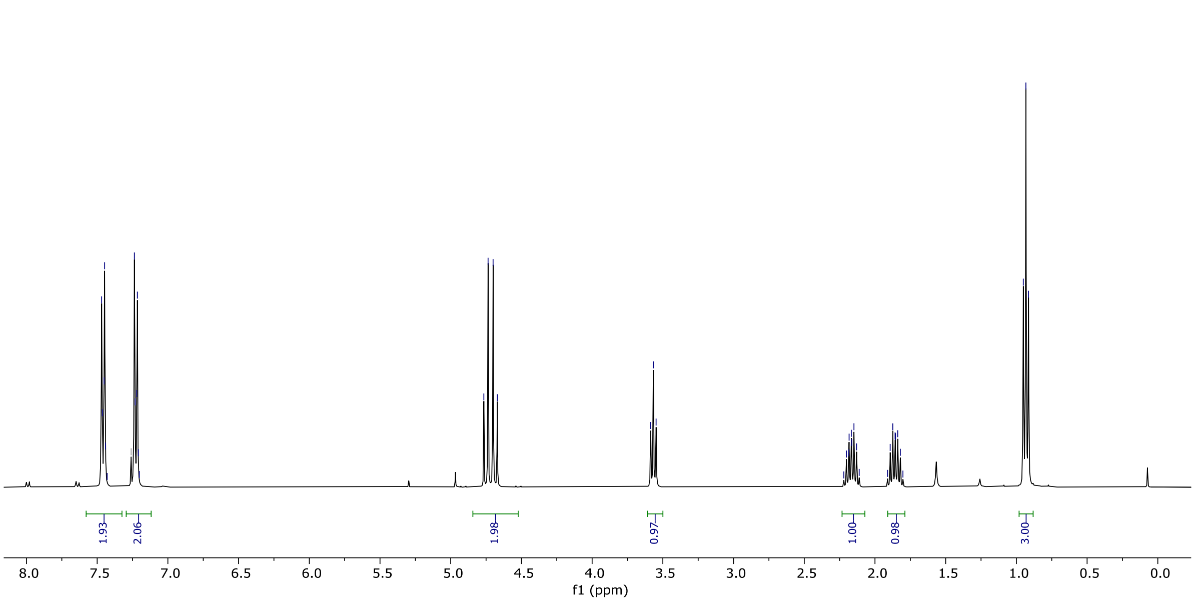

34b: ^{13}C NMR (101 MHz, $CDCl_3$):

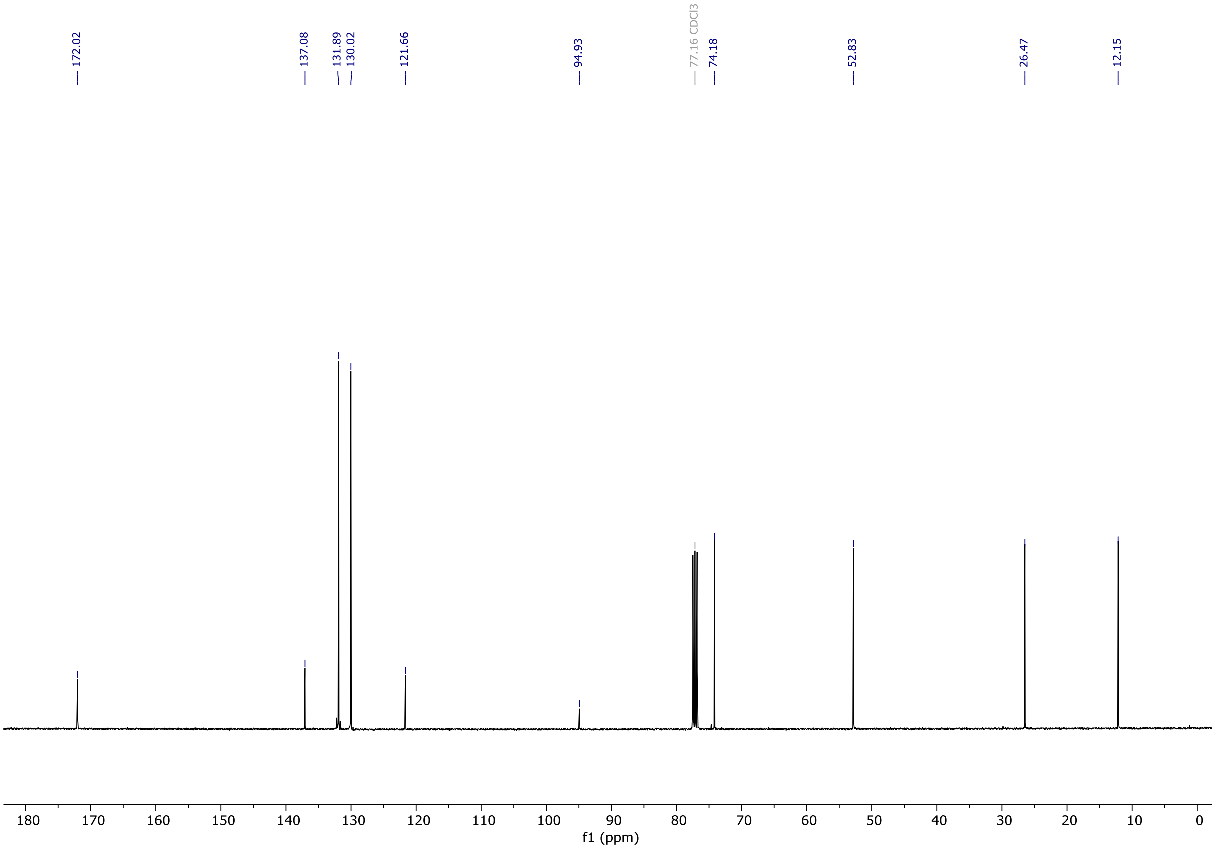

34c: ^{1}H NMR (400 MHz, $CDCl_3$):

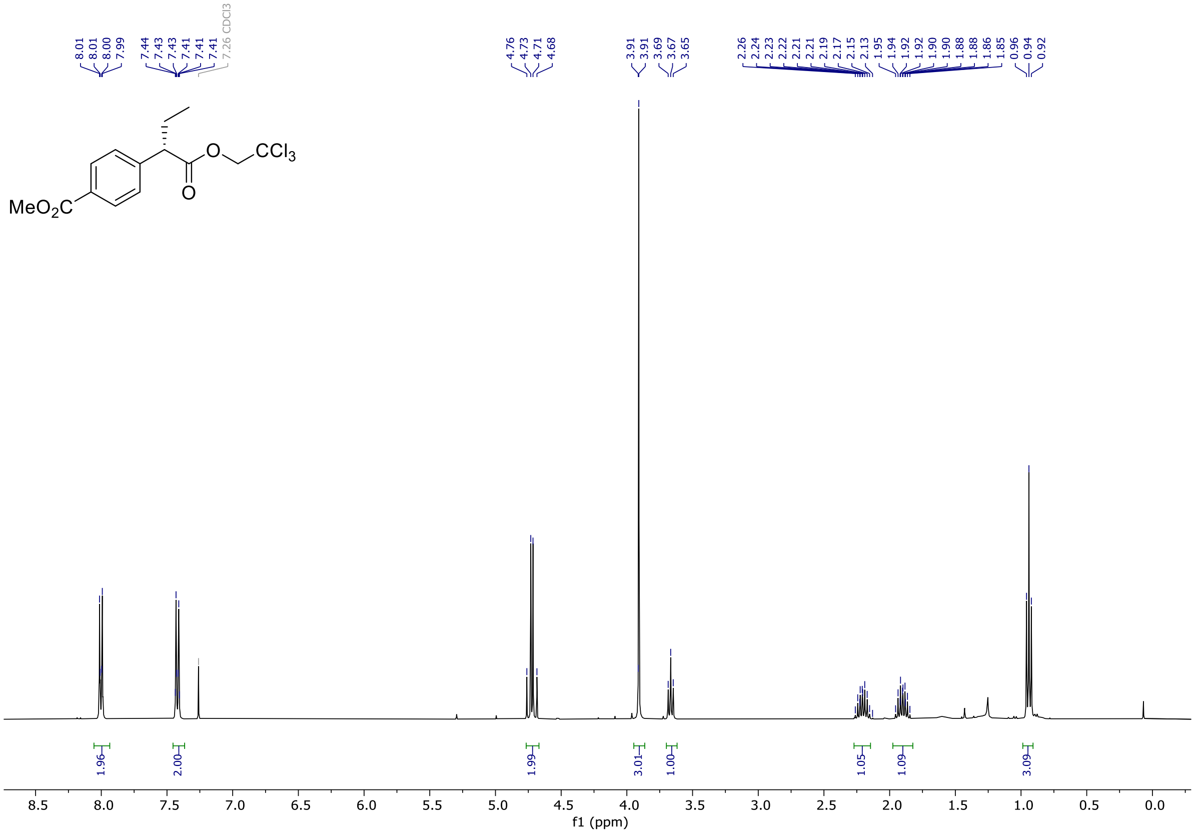

34c: ^{13}C NMR (101 MHz, $CDCl_3$):

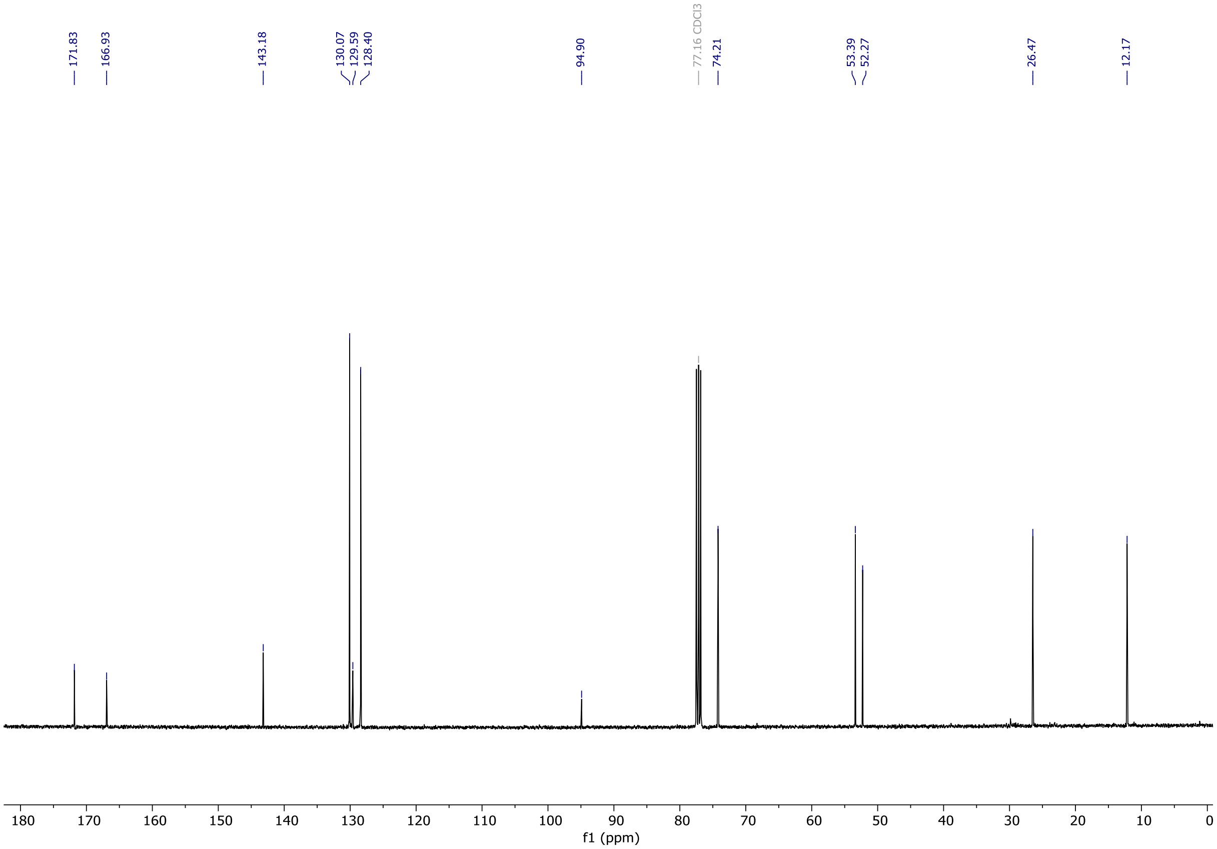

35: ^{1}H NMR (400 MHz, $CDCl_3$):

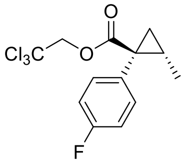

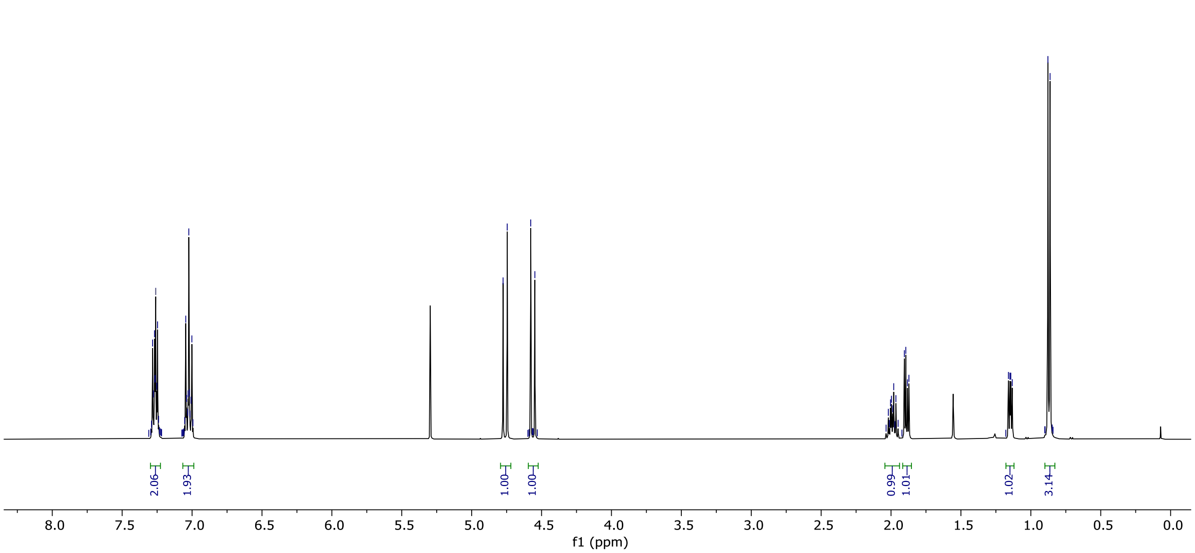

35: ^{13}C NMR (101 MHz, $CDCl_3$):

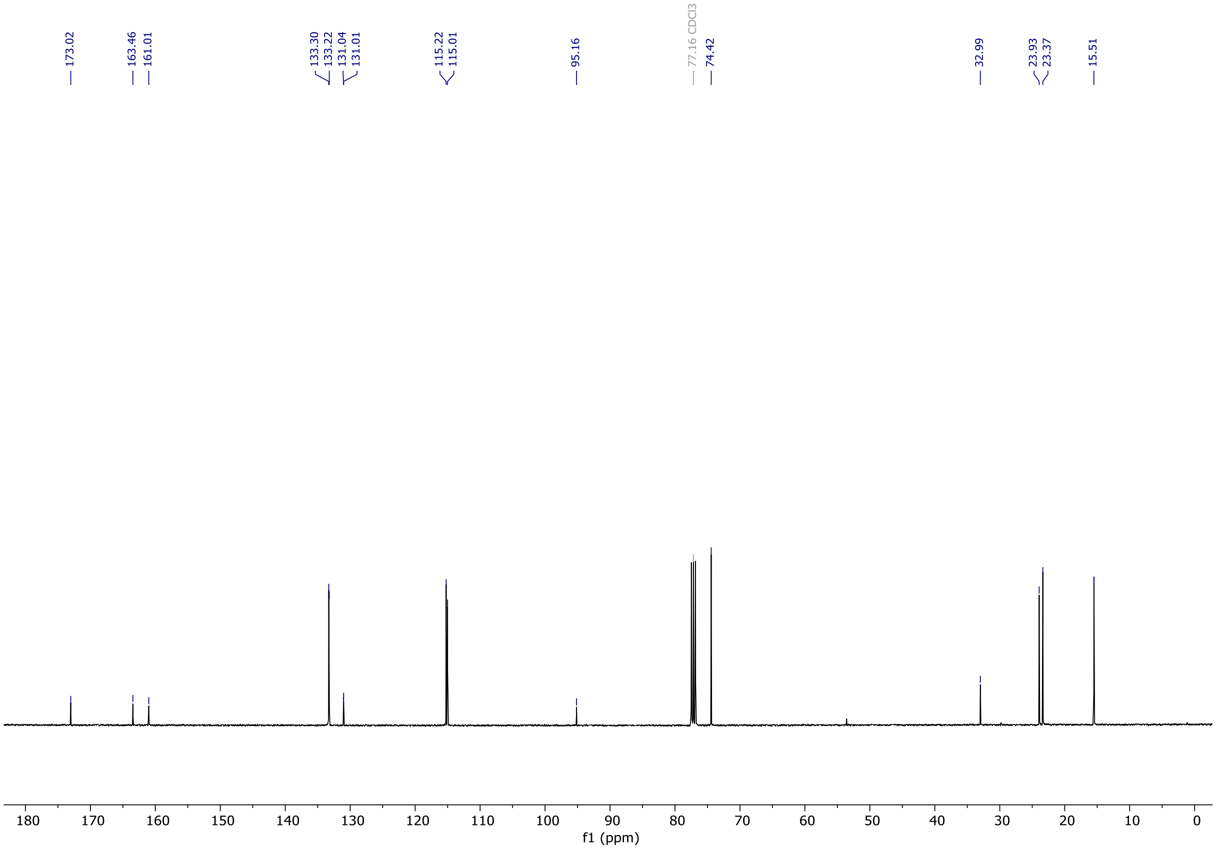

35: ^{19}F NMR (282 MHz, $CDCl_3$):

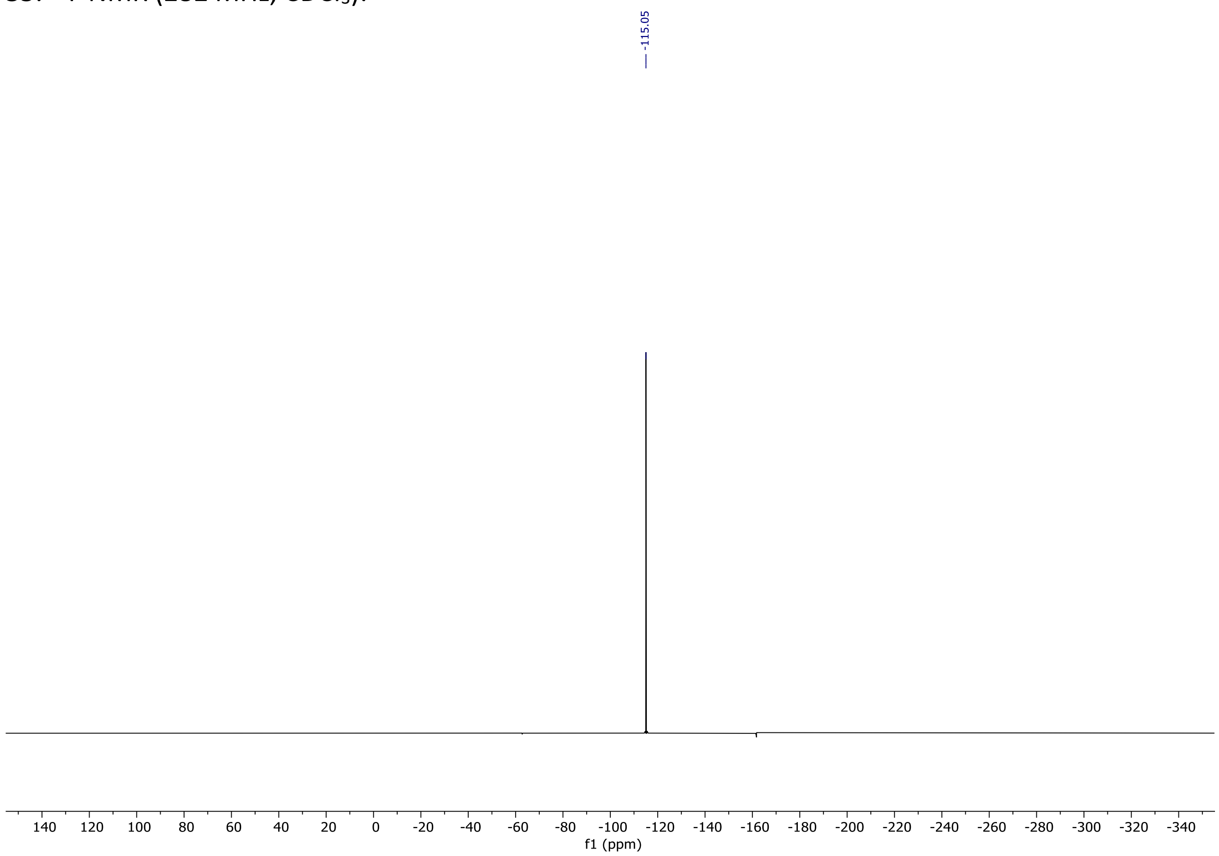

37a: ^{1}H NMR (400 MHz, $CDCl_3$); mixture of diastereomers (dr = 95:5)

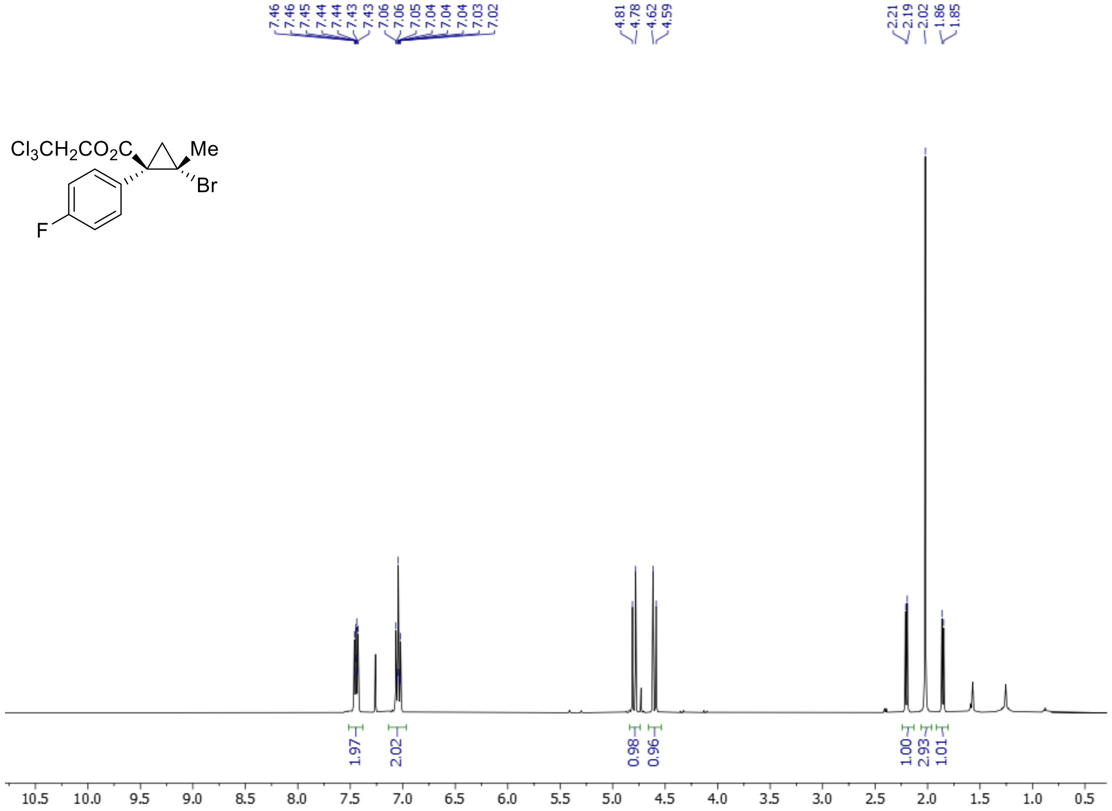

37a: ^{13}C NMR (101 MHz, $CDCl_3$); mixture of diastereomers (dr = 95:5)

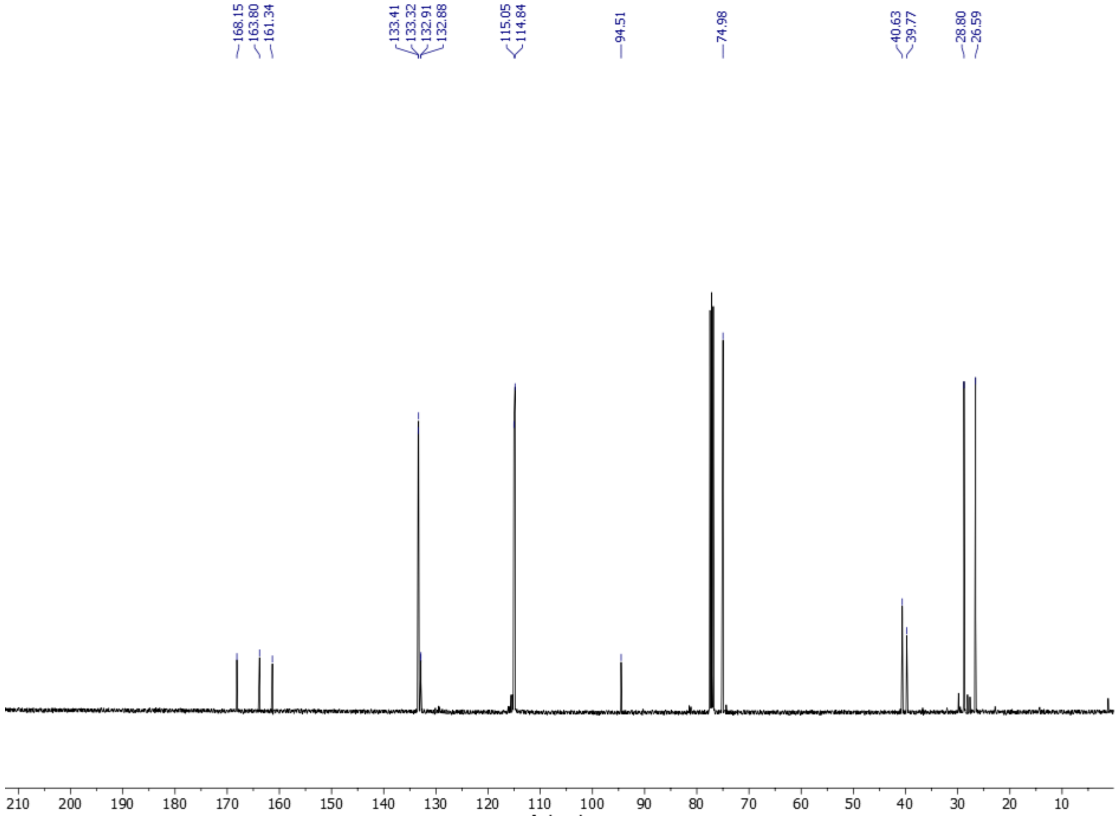

37a: ^{19}F NMR (282 MHz, $CDCl_3$)

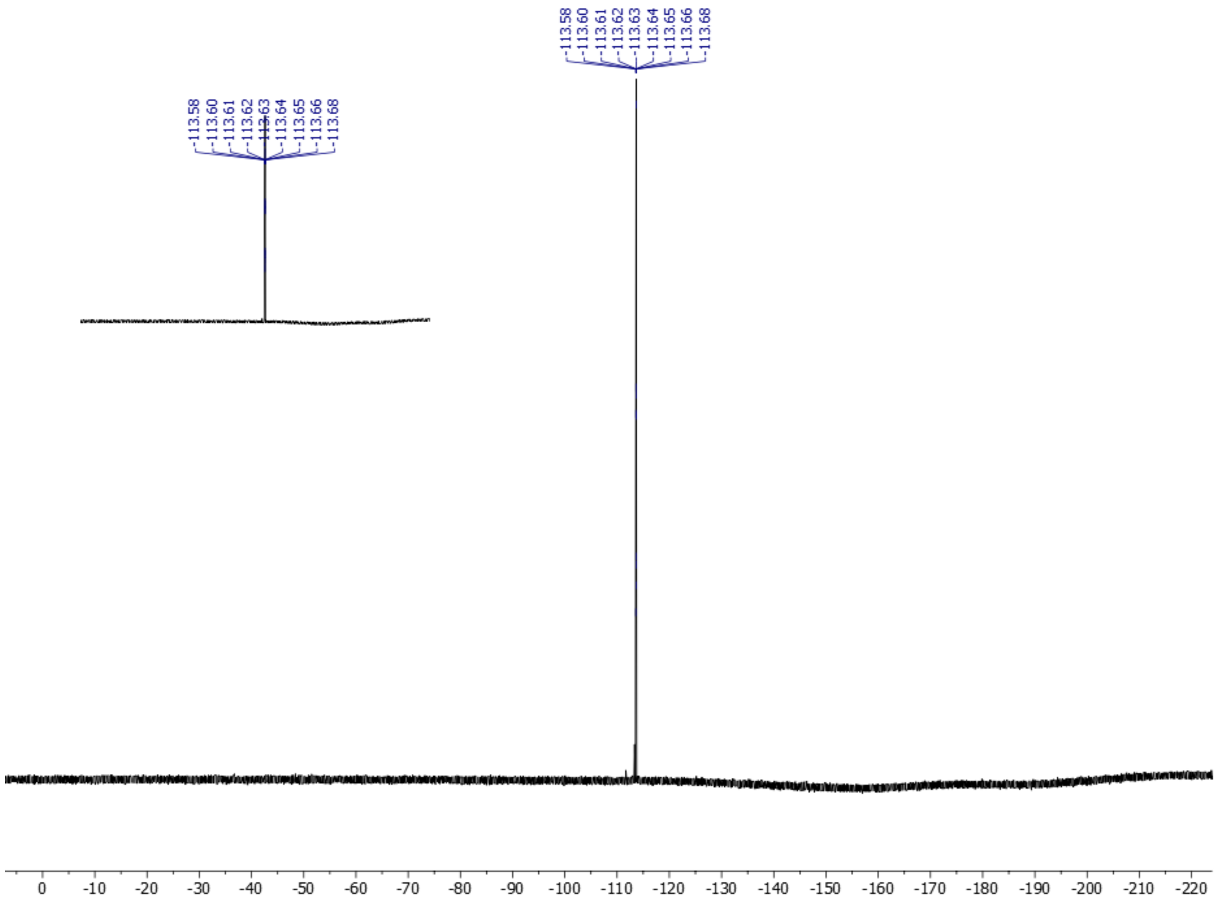

37b: ^{1}H NMR (400 MHz, $CDCl_3$); mixture of diastereomers

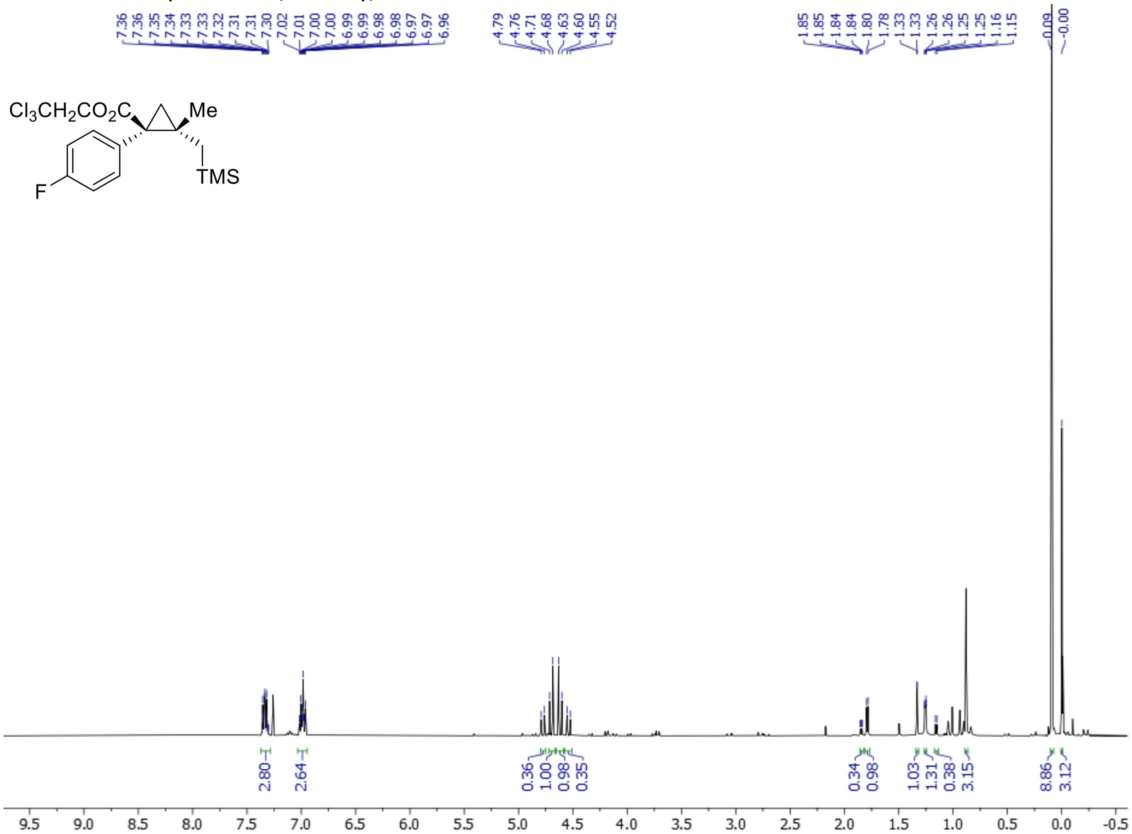

37b: ^{13}C NMR (101 MHz, $CDCl_3$); mixture of diastereomers

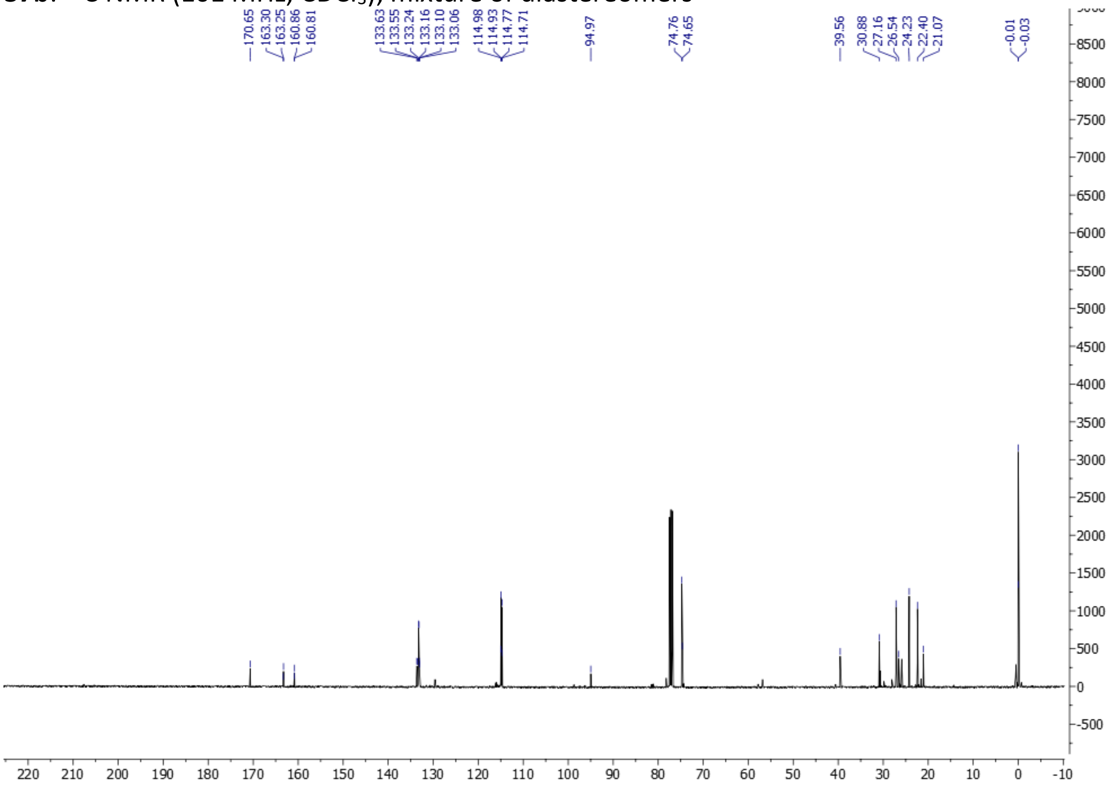

37b: ^{19}F NMR (282 MHz, $CDCl_3$)

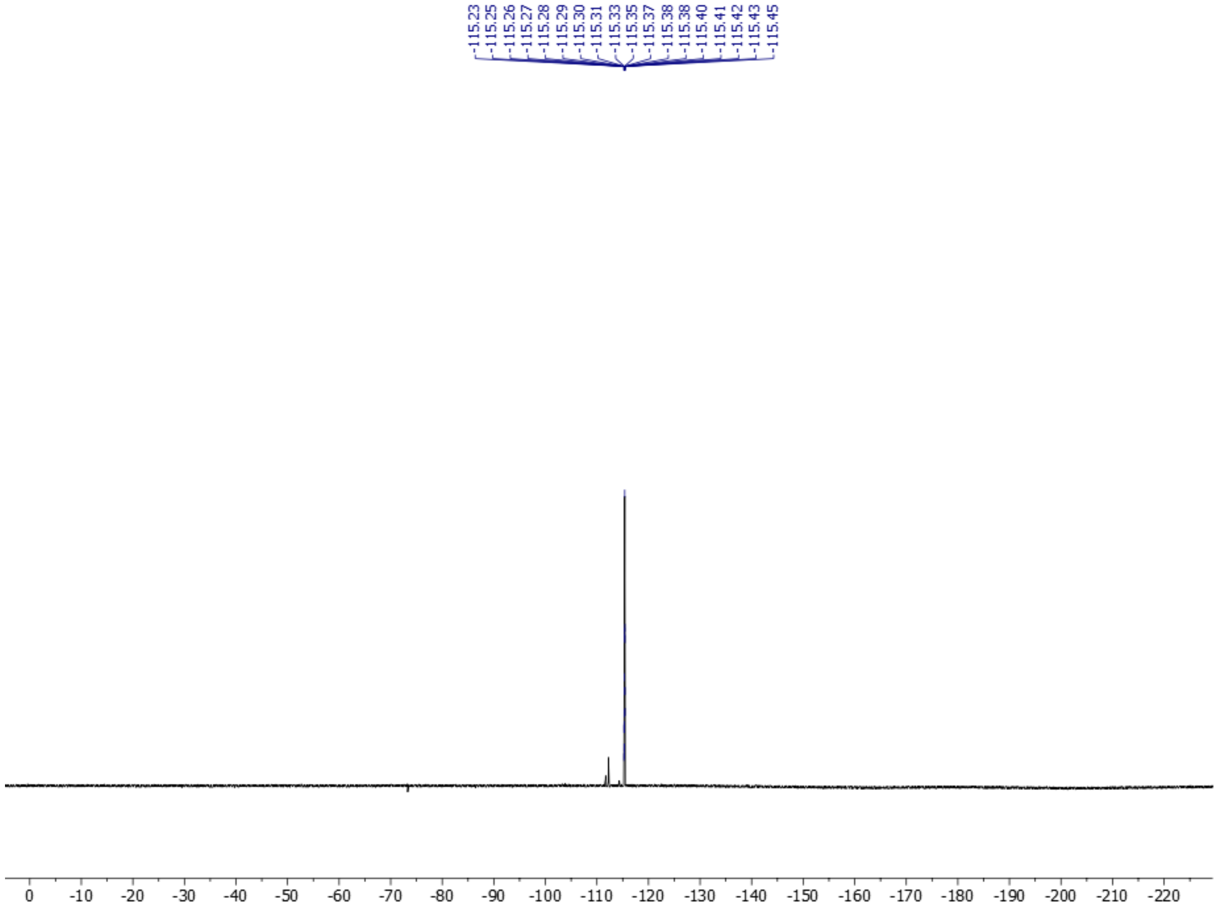

References

[1] L. E. Löffler, M. Buchsteiner, L. R. Collins, F. P. Caló, S. Singha, A. Fürstner, *Helv. Chim. Acta* **2021**, *104*, e2100042.

[2] C. Tortoreto, D. Rackl, H. M. L. Davies, *Org. Lett.* **2017**, *19*, 770.

[3] L. Fu, J. D. Mighion, E. A. Voight, H. M. L. Davies, *Chem. Eur. J.* **2017**, *23*, 3272.

[4] M. Dudic, I. Císarova, J. Michl, *J. Org. Chem.* **2012**, *77*, 68.

[5] S. Furukawa, T. Yasuda, *J. Mater. Chem. A* **2019**, *7*, 14806.

[6] S. Singha, M. Buchsteiner, G. Bistoni, R. Goddard, A. Fürstner, *J. Am. Chem. Soc.* **2021**, *143*, 5666.

[7] K. Liao, S. Negretti, D. G. Musaev, J. Bacsa, H. M. L. Davies, *Nature* **2016**, *533*, 230-234.

[8] H. Suematsu, T. Katsuki, *J. Am. Chem. Soc.* **2009**, *131*, 14218.

[9] D. M. Guptill, H. M. L. Davies, *J. Am. Chem. Soc*. **2014**, *136*, 17718-17721.
